# Synthesis, Functionalization, and Reactivity of Vinyl Sulfondiimidamides

**DOI:** 10.1002/anie.9885717

**Published:** 2026-03-28

**Authors:** Katherine G. Rodden, Agamemnon E. Crumpton, Samuel E. Dalton, Michael A. Clegg, Michael C. Willis

**Affiliations:** ^1^ Department of Chemistry Chemistry Research Laboratory University of Oxford Oxford UK; ^2^ Discovery Chemistry, MSD (UK) Limited London UK

**Keywords:** covalent warheads, cysteine, electrophiles, sulfur functional groups, synthetic methods

## Abstract

Acrylamides are the dominant electrophilic warheads used in bioactive covalent inhibitors. A limitation of acrylamides and related unsaturated sulfonamides is the narrow scope to modulate their electrophilicity, and hence reactivity, through simple structural modification. Here, we show that vinyl sulfondiimidamides are effective electrophilic motifs for reaction with both sulfur‐ and nitrogen‐based biologically relevant nucleophiles. We demonstrate that the electrophilicity of these new reagents can be tuned through variation of the imidic N‐substituents. Vinyl sulfondiimidamides are prepared via a short sequence that features a Cope elimination as the key alkene‐forming step. A broad range of N‐substituents can be installed.

## Graphical abstract

Combining unsymmetrical sulfurdiimide reagents (R‐NSN‐R^1^) and Grignard reagents, followed by oxidative amination, delivers a series of vinyl sulfondiimidamides. Reactivity with cysteine and lysine derivatives is shown, and the cysteine reactivity is mapped in detail. Variation of the two imidic N‐substituents can be used to tune reactivity of these new reagents, positioning them as promising groups for exploration as novel covalent warheads.

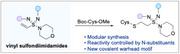

1

Interest in the development of covalent inhibitors has increased in recent years, with the success of drugs such as ibrutinib, lazertinib, adagrasib, and ritlecitinib highlighting the utility of this approach in medicinal chemistry (Scheme 1) [1, 2, 3, 4, 5]. Acrylamides are the most commonly employed warheads in these inhibitors [6, 7, 8]. Vinyl sulfonamides have also been used and shown to target cysteine [9] and lysine [10] residues. Despite the success of acrylamide‐based inhibitors, opportunities to modulate their reactivity through structural modification remain limited [11, 12, 13].

**SCHEME 1:**
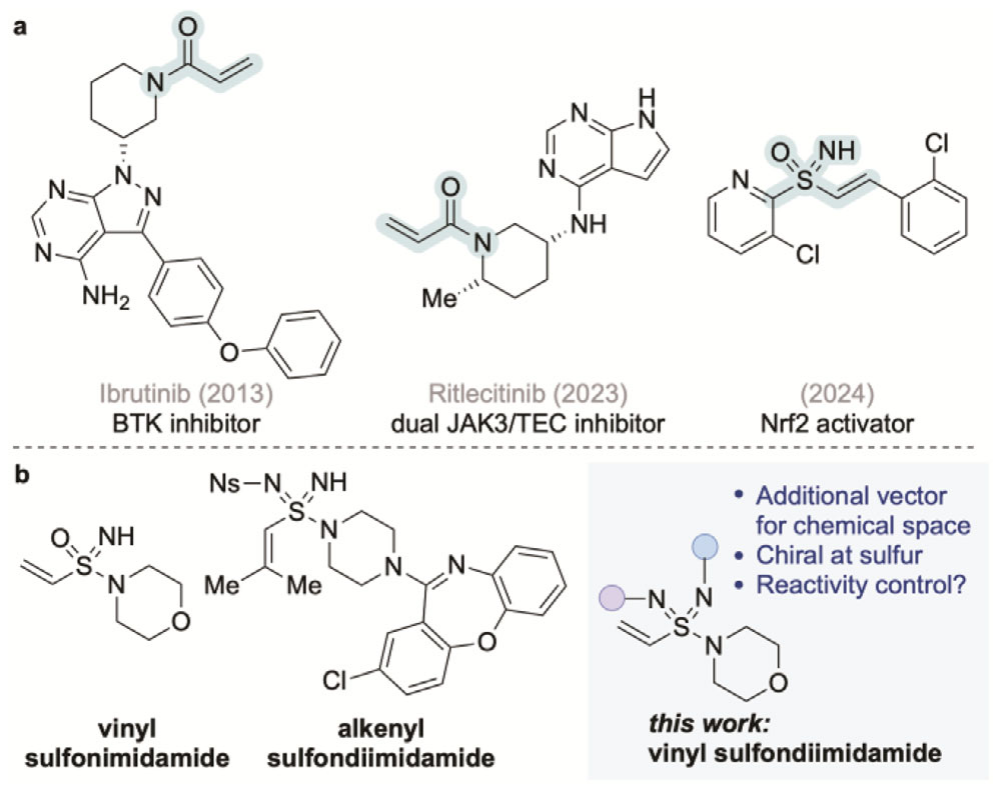
(a) Bioactive acrylamides and alkenyl sulfoximines; (b) vinyl sulfondiimidamides, alkenyl sulfondiimidamides, and this work: vinyl sulfondiimidamides.

We have recently reported the synthesis of a series of vinyl sulfondiimidamides [14] and explored their reactivity with sulfur‐ and nitrogen‐based biologically relevant nucleophiles. Importantly, we demonstrated that variation of the imidic N‐substituent could be used to modulate the electrophilicity of these compounds. The aza‐analogues of vinyl sulfones, namely vinyl sulfoximines, have recently been investigated as Nrf2 activators [15]. Sulfondiimidamides are a recently validated functional group, and reports from our laboratory [16, 17, 18] and others [19, 20, 21] have demonstrated that molecules featuring this group are synthetically accessible. Sulfondiimidamides offer several potential advantages, including additional vectors for chemical space exploration (via substitution at the two imidic N atoms) and the opportunity to control the acidity and basicity of the imidic NH groups. Sulfondiimidamides can also be chiral at sulfur, providing the possibility of further modulating interactions with biological targets. We were therefore drawn to exploiting these features in the design of new covalent warheads, specifically through exploration of vinyl sulfondiimidamide chemistry. Although the known syntheses of sulfondiimidamides include one example of a substituted alkenyl variant [17], an unsubstituted vinyl sulfondiimidamide has not been reported and represents a new class of conjugate acceptor.

To prepare the targeted vinyl sulfondiimidamides, we employed a modified procedure to that previously reported by our laboratory (Scheme 2) [17]. The synthesis begins with the addition of a vinyl Grignard reagent to the sulfurdiimide reagent TMS‐NSN‐TIPS (**1**), followed by direct TMS cleavage and N‐functionalization, to afford sulfinamidines **3**. Oxidative amination of the sulfinamidines (**3**) mediated by PhI(OAc)_2_ and DBU resulted in the addition of morpholine at both the 1,2‐ and 1,4‐positions, due to the electrophilicity of the alkene substituent, providing β‐amino sulfondiimidamides **4**. Our synthesis exploits this dual reactivity, with installation of the alkene achieved via a final‐step amine oxidation/elimination (see Scheme 3). Application of the oxidative amination procedure to vinyl sulfinamidines bearing various *N*‐substituents including, sulfonyl (**3a**, **3b**), carbonyl (**3c**) and carbamate (**3d**, **3e**) groups, proceeded smoothly to afford the corresponding β‐amino sulfondiimidamides (**4**) in good yields. We also applied this transformation to several different amines, including Boc‐piperazine **4f**, an acyclic amine **4g**, and tetrahydroisoquinoline **4h**.

**SCHEME 2:**
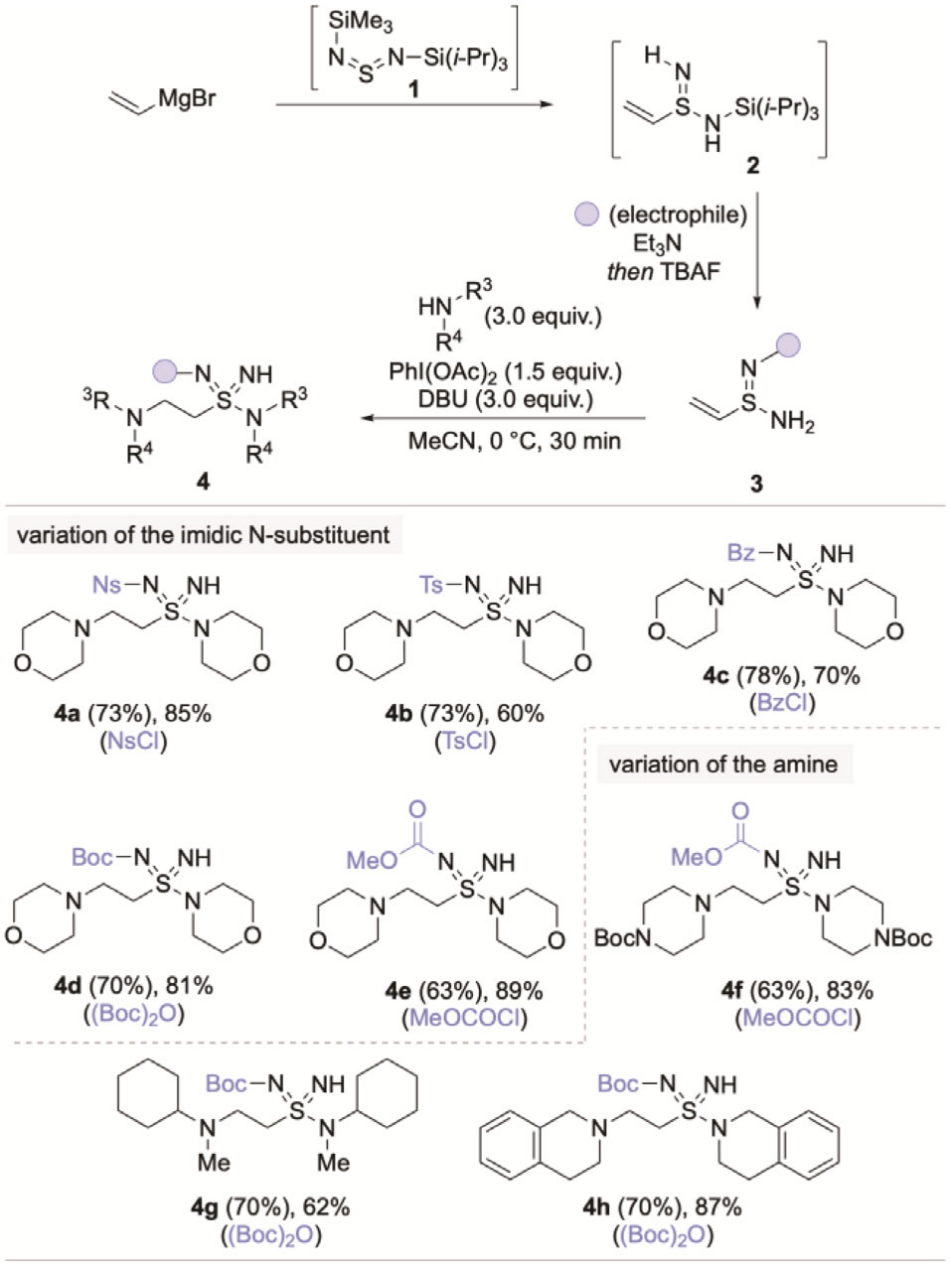
Synthesis of β‐amino sulfondiimidamides **4**. Yields in parentheses are for sulfinamidines **3**; final yields are for sulfondiimidamides **4**. See Supporting Information for full reaction details.

**SCHEME 3:**
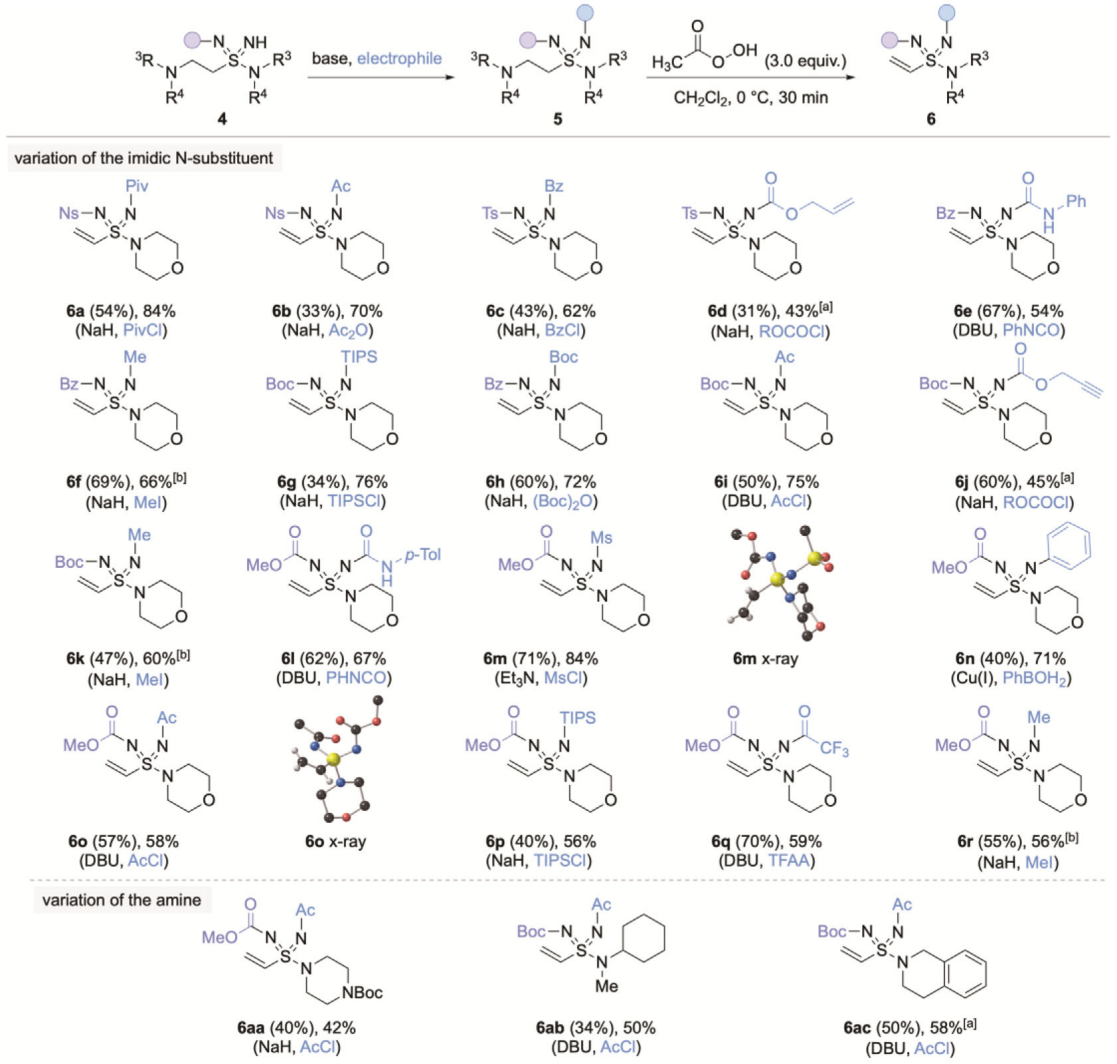
Imidic functionalization followed by amine oxidation/elimination for the synthesis of vinyl sulfondiimidamides **6**. ^(a)^ 1.0 equiv. of peracetic acid used. ^(b)^ Followed by NaHCO_3_ (1.5 equiv.), acetone: water (1:1), rt, 2 h. Yields in parentheses are for sulfondiimidamides **5**; final yields are for vinyl sulfondiimidamides **6**. See Supporting Information for full reaction details.

Functionalization of the second imidic N‐H, followed by β‐amine elimination, was then used to deliver the targeted vinyl sulfondiimidamides (Scheme 3): Treatment of sulfondiimidamides **4** with base and the appropriate electrophile afforded a range of *N*‐difunctionalized products including, acyl (**5a**–**c**), carbamate (**5h**), and urea (**5e**) derivatives. Carbamates featuring reactive functional groups such as an alkene (**5d**) and an alkyne (**5j**), which can be used for copper‐catalyzed azide‐alkyne cycloaddition (CuAAC), could also be prepared. Sulfondiimidamides substituted with electron‐donating alkyl (**5f**) and silyl groups (**5g**) were also synthesized. An N‐aryl substituent was introduced using a Cu‐catalyzed Chan‐Lam coupling (**5n**). With a range of structurally diverse β‐amino‐sulfondiimidamides available, elimination of the amine was achieved using an excess of peracetic acid to form an intermediate N‐oxide, which triggered a Cope elimination [22, 23]. Using this method, the β‐amino sulfondiimidamides **5** were converted to the corresponding vinyl sulfondiimidamides in generally good yields [24]. For the N‐alkyl‐substituted examples (**6f, 6k,** and **6r**), 1,4‐addition of acetic acid was observed under the reaction conditions, presumably due to the greater basicity of the relevant imidic N‐atom, and an additional base‐mediated elimination step was required to generate the alkene unit.

We next developed syntheses of α‐ and β‐methyl alkenyl sulfondiimidamides (Scheme 4). For these targets, we were able to use our original procedure [17], allowing alkenyl N‐H sulfondiimidamides (**7**) to be obtained directly from the PhI(OAc)_2_ mediated oxidative amination, due to the lower electrophilicity of the methyl‐substituted alkenes. This approach had the advantage of removing the oxidation/elimination step required in the vinyl series from the synthetic route. Functionalization of the imidic NH provided the desired alkenyl sulfondiimidamides (**8**).

**SCHEME 4:**
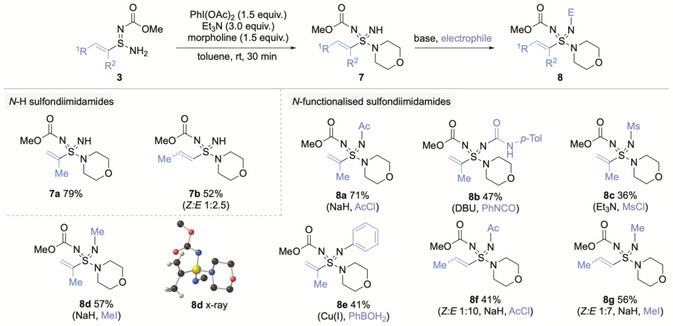
Synthesis of N‐functionalized α‐ and β‐methyl alkenyl sulfondiimidamides **8**. See Supporting Information for full reaction details.

Although the synthetic route detailed in Schemes 2 and 3 allows access to a range of functionalized vinyl sulfondiimidamides, it requires the use of three equivalents of the amine that ultimately features as the amidic group; for complex and/or synthetically valuable amines, this use of excess material is problematic. To overcome this issue, we showed that sulfinamidine **9**, which bears a simple sacrificial amine preinstalled at the β‐position, is a competent substrate in the PhI(OAc)_2_ mediated oxidative amination step (Scheme 5). Thus, treatment of sulfinamidine **9** with amine **10** (a component of buspirone) and PhI(OAc)_2_/Et_3_N provided the desired sulfondiimidamide **11** in 81% yield. *N*‐Functionalization with phenyl isocyanate and oxidation/elimination of the pyrrolidine group gave the targeted vinyl sulfondiimidamide **13** (via intermediate **12**).

**SCHEME 5:**
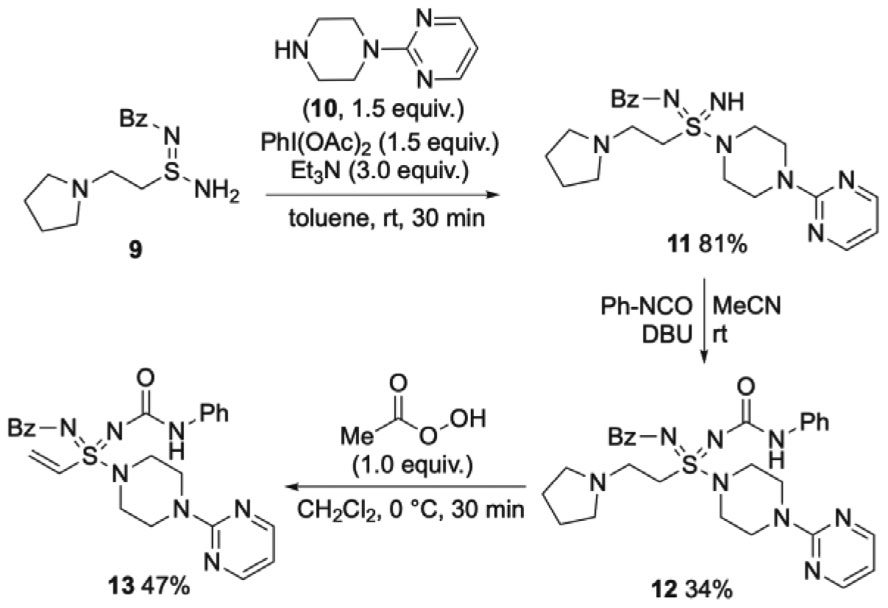
Conversion of β‐amino sulfinamidine **9** into vinyl sulfondiimidamide **13**.

We began our reactivity studies using two model vinyl sulfondiimidamide substrates in combination with a series of protected amino acid derivatives as nucleophiles (Scheme 6). The sulfondiimidamides used were **6i**, featuring two electron‐withdrawing N‐substituents (Boc and Ac), and **6k**, which we reasoned should be less reactive as it bears an N‐Boc group in combination with an electron‐donating N‐methyl substituent. When vinyl sulfondiimidamide **6i**, featuring two electron‐withdrawing N‐substituents, was subjected to protected amino acids under basic conditions, efficient reactivity with cysteine and lysine was observed; reaction with histidine was also detected, and delivered a mixture of regioisomers. Some reactivity with tyrosine was also seen, although in this case full conversion was not achieved. No reaction was observed with serine, tryptophan, or aspartic acid derivatives. When vinyl sulfondiimidamide **6k**, featuring the N‐Boc/*N*‐methyl combination of N‐substituents, was used, similar reactivity with cysteine and lysine was observed; however, no reactivity with histidine or tyrosine was detected. Use of the more reactive sulfondiimidamide **6i** in a competition experiment with both cysteine and lysine nucleophiles showed moderate selectivity for reaction with cysteine.

**SCHEME 6:**
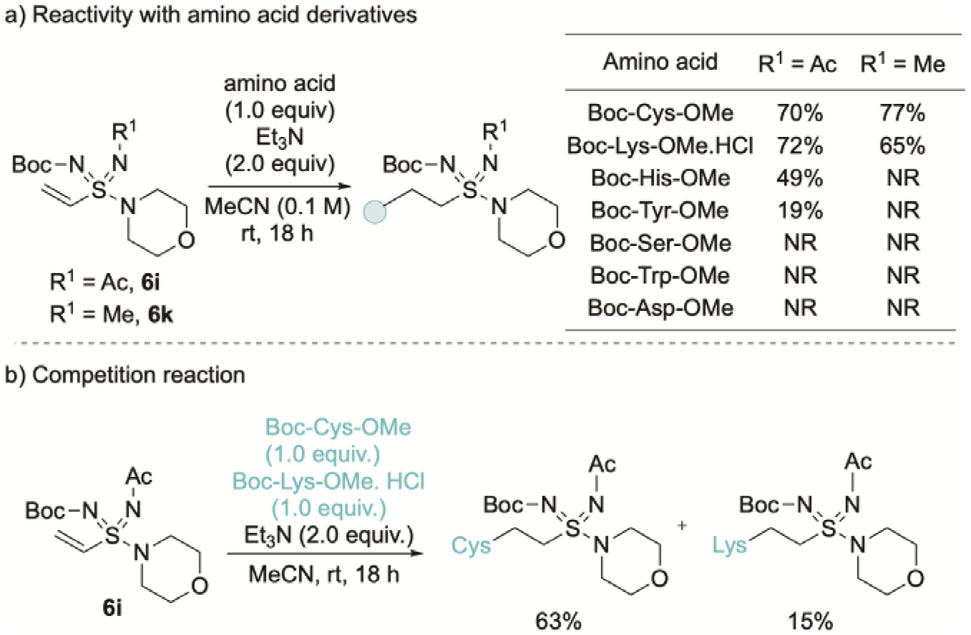
Reactivity of vinyl sulfondiimidamides **6i** and **6k** with protected amino acids.

Next, we explored the effect of the imidic N‐substituent on reactivity in more detail and set out to measure half‐lives for the reaction of a series of vinyl sulfondiimidamides with a fixed nucleophile, Boc‐Cys‐OMe (Scheme 7). The half‐life (*t*_1/2_) was determined using ^1^H NMR spectroscopy by monitoring consumption of the vinyl group (see the Supporting Information for full experimental details). It was apparent that the reactions of vinyl sulfondiimidamides were too rapid for the half‐life to be accurately determined by ^1^H NMR experiments (see Supporting Information). To achieve slower reaction rates, we moved to using α‐ and β‐alkenyl sulfondiimidamides. To benchmark these results against known electrophiles, half‐lives were measured for vinyl sulfonamide **13** and acrylamide **14**, which gave *t*_1/2_ values of 562 and 15 720 s, respectively, using 1.0 equivalent of cysteine. When α‐methyl *N*‐H sulfondiimidamide **7a** was employed with 1.0 equivalents of cysteine, we observed a half‐life of 52 859 s, which is significantly slower than both the acrylamide **13** and the vinyl sulfonamide **14**. Due to this slower reaction rate, reaction of N–H sulfondiimidamide **7a** with 10 equivalents of cysteine was carried out, and the half‐life was calculated using pseudo‐first‐order kinetics, giving a *t*_1/2_ of 8199 s. α‐Methyl alkenyl sulfondiimidamides bearing electron‐withdrawing groups such as *N*‐Ms **8c** (*t*_1/2_ = 25 s), *N*‐Ac **8a** (*t*_1/2_ = 218 s) and *N*‐urea **8b** (*t*_1/2_ = 287 s) displayed significantly faster reactivity. The half‐life of conjugate addition to these substrates was correlated with the β‐carbon ^13^C NMR chemical shift. Substrates bearing electron‐donating substituents displayed markedly decreased reactivity. For example, *N*‐phenyl derivative **8e** exhibited a similar half‐life to the *N*‐H derivative **7a** when using 10.0 equivalents of cysteine (*t*_1/2_ = 7567 vs. 8199 s). The introduction of an *N*‐methyl group in substrate **8d** had a pronounced impact on reactivity, decreasing the half‐life to 25 945 s with 10 equivalents of cysteine. This demonstrates that modification of the imidic substituent can be used to modulate the reactivity of these electrophiles. In all cases, β‐methyl sulfondiimidamides were more reactive than the corresponding α‐methyl derivatives, which is in agreement with findings for the sulfonimidamide analogues [14]. The β‐methyl sulfondiimidamides used were predominantly *E*‐isomers, although the exact ratio varied between substrates. *N*‐Ac substrate **8f** showed substantially faster reactivity than *N*‐H or *N*‐methyl derivatives.

**SCHEME 7:**
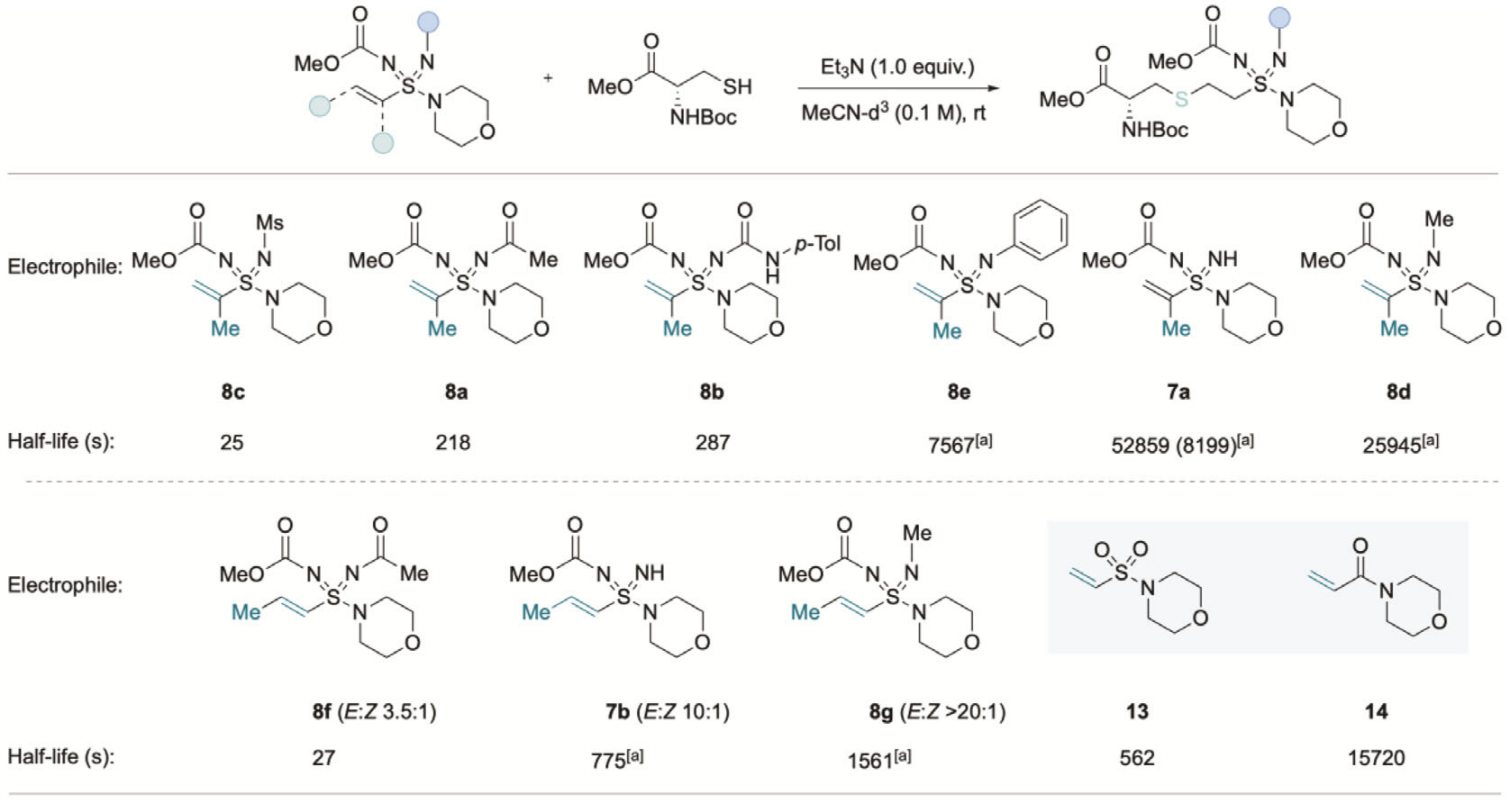
Reactivity and half‐life studies of alkenyl sulfondiimidamides with N‐Boc‐Cys‐OMe. ^(a)^ 10.0 equiv. of N‐Boc‐Cys‐OMe used.

In conclusion, we have demonstrated an efficient route to vinyl sulfondiimidamides that employs a Cope elimination as the key alkene‐forming transformation. The synthesis enables the preparation of vinyl sulfondiimidamides featuring a variety of substituents on both imidic N‐positions. These new electrophilic fragments show good reactivity with both cysteine‐ and lysine‐derived nucleophiles. Analysis of their reactions with a cysteine nucleophile demonstrates that reactivity depends on the identity of the imidic N‐substituents and substitution on the alkene. Variation of these two factors allowed alkenyl sulfondiimidamides with reactivities both above and below that of the two benchmarked substrates to be identified. Given the range of accessible reactivities, combined with their straightforward assembly, we anticipate that vinyl sulfondiimidamides will be of broad utility in medicinal chemistry and chemical biology applications.

## Conflicts of Interest

The authors declare no conflicts of interest.

## Supporting information




**Supporting File 1**: anie71913‐sup‐0001‐SuppMat.pdf.

## Data Availability

The data that supports the findings of this study are available in the supporting informaton of this article.
