# Supplementary material for: Synthesis, Functionalization, and Reactivity of Vinyl Sulfondiimidamides
Source: Angew Chem Int Ed Engl. 2026 Mar 28;65(19):e9885717. doi: 10.1002/anie.9885717 (PMC13134591; doi:10.1002/anie.9885717)
Supplement: Supplementary file 1 — Supporting File 1: anie71913‐sup‐0001‐SuppMat.pdf. [file ANIE-65-e9885717-s001.pdf]

Supporting Information

Synthesis, Functionalization and Reactivity of Vinyl Sulfondiimidamides

Katherine G. Rodden,[a] Agamemnon E. Crumpton,[a] Samuel E. Dalton,[b] Michael A. Clegg,[b] Michael C. Willis [a,*]

[a] Department of Chemistry, University of Oxford, Chemistry Research Laboratory, Mansfield Road, Oxford, OX1 3TA, UK.

[b] Discovery Chemistry, MSD (UK) Limited, 120 Moorgate, London EC2M 6UR, UK.

michael.willis@chem.ox.ac.uk

Table of Contents

1 General experimental considerations S2

2 Experimental procedures and data S4

2.1 Synthesis of vinyl sulfondiimidamides S4

2.1.1 Synthesis of *N*-sulfinyltriisopropylamine S4

2.1.2 General procedure A – synthesis of primary sulfinamidines (3) S4

2.1.3 General procedure B – synthesis of β-amino sulfondiimidamides (4) S9

2.1.4 *N*-functionalisation of β-amino sulfondiimidamides S14

2.1.5 General procedure C – synthesis of vinyl sulfondiimidamides (6) S29

2.2 Synthesis of alkenyl sulfondiimidamides S40

2.2.1 General Procedure D – Synthesis of N-H alkenyl sulfondiimidamides (7) S40

2.2.2 *N*-functionalisation of alkenyl sulfondiimidamides S42

2.3 Synthesis of vinyl sulfondiimidamides from β-amino sulfinamidine S47

2.4 General procedure E – conjugate addition reactions with amino acid derivatives S50

2.5 Procedure for competition reactions of Lys- and Cys- derivatives S54

2.6 General procedure F – determination of half-life for the conjugate addition of Boc-Cys-OMe S55

3. Single Crystal X-ray Diffraction S63

4. References S67

5. NMR Spectra S68

1 General experimental considerations

Handling techniques: Unless otherwise stated, all reactions were conducted under an atmosphere of nitrogen with anhydrous solvents using standard Schlenk techniques. Glassware was dried in an oven at 200 ˚C and allowed to cool to room temperature under a positive pressure of nitrogen before use. Cooling of reaction mixtures to 0 ˚C was achieved using an ice-water bath. Cooling of reaction mixtures to -78 ˚C or -30 ˚C was achieved using an acetone-dry ice bath. 'Room temperature' refers to an ambient temperature of 21 ± 2 ˚C.

Reagents: Unless otherwise stated, all chemicals were purchased from commercial sources (Sigma-Aldrich, Fluorochem, Fisher Scientific, Alfa-Aesar or Apollo Scientific and were used without further purification. Peroxyacetic acid was purchased from Thermo Scientific and was used as a ca. 35wt% solution in diluted acetic acid, stabilised. Anhydrous solvents were purified by filtration through dried alumina columns using the University of Oxford internal supplies and dried through $CaCl_2$ drying columns. 'Petrol' refers to the fraction of petroleum ether which boils in the range 40-60 ˚C.

Chromatography: Thin-layer chromatography (TLC) was performed on Merck silica gel 60 F_{254} pre-coated aluminium backed TLC sheets with visualisation under a UV lamp (λ_{max} = 254 nm) and/or by staining with $KMnO_4$ solution. Flash column chromatography was performed using Merck silica gel 60 (230-400 mesh) with the solvent system indicated in parenthesis.

NMR spectroscopy: ^{1}H NMR spectra were recorded on Bruker AVIII400 spectrometer (400 MHz), Bruker AVIIIHD 500 (500 MHz) and Bruker NEO 600 (600 MHz) spectrometers. ^{13}C NMR spectra were recorded on Bruker AVIII400 (101 MHz), Bruker AVIIIHD 500 (126 MHz) and Bruker NEO 600 (151 MHz) spectrometers. ^{19}F NMR spectra were recorded on Bruker AVIII400 (377 MHz), or Bruker AVIIIHD 500 (476 MHz) spectrometers. All reported ^{1}H and ^{13}C chemical shifts (δ_H, δ_C) are referenced to the residual signal of the deuterated solvent ($CDCl_3$: δ_H = 7.26 ppm, δ_C = 77.16 ppm; DMSO-d^6: δ_H = 2.50 ppm, δ_C = 39.52 ppm; C_6D_6: δ_H = 7.16 ppm, δ_C = 128.06 ppm; CD_3OD: δ_H = 3.31 ppm, δ_C = 49.00 ppm; CD_3CN: δ_H = 1.94 ppm, δ_C = 1.32, 118.26 ppm). ^{19}F chemical shifts (δ_F) are referenced externally to $CFCl_3$ (δ_F = 0.0 ppm). Chemical shifts (δ) are reported in parts per million (ppm) to the nearest

0.01 ppm for ^{1}H NMR, and 0.1 ppm for ^{13}C and ^{19}F NMR. Coupling constants (*J*) are reported in Hertz (Hz). Multiplicities are reported as following: s (singlet), d (doublet), t (triplet), q (quartet), pent. (pentet), m (multiplet), br. (broad signal), app. (apparent).

IR Spectroscopy: Infrared spectra were recorded on a Bruker Tensor 27 Fourier Transform spectrometer with an internal range 600-4000 cm^{-1} and all absorption maximum (ν_{max}) are given in wavenumbers (cm^{-1}).

Melting point: Melting points were recorded in degrees Celsius (˚C) a Reichert melting point apparatus and are reported uncorrected.

Mass Spectrometry: High resolution mass spectrometry (HRMS) measurements were recorded on an ACQUITY I-Class PLUS UPLC System (Waters, Milford, MA, USA) coupled to a ACQUITY RDa mass spectrometer (Waters, Milford, MA, USA) equipped with an electrospray ionisation (ESI) probe or on a Bruker Daltronics MicroTOF (ESI) spectrometer through ESI by the mass spectrometry service at the Chemistry Research Laboratory at the University of Oxford. m/z values are reported in Daltons (Da) and high-resolution values are calculated to four decimal places from the molecular formula. Samples for mass spectra were prepared in 1 mg/mL solution in MeCN or MeOH (HRMS-ESI).

2 Experimental procedures and data

2.1 Synthesis of vinyl sulfondiimidamides

2.1.1 Synthesis of *N*-sulfinyltriisopropylamine

N-Sulfinyltriisopropylamine (TIPS-NSO) was prepared following the procedure reported in the literature.[1]

2.1.2 General procedure A – synthesis of primary sulfinamidines (3)

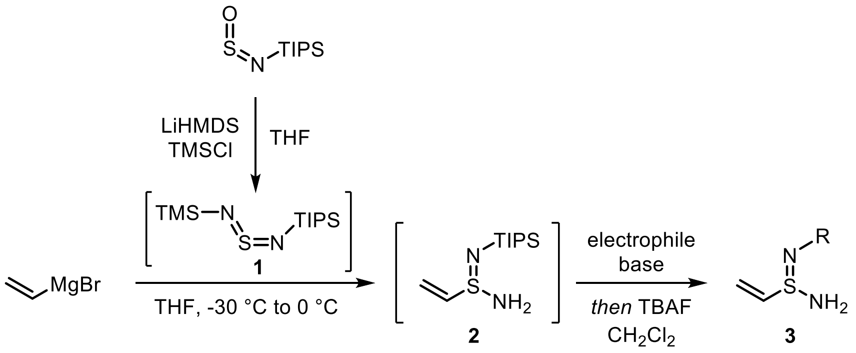

This procedure was adapted from the literature.[2] An oven-dried round-bottom flask containing TIPS-NSO (1.0 equiv.) was sealed and subjected to three N_2 evacuation/refill cycles before anhydrous THF (TIPS-NSO conc. 0.5 M) was added. The solution was cooled to −30 °C and then LiHMDS solution (1.0 M in THF, 1.0 equiv.) was added. The reaction was stirred at −30 °C for 5 min before being warmed to 0 °C and stirred for 5 min at the same temperature. TMSCl (1.0 equiv.) was then added and the reaction was stirred at 0 °C for 10 min. Then vinyl magnesium bromide solution (1.0 M in THF, 1.2 equiv.) was added and the reaction was stirred at 0 °C for another 10 min before being diluted with EtOAc (50 mL) and quenched with sat. aq. tetrasodium EDTA solution (100 mL). The aqueous layer was separated and extracted with EtOAc (3 × 20 mL). The combined organic extracts were dried over anhydrous Na_2SO_4, filtered and concentrated *in vacuo*. This crude mixture was then dissolved in anhydrous CH_2Cl_2 (TIPS-NSO conc. 0.2 M) and cooled to 0 °C. Et_3N (1.2 equiv.) and the corresponding electrophile (1.0 – 1.2 equiv.) were then added. The reaction was stirred at 0 °C or rt for the specified time prior to the addition of TBAF solution (1.0 M in THF, 1.1 equiv.) at 0 °C. The reaction mixture was stirred for 10 min at 0 °C and then diluted with EtOAc (30 mL) and sat. aq. NaCl solution (30 mL). The aqueous phase was extracted with EtOAc (3 × 20 mL). The combined organic extracts were then dried over anhydrous Na_2SO_4, filtered and concentrated

in vacuo. The resulting primary sulfinamidine was then purified by flash column chromatography with the appropriate solvent system.

Primary sulfinamidine 3a

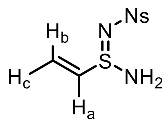

Following general procedure A, using TIPS-NSO (1.31 g, 6.00 mmol, 1.0 equiv.), THF (12.0 mL), LiHMDS solution (6.00 mL, 1.00 M in THF, 6.00 mmol, 1.0 equiv.), TMSCl (0.76 mL, 6.00 mmol, 1.0 equiv.) and vinylmagnesium bromide solution (8.00 mL, 0.90 M in THF, 7.20 mmol, 1.2 equiv.). The crude residue was combined with CH_2Cl_2 (30 mL), Et_3N (1.00 mL, 7.20 mmol, 1.2 equiv.), NsCl (1.33 g, 6.00 mmol, 1.0 equiv.) and stirred at 0 °C for 20 min before TBAF solution (6.60 mL, 1.00 M in THF, 6.60 mmol, 1.1 equiv.) was added. Purification by flash column chromatography (SiO_2, CH_2Cl_2/EtOAc, 5:1 to 4:1) afforded the desired product **3a** as a white solid (1.20 g, 73%).

***R*$_f$**= 0.55 (EtOAc); **m.p.** decomp at 172 ˚C (CH_2Cl_2); **^{1}H NMR** (400 MHz, DMSO-*d*^{6}): δ 8.33 (d, 2H, *J* = 8.8 Hz, Ar*H*), 8.01 (d, 2H, *J* = 8.8 Hz, Ar*H*), 6.75 – 6.70 (m, 3H, C*H$_a$,* N*H$_2$*), 6.03 (dd, 1H, *J* = 9.3, 0.95 Hz, C*H$_c$*), 5.98 (dd, 1H, *J* = 16.0, 0.95 Hz, C*H$_b$*); **^{13}C NMR** (101 MHz, DMSO-*d*^{6}): δ 150.8, 148.7, 136.0, 127.2, 125.0, 124.2; **IR** (ν_{max}, cm^{-1}) 1517, 1397, 1288, 1142, 1108, 980, 860, 794, 683; **HRMS** (ESI): *m/z* calcd for $C_8H_{10}N_3O_4S_2^+$: 276.0107 [M+H]$^+$; found: 276.0109.

Primary sulfinamidine 3b

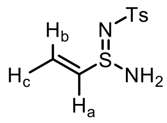

Following general procedure A, using TIPS-NSO (438.8 mg, 2.00 mmol, 1.0 equiv.), THF (4.0 mL), LiHMDS solution (2.00 mL, 1.00 M in THF, 2.00 mmol, 1.0 equiv.), TMSCl (0.25 mL, 2.00 mmol, 1.0 equiv.) and vinylmagnesium bromide solution (2.40 mL, 1.00 M in THF, 2.40 mmol, 1.2 equiv.). The crude residue was combined with CH_2Cl_2 (10 mL), Et_3N (0.33 mL, 2.40 mmol, 1.2 equiv.) and 4-Toluenesulfonyl chloride (458 mg, 2.40 mmol, 1.2 equiv.) and stirred at 0 °C for 30 min before TBAF solution (2.20 mL, 1.00 M in THF, 2.20 mmol, 1.1 equiv.)

was added. Purification by flash column chromatography (SiO_2, CH_2Cl_2/EtOAc, 1:1) afforded the desired product **3b** as a white solid (355 mg, 73%).

***R*f**= 0.36 (EtOAc); **m.p.** decomp at 148 ˚C (CH_2Cl_2); **^{1}H NMR** (400 MHz, DMSO-*d*^{6}): δ 7.64 (d, 2H, *J* = 8.0 Hz, Ar*H*), 7.29 (d, 2H, *J* = 7.9 Hz, Ar*H*), 6.68 (dd, 1H, *J* = 16.0, 9.2 Hz, C*Ha*), 6.59 (s, 2H, N*H*2), 5.98 (d, 1H, *J* = 9.2 Hz, C*Hc*), 5.93 (d, 1H, *J* = 16.0 Hz, C*Hb*), 2.34 (s, 3H, C*H*3); **^{13}C NMR** (101 MHz, DMSO-*d*^{6}): δ 142.6, 140.9, 136.5, 129.1, 125.6, 124.5, 20.9; **IR** (ν_{max}, cm^{-1}) 1279, 1137, 1086, 976, 777, 668; **HRMS** (ESI): *m/z* calcd for $C_9H_{13}N_2O_2S_2^+$: 245.0413 [M+H]$^+$; found: 245.0408.

Primary sulfinamidine 3c

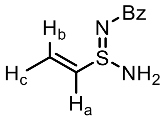

Following general procedure A, using TIPS-NSO (438.8 mg, 2.00 mmol, 1.0 equiv.), THF (4.0 mL), LiHMDS solution (2.00 mL, 1.00 M in THF, 2.00 mmol, 1.0 equiv.), TMSCl (0.25 mL, 2.00 mmol, 1.0 equiv.) and vinylmagnesium bromide solution (2.40 mL, 1.00 M in THF, 2.40 mmol, 1.2 equiv.). The crude residue was combined with CH_2Cl_2 (10 mL), Et_3N (0.33 mL, 2.40 mmol, 1.2 equiv.) and benzoyl chloride (0.28 mL, 2.40 mmol, 1.2 equiv.) and stirred at rt for 18 h before TBAF solution (2.20 mL, 1.00 M in THF, 2.20 mmol, 1.1 equiv.) was added. Purification by flash column chromatography (SiO_2, CH_2Cl_2/EtOAc, 1:1) afforded the desired product **3c** as a white solid (304 mg, 78%).

***R*f**= 0.36 (EtOAc); **m.p.** 98–102 ˚C (CH_2Cl_2); **^{1}H NMR** (400 MHz, DMSO-*d*^{6}): δ 8.01 (d, 2H, *J* = 6.8 Hz, Ar*H*), 7.45 (t, 1H, *J* = 7.1 Hz, Ar*H*), 7.39 (t, 2H, *J* = 7.3 Hz, Ar*H*), 6.81 (dd, 1H, *J* = 9.5, 16.4 Hz, C*Ha*), 6.17 – 6.08 (m, 3H, C*Hb*, N*H*2), 6.02 (dd, 1H, *J* = 9.6 Hz, C*H*c); **^{13}C NMR** (101 MHz, DMSO-*d*^{6}): δ 174.7, 137.2, 136.2, 130.6, 128.3, 127.8, 124.1; **IR** (ν_{max}, cm^{-1}) 1593, 1539, 1329, 1296, 1131, 962, 844, 710; **HRMS** (ESI): *m/z* calcd for $C_9H_{11}N_2OS^+$: 195.0587 [M+H]$^+$; found: 195.0583.

Primary sulfinamidine 3d

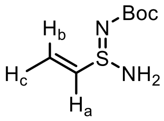

Following general procedure A, using TIPS-NSO (1.10 g, 5.00 mmol, 1.0 equiv.), THF (10.0 mL), LiHMDS solution (5.00 mL, 1.00 M in THF, 5.00 mmol, 1.0 equiv.), TMSCl (0.63 mL, 5.00 mmol, 1.0 equiv.) and vinylmagnesium bromide solution (6.00 mL, 1.00 M in THF, 6.00 mmol, 1.2 equiv.). The crude residue was combined with CH_2Cl_2 (25 mL), Et_3N (0.83 mL, 6.00 mmol, 1.2 equiv.), Di-*tert*-butyl dicarbonate (1.09 g, 5.00 mmol, 1.0 equiv.) and stirred at rt for 18 h before TBAF solution (5.50 mL, 1.00 M in THF, 5.50 mmol, 1.1 equiv.) was added. Purification by flash column chromatography (SiO_2, CH_2Cl_2/EtOAc, 1:1) afforded the desired product **3d** as a white solid (661 mg, 70%).

***R*$_f$**= 0.21 (EtOAc); **m.p.** 102–106 ˚C (CH_2Cl_2); **^{1}H NMR** (400 MHz, $CDCl_3$): δ 6.67 (dd, 1H, *J* = 16.5, 9.6 Hz, CH_a), 6.10 (dd, 1H, *J* = 16.5, 1.1 Hz, CH_b), 5.93 (dd, 1H, *J* = 9.6, 1.0 Hz, CH_c), 4.67 (s, 2H, NH_2), 1.45 (s, 9H, $(CH_3)_3$); **^{13}C NMR** (101 MHz, $CDCl_3$): δ 164.4, 135.9, 125.3, 79.5, 28.5; **IR** (ν_{max}, cm^{-1}) 1623, 1365, 1285, 1249, 1161, 843; **HRMS** (ESI): *m/z* calcd for $C_7H_{15}N_2O_2S^+$: 191.0849 $[M+H]^+$; found: 191.0843.

Primary sulfinamidine 3e

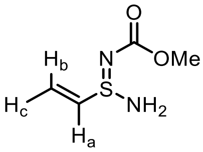

Following general procedure A, using TIPS-NSO (3.29 g, 15.0 mmol, 1.0 equiv.), THF (30.0 mL), LiHMDS solution (15.0 mL, 1.00 M in THF, 15.0 mmol, 1.0 equiv.), TMSCl (1.90 mL, 15.0 mmol, 1.0 equiv.) and vinylmagnesium bromide solution (18.0 mL, 1.00 M in THF, 18.0 mmol, 1.2 equiv.). The crude residue was combined with CH_2Cl_2 (75.0 mL), Et_3N (2.50 mL, 18.0 mmol, 1.2 equiv.), methyl chloroformate (1.16 mL, 15.0 mmol, 1.0 equiv.) and stirred at rt for 18 h before TBAF solution (16.5 mL, 1.00 M in THF, 16.5 mmol, 1.1 equiv.) was added. No aqueous workup was carried out. Direct purification by flash column chromatography (SiO_2, EtOAc) afforded the desired product **3e** as a white solid (1.40 g, 63%).

***R*$_f$**= 0.11 (EtOAc); **m.p.** 62–64 ˚C (CH_2Cl_2); **^{1}H NMR** (400 MHz, $CDCl_3$): δ 6.60 (dd, 1H *J* = 16.5, 9.6 Hz, C*H$_a$*), 6.05 (dd, 1H, *J* = 16.4, 1.0 Hz, C*H$_b$*), 5.89 (dd, 1H, *J* = 9.7, 1.0 Hz, C*H$_c$*), 5.26 (s, 2H, N*H$_2$*), 3.58 (s, 3H, C(O)OC*H$_3$*); **^{13}C NMR** (101 MHz, $CDCl_3$): δ 165.1, 135.5, 125.2, 52.8; **IR** (ν_{max}, cm^{-1}) 1623, 1437, 1267, 946, 858, 790, 732; **HRMS** (ESI): *m/z* calcd for $C_4H_9N_2O_2S^+$: 149.0379 $[M+H]^+$; found: 149.0378.

Primary sulfinamidine 3f

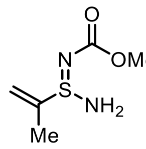

Following general procedure A, using TIPS-NSO (658.3 mg, 3.00 mmol, 1.0 equiv.), THF (6.0 mL), LiHMDS solution (3.00 mL, 1.00 M in THF, 3.00 mmol, 1.0 equiv.), TMSCl (0.38 mL, 3.00 mmol, 1.0 equiv.) and isopropenylmagnesium bromide solution (7.20 mL, 0.50 M in THF, 3.60 mmol, 1.2 equiv.). The crude residue was combined with CH_2Cl_2 (15.0 mL), Et_3N (0.50 mL, 3.60 mmol, 1.2 equiv.), methyl chloroformate (0.23 mL, 3.00 mmol, 1.0 equiv.) and stirred at rt for 18 h before TBAF solution (3.30 mL, 1.00 M in THF, 3.30 mmol, 1.1 equiv.) was added. Purification by flash column chromatography (SiO_2, EtOAc) afforded the desired product **3f** as a white solid (444 mg, 91%).

***R*$_f$**= 0.11 (EtOAc); **m.p.** 68–70 ˚C (CH_2Cl_2); **^{1}H NMR** (400 MHz, $CDCl_3$): δ 5.91 (s, 1H, C*H*), 5.63 (s, 1H, C*H*), 4.73 (s, 2H, N*H$_2$*), 3.64 (s, 3H, C(O)OC*H$_3$*), 2.12 (s, 3H, CC*H$_3$*); **^{13}C NMR** (101 MHz, $CDCl_3$): δ 165.2, 142.7, 121.3, 52.9, 17.2; **IR** (ν_{max}, cm^{-1}) 1621, 1438, 1272, 1192, 1100, 944, 863, 789, 735; **HRMS** (ESI): *m/z* calcd for $C_5H_{11}N_2O_2S^+$: 163.0536 $[M+H]^+$; found: 163.0528.

Primary sulfinamidine 3g

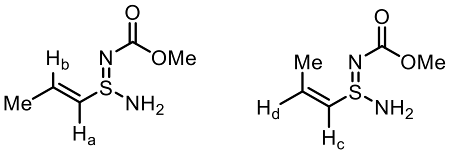

Following general procedure A, using TIPS-NSO (658.3 mg, 3.00 mmol, 1.0 equiv.), THF (6.0 mL), LiHMDS solution (3.00 mL, 1.00 M in THF, 3.00 mmol, 1.0 equiv.), TMSCl (0.38 mL, 3.00 mmol, 1.0 equiv.) and 1-propenylmagnesium bromide solution (7.20 mL, 0.50 M in THF, 3.60 mmol, 1.2 equiv.). The crude residue was combined with CH_2Cl_2 (15.0 mL), Et_3N (0.50 mL,

3.60 mmol, 1.2 equiv.) and methyl chloroformate (0.28 mL, 3.00 mmol, 1.0 equiv.) and stirred at rt for 18 h before TBAF solution (3.30 mL, 1.00 M in THF, 3.30 mmol, 1.1 equiv.) was added. Purification by flash column chromatography (SiO_2, EtOAc/MeOH, 1:0 to 25:1) afforded **3g** as a mixture of isomers (*E*:*Z* = 1.2:1.0) as a white solid (426 mg, 88%).

***R*f**= 0.11 (EtOAc); **m.p.** 60–64 ˚C (CH_2Cl_2); **^{1}H NMR** (400 MHz, $CDCl_3$) *Trans isomer:* δ 6.54 (dq, 1H, *J* = 6.6, 13.4 Hz, CH_b), 6.42 (dd, 1H, *J* = 1.9, 15.1, CH_a), 4.81 (s, 2H, NH_2), 3.64 (s, 3H, C(O)OCH_3), 1.90 (dd, 3H, *J* = 1.5, 6.6 Hz,CHCH_3); *Cis isomer:* δ 6.65 (dd, 1H, *J* = 1.94, 9.7 Hz, CH_c), 6.23 (dq, 1H, *J* = 7.1, 9.6 Hz, CH_d), 4.81 (s, 2H, NH_2), 3.64 (s, 3H, C(O)OCH_3), 1.98 (dd, 3H, *J* = 1.7, 7.2 Hz, CHCH_3); **^{13}C NMR** (101 MHz, $CDCl_3$) *Trans isomer*: δ 165.6, 139.4, 127.9, 52.9, 17.9; *Cis isomer*: δ 165.3, 137.7, 129.7, 52.9, 15.4; **IR** (ν_{max}, cm^{-1}) 1616, 1437, 1263, 1191, 1098, 947, 857, 791, 713; **HRMS** (ESI): *m/z* calcd for $C_5H_{11}N_2O_2S^+$: 163.0536 $[M+H]^+$; found: 163.0533.

2.1.3 General procedure B – synthesis of β-amino sulfondiimidamides (4)

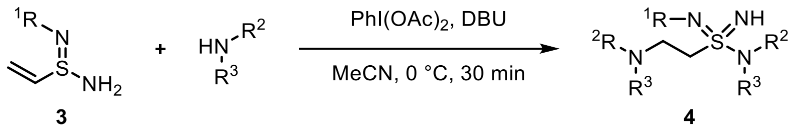

An oven-dried round-bottom flask containing primary sulfinamidine **3a-e** (1.0 equiv.) and $PhI(OAc)_2$ (1.5 equiv.) was sealed and flushed with N_2. Anhydrous MeCN (0.1 or 0.2 M sulfinamidine conc.) was added and the reaction was cooled to 0 ˚C. DBU (3.0 equiv.) was added to the solution, followed immediately by the addition of the amine (3.0 equiv.). The reaction mixture was stirred at 0 ˚C for 30 min then diluted with EtOAc (20 mL) and quenched with sat. aq. NaCl solution (30 mL). The aqueous phase was separated and extracted with EtOAc (3 × 20 mL). The combined organic layers were dried over anhydrous Na_2SO_4, filtered and concentrated *in vacuo*. The sulfondiimidamide was purified by flash column chromatography with the appropriate solvent system.

B-amino sulfondiimidamide 4a

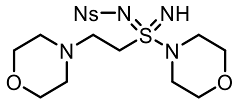

Following general procedure B, using sulfinamidine **3a** (275.3 mg, 1.00 mmol, 1.0 equiv.), PhI(OAc)$_2$ (483 mg, 1.50 mmol, 1.5 equiv.), MeCN (10.0 mL), DBU (0.45 mL, 3.00 mmol, 3.0 equiv.) and morpholine (0.26 mL, 3.00 mmol, 3.0 equiv.). Purification by flash column chromatography (SiO$_2$, EtOAc/MeOH, 12:1 to 10:1) afforded the desired product **4a** as a pale-yellow oil (380 mg, 85%).

***R*$_f$**= 0.59 (EtOAc/MeOH 5:1); **^{1}H NMR** (400 MHz, CDCl$_3$): δ 8.30 (d, 2H, *J* = 8.5 Hz, Ar*H*), 8.09 (d, 2H, *J* = 8.5 Hz, Ar*H*), 3.72 (t, 4H, *J* = 4.7 Hz, OC*H*$_2$), 3.62 (t, 4H, *J* = 4.7 Hz, OC*H*$_2$), 3.45 – 3.15 (m, 6H, SCH$_2$C*H*$_2$, NC*H*$_2$), 2.83 (dt, 1H, *J* = 13.6, 7.2 Hz, SC*H*$_2$), 2.72 (ddd, 1H, *J* = 13.3, 7.6, 5.8 Hz, SC*H*$_2$), 2.40 (q, 4H, *J* = 3.6 Hz, NC*H*$_2$); **^{13}C NMR** (101 MHz, CDCl$_3$): δ 149.5, 128.2, 127.8, 124.1, 66.7, 66.6, 53.5, 51.9, 49.3, 46.9; **IR** (ν_{max}, cm^{-1}) 1529, 1458, 1352, 1263, 1150, 1112, 912, 857, 805, 743; **HRMS** (ESI): *m/z* calcd for $C_{16}H_{26}N_5O_6S_2^+$: 448.1319 [M+H]$^+$; found: 448.1314.

B-amino sulfondiimidamide 4b

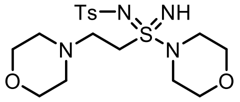

Following general procedure B, using sulfinamidine **3b** (244.3 mg, 1.00 mmol, 1.0 equiv.), PhI(OAc)$_2$ (483 mg, 1.50 mmol, 1.5 equiv.), MeCN (5.0 mL), DBU (0.45 mL, 3.00 mmol, 3.0 equiv.) and morpholine (0.26 mL, 3.00 mmol, 3.0 equiv.). Purification by flash column chromatography (SiO$_2$, EtOAc/MeOH, 12:1 to 10:1) afforded the desired product **4b** as a pale-yellow oil (248 mg, 60%).

***R*$_f$**= 0.55 (EtOAc/MeOH 5:1); **^{1}H NMR** (400 MHz, CDCl$_3$): δ 7.79 (d, 2H, *J* = 8.2 Hz, Ar*H*), 7.26 (d, 2H, *J* = 7.8 Hz, Ar*H*), 3.63 (dt, 8H, *J* = 4.8, 3.4 Hz, OC*H*$_2$), 3.38 – 3.22 (m, 4H, NC*H*$_2$), 3.22 – 3.03 (m, 2H, SCH$_2$C*H*$_2$), 2.90 – 2.64 (m, 2H, SC*H*$_2$CH$_2$), 2.45 – 2.30 (m, 7H, NC*H*$_2$, C*H*$_3$); **^{13}C NMR** (101 MHz, CDCl$_3$): δ 142.8, 141.1, 129.4, 126.6, 66.8, 66.5, 53.5, 51.8, 48.9, 46.8, 21.6; **IR** (ν_{max}, cm^{-1}) 1619, 1457, 1279, 1146, 1112, 1090, 1005, 914, 868, 816, 744, 666; **HRMS** (ESI): *m/z* calcd for $C_{17}H_{29}N_4O_4S_2^+$: 417.1625 [M+H]$^+$; found: 417.1637.

B-amino sulfondiimidamide 4c

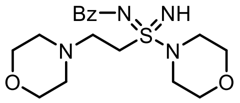

Following general procedure B, using sulfinamidine **3c** (194.3 mg, 1.00 mmol, 1.0 equiv.), $PhI(OAc)_2$ (483 mg, 1.50 mmol, 1.5 equiv.), MeCN (5 mL), DBU (0.45 mL, 3.00 mmol, 3.0 equiv.) and morpholine (0.26 mL, 3.00 mmol, 3.0 equiv.). Purification by flash column chromatography (SiO_2, EtOAc/MeOH, 20:1) afforded the desired product **4c** as a pale-yellow oil (256 mg, 70%).

***R*f**= 0.31 (EtOAc/MeOH 5:1); **^{1}H NMR** (500 MHz, $CDCl_3$): δ 8.05 (d, 2H, *J* = 6.9 Hz, Ar*H*), 7.46 (t, 1H, *J* = 7.4 Hz, Ar*H*), 7.37 (t, 2H, *J* = 7.7 Hz, Ar*H*), 3.75 – 3.64 (m, 8H, OC*H*2), 3.41 – 3.22 (m, 6H, NC*H*2, SCH_2CH_2), 3.06 – 2.91 (m, 2H, SC*H*2CH2), 2.52 (t, 4H, *J* = 4.7 Hz, NC*H*2); **^{13}C NMR** (126 MHz, $CDCl_3$): δ 174.6, 135.9, 131.9, 129.0, 128.1, 66.9, 66.8, 53.6, 52.1, 49.1, 46.6; **IR** (ν_{max}, cm^{-1}) 1605, 1572, 1450, 1315, 1292, 1257, 1114, 1068, 922, 829, 716; **HRMS** (ESI): *m/z* calcd for $C_{17}H_{27}N_4O_3S^+$: 367.1798 $[M+H]^+$; found: 367.1792.

B-amino sulfondiimidamide 4d

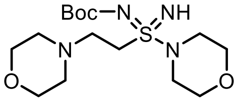

Following general procedure B, using sulfinamidine **3d** (951.3 mg, 5.00 mmol, 1.0 equiv.), $PhI(OAc)_2$ (2.42 g, 7.50 mmol, 1.5 equiv.), MeCN (25.0 mL), DBU (2.24 mL, 15.0 mmol, 3.0 equiv.) and morpholine (1.31 mL, 15.0 mmol, 3.0 equiv.). Purification by flash column chromatography (SiO_2, EtOAc/MeOH, 1:0 to 10:1) afforded the desired product **4d** as a pale-yellow oil (1.47 g, 81%).

***R*f**= 0.38 (EtOAc/MeOH 5:1); **^{1}H NMR** (400 MHz, $CDCl_3$): δ 3.69 (dq, 8H, *J* = 14.3, 4.5 Hz, OC*H*2), 3.29 (t, 4H *J* = 4.7 Hz, NC*H*2), 3.27 – 3.08 (m, 2H, SC*H*2), 2.90 (ddd, 2H, *J* = 8.9, 6.0, 2.3 Hz, SCH2C*H*2), 2.47 (dd, 4H, *J* = 5.6, 3.7 Hz, NC*H*2), 1.45 (s, 9H, C(C*H*3)3); **^{13}C NMR** (101 MHz, $CDCl_3$): δ 159.3, 80.0, 67.0, 66.9, 53.6, 51.9, 48.9, 46.7, 28.3; **IR** (ν_{max}, cm^{-1}) 1656, 1366, 1287, 1252, 1161, 1115, 1006, 927, 861, 734; **HRMS** (ESI): *m/z* calcd for $C_{15}H_{31}N_4O_4S^+$: 363.2061 $[M+H]^+$; found: 363.2064.

B-amino sulfondiimidamide 4e

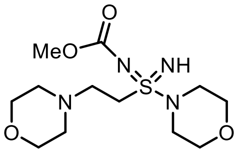

Following general procedure B, using sulfinamidine **3e** (370.5 mg, 2.50 mmol, 1.0 equiv.), $PhI(OAc)_2$ (1.21 g, 3.75 mmol, 1.5 equiv.), MeCN (12.5 mL), DBU (1.12 mL, 7.50 mmol, 3.0 equiv.) and morpholine (0.66 mL, 7.50 mmol, 3.0 equiv.) without aqueous workup. Purification by flash column chromatography (SiO_2, EtOAc/MeOH, 10:1 to 5:1) afforded the desired product **4e** as a pale-yellow oil (716 mg, 89%).

$\boldsymbol{R}_f$= 0.30 (EtOAc/MeOH 5:1); **^{1}H NMR** (400 MHz, $CDCl_3$): δ 3.70 – 3.56 (m, 11H, OCH_2, C(O)OCH_3), 3.24 (t, 4H J = 4.8 Hz, NCH_2), 3.21 – 3.06 (m, 2H, SCH_2), 2.85 (t, 2H, J = 7.4 Hz, SCH$_2$CH_2), 2.43 (t, 4H, J = 4.7 Hz, NCH_2); **^{13}C NMR** (101 MHz, $CDCl_3$): δ 160.1, 66.7, 53.4, 53.1, 52.8, 51.8, 48.1, 46.5; **IR** (ν_{max}, cm^{-1}) 1657, 1438, 1255, 1113, 1069, 1005, 925, 863, 789, 733; **HRMS** (ESI): m/z calcd for $C_{12}H_{25}N_4O_4S^+$: 321.1591 $[M+H]^+$; found: 321.1587.

B-amino sulfondiimidamide 4f

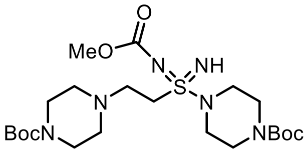

Following general procedure B, using sulfinamidine **3e** (148.2 mg, 1.00 mmol, 1.0 equiv.), $PhI(OAc)_2$ (483 mg, 1.50 mmol, 1.5 equiv.), MeCN (5.0 mL), DBU (0.45 mL, 3.00 mmol, 3.0 equiv.) and *tert*-Butyl piperazine-1-carboxylate (558 mg, 3.00 mmol, 3.0 equiv.) without aqueous workup. Purification by flash column chromatography (SiO_2, EtOAc/MeOH, 1:0 to 20:1) afforded the desired product **4f** as a pale-yellow oil (428 mg, 83%).

$\boldsymbol{R}_f$= 0.67 (EtOAc/MeOH 5:1); **^{1}H NMR** (400 MHz, $CDCl_3$): δ 3.63 (s, 3H, C(O)OCH_3), 3.51 – 3.41 (m, 4H, NCH_2), 3.37 (t, 4H, J = 5.1 Hz, NCH_2), 3.25 (t, 4H, J = 5.1 Hz, NCH_2), 3.15 (ddd, 2H, J = 20.5, 14.4, 7.1 Hz, SCH_2), 2.89 (t, 2H J = 7.3 Hz, SCH$_2$CH_2), 2.39 (t, 4H, J = 5.1 Hz, NCH_2), 1.42 (s, 9H, C(CH_3)$_3$), 1.41 (s, 9H, C(CH_3)$_3$); **^{13}C NMR** (101 MHz, $CDCl_3$): δ 160.3, 154.7, 154.3, 80.5, 79.9, 52.94, 52.88, 51.6, 49.1, 46.2, 43.9, 43.3, 28.5, 28.4; **IR** (ν_{max}, cm^{-1}) 1694, 1421, 1366,

1258, 1169, 1126, 1000, 911, 863, 769, 735; **HRMS** (ESI): *m/z* calcd for $C_{22}H_{43}N_6O_6S^+$: 519.2959 $[M+H]^+$; found: 519.2965.

B-amino sulfondiimidamide 4g

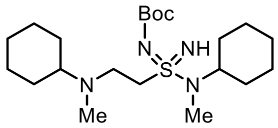

Following general procedure B, using sulfinamidine **3d** (95.1 mg, 0.50 mmol, 1.0 equiv.), $PhI(OAc)_2$ (242 mg, 0.75 mmol, 1.5 equiv.), MeCN (2.5 mL), DBU (0.22 mL, 1.50 mmol, 3.0 equiv.) and *N*-cyclohexylmethylamine (0.19 mL, 1.50 mmol, 3.0 equiv.). Purification by flash column chromatography (SiO_2, EtOAc) afforded the desired product **4g** as a pale-yellow oil (128 mg, 62%).

$\boldsymbol{R_f}$= 0.46 (EtOAc/MeOH 5:1); **^{1}H NMR** (400 MHz, $CDCl_3$): δ 3.18 (ddt, 1H, *J* = 11.7, 7.3, 3.7 Hz, C*H*), 3.19 – 3.08 (m, 1H, C*H*), 3.01 – 2.88 (m, 4H, alkyl*H*), 2.78 (s, 3H, NCH_3), 2.22 (s, 3H, NCH_3), 1.81 – 1.66 (m, 8H, alkyl*H*), 1.64 – 1.51 (m, 2H, alkyl*H*), 1.45 –1.26 (m, 13H, C(CH_3)$_3$), alkyl*H*), 1.17 (tt, 4H, *J* = 8.5, 3.3 Hz, alkyl*H*), 1.03 (ddt, 2H, *J* = 12.7, 9.2, 3.8 Hz, alkyl*H*); **^{13}C NMR** (101 MHz, $CDCl_3$): δ 159.5, 79.3, 63.3, 56.7, 52.3, 48.0, 38.0, 31.3, 31.0, 29.1, 29.0, 28.9, 28.4, 28.3, 26.23, 25.93, 25.90, 25.86, 25.4 (Note: for the cyclohexane rings, 6 peaks were found instead of 4 due to the loss of symmetry caused by the stereogenic sulfur atom); **IR** (ν_{max}, cm^{-1}) 1649, 1451, 1365, 1284, 1251, 1163, 1044, 997, 942, 886, 858, 792; **HRMS** (ESI): *m/z* calcd for $C_{21}H_{43}N_4O_2S^+$: 415.3101 $[M+H]^+$; found: 415.3087.

B-amino sulfondiimidamide 4h

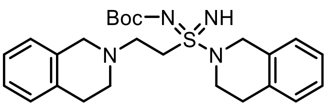

Following general procedure B, using sulfinamidine **3d** (95.1 mg, 0.50 mmol, 1.0 equiv.), $PhI(OAc)_2$ (242 mg, 0.75 mmol, 1.5 equiv.), MeCN (2.5 mL), DBU (0.22 mL, 1.50 mmol, 3.0 equiv.) and tetrahydroisoquinoline (0.19 mL, 1.50 mmol, 3.0 equiv.). Purification by flash column chromatography (SiO_2, EtOAc) afforded the desired product **4h** as a pale-yellow oil (197 mg, 87%).

***R*f**= 0.79 (EtOAc/MeOH 5:1); **^{1}H NMR** (400 MHz, $CDCl_3$): δ 7.16 (q, 2H, *J* = 4.9 Hz, Ar*H*), 7.10 (td, 3H *J* = 6.8, 4.0 Hz, Ar*H*), 7.05 (q, 2H, *J* = 4.2 Hz, Ar*H*), 6.95 (d, 1H, *J* = 6.4 Hz, Ar*H*), 4.55 (d, 2H, *J* = 3.7 Hz, NCH_2), 3.72 (dt, 1H, *J* = 12.0, 6.0 Hz, alkyl*H*), 3.63 (d, 3H, *J* = 5.4 Hz, alkyl*H*), 3.46 – 3.35 (m, 1H, alkyl*H*), 3.34 – 3.23 (m, 1H, alkyl*H*), 3.10 (ddd, 2H, *J* = 9.3, 5.9, 3.2 Hz, alkyl*H*) 3.02 – 2.86 (m, 2H, alkyl*H*), 2.83 (t, 2H, *J* = 5.9 Hz, alkyl*H*), 2.74 (t, 2H, *J* = 6.1 Hz, alkyl*H*), 1.45 (s, 9H, $C(CH_3)_3$); **^{13}C NMR** (101 MHz, $CDCl_3$): δ 159.4, 134.1, 133.9, 133.6, 132.8, 129.1, 128.8, 127.0, 126.6, 126.5, 126.4, 125.8, 79.9, 55.9, 51.5, 51.1, 50.6, 48.2, 44.1, 29.5, 29.1, 28.4; **IR** ($ν_{max}$, cm^{-1}) 1651, 1366, 1283, 1251, 1161, 1095, 1015, 935, 884, 858, 746; **HRMS** (ESI): *m/z* calcd for $C_{25}H_{35}N_4O_2S^+$: 455.2475 $[M+H]^+$; found: 455.2486.

2.1.4 *N*-functionalisation of β-amino sulfondiimidamides

Β-amino sulfondiimidamide 5a

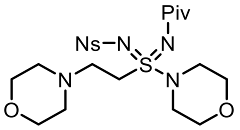

An oven-dried round-bottom flask containing sulfondiimidamide **4a** (349.1 mg, 0.78 mmol, 1.0 equiv.) was sealed and flushed with N_2 before anhydrous THF (8.0 mL) was added. The reaction was cooled to 0 ˚C, NaH (31.2 mg, 0.78 mmol, 60% dispersion in mineral oil, 1.0 equiv.) was added and the reaction was stirred for 20 min at 0 ˚C. Trimethylacetyl chloride (0.12 mL, 0.94 mmol, 1.2 equiv.) was then added and the reaction was warmed to rt and stirred for 4 h. The reaction mixture was then diluted with sat. aq. NaCl solution (15 mL) and extracted with EtOAc (3 × 15 mL). The combined organic layers were dried over Na_2SO_4, filtered and concentrated *in vacuo*. Purification by flash column chromatography (SiO_2, EtOAc) afforded the desired product **5a** as a colourless oil (224 mg, 54%).

***R*f**= 0.60 (EtOAc/MeOH 10:1); **^{1}H NMR** (400 MHz, $CDCl_3$): δ 8.30 (d, 2H, *J* = 9.0, Ar*H*), 8.10 (d, 2H, *J* = 8.8, Ar*H*), 3.94 – 3.83 (m, 2H, SCH_2), 3.81 (td, 4H, *J* = 4.2, 2.1, OCH_2), 3.67 (t, 4H, *J* = 4.7, OCH_2), 3.55 – 3.36 (m, 4H, NCH_2), 2.74 – 2.63 (m, 2H, SCH_2CH_2), 2.52 – 2.37 (m, 4H, NCH_2), 0.98 (s, 9H, $C(CH_3)_3$); **^{13}C NMR** (101 MHz, $CDCl_3$): δ 186.3, 149.9, 148.6, 128.3, 123.9, 66.8, 66.4, 53.7, 51.4, 49.6, 46.3, 42.1, 27.5; **IR** ($ν_{max}$, cm^{-1}) 1645, 1530, 1456, 1351, 1303, 1160, 1113, 1070, 1032, 1009, 1032, 1009, 939, 855, 746, 685, 620; **HRMS** (ESI): *m/z* calcd for $C_{21}H_{34}N_5O_7S_2^+$: 532.1894 $[M+H]^+$; found: 532.1895.

B-amino sulfondiimidamide 5b

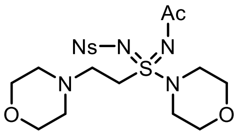

An oven-dried round-bottom flask containing sulfondiimidamide **4a** (250.6 mg, 0.56 mmol, 1.0 equiv.) was sealed and flushed with N_2 before anhydrous THF (5.6 mL) was added. The reaction was cooled to 0 ˚C, NaH (22.4 mg, 0.56 mmol, 60% dispersion in mineral oil, 1.0 equiv.) was added and the reaction was stirred for 20 min at 0 ˚C. Acetic anhydride (63 µL, 0.67 mmol, 1.2 equiv.) was then added and the reaction was warmed to rt and stirred for 6 h. The reaction mixture was then diluted with sat. aq. NaCl solution (10 mL) and extracted with EtOAc (3 × 10 mL). The combined organic layers were dried over Na_2SO_4, filtered and concentrated *in vacuo*. Purification by flash column chromatography (SiO_2, EtOAc) afforded the desired product **5b** as a colourless oil (90.2 mg, 33%).

$\boldsymbol{R}_{\mathbf{f}}$= 0.26 (EtOAc); **^{1}H NMR** (400 MHz, $CDCl_3$): δ 8.31 (d, 2H, *J* = 8.5 Hz, Ar*H*), 8.11 (d, 2H, *J* = 8.5 Hz, Ar*H*), 3.95 – 3.74 (m, 6H, OC*H*$_2$, SC*H*$_2$), 3.66 (t, 4H, *J* = 4.7 Hz, OC*H*$_2$), 3.46 (dddd, 4H, *J* = 21.8, 16.5, 10.7, 4.7 Hz, NC*H*$_2$), 2.72 (q, 2H, *J* = 8.7 Hz, SCH$_2$C*H*$_2$), 2.53 – 2.35 (m, 4H, NC*H*$_2$), 1.82 (s, 3H, C(O)C*H*$_3$); **^{13}C NMR** (101 MHz, $CDCl_3$): δ 177.9, 149.9, 148.2, 128.6, 123.9, 66.8, 66.4, 53.7, 51.4, 49.7, 46.3, 26.7; **IR** (ν_{max}, cm^{-1}) 1653, 1526, 1355, 1302, 1237, 1161, 1110, 1065, 919, 853, 776, 683, 620; **HRMS** (ESI): *m/z* calcd for $C_{18}H_{28}N_5O_7S_2^+$: 490.1425 [M+H]$^+$; found: 490.1410.

B-amino sulfondiimidamide 5c

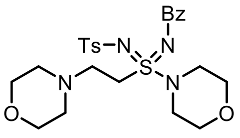

An oven-dried round-bottom flask containing sulfondiimidamide **4b** (120.8 mg, 0.29 mmol, 1.0 equiv.) was sealed and flushed with N_2 before anhydrous THF (2.9 mL) was added. The reaction was cooled to 0 ˚C, NaH (11.6 mg, 0.29 mmol, 60% dispersion in mineral oil, 1.0 equiv.) and 15-crown-5 (57 µL, 0.29 mmol, 1.0 equiv.) were added and the reaction was stirred for 20 min at 0 ˚C. Benzoyl chloride (41 µL, 0.35 mmol, 1.2 equiv.) was then added and the reaction was warmed to rt and stirred for 2 h. The reaction mixture was then diluted with

sat. aq. NaCl solution (10 mL) and extracted with EtOAc (3 × 10 mL). The combined organic layers were dried over Na_2SO_4, filtered and concentrated *in vacuo*. Purification by flash column chromatography (SiO_2, PE/EtOAc/MeOH, 1:5:0 to 0:1:0 to 0:12:1) afforded the desired product **5c** as a colourless oil (64.5 mg, 43%).

***R*f**= 0.59 (EtOAc/MeOH 10:1); **^{1}H NMR** (400 MHz, $CDCl_3$): δ 7.68 (td, 4H, *J* = 6.0, 2.9 Hz, Ar*H*), 7.46 (td, 1H, *J* = 7.3, 1.5 Hz, Ar*H*), 7.30 (t, 2H, *J* = 7.7 Hz, Ar*H*), 6.91 (d, 2H, *J* = 8.0 Hz, Ar*H*), 4.09 – 3.90 (m, 2H, SC*H*2), 3.88 – 3.75 (m, 4H, OC*H*2), 3.67 – 3.45 (m, 8H, OC*H*2, NC*H*2), 2.84 – 2.67 (m, 2H, SCH2C*H*2), 2.41 (dq, 4H, *J* = 11.8, 5.7 Hz, NC*H*2), 2.13 (s, 3H, C*H*3); **^{13}C NMR** (101 MHz, $CDCl_3$): δ 171.3, 142.8, 139.5, 134.7, 132.5, 129.5, 129.1, 127.9, 127.1, 66.6, 66.5, 53.6, 51.5, 49.5, 46.3, 21.4; **IR** (ν_{max}, cm^{-1}) 1637, 1451, 1313, 1285, 1145, 1115, 1070, 943, 916, 732, 712, 649; **HRMS** (ESI): *m/z* calcd for $C_{24}H_{33}N_4O_5S_2^+$: 521.1887 $[M+H]^+$; found: 521.1891.

B-amino sulfondiimidamide 5d

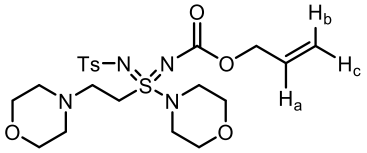

An oven-dried round-bottom flask containing sulfondiimidamide **4b** (120.8 mg, 0.29 mmol, 1.0 equiv.) was sealed and flushed with N_2 before anhydrous THF (2.9 mL) was added. The reaction was cooled to 0 ˚C, NaH (11.6 mg, 0.29 mmol, 60% dispersion in mineral oil, 1.0 equiv.) and 15-crown-5 (57 µL, 0.29 mmol, 1.0 equiv.) were added and the reaction was stirred for 20 min at 0 ˚C. Allyl chloroformate (37 µL, 0.35 mmol, 1.2 equiv.) was then added and the reaction was warmed to rt and stirred for 2 h. The reaction mixture was then diluted with sat. aq. NaCl solution (10 mL) and extracted with EtOAc (3 × 10 mL). The combined organic layers were dried over Na_2SO_4, filtered and concentrated *in vacuo*. Purification by flash column chromatography (SiO_2, EtOAc/MeOH, 1:0 to 15:1) afforded the desired product **5d** as a colourless oil (44.8 mg, 31%).

***R*f**= 0.45 (EtOAc/MeOH 10:1); **^{1}H NMR** (400 MHz, $CDCl_3$): δ 7.81 (d, 2H, *J* = 8.3 Hz, Ar*H*), 7.23 (d, 2H, *J* = 8.0 Hz, Ar*H*), 5.76 (ddt, 1H, *J* = 17.2, 10.3, 5.8 Hz, C*Ha*), 5.23 (dd, 1H, *J* = 17.2, 1.5 Hz, C*Hb*), 5.17 (dd, 1H, *J* = 10.4, 1.3 Hz, C*Hc*), 4.15 (dd, 2H, *J* = 5.8, 1.4 Hz, C(O)OC*H*2), 3.81 (td, 2H, *J* = 6.6, 4.4 Hz, SC*H*2), 3.73 (t, 4H, *J* = 4.7 Hz, OC*H*2), 3.64 (t, 4H, *J* = 4.6 Hz, OC*H*2), 3.53 – 3.35 (m, 4H, NC*H*2), 2.80 – 2.63 (m, 2H, SCH2C*H*2), 2.46 – 2.40 (m, 4H, NC*H*2), 2.37 (s, 3H, C*H*3);

^{13}C NMR (101 MHz, $CDCl_3$): δ 156.2, 143.0, 139.8, 132.1, 129.3, 127.3, 118.4, 66.9, 66.7, 66.4, 53.7, 51.3, 49.8, 46.2, 21.6; **IR** (ν_{max}, cm^{-1}) 1678, 1456, 1361, 1253, 1156, 1115, 1070, 1034, 1005, 914, 732; **HRMS** (ESI): *m/z* calcd for $C_{21}H_{33}N_4O_6S_2^+$: 501.1836 $[M+H]^+$; found: 501.1828.

Β-amino sulfondiimidamide 5e

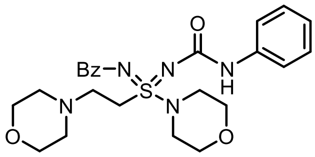

An oven-dried round-bottom flask containing sulfondiimidamide **4c** (73.3 mg, 0.20 mmol, 1.0 equiv.) was sealed and flushed with N_2 before anhydrous MeCN (2.5 mL) was added. The reaction was cooled to 0 ˚C, DBU (45 µL, 0.30 mmol, 1.5 equiv.) was added and the reaction was stirred for 20 min at 0 ˚C. Phenyl isocyanate (32 µL, 0.30 mmol, 1.5 equiv.) was then added and the reaction was warmed to rt and stirred for 30 min. The reaction mixture was then diluted with sat. aq. NaCl solution (10 mL) and extracted with EtOAc (3 × 10 mL). The combined organic layers were dried over Na_2SO_4, filtered and concentrated *in vacuo*. Purification by flash column chromatography (SiO_2, EtOAc) afforded the desired product **5e** as a colourless oil (65.2 mg, 67%).

***R*$_f$**= 0.44 (EtOAc/MeOH 5:1); **^{1}H NMR** (400 MHz, $CDCl_3$): δ 8.12 (d, 2H, *J* = 7.1 Hz, Ar*H*), 7.50 (t, 1H, *J* = 7.5 Hz, Ar*H*), 7.41 (t, 4H, *J* = 7.7 Hz, Ar*H*), 7.26 (t, 2H, *J* = 15.9 Hz, Ar*H*), 7.01 (t, 1H, *J* = 7.4 Hz, Ar*H*), 4.22 – 4.11 (m, 1H, SC*H*), 3.97 (dt, 1H, *J* = 13.7, 6.8 Hz, SC*H*), 3.80 (t, 4H, *J* = 4.8 Hz, OC*H*$_2$), 3.62 (t, 4H, *J* = 4.6 Hz, OC*H*$_2$), 3.50 (dtd, 4H, *J* = 12.4, 7.5, 4.9 Hz, NC*H*$_2$), 2.89 (t, 2H, *J* = 6.9 Hz, SCH_2C*H*$_2$), 2.49 (t, 4H, *J* = 4.7 Hz, NC*H*$_2$); **^{13}C NMR** (101 MHz, $CDCl_3$): δ 173.0, 156.0, 138.9, 135.6, 132.4, 129.6, 129.0, 128.2, 123.2, 118.9, 66.8, 66.7, 53.7, 51.9, 48.0, 46.6; **IR** (ν_{max}, cm^{-1}) 1633, 1597, 1532, 1439, 1313, 1289, 1256, 1225, 1139, 1113, 934, 847, 758, 714; **HRMS** (ESI): *m/z* calcd for $C_{24}H_{32}N_5O_4S^+$: 486.2170 $[M+H]^+$; found: 486.2159.

B-amino sulfondiimidamide 5f

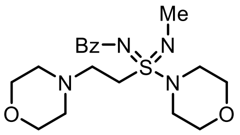

An oven-dried round-bottom flask containing sulfondiimidamide **4c** (183.2 mg, 0.50 mmol, 1.0 equiv.) was sealed and flushed with N_2 before anhydrous DMF (2.5 mL) was added. The reaction was cooled to 0 ˚C, NaH (24.0 mg, 0.60 mmol, 60% dispersion in mineral oil, 1.2 equiv.) was added and the reaction was stirred for 20 min at 0 ˚C. Methyl iodide (34 µL, 0.55 mmol, 1.1 equiv.) was then added and the reaction was warmed to rt and stirred for 4 h. The reaction mixture was then diluted with sat. aq. NaCl solution (15 mL) and extracted with EtOAc (3 × 15 mL). The combined organic layers were dried over Na_2SO_4, filtered and concentrated *in vacuo*. Purification by flash column chromatography (SiO_2, EtOAc/MeOH, 1:0 to 10:1) afforded the desired product **5f** as a colourless oil (131 mg, 69%).

$\boldsymbol{R}_f$= 0.33 (EtOAc/MeOH 10:1); **^{1}H NMR** (400 MHz, $CDCl_3$): δ 8.14 – 8.06 (m, 2H, Ar*H*), 7.51 – 7.43 (m, 1H, Ar*H*), 7.42 – 7.33 (m, 2H, Ar*H*), 3.77 (ddd, 4H, *J* = 5.7, 4.6, 3.6 Hz, OC*H*$_2$), 3.70 – 3.53 (m, 6H, OC*H*$_2$, SC*H*$_2$), 3.36 (qdd, 4H, *J* = 12.1, 5.6, 3.6 Hz, NC*H*$_2$), 2.91 (m, 2H, SCH$_2$C*H*$_2$), 2.78 (s, 3H, C*H*$_3$), 2.47 (q, 4H, *J* = 4.0 Hz, NC*H*$_2$); **^{13}C NMR** (101 MHz, $CDCl_3$): δ 172.8, 136.4, 131.8, 129.3, 128.0, 67.0, 66.9, 53.7, 52.3, 48.9, 46.0, 30.3; **IR** (ν_{max}, cm^{-1}) 1624, 1575, 1449, 1313, 1290, 1255, 1225, 1113, 1068, 1025, 1006, 930, 865, 714; **HRMS** (ESI): *m/z* calcd for $C_{18}H_{29}N_4O_3S^+$: 381.1955 [M+H]$^+$; found: 381.1949.

B-amino sulfondiimidamide 5g

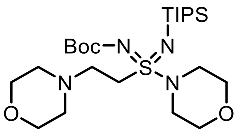

An oven-dried round-bottom flask containing sulfondiimidamide **4d** (152.2 mg, 0.42 mmol, 1.0 equiv.) was sealed and flushed with N_2 before anhydrous THF (4.2 mL) was added. The reaction was cooled to 0 ˚C, NaH (16.8 mg, 0.42 mmol, 60% dispersion in mineral oil, 1.0 equiv.) was added and the reaction was stirred for 20 min at 0 ˚C. Triisopropylsilyl chloride (0.11 mL, 0.50 mmol, 1.2 equiv.) was then added and the reaction was warmed to rt and stirred for 18 h. The reaction mixture was then diluted with sat. aq. NaCl solution (10 mL) and

extracted with EtOAc (3 × 10 mL). The combined organic layers were dried over Na_2SO_4, filtered and concentrated *in vacuo*. Purification by flash column chromatography (SiO_2, PE/EtOAc/MeOH, 1:5:0 to 0:1:0 to 0:10:1) afforded the desired product **5g** as a colourless oil (74.1 mg, 34%).

***R*f**= 0.55 (EtOAc/MeOH 10:1); **^{1}H NMR** (400 MHz, $CDCl_3$): δ 3.69 (dt, 8H, *J* = 13.5, 4.7 Hz, OC*H*$_2$), 3.47 – 3.18 (m, 6H, SC*H*$_2$, NC*H*$_2$), 2.74 (ddd, 2H, *J* = 8.7, 6.2, 2.2 Hz, SCH_2CH_2), 2.43 (ddd, 4H, *J* = 5.7, 3.5, 1.5, NC*H*$_2$), 1.42 (s, 9H, C(C*H*$_3$)$_3$), 1.06 (s, 21H, SiC*H*, C*H*$_3$); **^{13}C NMR** (101 MHz, $CDCl_3$): δ 158.2, 78.9, 67.0, 66.9, 53.8, 52.5, 51.6, 46.3, 28.4, 18.5, 13.4; **IR** (ν_{max}, cm^{-1}) 1656, 1454, 1391, 1277, 1253, 1172, 1117, 937, 887, 866, 735, 679; **HRMS** (ESI): *m/z* calcd for $C_{24}H_{51}N_4O_4SSi^+$: 519.3395 [M+H]$^+$; found: 519.3387.

B-amino sulfondiimidamide 5h

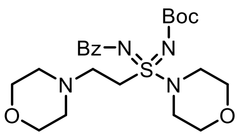

An oven-dried round-bottom flask containing sulfondiimidamide **4c** (91.6 mg, 0.25 mmol, 1.0 equiv.) was sealed and flushed with N_2 before anhydrous THF (2.5 mL) was added. The reaction was cooled to 0 ˚C, NaH (10.0 mg, 0.25 mmol, 60% dispersion in mineral oil, 1.0 equiv.) and 15-crown-5 (49 µL, 0.25 mmol, 1.0 equiv.) were added and the reaction was stirred for 20 min at 0 ˚C. Di-*tert*-butyl dicarbonate (54.6 mg, 0.25 mmol, 1.0 equiv.) was then added and the reaction was warmed to rt and stirred for 6 h. The reaction mixture was then diluted with sat. aq. NaCl solution (10 mL) and extracted with EtOAc (3 × 10 mL). The combined organic layers were dried over Na_2SO_4, filtered and concentrated *in vacuo*. Purification by flash column chromatography (SiO_2, EtOAc/MeOH, 1:0 to 5:1) afforded the desired product **5h** as a colourless oil (70.2 mg, 60%).

***R*f**= 0.51 (EtOAc/MeOH 10:1); **^{1}H NMR** (400 MHz, $CDCl_3$): δ 8.10 (dd, 2H, *J* = 8.2, 1.5 Hz, Ar*H*), 7.53 – 7.44 (m, 1H, Ar*H*), 7.38 (t, 2H, *J* = 7.6 Hz, Ar*H*), 3.97 (td, 2H, *J* = 14.2, 7.2 Hz, SC*H*$_2$), 3.79 (t, 4H, *J* = 4.7 Hz, OC*H*$_2$), 3.60 (t, 4H, *J* = 4.6 Hz, OC*H*$_2$), 3.52 – 3.43 (m, 4H, NC*H*$_2$), 2.82 (t, 2H, *J* = 6.9 Hz, SCH_2CH_2), 2.45 (t, 4H, *J* = 4.7 Hz, NC*H*$_2$), 1.44 (s, 9H, C(C*H*$_3$)$_3$); **^{13}C NMR** (101 MHz, $CDCl_3$): δ 172.4, 156.6, 135.7, 132.3, 129.6, 128.4, 80.8, 66.8, 66.7, 53.6, 51.7, 48.3, 46.6, 28.1;

IR (ν_{max}, cm^{-1}) 1670, 1632, 1451, 131, 1292, 1272, 1251, 1139, 1115, 1069, 936, 865, 714; **HRMS** (ESI): *m/z* calcd for $C_{22}H_{35}N_4O_5S^+$: 467.2323 [M+H]$^+$; found: 467.2321.

B-amino sulfondiimidamide 5i

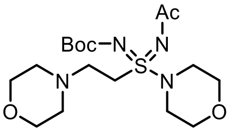

An oven-dried round-bottom flask containing sulfondiimidamide **4d** (460.4 mg, 1.27 mmol, 1.0 equiv.) was sealed and flushed with N_2 before anhydrous MeCN (12.7 mL) was added. The reaction was cooled to 0 ˚C, DBU (0.57 mL, 3.82 mmol, 3.0 equiv.) was added and the reaction was stirred for 20 min at 0 ˚C. Acetyl chloride (0.23 mL, 3.17 mmol, 2.5 equiv.) was then added and the reaction was warmed to rt and stirred for 3 h. The reaction mixture was then diluted with sat. aq. NaCl solution (20 mL) and extracted with EtOAc (3 × 20 mL). The combined organic layers were dried over Na_2SO_4, filtered and concentrated *in vacuo*. Purification by flash column chromatography (SiO_2, EtOAc/MeOH, 20:1 to 10:1) afforded the desired product **5i** as a colourless oil (255 mg, 50%).

***R*$_f$**= 0.41 (EtOAc/MeOH 10:1); **^{1}H NMR** (400 MHz, $CDCl_3$): δ 3.88 – 3.70 (m, 6H, SC*H*$_2$, OC*H*$_2$), 3.70 – 3.61 (m, 4H, OC*H*$_2$), 3.40 – 3.34 (m, 4H, NC*H*$_2$), 2.76 (t, 2H, *J* = 7.1 Hz, SCH$_2$C*H*$_2$), 2.46 (t, 4H, *J* = 4.7 Hz, NC*H*$_2$), 2.12 (s, 3H, C*H*$_3$), 1.45 (s, 9H, C(C*H*$_3$)$_3$); **^{13}C NMR** (101 MHz, $CDCl_3$): δ 178.6, 156.8, 80.8, 66.8, 66.6, 53.7, 51.6, 48.0, 46.4, 28.2, 27.1; **IR** (ν_{max}, cm^{-1}) 1648, 1365, 1287, 1245, 1143, 1114, 1068, 1038, 923, 865, 731; **HRMS** (ESI): *m/z* calcd for $C_{17}H_{33}N_4O_5S^+$: 405.2166 [M+H]$^+$; found: 405.2155.

B-amino sulfondiimidamide 5j

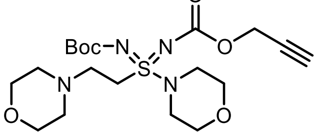

An oven-dried round-bottom flask containing sulfondiimidamide **4d** (181.2 mg, 0.50 mmol, 1.0 equiv.) was sealed and flushed with N_2 before anhydrous THF (5.0 mL) was added. The reaction was cooled to 0 ˚C, NaH (20.0 mg, 0.50 mmol, 60% dispersion in mineral oil, 1.0 equiv.) was added and the reaction was stirred for 20 min at 0 ˚C. Propargyl chloroformate

(59 µL, 0.60 mmol, 1.2 equiv.) was then added and the reaction was warmed to rt and stirred for 18 h. The reaction mixture was then diluted with sat. aq. NaCl solution (15 mL) and extracted with EtOAc (3 × 15 mL). The combined organic layers were dried over Na_2SO_4, filtered and concentrated *in vacuo*. Purification by flash column chromatography (SiO_2, EtOAc/MeOH, 1:0 to 20:1) afforded the desired product **5j** as a white solid (133 mg, 60%).

***R*$_f$**= 0.49 (EtOAc/MeOH 5:1); **^{1}H NMR** (400 MHz, $CDCl_3$): δ 4.66 – 4.60 (m, 2H, C(O)OCH_2), 3.73 (q, 5H, *J* = 5.7 Hz, SC*H*, OCH_2), 3.64 (t, 5H, *J* = 4.6 Hz, SC*H*, OCH_2), 3.39 (t, 4H, *J* = 4.2 Hz, NCH_2), 2.84 – 2.72 (m, 2H, SCH_2CH_2), 2.47 – 2.39 (m, 5H, NCH_2, alkynylC*H*), 1.43 (s, 9H, C(CH_3)$_3$); **^{13}C NMR** (101 MHz, $CDCl_3$): δ 156.3, 156.0, 81.1, 78.2, 74.8, 66.8, 66.5, 53.59, 53.57, 51.5, 48.9, 46.3, 28.1; **IR** (ν_{max}, cm^{-1}) 1680, 1456, 1368, 1289, 1238, 1143, 1114, 1068, 1006, 982, 862, 784, 635; **HRMS** (ESI): *m/z* calcd for $C_{19}H_{33}N_4O_6S^+$: 445.2115 [M+H]$^+$; found: 445.2119.

B-amino sulfondiimidamide 5k

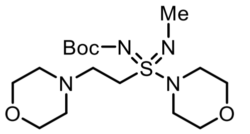

An oven-dried round-bottom flask containing sulfondiimidamide **4d** (543.7 mg, 1.50 mmol, 1.0 equiv.) was sealed and flushed with N_2 before anhydrous THF (7.5 mL) was added. The reaction was cooled to 0 ˚C, NaH (72 mg, 1.80 mmol, 60% dispersion in mineral oil, 1.2 equiv.) was added and the reaction was stirred for 20 min at 0 ˚C. Methyl iodide (0.10 mL, 1.65 mmol, 1.1 equiv.) was then added and the reaction was warmed to rt and stirred for 4 h. The reaction mixture was then diluted with sat. aq. NaCl solution (20 mL) and extracted with EtOAc (3 × 20 mL). The combined organic layers were dried over Na_2SO_4, filtered and concentrated *in vacuo*. Purification by flash column chromatography (SiO_2, EtOAc/MeOH, 20:1) afforded the desired product **5k** as a colourless oil (266 mg, 47%).

***R*$_f$**= 0.19 (EtOAc/MeOH 5:1); **^{1}H NMR** (500 MHz, $CDCl_3$): δ 3.73 (ddd, 4H, *J* = 10.5, 6.1, 3.1 Hz, OCH_2), 3.69 (t, 4H, *J* = 4.6 Hz, OCH_2), 3.57 – 3.42 (m, 2H, SCH_2), 3.39 – 3.23 (m, 4H, NCH_2), 2.87 – 2.77 (m, 2H, SCH_2CH_2), 2.76 (s, 3H, C(O)CH_3), 2.51 – 2.45 (m, 4H, NCH_2), 1.47 (s, 9H, C(CH_3)$_3$); **^{13}C NMR** (126 MHz, $CDCl_3$): δ 158.4, 79.7, 70.0, 66.9, 53.8, 52.3, 48.7, 46.2, 30.1, 28.3; **IR** (ν_{max}, cm^{-1}) 1658, 1286, 1253, 1160, 1116, 1067, 1006, 928, 903, 869; **HRMS** (ESI): *m/z* calcd for $C_{16}H_{33}N_4O_4S^+$: 377.2217 [M+H]$^+$; found: 377.2209.

B-amino sulfondiimidamide 5l

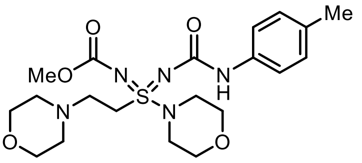

An oven-dried round-bottom flask containing sulfondiimidamide **4e** (320.4 mg, 1.00 mmol, 1.0 equiv.) was sealed and flushed with N_2 before anhydrous MeCN (10.0 mL) was added. DBU (0.22 mL, 1.50 mmol, 1.5 equiv.) was then added followed by *p*-tolyl isocyanate (0.19 mL, 1.50 mmol, 1.5 equiv.). The reaction mixture was stirred at rt for 30 min. The reaction mixture was then diluted with sat. aq. NaCl solution (20 mL) and extracted with EtOAc (3 × 20 mL). The combined organic layers were dried over Na_2SO_4, filtered and concentrated *in vacuo*. Purification by flash column chromatography (SiO_2, EtOAc/MeOH, 1:0 to 20:1) afforded the desired product **5l** as a colourless oil (281 mg, 62%).

***R*f**= 0.42 (EtOAc/MeOH 5:1); **^{1}H NMR** (400 MHz, $CDCl_3$): δ 7.28 (d, 2H, *J* = 8.4 Hz, Ar*H*), 7.08 (d, 2H, *J* = 8.5 Hz, Ar*H*), 4.06 – 3.92 (m, 1H, SC*H*2), 3.77 (t, 5H, *J* = 4.6 Hz, SC*H*2, OC*H*2), 3.69 (s, 3H, C(O)OC*H*3), 3.66 (t, 4H, *J* = 4.7, OC*H*2), 3.51 – 3.36 (m, 4H, NC*H*2), 2.85 (t, 2H, *J* = 6.9 Hz, SCH2C*H*2), 2.49 (t, 4H, *J* = 4.7 Hz, NC*H*2), 2.28 (s, 3H, C*H*3); **^{13}C NMR** (101 MHz, $CDCl_3$): δ 158.6, 155.7, 136.3, 132.9, 129.5, 118.9, 66.9, 66.7, 53.7, 53.4, 51.8, 48.3, 46.5, 20.9; **IR** (ν_{max}, cm^{-1}) 1652, 1593, 1522, 1437, 1253, 1224, 1147, 1114, 934, 869, 819, 786, 735, 702; **HRMS** (ESI): *m/z* calcd for $C_{20}H_{32}N_5O_5S^+$: 454.2119 [M+H]$^+$; found: 454.2110.

B-amino sulfondiimidamide 5m

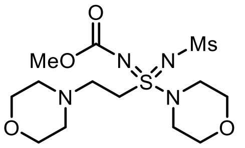

An oven-dried round-bottom flask containing sulfondiimidamide **4e** (320.4 mg, 1.00 mmol, 1.0 equiv.) was sealed and flushed with N_2 before CH_2Cl_2 (5.0 mL) was added. Et_3N (0.21 mL, 1.50 mmol, 1.5 equiv.) was then added followed by methanesulfonyl chloride (0.14 mL, 1.20 mmol, 1.2 equiv.) and DMAP (24.4 mg, 0.20 mmol, 0.2 equiv.). The reaction mixture was stirred at rt for 1 h. The reaction mixture was diluted with sat. aq. NaCl solution (20 mL) and extracted with CH_2Cl_2 (3 × 20 mL). The combined organic layers were dried over Na_2SO_4,

filtered and concentrated *in vacuo*. Purification by flash column chromatography (SiO_2, EtOAc) afforded the desired product **5m** as a colourless oil (284 mg, 71%).

***R*f**= 0.50 (EtOAc/MeOH 5:1); **^{1}H NMR** (500 MHz, $CDCl_3$): δ 3.82 – 3.73 (m, 6H, SC*H*2, OC*H*2), 3.70 (s, 3H, C(O)OC*H*3), 3.68 – 3.63 (m, 4H, OC*H*2), 3.49 – 3.36 (m, 4H, NC*H*2), 3.07 (s, 3H, C*H*3), 2.78 (td, 2H, *J* = 6.7, 1.4 Hz, SCH2C*H*2), 2.46 (t, 4H, *J* = 4.7 Hz, NC*H*2); **^{13}C NMR** (126 MHz, $CDCl_3$): δ 157.8, 66.8, 66.4, 53.71, 53.65, 51.5, 49.9, 46.3, 43.8; **IR** (ν_{max}, cm^{-1}) 1681, 1439, 1255, 1141, 1112, 1071, 1036, 1006, 966, 939, 887, 791; **HRMS** (ESI): *m/z* calcd for $C_{13}H_{27}N_4O_6S_2^+$: 399.1367 [M+H]$^+$; found: 399.1359.

B-amino sulfondiimidamide 5n

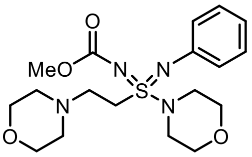

An oven-dried round-bottom flask containing sulfondiimidamide **4e** (96.1 mg, 0.30 mmol, 1.0 equiv.), phenyl boronic acid (91.4 mg, 0.75 mmol, 2.5 equiv.) and $Cu(CH_3CN)_4PF_6$ (55.9 mg, 0.15 mmol, 0.5 equiv.) was sealed under an O_2 atmosphere before anhydrous MeCN (3.0 mL) was added. *N*-methyl piperidine (0.27 mL, 2.70 mmol, 9.0 equiv.) was added and the reaction mixture was stirred at rt for 3 h. The reaction mixture was diluted with sat. aq. NaCl solution (10 mL) and extracted with EtOAc (3 × 10 mL). The combined organic layers were dried over Na_2SO_4, filtered and concentrated *in vacuo.* Purification by flash column chromatography (SiO_2, PE/EtOAc/MeOH, 1:1:0 to 0:1:0 to 0:20:1) afforded the desired product **5n** as a colourless oil (46.8 mg, 40%).

***R*f**= 0.62 (EtOAc/MeOH 5:1); **^{1}H NMR** (400 MHz, $CDCl_3$): δ 7.19 (t, 2H, *J* = 7.7 Hz, Ar*H*), 7.12 (d, 2H, *J* = 7.8 Hz, Ar*H*), 6.96 (t, 1H, *J* = 7.3 Hz, Ar*H*), 3.81 (td, 2H, *J* = 6.8, 2.9 Hz, SC*H*2), 3.74 – 3.54 (m, 11H, OC*H*2, C(O)OC*H*3), 3.47 (ddd, 2H, *J* = 12.4, 6.3, 2.9 Hz, NC*H*2), 3.34 (ddd, 2H, *J* = 12.1, 6.5, 3.0 Hz, NC*H*2), 2.84 (t, 2H, *J* = 6.8 Hz, SCH2C*H*2), 2.50 (q, 4H, *J* = 5.4 Hz, NC*H*2); **^{13}C NMR** (101 MHz, $CDCl_3$): δ 159.0, 142.9, 129.1, 123.7, 122.6, 66.8, 66.7, 53.8, 53.1, 52.1, 49.4, 46.2; **IR** (ν_{max}, cm^{-1}) 1672, 1595, 1489, 1438, 1252, 1115, 1074, 1004, 939, 869, 760, 696; **HRMS** (ESI): *m/z* calcd for $C_{18}H_{29}N_4O_4S^+$: 397.1904 [M+H]$^+$; found: 397.1909.

B-amino sulfondiimidamide 5o

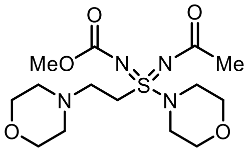

An oven-dried round-bottom flask containing sulfondiimidamide **4e** (294.4 mg, 0.92 mmol, 1.0 equiv.) was sealed and flushed with N_2 before anhydrous MeCN (9.2 mL) was added. DBU (0.21 mL, 1.38 mmol, 1.5 equiv.) was then added followed by acetyl chloride (0.14 mL, 1.84 mmol, 2.0 equiv.). The reaction mixture was stirred at rt for 4 h and then diluted with sat. aq. NaCl solution (20 mL) and extracted with EtOAc (3 × 20 mL). The combined organic layers were dried over Na_2SO_4, filtered and concentrated *in vacuo*. Purification by flash column chromatography (SiO_2, EtOAc/MeOH, 1:0 to 10:1) afforded the desired product **5o** as a colourless oil (190 mg, 57%).

***R*$_f$**= 0.37 (EtOAc/MeOH 10:1); **^{1}H NMR** (400 MHz, $CDCl_3$): δ 3.88 – 3.72 (m, 6H, SC*H*$_2$, OC*H*$_2$), 3.69 (m, 7H, C(O)OC*H*$_3$, OC*H*$_2$), 3.44 – 3.32 (m, 4H, NC*H*$_2$), 2.80 (t, 2H, *J* = 7.0 Hz, SCH$_2$C*H*$_2$), 2.48 (t, 4H, *J* = 4.6 Hz, NC*H*$_2$), 2.14 (s, 3H, C(O)C*H*$_3$); **^{13}C NMR** (101 MHz, $CDCl_3$): δ 178.7, 158.2, 66.9, 66.6, 53.7, 53.5, 51.7, 48.2, 46.4, 27.1; **IR** (ν_{max}, cm^{-1}) 1677, 1646, 1438, 1363, 1235, 1113, 1069, 1038, 1007, 964, 922, 885, 765, 646; **HRMS** (ESI): *m/z* calcd for $C_{14}H_{27}N_4O_5S^+$: 363.1697 [M+H]$^+$; found: 363.1705.

B-amino sulfondiimidamide 5p

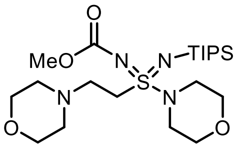

An oven-dried round-bottom flask containing sulfondiimidamide **4e** (230.7 mg, 0.72 mmol, 1.0 equiv.) was sealed and flushed with N_2 before anhydrous THF (7.2 mL) was added. The reaction was cooled to 0 ˚C, NaH (34.4 mg, 0.86 mmol, 1.2 equiv.) was added and the reaction was stirred for 20 min at 0 ˚C. Triisopropylsilyl chloride (0.18 mL, 0.86 mmol, 1.2 equiv.) was then added and the reaction was warmed to rt and stirred for 3 h. The reaction mixture was then diluted with sat. aq. NaCl solution (15 mL) and extracted with EtOAc (3 × 15 mL). The combined organic layers were dried over Na_2SO_4, filtered and concentrated *in vacuo*.

Purification by flash column chromatography (SiO_2, EtOAc/MeOH, 1:0 to 50:1) afforded the desired product **5p** as a colourless oil (137 mg, 40%).

***R*$_f$**= 0.68 (EtOAc/MeOH 10:1); **^{1}H NMR** (400 MHz, $CDCl_3$): δ 3.69 (dt, 8H, *J* = 13.6, 5.0 Hz, OC*H*$_2$), 3.60 (s, 3H, C(O)OC*H*$_3$), 3.55 – 3.45 (m, 1H, SC*H*), 3.38 – 3.17 (m, 5H, SC*H*, NC*H*$_2$), 2.78 (t, 2H, *J* = 7.3 Hz, SCH_2C*H*$_2$), 2.44 (q, 4H, *J* = 4.8 Hz, NC*H*$_2$), 1.04 (s, 21H, SiC*H*, C*H*$_3$); **^{13}C NMR** (101 MHz, $CDCl_3$): δ 159.1, 66.89, 66.87, 53.8, 52.7, 52.6, 51.1, 46.2, 18.4, 13.3; **IR** (ν_{max}, cm^{-1}) 2945, 1668, 1437, 1338, 1253, 1117, 1043, 1006, 965, 934, 882, 788, 678; **HRMS** (ESI): *m/z* calcd for $C_{21}H_{45}N_4O_4SSi^+$: 477.2925 [M+H]$^+$; found: 477.2921.

B-amino sulfondiimidamide 5q

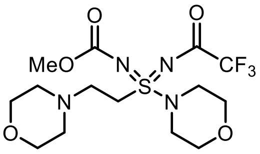

An oven-dried round-bottom flask containing sulfondiimidamide **4e** (160.2 mg, 0.50 mmol, 1.0 equiv.) was sealed and flushed with N_2 before anhydrous MeCN (5.0 mL) was added. DBU (0.11 mL, 0.75 mmol, 1.5 equiv.) was added followed by trifluoroacetic anhydride (0.10 mL, 0.75 mmol, 1.5 equiv.) and the reaction was stirred at rt for 2 h. The reaction mixture was then diluted with sat. aq. NaCl solution (15 mL) and extracted with EtOAc (3 × 15 mL). The combined organic layers were dried over Na_2SO_4, filtered and concentrated *in vacuo*. Purification by flash column chromatography (SiO_2, EtOAc/MeOH, 1:0 to 50:1) afforded the desired product **5q** as a colourless oil (145 mg, 70%).

***R*$_f$**= 0.54 (EtOAc/MeOH 10:1); **^{1}H NMR** (400 MHz, $CDCl_3$): δ 3.89 – 3.71 (m, 6H, OC*H*$_2$, SC*H*$_2$), 3.68 (s, 3H, C(O)OC*H*$_3$), 3.64 (t, 4H, *J* = 4.7 Hz, OC*H*$_2$), 3.40 (q, 4H, *J* = 4.4 Hz, NC*H*$_2$), 2.79 (t, 2H, *J* = 6.4 Hz, SCH_2C*H*$_2$), 2.44 (t, 4H, *J* = 4.5 Hz, NC*H*$_2$); **^{13}C NMR** (101 MHz, $CDCl_3$): δ 162.2 (q, ^2J = 37.9 Hz) 157.0, 115.8 (q, ^1J = 288.8 Hz), 66.7, 66.3, 53.8, 53.6, 51.4, 49.3, 46.2; **^{19}F NMR** (377 MHz, $CDCl_3$) δ -75.6; **IR** (ν_{max}, cm^{-1}) 1683, 1439, 1376, 1257, 1186, 1149, 1115, 1070, 1007, 980, 939, 874, 764, 730, 640; **HRMS** (ESI): *m/z* calcd for $C_{14}H_{24}F_3N_4O_5S^+$: 417.14.14 [M+H]$^+$; found: 417.1407.

B-amino sulfondiimidamide 5r

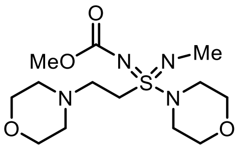

An oven-dried round-bottom flask containing sulfondiimidamide **4e** (333.2 mg, 1.04 mmol, 1.0 equiv.) was sealed and flushed with N_2 before anhydrous THF (5.0 mL) was added. The reaction was cooled to 0 ˚C, NaH (50.0 mg, 1.25 mmol, 1.2 equiv.) was added and the reaction was stirred for 20 min at 0 ˚C. Methyl iodide (68 µL, 1.14 mmol, 1.1 equiv.) was then added and the reaction was warmed to rt and stirred for 18 h. The reaction mixture was then diluted with sat. aq. NaCl solution (20 mL) and extracted with EtOAc (3 × 20 mL). The combined organic layers were dried over Na_2SO_4, filtered and concentrated *in vacuo*. Purification by flash column chromatography (SiO_2, CH_2Cl_2/MeOH 1:0 to 20:1) afforded the desired product **5r** as a colourless oil (191 mg, 55%).

***R*$_f$**= 0.16 (EtOAc/MeOH 10:1); **^{1}H NMR** (400 MHz, $CDCl_3$): δ 3.71 (ddd, 4H, *J* = 7.9, 6.0, 3.3 Hz, OC*H*$_2$), 3.68 – 3.61 (m, 7H, OC*H*$_2$, C(O)OC*H*$_3$), 3.50 (t, 2H, *J* = 7.1 Hz, SC*H*$_2$), 3.33 (ddd, 2H, *J* = 12.4, 6.0, 3.4 Hz, NC*H*$_2$), 3.24 (ddd, 2H, *J* = 12.2, 6.2, 3.3 Hz, NC*H*$_2$), 2.77 (dt, 2H, *J* = 11.4, 6.9 Hz, SCH$_2$C*H*$_2$), 2.71 (s, 3H, C*H*$_3$), 2.45 (q, 4H, *J* = 4.5 Hz, NC*H*$_2$); **^{13}C NMR** (101 MHz, $CDCl_3$): δ 159.4, 66.9, 66.8, 53.7, 52.9, 52.1, 48.6, 46.1, 29.7; **IR** (ν_{max}, cm^{-1}) 1667, 1437, 1272, 1253, 1113, 1068, 1005, 964, 927, 897, 867, 788; **HRMS** (ESI): *m/z* calcd for $C_{13}H_{27}N_4O_4S^+$: 335.1748 $[M+H]^+$; found: 335.1743.

B-amino sulfondiimidamide 5aa

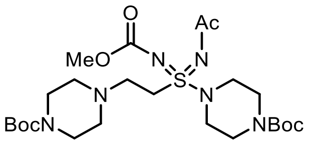

An oven-dried round-bottom flask containing sulfondiimidamide **4f** (259.3 mg, 0.50 mmol, 1.0 equiv.) was sealed and flushed with N_2 before anhydrous THF (5.0 mL) was added. The reaction was cooled to 0 ˚C, NaH (24.0 mg, 0.60 mmol, 60% dispersion in mineral oil, 1.2 equiv.) was added and the reaction was stirred for 20 min at 0 ˚C. Acetyl chloride (43 µL, 0.60 mmol, 1.2 equiv.) was then added and the reaction was warmed to rt and stirred for 2 h. The reaction mixture was then diluted with sat. aq. NaCl solution (15 mL) and extracted with

EtOAc (3 × 15 mL). The combined organic layers were dried over Na_2SO_4, filtered and concentrated *in vacuo*. Purification by flash column chromatography (SiO_2, EtOAc) afforded the desired product **5aa** as a colourless oil (114 mg, 40%).

***R*f**= 0.19 (EtOAc/MeOH 5:1); **^{1}H NMR** (400 MHz, $CDCl_3$): δ 3.87 – 3.71 (m, 2H, SC*H*$_2$), 3.66 (s, 3H, C(O)OC*H*$_3$), 3.51 (dd, 4H, *J* = 6.6, 3.7 Hz, NC*H*$_2$), 3.44 – 3.35 (m, 4H, NC*H*$_2$), 3.33 (dd, 4H, *J* = 6.3, 4.0 Hz, NC*H*$_2$), 2.79 (t, 2H, *J* = 7.1 Hz, SCH$_2$C*H*$_2$), 2.44 – 2.34 (m, 4H, NC*H*$_2$), 2.11 (s, 3H, C(O)C*H*$_3$), 1.43 (s, 9H, C(C*H*$_3$)$_3$), 1.43 (s, 9H, C(C*H*$_3$)$_3$); **^{13}C NMR** (101 MHz, $CDCl_3$): δ 178.7, 158.1, 154.7, 154.2, 80.7, 80.0, 53.4, 53.0, 51.3, 48.5, 46.0, 43.9, 41.3, 28.5, 28.4, 27.1; **IR** (ν_{max}, cm^{-1}) 1693, 1651, 1457, 1420, 1365, 1281, 1245, 1169, 1126, 1041, 1002, 968, 920, 770, 732; **HRMS** (ESI): *m/z* calcd for $C_{24}H_{45}N_6O_7S^+$: 561.3065 [M+H]$^+$; found: 561.3046.

B-amino sulfondiimidamide 5ab

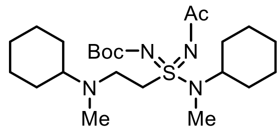

An oven-dried round-bottom flask containing sulfondiimidamide **4g** (82.9 mg, 0.20 mmol, 1.0 equiv.) was sealed and flushed with N_2 before anhydrous MeCN (2.0 mL) was added. DBU (90 µL, 0.60 mmol, 3.0 equiv.) was added followed by acetyl chloride (36 µL, 0.50 mmol, 2.5 equiv.) and the reaction was stirred at rt for 2 h. The reaction mixture was then diluted with sat. aq. NaCl solution (10 mL) and extracted with EtOAc (3 × 10 mL). The combined organic layers were dried over Na_2SO_4, filtered and concentrated *in vacuo*. Purification by flash column chromatography (SiO_2, EtOAc) afforded the desired product **5ab** as a colourless oil (31.3 mg, 34%).

***R*f**= 0.31 (EtOAc/MeOH 10:1); **^{1}H NMR** (400 MHz, $CDCl_3$): δ 3.78 (tdd, 3H, *J* = 19.6, 8.5, 5.1 Hz, alkylC*H*), 2.87 (s, 3H, NC*H*$_3$), 2.24 (s, 3H, NC*H*$_3$), 2.09 (s, 3H, C(O)C*H*$_3$), 1.77 (dd, 8H, *J* = 18.0, 10.7 Hz, alkylC*H*), 1.61 (td, 3H, *J* = 13.7, 4.2 Hz, alkylC*H*), 1.44 (s, 9H, C(C*H*$_3$)$_3$), 1.32 (ddt, 4H, *J* = 13.6, 10.2, 3.7 Hz, alkylC*H*), 1.24 – 1.11 (m, 4H, alkylC*H*), 1.11 – 0.90 (m, 2H, alkylC*H*); **^{13}C NMR** (101 MHz, $CDCl_3$): δ 178.7, 157.2, 80.1, 63.0, 56.5, 49.6, 47.2, 38.1, 31.0, 30.9, 30.6, 30.0, 29.4, 29.1, 28.9, 28.2, 27.2, 26.3, 26.0, 25.94, 25.90, 25.87, 25.7, 25.4 (note: for 2 secondary carbons attached to tertiary carbon in cyclohexane ring, 2 peaks were found instead of 1 due to the loss of symmetry caused by the stereogenic sulfur atom); **IR** (ν_{max}, cm^{-1})

2929, 1645, 1451, 1405, 1390, 1364, 1286, 1245, 1155, 1040, 998, 944, 893, 868, 850, 806, 659; **HRMS** (ESI): *m/z* calcd for $C_{23}H_{45}N_4O_3S^+$: 457.3207 $[M+H]^+$; found: 457.3206.

B-amino sulfondiimidamide 5ac

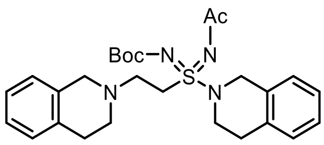

An oven-dried round-bottom flask containing sulfondiimidamide **4h** (90.9 mg, 0.20 mmol, 1.0 equiv.) was sealed and flushed with N_2 before anhydrous MeCN (2.0 mL) was added. DBU (90 µL, 0.60 mmol, 3.0 equiv.) was added followed by acetyl chloride (36 µL, 0.50 mmol, 2.5 equiv.) and the reaction was stirred at rt for 2 h. The reaction mixture was then diluted with sat. aq. NaCl solution (10 mL) and extracted with EtOAc (3 × 10 mL). The combined organic layers were dried over Na_2SO_4, filtered and concentrated *in vacuo*. Purification by flash column chromatography (SiO_2, PE/EtOAc, 1:1) afforded the desired product **5ac** as a white solid (50.1 mg, 50%).

***R*$_f$**= 0.54 (EtOAc); **m.p.** 112 – 114 ˚C (CH_2Cl_2) **^{1}H NMR** (400 MHz, $CDCl_3$): δ 7.19 – 7.13 (m, 2H, Ar*H*), 7.09 (ddd, 3H, *J* = 6.8, 4.9, 2.5 Hz, Ar*H*), 7.04 (dt, 2H, *J* = 6.4, 3.1 Hz, Ar*H*), 6.93 (dd, 1H, *J* = 7.2, 1.8 Hz, Ar*H*), 4.59 (d, 2H, *J* = 2.6 Hz, NC*H*$_2$), 3.98 (ddt, 2H, *J* = 46.7, 14.0, 7.1 Hz, SC*H*$_2$), 3.78 – 3.62 (m, 2H, alkylC*H*), 3.60 (s, 2H, NC*H*$_2$), 2.95 (q, 4H, *J* = 6.5 Hz, alkylC*H*), 2.81 (t, 2H, *J* = 5.8 Hz, alkylC*H*), 2.76 – 2.65 (m, 2H, alkylC*H*), 2.15 (s, 3H, C(O)C*H*$_3$), 1.44 (s, 9H, C(C*H*$_3$)$_3$); **^{13}C NMR** (101 MHz, $CDCl_3$): δ 178.7, 156.9, 134.0, 133.8, 133.4, 132.0, 128.9, 128.7, 127.1, 126.6, 126.49, 126.45, 126.4, 125.8, 80.6, 55.9, 51.0, 50.9, 49.3, 47.8, 43.8, 29.3, 28.9, 28.1, 27.1; **IR** (ν_{max}, cm^{-1}) 1648, 1391, 1246, 1155, 1097, 1039, 1018, 985, 945, 935, 903, 841, 787, 740, 703; **HRMS** (ESI): *m/z* calcd for $C_{27}H_{37}N_4O_3S^+$: 497.2581 $[M+H]^+$; found: 497.2571.

2.1.5 General procedure C – synthesis of vinyl sulfondiimidamides (6)

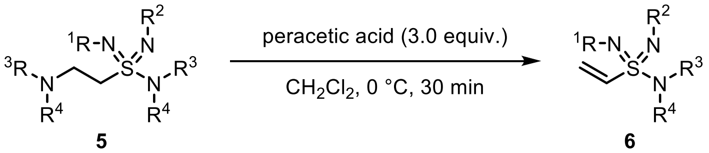

An oven-dried microwave vial containing sulfondiimidamide **5** was sealed and flushed with N_2 before CH_2Cl_2 (sulfondiimidamide conc. 0.05 M) was added. The reaction was cooled to 0 ˚C, peroxyacetic acid (1.0 or 3.0 equiv., 35wt.% solution in diluted acetic acid) was added and the reaction mixture stirred at 0 ˚C for 30 minutes. After this time, the reaction was quenched with $Na_2S_2O_4$ (10 mL) and diluted with EtOAc (10 mL). The aqueous phase was separated and extracted with EtOAc (3 × 10 mL). The combined organic layers were dried over anhydrous Na_2SO_4, filtered and concentrated *in vacuo*. The crude residue was purified by flash column chromatography with the appropriate solvent system to afford the desired vinyl sulfondiimidamide.

Vinyl sulfondiimidamide 6a

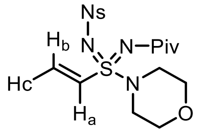

Following general procedure C, sulfondiimidamide **5a** (53.2 mg, 0.10 mmol, 1.0 equiv.), CH_2Cl_2 (2.0 mL) and peroxyacetic acid (58 µL, 0.30 mmol, 3.0 equiv.) were combined and stirred at 0 ˚C for 30 min. Purification by flash column chromatography (SiO_2, PE/EtOAc, 1:1 to 1:2) afforded vinyl sulfondiimidamide **6a** as an off-white solid (36.7 mg, 84%).

***R*f**= 0.61 (EtOAc); **m.p.** 110 – 112 ˚C (CH_2Cl_2); **^{1}H NMR** (400 MHz, $CDCl_3$): δ 8.30 (d, 2H, *J* = 9.1, Ar*H*), 8.13 (d, 2H, *J* = 9.1, Ar*H*), 6.59 (dd, 2H, *J* = 0.9, 4.0, C*Ha*, C*Hb*), 6.33 (app.t, 1H, *J* = 4.0, C*H*c), 3.79 (t, 4H, *J* = 4.7, OC*H2*), 3.43 – 3.28 (m, 4H, NC*H*2) 1.00 (s, 9H, C(C*H*3)3); **^{13}C NMR** (101 MHz, $CDCl_3$): δ 185.9, 149.8, 148.7, 132.5, 132.3, 128.4, 124.0, 66.3, 45.9, 42.0, 27.5; **IR** (ν_{max}, cm^{-1}) 1654, 1531, 1351, 1301, 1162, 1111, 1087, 988, 925, 855, 763, 747, 686; **HRMS** (ESI): *m/z* calcd for $C_{17}H_{25}N_4O_6S_2^+$: 445.1210 [M+H]$^+$; found: 445.1217.

Vinyl sulfondiimidamide 6b

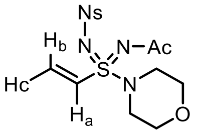

Following general procedure C, sulfondiimidamide **5b** (49.0 mg, 0.10 mmol, 1.0 equiv.), CH_2Cl_2 (2.0 mL) and peroxyacetic acid (58 µL, 0.30 mmol, 3.0 equiv.) were combined at 0 ˚C for 30 min. Purification by flash column chromatography (SiO_2, PE/EtOAc, 1:3) afforded vinyl sulfondiimidamide **6b** as a colourless oil (27.6 mg, 70%).

***R*f**= 0.44 (EtOAc); **^{1}H NMR** (400 MHz, $CDCl_3$): δ 8.31 (d, 2H, *J* = 9.4 Hz, Ar*H*), 8.13 (d, 2H, *J* = 8.5 Hz, Ar*H*), 6.59 – 6.53 (m, 2H, C*Ha,* C*Hb*), 6.34 (dd, 1H, *J* = 5.6, 2.4 Hz, C*Hc*), 3.79 (t, 4H, *J* = 7.4 Hz, OC*H2*), 3.37 (qt, 4H, *J* = 12.1, 4.7 Hz, NC*H2*) 1.88 (s, 3H, C*H3*); **^{13}C NMR** (101 MHz, $CDCl_3$): δ 177.4, 150.0, 148.3, 132.6, 132.3, 128.6, 123.9, 66.2, 45.9, 26.6; **IR** (ν_{max}, cm^{-1}) 1610, 1533, 1353, 1300, 1236, 1161, 1110, 1067, 1010, 936, 745; **HRMS** (ESI): *m/z* calcd for $C_{14}H_{19}N_4O_6S_2^+$: 403.0741 $[M+H]^+$; found: 403.0733.

Vinyl sulfondiimidamide 6c

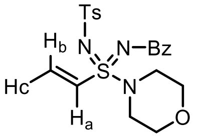

Following general procedure C, sulfondiimidamide **5c** (62.4 mg, 0.12 mmol, 1.0 equiv.), CH_2Cl_2 (2.4 mL) and peroxyacetic acid (69 µL, 0.36 mmol, 3.0 equiv.) were combined at 0 ˚C for 30 min. Purification by flash column chromatography (SiO_2, PE/EtOAc, 1:1) afforded vinyl sulfondiimidamide **6c** as a white solid (32.3 mg, 62%).

***R*f**= 0.60 (EtOAc); **m.p.** 56 – 58 ˚C (CH_2Cl_2); **^{1}H NMR** (400 MHz, $CDCl_3$): δ 7.73 (td, 4H, *J* = 4.6, 2.1 Hz, Ar*H*), 7.52 – 7.39 (m, 1H, Ar*H*), 7.31 (t, 2H, *J* = 7.6 Hz, Ar*H*), 6.95 (d, 2H, *J* = 8.0 Hz, Ar*H*), 6.74 – 6.59 (m, 2H, C*Ha, CHb*), 6.33 (d, 1H, *J* = 7.3 Hz, C*Hc*), 3.81 (ddd, 4H, *J* = 5.6, 3.7, 1.6 Hz, OC*H2*), 3.57 – 3.39 (m, 4H, NC*H2*) 2.15 (s, 3H, C*H3*); **^{13}C NMR** (101 MHz, $CDCl_3$): δ 171.1, 142.9, 139.6, 134.7, 133.2, 132.5, 131.9, 129.5, 129.2, 127.9, 127.2, 66.4, 45.9, 21.5; **IR** (ν_{max}, cm^{-1}) 1638, 1451, 1312, 1279, 1258, 1157, 1109, 1068, 937, 733, 712, 666; **HRMS** (ESI): *m/z* calcd for $C_{20}H_{24}N_3O_4S_2^+$: 434.1203 $[M+H]^+$; found: 434.1196.

Vinyl sulfondiimidamide 6d

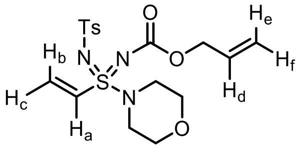

Following general procedure C, sulfondiimidamide **5d** (45.1 mg, 0.09 mmol, 1.0 equiv.), CH_2Cl_2 (1.8 mL) and peroxyacetic acid (16 µL, 0.09 mmol, 1.0 equiv.) were combined at 0 ˚C for 30 min. Purification by flash column chromatography (SiO_2, PE/EtOAc, 1:1) afforded vinyl sulfondiimidamide **6d** as a white solid (15.2 mg, 43%).

***R*$_f$**= 0.72 (EtOAc); **m.p.** 54 – 56 ˚C (CH_2Cl_2); **^{1}H NMR** (400 MHz, $CDCl_3$): δ 7.84 (d, 2H, *J* = 8.2 Hz, Ar*H*), 7.25 (d, 2H, *J* = 7.5 Hz, Ar*H*), 6.53 (d, 1H, *J* = 16.0 Hz, C*H$_b$*), 6.42 (dd, 1H, *J* = 15.9, 9.1 Hz, C*H$_a$*), 6.27 (d, 1H, *J* = 9.1 Hz, C*H$_c$*), 5.79 (ddt, 1H, *J* = 16.4, 11.0, 5.8 Hz, C*H$_d$*), 5.34 – 5.15 (m, 2H, C*H$_e$, CH$_f$*), 4.23 (d, 2H, *J* = 5.8 Hz, C(O)OC*H$_2$*), 3.74 (t, 4H, *J* = 4.7 Hz, OC*H$_2$*), 3.40 (tq, 4H, *J* = 12.2, 6.0 Hz, NC*H$_2$*), 2.39 (s, 3H, C*H$_3$*); **^{13}C NMR** (101 MHz, $CDCl_3$): δ 156.1, 143.2, 139.8, 132.9, 132.2, 132.1, 129.3, 127.4, 118.5, 67.1, 66.4, 46.0, 21.7; **IR** (ν_{max}, cm^{-1}) 1684, 1455, 1377, 1293, 1256, 1161, 1112, 1082, 975, 928, 737; **HRMS** (ESI): *m/z* calcd for $C_{17}H_{24}N_3O_5S_2^+$: 414.1152 $[M+H]^+$; found: 414.1154.

Vinyl sulfondiimidamide 6e

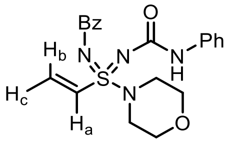

Following general procedure C, sulfondiimidamide **5e** (48.6 mg, 0.10 mmol, 1.0 equiv.), CH_2Cl_2 (2.0 mL) and peroxyacetic acid (58 µL, 0.30 mmol, 3.0 equiv.) were combined and stirred at 0 ˚C for 30 min. Purification by flash column chromatography (SiO_2, PE/EtOAc, 1:1) afforded vinyl sulfondiimidamide **6e** as a white solid (21.4 mg, 54%).

***R*$_f$**= 0.64 (EtOAc); **m.p.** 136 – 138 ˚C (CH_2Cl_2); **^{1}H NMR** (400 MHz, $CDCl_3$): δ 8.14 (d, 2H, *J* = 6.8 Hz, Ar*H*), 7.50 (t, 1H, *J* = 7.5 Hz, Ar*H*), 7.45 – 7.36 (m, 4H, Ar*H*), 7.32 – 7.21 (m, 3H, Ar*H*), 7.01 (t, 1H, *J* = 7.3 Hz, Ar*H*), 6.90 (dd, 1H, *J* = 16.2, 9.4 Hz, C*H$_a$*), 6.69 (d, 1H, *J* = 16.3 Hz, C*H$_b$*), 6.31 (d, 1H, *J* = 9.3 Hz, C*H$_c$*), 3.81 (t, 4H, *J* = 4.7 Hz, OC*H$_2$*), 3.46 (qt, 4H, *J* = 12.1, 4.7 Hz, NC*H$_2$*); **^{13}C NMR** (101 MHz, $CDCl_3$): δ 172.7, 155.5, 138.9, 135.6, 132.8, 132.4, 130.7, 129.7, 129.0,

128.2, 123.3, 118.9, 66.5, 46.1; **IR** (ν_{max}, cm^{-1}) 1635, 1598, 1531, 1439, 1314, 1287, 1224, 1138, 1111, 930, 757, 713; **HRMS** (ESI): *m/z* calcd for $C_{20}H_{23}N_4O_3S^+$: 399.1485 [M+H]$^+$; found: 399.1487.

Vinyl sulfondiimidamide 6f

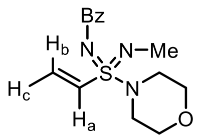

Following general procedure C, sulfondiimidamide **5f** (38.1 mg, 0.10 mmol, 1.0 equiv.), CH_2Cl_2 (2.0 mL) and peroxyacetic acid (58 µL, 0.30 mmol, 3.0 equiv.) were combined at 0 ˚C for 30 min. The crude residue was dissolved in acetone (0.5 mL) and water (0.5 mL), $NaHCO_3$ (12.6 mg, 0.15 mmol, 1.5 equiv.) was added and the reaction was stirred for 5 h at rt. Purification by flash column chromatography (SiO_2, EtOAc) afforded vinyl sulfondiimidamide **6f** as a colourless oil (19.1 mg, 66%).

***R*f**= 0.17 (EtOAc); **^{1}H NMR** (600 MHz, $CDCl_3$): δ 8.16 – 8.12 (m, 2H, Ar*H*), 7.51 – 7.46 (m, 1H, Ar*H*), 7.43 – 7.37 (m, 2H, Ar*H*), 6.55 (dd, 1H, *J* = 16.5, 9.8 Hz, C*Ha*), 6.31 (dd, 1H, *J* = 16.5, 0.7 Hz, C*Hb*), 6.11 (dd, 1H, *J* = 9.8, 0.7 Hz, C*Hc*), 3.81 (qdd, 4H, *J* = 11.6, 6.3, 3.1 Hz, OCH_2), 3.36 – 3.28 (m, 2H, NCH_2), 3.21 (dddd, 2H, *J* = 12.1, 6.4, 3.2, 1.0 Hz, NCH_2), 2.86 (s, 3H, CH_3); **^{13}C NMR** (151 MHz, $CDCl_3$): δ 172.9, 136.5, 132.5, 131.9, 129.4, 128.1, 127.8, 66.8, 46.0, 29.8; **IR** (ν_{max}, cm^{-1}) 1627, 1450, 1312, 1287, 1254, 1112, 1067, 1026, 925, 714; **HRMS** (ESI): *m/z* calcd for $C_{14}H_{20}N_3O_2S^+$: 294.1271 [M+H]$^+$; found: 294.1267.

Vinyl sulfondiimidamide 6g

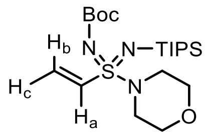

Following general procedure C, sulfondiimidamide **5g** (51.9 mg, 0.10 mmol, 1.0 equiv.), CH_2Cl_2 (2.0 mL) and peroxyacetic acid (58 µL, 0.30 mmol, 3.0 equiv.) were combined and stirred at 0 ˚C for 30 min. Purification by flash column chromatography (SiO_2, PE/EtOAc, 4:1 to 3:1) afforded vinyl sulfondiimidamide **6g** as a white solid (33.9 mg, 76%).

***R*f**= 0.56 (PE/EtOAc 1:1); **m.p.** 68 – 70 ˚C (CH_2Cl_2); **^{1}H NMR** (400 MHz, $CDCl_3$): δ 6.46 (dd, 1H, *J* = 16.1, 9.4 Hz, C*Ha*), 6.21 (d, 1H, *J* = 16.2 Hz, C*Hb*), 6.00 (d, 1H, *J* = 9.4 Hz, C*Hc*), 3.71 (qdd, 4H, *J* = 11.5, 6.1, 3.2 Hz, OC*H2*), 3.21 (ddd, 2H, *J* = 11.9, 6.1, 3.2 Hz, NC*H2*), 3.08 (ddd, 2H, *J* = 11.6, 6.0, 3.2 Hz, NC*H2*) 1.42 (s, 9H, C(CH_3)$_3$), 1.06 (s, 21H, SiC*H*, C*H3*); **^{13}C NMR** (101 MHz, $CDCl_3$): δ 158.0, 136.2, 126.3, 79.0, 66.6, 45.9, 28.3, 18.3, 13.2; **IR** ($ν_{max}$, cm^{-1}) 2865, 1658, 1453, 1277, 1251, 1155, 1066, 925, 867, 732; **HRMS** (ESI): *m/z* calcd for $C_{20}H_{42}N_3O_3SSi^+$: 432.2711 $[M+H]^+$; found: 432.2714.

Vinyl sulfondiimidamide 6h

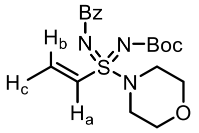

Following general procedure C, sulfondiimidamide **5h** (60.7 mg, 0.13 mmol, 1.0 equiv.), CH_2Cl_2 (2.6 mL) and peroxyacetic acid (74 µL, 0.39 mmol, 3.0 equiv.) were combined at 0 ˚C for 30 min. Purification by flash column chromatography (SiO_2, PE/EtOAc, 1:1) afforded vinyl sulfondiimidamide **6h** as a colourless oil (35.1 mg, 72%).

***R*f**= 0.70 (EtOAc); **^{1}H NMR** (400 MHz, $CDCl_3$): δ 8.12 (d, 2H, *J* = 7.4 Hz, Ar*H*), 7.49 (t, 1H, *J* = 7.4 Hz, Ar*H*), 7.39 (t, 2H, *J* = 7.4 Hz, Ar*H*), 6.77 (dd, 1H, *J* = 16.2, 9.2 Hz, C*Ha*), 6.66 (d, 1H, *J* = 16.3 Hz, C*Hb*), 6.28 (d, 1H, *J* = 9.1 Hz, C*Hc*), 3.79 (dd, 4H, *J* = 5.9, 3.6 Hz, OC*H2*), 3.42 (dd, 4H, *J* = 5.9, 3.6 Hz, NC*H2*), 1.44 (s, 9H, C(CH_3)$_3$); **^{13}C NMR** (101 MHz, $CDCl_3$): δ 172.1, 156.3, 135.6, 132.7, 132.3, 130.8, 129.7, 128.2, 81.0, 66.6, 46.1, 28.1; **IR** ($ν_{max}$, cm^{-1}) 1673, 1635, 1451, 1367, 1291, 1270, 1248, 1147, 1112, 930, 714; **HRMS** (ESI): *m/z* calcd for $C_{18}H_{26}N_3O_4S^+$: 380.1639 $[M+H]^+$; found: 380.1631.

Vinyl sulfondiimidamide 6i

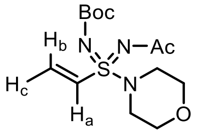

Following general procedure C, sulfondiimidamide **5i** (202.3 mg, 0.50 mmol, 1.0 equiv.), CH_2Cl_2 (10.0 mL) and peroxyacetic acid (0.29 mL, 1.50 mmol, 3.0 equiv.) were combined and

stirred at 0 ˚C for 30 min. Purification by flash column chromatography (SiO_2, PE/EtOAc, 3:1) afforded vinyl sulfondiimidamide **6i** as a colourless oil (138 mg, 75%).

***R*f**= 0.28 (EtOAc); **^{1}H NMR** (400 MHz, $CDCl_3$): δ 6.67 – 6.49 (m, 2H, C*Ha*, C*Hb*), 6.21 (d, 1H, *J* = 8.7 Hz, C*Hc*), 3.78 – 3.71 (m, 4H, OC*H*2), 3.35 – 3.28 (m, 4H, NC*H*2), 2.13 (s, 3H, C*H*3), 1.45 (s, 9H, C(C*H*3)3); **^{13}C NMR** (101 MHz, $CDCl_3$): δ 178.1, 156.5, 132.4, 130.6, 80.9, 66.5, 46.0, 28.2, 27.0; **IR** (ν_{max}, cm^{-1}) 1670, 1648, 1263, 1108, 1038, 863, 810, 750, 656; **HRMS** (ESI): *m/z* calcd for $C_{13}H_{24}N_3O_4S^+$: 318.1482 [M+H]$^+$; found: 318.1478.

Vinyl sulfondiimidamide 6j

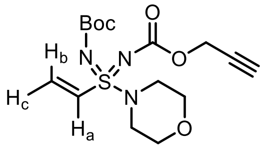

Following general procedure C, sulfondiimidamide **5j** (44.4 mg, 0.10 mmol, 1.0 equiv.), CH_2Cl_2 (2.0 mL) and peroxyacetic acid (19 µL, 0.10 mmol, 1.0 equiv.) were combined and stirred at 0 ˚C for 30 min. Purification by flash column chromatography (SiO_2, PE/EtOAc, 1:1) afforded vinyl sulfondiimidamide **6j** as a colourless oil (16.2 mg, 45%).

***R*f**= 0.64 (EtOAc); **^{1}H NMR** (400 MHz, $CDCl_3$): δ 6.64 – 6.47 (m, 2H, C*Ha*, C*Hb*), 6.26 (d, 1H, *J* = 7.9 Hz, C*Hc*), 4.66 (d, 2H *J* = 2.5 Hz, C(O)OC*H*2), 3.75 (t, 4H, *J* = 4.7 Hz, OC*H*2), 3.35 (td, 4H, *J* = 4.1, 1.4 Hz, NC*H*2), 2.43 (t, 1H, *J* = 2.4 Hz, CC*H*), 1.45 (s, 9H, C(C*H*3)3); **^{13}C NMR** (101 MHz, $CDCl_3$): δ 156.2, 155.8, 132.4, 131.2, 81.3, 78.2, 74.9, 66.4, 53.7, 45.9, 38.1; **IR** (ν_{max}, cm^{-1}) 1687, 1455, 1370, 1291, 1244, 1158, 1113, 1068, 985, 932, 863, 736, 645; **HRMS** (ESI): *m/z* calcd for $C_{15}H_{24}N_3O_5S^+$: 358.1431 [M+H]$^+$; found: 358.1440.

Vinyl sulfondiimidamide 6k

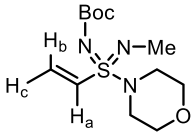

Following general procedure C, sulfondiimidamide **5k** (113.0 mg, 0.30 mmol, 1.0 equiv.), CH_2Cl_2 (6.0 mL) and peroxyacetic acid (0.17 mL, 0.90 mmol, 3.0 equiv.) were combined at 0 ˚C for 30 min. The crude residue was dissolved in acetone (1.5 mL) and water (1.5 mL), $NaHCO_3$ (37.8 mg, 0.45 mmol, 1.5 equiv.) was added and the reaction was stirred for 4 h at rt.

Purification by flash column chromatography (SiO_2, EtOAc) afforded vinyl sulfondiimidamide **6k** as a white solid (114 mg, 60%).

***R*$_f$**= 0.28 (EtOAc); **m.p.** 58 – 60 ˚C (CH_2Cl_2); **^{1}H NMR** (400 MHz, $CDCl_3$): δ 6.40 (dd, 1H, *J* = 16.6, 9.7 Hz, C*H$_a$*), 6.21 (d, 1H, *J* = 16.6 Hz, C*H$_b$*), 6.06 (d, 1H, *J* = 9.8 Hz, C*H$_c$*), 3.73 (qdd, 4H, *J* = 11.6, 6.1, 3.2 Hz, OC*H$_2$*), 3.22 (dddd, 2H, *J* = 12.0, 6.4, 3.2, 1.0 Hz, NC*H$_2$*), 3.08 (dddd, 2H, *J* = 12.0, 6.4, 3.2, 1.0 Hz, NC*H$_2$*), 2.79 (s, 3H, NC*H$_3$*), 1.45 (s, 9H, C(C*H$_3$*)$_3$); **^{13}C NMR** (101 MHz, $CDCl_3$): δ 157.9, 132.4, 127.7, 79.4, 66.6, 46.0, 29.1, 28.3; **IR** (ν_{max}, cm^{-1}) 1666, 1366, 1280, 1247, 1158, 1113, 927, 907; **HRMS** (ESI): *m/z* calcd for $C_{12}H_{24}N_3O_3S^+$: 290.1533 [M+H]$^+$; found: 290.1523.

Vinyl sulfondiimidamide 6l

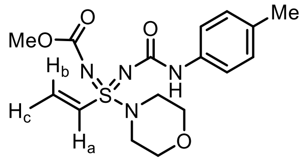

Following general procedure C, sulfondiimidamide **5l** (190.5 mg, 0.42 mmol, 1.0 equiv.), CH_2Cl_2 (8.4 mL) and peroxyacetic acid (0.24 mL, 1.50 mmol, 3.0 equiv.) were combined and stirred at 0 ˚C for 30 min. Purification by flash column chromatography (SiO_2, PE/EtOAc, 1:1) afforded vinyl sulfondiimidamide **6l** as a white solid (104 mg, 67%).

***R*$_f$**= 0.41 (EtOAc); **m.p.** 64 – 68 ˚C (CH_2Cl_2); **^{1}H NMR** (400 MHz, $CDCl_3$): δ 7.29 (d, 2H, *J* = 8.1 Hz, Ar*H*), 7.04 (d, 2H, *J* = 7.9 Hz, Ar*H*), 6.68 (dd, 1H, *J* = 16.2, 9.4 Hz, C*H$_a$*), 6.55 (d, 1H, *J* = 16.1 Hz, C*H$_b$*), 6.22 (d, 1H, *J* = 9.2 Hz, C*H$_c$*), 3.73 (t, 4H, *J* = 4.6 Hz, OC*H$_2$*), 3.66 (s, 3H, C(O)OC*H$_3$*), 3.33 (dq, 4H, *J* = 12.2, 6.1 Hz, NC*H$_2$*), 2.25 (s, 3H, ArC*H$_3$*); **^{13}C NMR** (101 MHz, $CDCl_3$): δ 158.2, 155.2, 136.3, 132.6, 132.3, 130.8, 129.3, 118.8, 66.3, 53.3, 45.9, 20.8; **IR** (ν_{max}, cm^{-1}) 1676, 1655, 1573, 1437, 1276, 1223, 1110, 973, 934, 873; **HRMS** (ESI): *m/z* calcd for $C_{16}H_{23}N_4O_4S^+$: 367.1435 [M+H]$^+$; found: 367.1420.

Vinyl sulfondiimidamide 6m

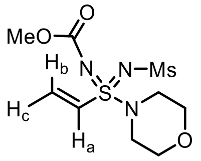

Following general procedure C, sulfondiimidamide **5m** (199.2 mg, 0.50 mmol, 1.0 equiv.), CH_2Cl_2 (10.0 mL) and peroxyacetic acid (0.29 mL, 1.50 mmol, 3.0 equiv.) were combined and stirred at 0 ˚C for 30 min. Purification by flash column chromatography (SiO_2, PE/EtOAc, 1:1) afforded vinyl sulfondiimidamide **6m** as a white solid (132 mg, 84%).

***R*f**= 0.38 (EtOAc); **m.p.** 86 – 88 ˚C (CH_2Cl_2); **^{1}H NMR** (400 MHz, $CDCl_3$): δ 6.67 – 6.51 (m, 2H, C*Ha,* C*Hb*), 6.36 (d, 1H, *J* = 7.7 Hz, C*Hc*), 3.77 (t, 4H, *J* = 4.7 Hz, OC*H2*), 3.71 (s, 3H, C(O)OC*H3*), 3.36 (qt, 4H, *J* = 12.4, 4.7 Hz, NC*H2*), 3.13 (s, 3H, SO_2CH_3); **^{13}C NMR** (101 MHz, $CDCl_3$): δ 157.5, 132.6, 132.1, 66.3, 53.8, 46.0, 44.0; **IR** (ν_{max}, cm^{-1}) 1687, 1438, 1384, 1275, 1209, 1148, 1111, 1068, 971, 926, 885, 795, 735; **HRMS** (ESI): *m/z* calcd for $C_9H_{18}N_3O_5S_2^+$: 312.0682 [M+H]$^+$; found: 312.0688.

Vinyl sulfondiimidamide 6n

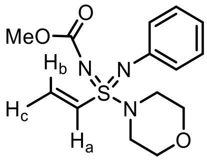

Following general procedure C, sulfondiimidamide **5n** (47.6 mg, 0.12 mmol, 1.0 equiv.), CH_2Cl_2 (2.2 mL) and peroxyacetic acid (66 µL, 0.66 mmol, 3.0 equiv.) were combined and stirred at 0 ˚C for 30 min. Purification by flash column chromatography (SiO_2, PE/EtOAc, 1:1) afforded vinyl sulfondiimidamide **6n** as a colourless oil (25.2 mg, 71%).

***R*f**= 0.52 (EtOAc); **^{1}H NMR** (400 MHz, $CDCl_3$): δ 7.25 – 7.16 (m, 4H, Ar*H*), 6.99 (tt, 1H, *J* = 7.0, 1.7 Hz, Ar*H*), 6.56 (dd, 1H, *J* = 16.4, 9.5 Hz, C*Ha*), 6.40 (d, 1H, *J* = 16.6 Hz, C*Hb*), 6.21 (d, 1H, *J* = 9.7 Hz, C*Hc*), 3.79 – 3.65 (m, 4H, OC*H2*), 3.64 (s, 3H, C(O)OC*H3*), 3.36 (ddd, 2H, *J* = 12.1, 6.3, 3.2 Hz, NC*H2*), 3.20 (ddd, 2H, *J* = 11.9, 6.4, 3.1 Hz, NC*H2*); **^{13}C NMR** (101 MHz, $CDCl_3$): δ 158.7, 142.3, 132.6, 129.2, 129.1, 124.0, 122.8, 66.5, 53.1, 46.1; **IR** (ν_{max}, cm^{-1}) 1680, 1595, 1489, 1436, 1298, 1274, 1252, 1113, 1078, 1060, 965, 926, 876, 787, 696; **HRMS** (ESI): *m/z* calcd for $C_{14}H_{20}N_3O_3S^+$: 310.1220 [M+H]$^+$; found: 310.1235.

Vinyl sulfondiimidamide 6o

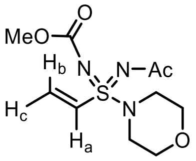

Following general procedure C, sulfondiimidamide **5o** (137.7 mg, 0.38 mmol, 1.0 equiv.), CH_2Cl_2 (7.6 mL) and peroxyacetic acid (0.22 mL, 1.14 mmol, 3.0 equiv.) were combined and stirred at 0 ˚C for 30 min. Purification by flash column chromatography (SiO_2, PE/EtOAc, 1:1) afforded vinyl sulfondiimidamide **6o** as a colourless oil (65.7 mg, 58%).

***R*f**= 0.29 (EtOAc); **¹H NMR** (500 MHz, $CDCl_3$): δ 6.60 (m, 2H, C*Ha,* C*Hb*), 6.26 (dd, 1H, *J* = 6.3, 2.1 Hz, C*Hc*), 3.75 (t, 4H, *J* = 4.7 Hz, OC*H2*), 3.68 (s, 3H, C(O)OC*H3*), 3.31 (dd, 4H, *J* = 6.0, 3.6 Hz, NC*H2*), 2.14 (s, 3H, C(O)C*H3*); **¹³C NMR** (126 MHz, $CDCl_3$): δ 178.3, 157.9, 132.1, 131.3, 66.4, 53.5, 45.9, 27.0; **IR** (ν_{max}, cm^{-1}) 1685, 1654, 1438, 1365, 1286, 1242, 1112, 1069, 1046, 971, 924, 884, 741; **HRMS** (ESI): *m/z* calcd for $C_{10}H_{18}N_3O_4S^+$: 276.1013 $[M+H]^+$; found: 276.1011.

Vinyl sulfondiimidamide 6p

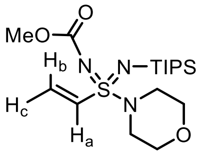

Following general procedure C, sulfondiimidamide **5p** (47.7 mg, 0.10 mmol, 1.0 equiv.), CH_2Cl_2 (2.0 mL) and peroxyacetic acid (58 µL, 0.30 mmol, 3.0 equiv.) were combined at 0 ˚C for 30 min. Purification by flash column chromatography (SiO_2, PE/EtOAc, 1:1) afforded vinyl sulfondiimidamide **6p** as a white solid (21.7 mg, 56%).

***R*f**= 0.68 (EtOAc); **m.p.** 58 – 60 ˚C (CH_2Cl_2); **¹H NMR** (400 MHz, $CDCl_3$): δ 6.47 (dd, 1H, *J* = 16.1, 9.4 Hz, C*Ha*), 6.26 (d, 1H, *J* = 16.2 Hz, C*Hb*), 6.04 (d, 1H, *J* = 9.3 Hz, C*Hc*), 3.80 – 3.66 (m, 4H, OC*H2*), 3.62 (s, 3H, C(O)OC*H3*), 3.21 (ddd, 2H, *J* = 12.0, 6.0, 3.6, NC*H2*), 3.10 (ddd, 2H, *J* = 11.9, 6.1, 3.5, NC*H2*), 1.06 (d, 21H, *J* = 3.5 Hz, SiC*H*, C*H3*); **¹³C NMR** (101 MHz, $CDCl_3$): δ 159.1, 135.9, 127.1, 66.7, 52.7, 45.9, 18.4, 18.3, 13.3 (note: for dimethyl carbons in tri-*iso*propylsilyl group, NSi(CH**(*CH3*)**2)3, 2 peaks were found instead of 1 due to the loss of symmetry caused by the stereogenic sulfur atom); **IR** (ν_{max}, cm^{-1}) 2945, 1676, 1436, 1275, 1253, 1115, 1067, 970, 930, 883, 741, 679; **HRMS** (ESI): *m/z* calcd for $C_{17}H_{36}N_3O_3SSi^+$: 390.2241 $[M+H]^+$; found: 390.2241.

Vinyl sulfondiimidamide 6q

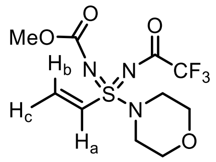

Following general procedure C, sulfondiimidamide **5q** (45.8 mg, 0.11 mmol, 1.0 equiv.), CH_2Cl_2 (2.2 mL) and peroxyacetic acid (63 µL, 0.33 mmol, 3.0 equiv.) were combined and stirred at 0 ˚C for 30 min. Purification by flash column chromatography (SiO_2, PE/EtOAc, 1:1) afforded vinyl sulfondiimidamide **6q** as a white solid (21.4 mg, 59%).

***R*f**= 0.55 (EtOAc); **m.p.** 42 – 44 ˚C (CH_2Cl_2); **^{1}H NMR** (500 MHz, $CDCl_3$): δ 6.72 (dd, 1H, *J* = 16.0, 1.3 Hz, C*Hb*), 6.56 (dd, 1H, *J* = 15.9, 9.4 Hz, C*Ha*), 6.42 (dd, 1H, *J* = 9.4, 1.3 Hz, C*Hc*), 3.78 (t, 4H, *J* = 4.8, OC*H2*), 3.72 (s, 3H, C(O)OC*H3*), 3.43 – 3.31 (m, 4H, NC*H2*); **^{13}C NMR** (126 MHz, $CDCl_3$): δ 162.3 (q, $^2J_{C-F}$ = 38.5 Hz), 156.9, 133.7, 130.9, 115.9 (q, $^1J_{C-F}$ = 288.2 Hz), 66.2, 53.9, 45.9; **^{19}F NMR** (377 MHz, $CDCl_3$) δ -75.7; **IR** (ν_{max}, cm^{-1}) 1686, 1456, 1375, 1275, 1246, 1184, 1150, 1111, 982, 935, 881, 823, 731, 642; **HRMS** (ESI): *m/z* calcd for $C_{10}H_{15}F_3N_3O_4S^+$: 330.0730 $[M+H]^+$; found: 330.0733.

Vinyl sulfondiimidamide 6r

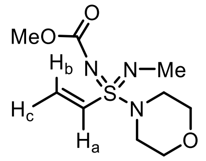

Following general procedure C, sulfondiimidamide **5r** (73.6 mg, 0.22 mmol, 1.0 equiv.), CH_2Cl_2 (4.4 mL) and peroxyacetic acid (0.13 mL, 0.66 mmol, 3.0 equiv.) were combined at 0 ˚C for 30 min. The crude residue was dissolved in acetone (1.5 mL) and water (1.5 mL), $NaHCO_3$ (37.8 mg, 0.45 mmol, 1.5 equiv.) was added and the reaction was stirred for 4 h at rt. Purification by flash column chromatography (SiO_2, PE/EtOAc, 1:1) afforded vinyl sulfondiimidamide **6r** as a colourless oil (30.4 mg, 56%).

***R*f**= 0.17 (EtOAc); **^{1}H NMR** (500 MHz, $CDCl_3$): δ 6.43 (dd, 1H, *J* = 16.6, 9.8 Hz, C*Ha*), 6.24 (dd, 1H, *J* = 16.5, 0.8 Hz, C*Hb*), 6.10 (dd, 1H, *J* = 9.8, 0.8 Hz, C*Hc*), 3.82 – 3.69 (m, 4H, OC*H2*), 3.68 (s, 3H, C(O)OC*H3*), 3.24 (ddd, 2H, *J* = 12.3, 6.4, 3.2 Hz, NC*H2*), 3.14 – 3.06 (m, 2H, NC*H2*), 2.80 (s, 3H, NC*H3*); **^{13}C NMR** (126 MHz, $CDCl_3$): δ 159.2, 132.2, 128.3, 66.7, 53.3, 46.1, 29.0; **IR** (ν_{max},

cm^{-1}) 1671, 1439, 1274, 1254, 1112, 1068, 929, 897, 788, 716; **HRMS** (ESI): *m/z* calcd for $C_9H_{18}N_3O_3S^+$: 248.1063 $[M+H]^+$; found 248.1063.

Vinyl sulfondiimidamide 6aa

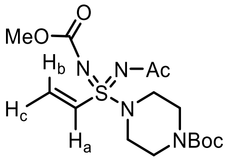

Following general procedure C, sulfondiimidamide **5aa** (112.1 mg, 0.20 mmol, 1.0 equiv.), CH_2Cl_2 (4.0 mL) and peroxyacetic acid (0.12 mL, 0.60 mmol, 3.0 equiv.) were combined at 0 ˚C for 30 min. Purification by flash column chromatography (SiO_2, PE/EtOAc, 1:3 to 2:1) afforded vinyl sulfondiimidamide **6aa** as a colourless oil (30.2 mg, 42%).

***R*f**= 0.62 (EtOAc); **^{1}H NMR** (400 MHz, $CDCl_3$): δ 6.63 – 6.57 (m, 2H, C*Ha,* C*Hb*), 6.25 (dd, 1H, *J* = 6.2, 2.2 Hz, C*Hc*), 3.67 (s, 3H, C(O)OC*H*3), 3.51 (dd, 4H, *J* = 6.4, 4.0 Hz, C*H*2), 3.38 – 3.24 (m, 4H, C*H*2), 2.13 (s, 3H, C(O)C*H*3), 1.43 (s, 9H, C(C*H*3)3); **^{13}C NMR** (101 MHz, $CDCl_3$): δ 178.3, 158.0, 154.2, 132.3, 131.3, 80.7, 53.4, 45.6, 42.7, 28.4, 27.0; **IR** ($ν_{max}$, cm^{-1}) 1692, 1422, 1365, 1280, 1241, 1168, 1126, 1044, 970, 917, 884, 826, 736; **HRMS** (ESI): *m/z* calcd for $C_{15}H_{27}N_4O_5S^+$: 375.1697 $[M+H]^+$; found 375.1689.

Vinyl sulfondiimidamide 6ab

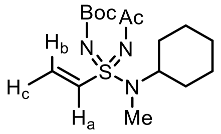

Following general procedure C, sulfondiimidamide **5ab** (32.0 mg, 0.07 mmol, 1.0 equiv.), CH_2Cl_2 (1.4 mL) and peroxyacetic acid (38 µL, 0.21 mmol, 3.0 equiv.) were combined and stirred at 0 ˚C for 30 min. Purification by flash column chromatography (SiO_2, PE/EtOAc, 2:1 to 1:1) afforded vinyl sulfondiimidamide **6ab** as a colourless oil (11.2 mg, 50%).

***R*f**= 0.59 (EtOAc); **^{1}H NMR** (400 MHz, $CDCl_3$): δ 6.74 (dd, 1H, *J* = 16.2, 9.5 Hz, C*Ha*), 6.49 (d, 1H, *J* = 16.3 Hz, C*Hb*), 6.13 (d, 1H, *J* = 9.4 Hz, C*Hc*), 3.68 (tt, 1H, *J* = 11.8, 3.5 Hz, Cy*H*), 2.86 (s, 3H, NC*H*3), 2.13 (s, 3H, C(O)C*H*3), 1.81 (d, 4H, *J* = 12.3 Hz, Cy*H*), 1.62 (t, 2H, *J* = 15.2 Hz, Cy*H*), 1.46 (s, 9H, C(C*H*3)3, 1.38 – 1.20 (m, 3H, Cy*H*), 1.07 (tt, 1H, *J* = 13.0, 3.4 Hz, Cy*H*); **^{13}C NMR** (101 MHz, $CDCl_3$): δ 178.2, 156.9, 133.8, 129.3, 80.4, 56.6, 30.6, 30.5, 29.1, 28.3, 27.2, 26.0, 26.0, 25.4

(note: for cyclohexane ring, 6 peaks were found instead of 4 due to the loss of symmetry caused by the chiral sulfur atom); **IR** (ν_{max}, cm^{-1}) 1671, 1655, 1365, 1287, 1245, 1154, 1042, 945, 897, 828; **HRMS** (ESI): *m/z* calcd for $C_{16}H_{30}N_3O_3S^+$: 344.2002 $[M+H]^+$; found 344.2016.

Vinyl sulfondiimidamide 6ac

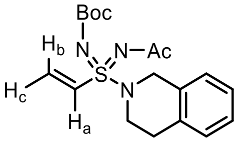

Following general procedure C, sulfondiimidamide **5ac** (24.8 mg, 0.05 mmol, 1.0 equiv.), CH_2Cl_2 (1.0 mL) and peroxyacetic acid (10 µL, 0.05 mmol, 1.0 equiv.) were combined at 0 ˚C for 30 min. Purification by flash column chromatography (SiO_2, PE/EtOAc, 3:1 to 2:1) afforded vinyl sulfondiimidamide **6ac** as a colourless oil (11.3 mg, 58%).

***R*f**= 0.78 (EtOAc); **^{1}H NMR** (500 MHz, $CDCl_3$): δ 7.21 – 7.16 (m, 2H, Ar*H*), 7.14 (q, 1H, *J* = 4.8 Hz, Ar*H*), 7.08 (q, 1H, *J* = 4.8 Hz, Ar*H*), 6.69 (dd, 1H, *J* = 16.2, 9.3 Hz, C*Ha*), 6.55 (d, 1H, *J* = 16.2 Hz, C*Hb*), 6.19 (d, 1H, *J* = 9.4 Hz, C*Hc*), 4.51 (s, 2H, NC*H*2), 3.73 – 3.57 (m, 2H, Cy*H*), 2.97 (dt, 2H, *J* = 7.6, 3.7 Hz, Cy*H*), 2.17 (s, 3H, C(O)C*H*3), 1.45 (s, 9H, C(C*H*3)3); **^{13}C NMR** (126 MHz, $CDCl_3$): δ 178.3, 156.8, 133.4, 133.1, 131.8, 130.2, 129.0, 127.1, 126.6, 126.5, 80.9, 47.5, 43.6, 29.2, 28.2, 27.1; **IR** (ν_{max}, cm^{-1}) 1669, 1655, 1366, 1284, 1248, 1154, 1032, 911, 759; **HRMS** (ESI): *m/z* calcd for $C_{18}H_{26}N_3O_3S^+$: 364.1689 $[M+H]^+$; found 364.1687.

2.2 Synthesis of alkenyl sulfondiimidamides

2.2.1 General Procedure D – Synthesis of N-H alkenyl sulfondiimidamides (7)

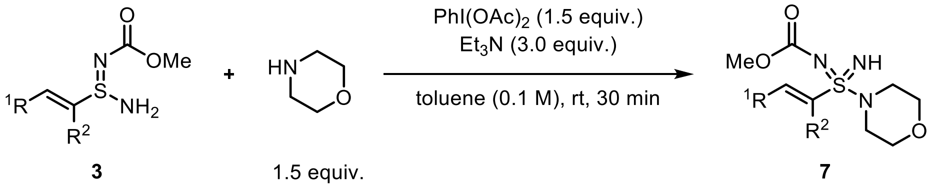

This procedure was adapted from the literature.[2] An oven-dried round-bottom flask containing primary sulfinamidine **3f-g** (1.0 equiv.) and $PhI(OAc)_2$ (1.5 equiv.) was sealed and flushed with N_2 and anhydrous THF (0.1 M) was added. Et_3N (3.0 equiv.) was added to the solution, followed immediately by morpholine (1.5 equiv.). The reaction mixture was stirred

at rt for 30 min then diluted with EtOAc (10 mL) and quenched with sat. aq. NaCl solution (10 mL). The aqueous phase was separated and extracted with EtOAc (3 × 10 mL). The combined organic layers were dried over anhydrous Na_2SO_4, filtered and concentrated *in vacuo*. The *N*-H sulfondiimidamide was purified by flash column chromatography with the appropriate solvent system.

α-methyl alkenyl sulfondiimidamide 7a

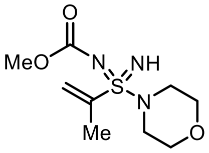

Following general procedure D, sulfinamidine **3f** (81.1 mg, 0.50 mmol, 1.0 equiv.), $PhI(OAc)_2$ (241.5 mg, 0.75 mmol, 1.5 equiv.), toluene (5.0 mL), Et_3N (0.21 mL, 1.5 mmol, 3.0 equiv.) and morpholine (67 µL, 0.75 mmol, 1.5 equiv.) were combined and stirred at rt for 30 min. Purification by flash column chromatography (SiO_2, EtOAc) afforded sulfondiimidamide **7a** as a colourless oil (98.0 mg, 79%).

***R*$_f$**= 0.20 (EtOAc); **^{1}H NMR** (400 MHz, $CDCl_3$): δ 6.19 (s, 1H, C*H*), 5.74 (s, 1H, C*H*), 3.66 (t, 4H, *J* = 4.7 Hz, OC*H$_2$*), 3.62 (s, 3H, C(O)OC*H*$_3$), 3.17 (t, 4H, *J* = 4.7 Hz, NC*H$_2$*), 2.10 (s, 3H, CC*H*$_3$); **^{13}C NMR** (101 MHz, $CDCl_3$): δ 160.1, 141.4, 124.7, 66.6, 52.8, 45.8, 16.9; **IR** (ν_{max}, cm^{-1}) 1656, 1438, 1390, 1276, 1256, 1113, 993, 930, 861, 788, 736, 704, 689; **HRMS** (ESI): *m/z* calcd for $C_9H_{18}N_3O_3S^+$: 248.1063 $[M+H]^+$; found 248.1057.

β-methyl alkenyl sulfondiimidamide 7b

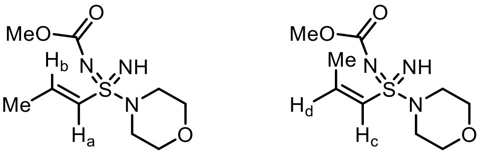

Following general procedure D, sulfinamidine **3g** (162.2 mg, 1.00 mmol, 1.0 equiv.), $PhI(OAc)_2$ (483 mg, 1.50 mmol, 1.5 equiv.), toluene (10.0 mL), Et_3N (0.42 mL, 3.0 mmol, 3.0 equiv.) and morpholine (0.13 mL, 1.5 mmol, 1.5 equiv.) were combined and stirred at rt for 30 min. Purification by flash column chromatography (SiO_2, EtOAc) afforded sulfondiimidamide **7b** as a mixture of isomers (*E*:*Z* = 2.5:1) as a white solid (129 mg, 52%).

***R*$_f$**= 0.40 (EtOAc); **m.p.** 78 – 80 ˚C (CH_2Cl_2); **^{1}H NMR** (400 MHz, $CDCl_3$): *Trans isomer:* δ 6.93 (dq, 1H, *J* = 14.8, 7.0 Hz, C*H$_b$*), 6.02 (dq, 1H, *J* = 14.8, 1.8 Hz, C*H$_a$*), 3.72 – 3.62 (m, 4H, OC*H*$_2$), 3.60 (s, 3H, C(O)OC*H*$_3$), 3.16 – 3.03 (m, 4H, NC*H*$_2$), 1.92 (dd, 3H, *J* = 7.0, 1.7 Hz, CHC*H*$_3$); *Cis isomer:* δ 6.44 (dq, 1H, *J* = 11.0, 7.4 Hz, C*H$_d$*), 6.02 (dq, 1H, *J* = 14.8, 1.8 Hz, C*H$_c$*), 3.72 – 3.62 (m, 4H, OC*H*$_2$), 3.60 (s, 3H, C(O)OC*H*$_3$), 3.23 – 3.16 (m, 4H, NC*H*$_2$), 2.16 (dd, 3H, *J* = 7.5, 1.8 Hz, CHC*H*$_3$); **^{13}C NMR** (101 MHz, $CDCl_3$): *Trans isomer*: δ 159.6, 143.8, 123.7, 66.4, 52.7, 46.2, 17.2; *Cis isomer*: δ 159.9, 143.6, 125.3, 66.4, 52.7, 46.0, 14.7; **IR** (ν_{max}, cm^{-1}) 1669, 1438, 1254, 1111, 1065, 918, 863, 788, 697; **HRMS** (ESI): *m/z* calcd for $C_9H_{18}N_3O_3S^+$: 248.1063 $[M+H]^+$; found: 248.1061.

2.2.2 *N*-functionalisation of alkenyl sulfondiimidamides

α-methyl alkenyl sulfondiimidamide 8a

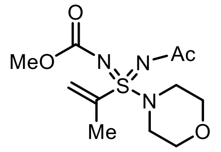

N-H sulfondiimidamide **7a** (39.6 mg, 0.16 mmol, 1.0 equiv.), was dissolved in THF (1.6 mL) in an oven-dried round-bottom flask and the solution was cooled to 0 ˚C. NaH (7 mg, 0.17 mmol, 1.1 equiv.) was added and the solution was stirred for 10 minutes at 0 ˚C before the addition of acetyl chloride (12 µL, 0.17 mmol, 1.1 equiv.). The reaction mixture was stirred at 0 ˚C for 5 min before being warmed to rt and stirred for 2 h. This was then diluted with EtOAc (10 mL) and quenched with sat. aq. NaCl solution (10 mL). The aqueous phase was separated and extracted with EtOAc (3 × 10 mL). The combined organic layers were dried over anhydrous Na_2SO_4, filtered and concentrated *in vacuo*. Purification by flash column chromatography (SiO_2, EtOAc) afforded sulfondiimidamide **8a** as a white solid (32.7 mg, 71%).

***R*$_f$**= 0.30 (EtOAc); **m.p.** 98 – 100 ˚C (CH_2Cl_2); **^{1}H NMR** (400 MHz, $CDCl_3$): δ 6.48 (s, 1H, C*H*), 5.99 (s, 1H, C*H*), 3.77 – 3.70 (m, 4H, OC*H*$_2$), 3.68 (s, 3H, C(O)OC*H*$_3$), 3.37 – 3.30 (m, 4H, NC*H*$_2$), 2.14 (s, 3H, C(O)C*H*$_3$), 2.11 (s, 3H, CC*H*$_3$); **^{13}C NMR** (101 MHz, $CDCl_3$): δ 178.2, 157.9, 140.0, 128.5, 66.6, 53.4, 45.6, 27.0, 16.0; **IR** (ν_{max}, cm^{-1}) 1683, 1654, 1437, 1363, 1278, 1239, 1113, 1068, 1043, 970, 924, 886, 785, 736; **HRMS** (ESI): *m/z* calcd for $C_{11}H_{20}N_3O_4S^+$: 290.1169 $[M+H]^+$; found 290.1159.

α-methyl alkenyl sulfondiimidamide 8b

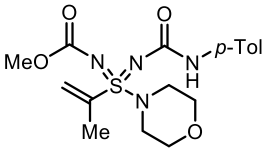

N-H sulfondiimidamide **7a** (106.3 mg, 0.43 mmol, 1.0 equiv.), was dissolved in MeCN (4.0 mL). DBU (77 µL, 0.51 mmol, 1.2 equiv.) was added to the stirred solution, followed by *p*-tolyl isocyanate (64 µL, 0.51 mmol, 1.2 equiv.). The reaction mixture was stirred at rt for 30 min then diluted with EtOAc (10 mL) and quenched with sat. aq. NaCl solution (10 mL). The aqueous phase was separated and extracted with EtOAc (3 × 10 mL). The combined organic layers were dried over anhydrous Na_2SO_4, filtered and concentrated *in vacuo*. Purification by flash column chromatography (SiO_2, PE/EtOAc, 1:1) afforded sulfondiimidamide **8b** as a white solid (71.5 mg, 47%).

***R*$_f$**= 0.48 (EtOAc); **m.p.** 136 – 138 ˚C (CH_2Cl_2); **^{1}H NMR** (400 MHz, $CDCl_3$): δ 7.30 (d, 2H, *J* = 8.0 Hz, Ar*H*), 7.11 (s, 1H, N*H*), 7.05 (d, 2H, *J* = 8.2 Hz, Ar*H*), 6.46 (s, 1H, C*H*), 5.98 (s, 1H, C*H*), 3.74 (t, 4H, *J* = 4.7 Hz, OCH_2), 3.67 (s, 3H, C(O)OCH_3), 3.39 (ddt, 4H, *J* = 33.0, 12.4, 4.7 Hz, NCH_2), 2.27 (s, 3H, ArCH_3), 2.13 (s, 3H, CCH_3); **^{13}C NMR** (101 MHz, $CDCl_3$): δ 158.2, 155.3, 140.5, 136.4, 132.6, 129.4, 128.1, 118.8, 66.5, 53.3, 45.7, 20.8, 16.2; **IR** (ν_{max}, cm^{-1}) 1675, 1655, 1524, 1438, 1254, 1225, 1112, 974, 872, 821; **HRMS** (ESI): *m/z* calcd for $C_{17}H_{25}N_4O_4S^+$: 381.1591 $[M+H]^+$; found 381.1585.

α-methyl alkenyl sulfondiimidamide 8c

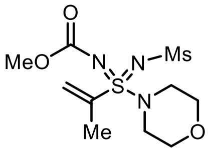

N-H sulfondiimidamide **7a** (98.9 mg, 0.40 mmol, 1.0 equiv.), was dissolved in CH_2Cl_2 (2.0 mL). Et_3N (84 µL, 0.60 mmol, 1.5 equiv.) was added to the stirred solution, followed by, methanesulfonyl chloride (37 µL, 0.48 mmol, 1.2 equiv.) and DMAP (10 mg, 0.08 mmol, 0.2 equiv.). The reaction mixture was stirred at rt for 1 h then diluted with CH_2Cl_2 (10 mL) and quenched with sat. aq. NaCl solution (10 mL). The aqueous phase was separated and extracted with CH_2Cl_2 (3 × 10 mL). The combined organic layers were dried over anhydrous

Na_2SO_4, filtered and concentrated *in vacuo*. Purification by flash column chromatography (SiO_2, PE/EtOAc, 1:2) afforded sulfondiimidamide **8c** as a colourless oil (47.0 mg, 36%).

***R*$_f$**= 0.36 (EtOAc); **^{1}H NMR** (500 MHz, $CDCl_3$): δ 6.48 (s, 1H, C*H*), 6.06 (s, 1H, C*H*), 3.76 (dt, 4H, *J* = 6.0, 3.1 Hz, OC*H*$_2$), 3.73 (s, 3H, C(O)OC*H*$_3$), 3.50 – 3.29 (m, 4H, NC*H*$_2$), 3.11 (s, 3H, SO_2C*H*$_3$), 2.15 (s, 3H, CC*H*$_3$); **^{13}C NMR** (126 MHz, $CDCl_3$): δ 157.7, 140.6, 129.5, 66.4, 53.8, 45.7, 43.7, 16.2; **IR** (ν_{max}, cm^{-1}) 1683, 1438, 1310, 1255, 1142, 1109, 1078, 968, 938, 926, 794, 698, 645; **HRMS** (ESI): *m/z* calcd for $C_{10}H_{20}N_3O_5S_2^+$: 326.0839 [M+H]$^+$; found 326.0832.

α-methyl alkenyl sulfondiimidamide 8d

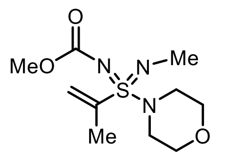

N-H sulfondiimidamide **7a** (24.7 mg, 0.10 mmol, 1.0 equiv.) was dissolved in THF (1.0 mL) and cooled to 0 ˚C. NaH (4.4 mg, 0.11 mmol, 1.1 equiv.) was added and the solution was stirred for 10 minutes at 0 ˚C before the addition of methyl iodide (7 µL, 0.1 mmol, 1.0 equiv.). The reaction mixture was stirred at 0 ˚C for 5 min before being warmed to rt and stirred for 18 h. This was then diluted with EtOAc (10 mL) and quenched with sat. aq. NaCl solution (10 mL). The aqueous phase was separated and extracted with EtOAc (3 × 10 mL). The combined organic layers were dried over anhydrous Na_2SO_4, filtered and concentrated *in vacuo*. Purification by flash column chromatography (SiO_2, EtOAc) afforded sulfondiimidamide **8d** as a white solid (15.2 mg, 57%).

***R*$_f$**= 0.30 (EtOAc); **^{1}H NMR** (400 MHz, $CDCl_3$): δ 6.02 (s, 1H, C*H*), 5.77 (s, 1H, C*H*), 3.78 – 3.68 (m, 4H, OC*H*$_2$), 3.66 (s, 3H, C(O)OC*H*$_3$), 3.26 (ddd, 2H, *J* = 12.4, 6.1, 3.3 Hz, NC*H*$_2$), 3.15 (ddd, 2H, *J* = 12.1, 6.2, 3.2 Hz, NC*H*$_2$), 2.77 (s, 3H, NC*H*$_3$), 2.11 (s, 3H, CC*H*$_3$); **^{13}C NMR** (101 MHz, $CDCl_3$): δ 159.2, 141.8, 124.0, 66.8, 52.9, 46.0, 29.3, 18.4; **IR** (ν_{max}, cm^{-1}) 1679, 1437, 1273, 1254, 1113, 1066, 969, 933, 903, 737, 648; **HRMS** (ESI): *m/z* calcd for $C_{10}H_{20}N_3O_3S^+$: 262.1220 [M+H]$^+$; found 262.1211.

α-methyl alkenyl sulfondiimidamide 8e

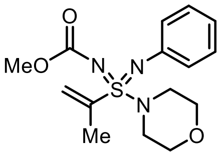

N-H sulfondiimidamide **7a** (123.7 mg, 0.50 mmol, 1.0 equiv.), phenylboronic acid (152.4 mg, 1.25 mmol, 2.5 equiv.) and $Cu(CH_3CN)_4PF_6$ (93.2 mg, 0.25 mmol, 0.5 equiv.) were added to an oven-dried round-bottom flask under an O_2 atmosphere and dissolved in MeCN (5.0 mL). *N*-methyl piperidine was then added to the stirred solution and the reaction was stirred under an O_2 atmosphere at rt for 4 h. This was then diluted with EtOAc (15 mL) and quenched with sat. aq. NaCl solution (15 mL). The aqueous phase was separated and extracted with EtOAc (3 × 15 mL). The combined organic layers were dried over anhydrous Na_2SO_4, filtered and concentrated *in vacuo*. Purification by flash column chromatography (SiO_2, PE/EtOAc, 2:1 to 1:1) afforded sulfondiimidamide **8e** as a colourless oil (65.7 mg, 41%).

***R*$_f$**= 0.73 (EtOAc); **^{1}H NMR** (400 MHz, $CDCl_3$): δ 7.25 – 7.12 (m, 4H, Ar*H*), 6.97 (tt, 1H, *J* = 7.1, 1.5 Hz, Ar*H*), 6.24 (s, 1H, C*H*), 5.90 (s, 1H, C*H*), 3.77 – 3.57 (m, 7H, OCH_2, C(O)OCH_3), 3.37 (ddd, 2H, *J* = 12.4, 6.3, 3.2 Hz, NCH_2), 3.26 (ddd, 2H, *J* = 12.1, 6.3, 3.1 Hz, NCH_2), 2.19 (s, 3H, CCH_3); **^{13}C NMR** (101 MHz, $CDCl_3$): δ 158.8, 142.7, 142.1, 129.1, 125.2, 123.9, 122.6, 66.7, 53.1, 46.1, 18.2; **IR** (v_{max}, cm^{-1}) 1677, 1488, 1437, 1269, 1249, 1112, 1059, 925, 874, 846, 759, 696; **HRMS** (ESI): *m/z* calcd for $C_{15}H_{22}N_3O_3S^+$: 324.1376 [M+H]$^+$; found 324.1377.

β-methyl alkenyl sulfondiimidamide 8f

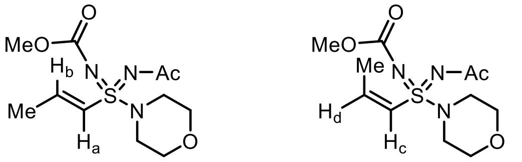

N-H sulfondiimidamide **7b** (24.7 mg, 0.10 mmol, 1.0 equiv.), was dissolved in THF (1.0 mL) and the solution was cooled to 0 ˚C. NaH (4.4 mg, 0.17 mmol, 1.1 equiv.) was added and the solution was stirred for 10 minutes at 0 ˚C before the addition of acetyl chloride (8 µL, 0.11 mmol, 1.1 equiv.). The reaction mixture was stirred at 0 ˚C for 10 min before being warmed to rt and stirred for 2 h. This was then diluted with EtOAc (10 mL) and quenched with sat. aq. NaCl solution (10 mL). The aqueous phase was separated and extracted with EtOAc

(3 × 10 mL). The combined organic layers were dried over anhydrous Na_2SO_4, filtered and concentrated *in vacuo*. Purification by flash column chromatography (SiO_2, EtOAc) afforded sulfondiimidamide **8f** as a mixture of isomers (*E*:*Z* = 10:1) as a colourless oil (11.8 mg, 41%).

***R*f**= 0.30 (EtOAc); **^{1}H NMR** (400 MHz, $CDCl_3$) *Trans isomer:* δ 7.14 (dq, 1H, *J* = 14.2, 7.1 Hz, C*H$_b$*), 6.30 (dq, 1H, *J* = 14.7, 1.7 Hz, C*H$_a$*), 3.75 (t, 4H, *J* = 4.8 Hz, OC*H$_2$*), 3.68 (s, 3H, C(O)OC*H$_3$*), 3.30 (dd, 4H, *J* = 6.5, 3.6 Hz, NC*H$_2$*), 2.13 (s, 3H, C(O)C*H$_3$*), 2.01 (dd, 3H, *J* = 7.1, 1.7 Hz, CC*H$_3$*); *Cis isomer:* δ 6.55 (dq, 1H, *J* = 10.8, 7.5 Hz, C*H$_d$*), 6.30 (dq, 1H, *J* = 14.7, 1.7 Hz, C*H$_c$*), 3.75 (t, 4H, *J* = 4.8 Hz, OC*H$_2$*), 3.68 (s, 3H, C(O)OC*H$_3$*), 3.43 (q, 4H, *J* = 4.1 Hz, NC*H$_2$*), 2.17 (dd, 3H, *J* = 7.5, 1.8 Hz, CC*H$_3$*), 2.13 (s, 3H, C(O)C*H$_3$*); **^{13}C NMR** (101 MHz, $CDCl_3$) δ 178.3, 158.1, 147.2, 124.0, 66.5, 53.4, 45.9, 27.1, 17.8; **IR** (ν_{max}, cm^{-1}) 1680, 1650, 1437, 1362, 1271, 1237, 1112, 1067, 1040, 922, 883, 835, 711; **HRMS** (ESI): *m/z* calcd for $C_{11}H_{20}N_3O_4S^+$: 290.1169 $[M+H]^+$; found: 290.1155.

β-methyl alkenyl sulfondiimidamide 8g

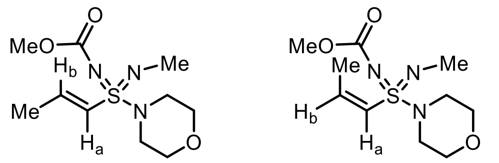

N-H sulfondiimidamide **7b** (47.5 mg, 0.19 mmol, 1.0 equiv.) was dissolved in THF (1.9 mL) and cooled to 0 ˚C. NaH (9.2 mg, 0.23 mmol, 1.2 equiv.) was added and the solution was stirred for 10 minutes at 0 ˚C before the addition of methyl iodide (35 µL, 0.57 mmol, 3.0 equiv.). The reaction mixture was stirred at 0 ˚C for 5 min before being warmed to rt and stirred for 18 h. This was then diluted with EtOAc (10 mL) and quenched with sat. aq. NaCl solution (10 mL). The aqueous phase was separated and extracted with EtOAc (3 × 10 mL). The combined organic layers were dried over anhydrous Na_2SO_4, filtered and concentrated *in vacuo*. Purification by flash column chromatography (SiO_2, EtOAc) afforded sulfondiimidamide **8g** as a mixture of isomers (*E*:*Z* = 7:1) as a colourless oil (27.9 mg, 56%).

***R*f**= 0.18 (EtOAc); **^{1}H NMR** (600 MHz, $CDCl_3$) *Trans isomer:* δ 6.77 (1H, dq, *J* = 14.1, 6.9 Hz, C*H$_b$*), 6.12 (1H, dq, *J* = 15.1, 1.7 Hz, C*H$_a$*), 3.83 – 3.63 (7H, m, OC*H$_2$*, C(O)OC*H$_3$*), 3.21 (2H, ddd, *J* = 12.2, 6.4, 3.1 Hz, NC*H$_2$*), 3.07 (2H, ddd, *J* = 12.0, 6.4, 3.0 Hz, NC*H$_2$*), 2.78 (3H, s, NC*H$_3$*), 1.97 (3H, dd, *J* = 7.0, 1.7 Hz, CHC*H$_3$*); **^{13}C NMR** (151 MHz, $CDCl_3$) δ 159.1, 143.2, 124.6, 66.7, 52.9,

46.0, 29.1, 17.5; **IR** (ν_{max}, cm^{-1}) 1674, 1437, 1267, 1252, 1111, 1065, 967, 925, 901, 841, 788, 695; **HRMS** (ESI): *m/z* calcd for $C_{10}H_{20}N_3O_3S^+$: 262.1220 $[M+H]^+$; found: 262.1227.

2.3 Synthesis of vinyl sulfondiimidamides from β-amino sulfinamidine

Β-amino sulfinamidine 9

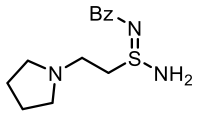

Sulfinamidine **3c** (194.3 mg, 1.00 mmol, 1.0 equiv.) was dissolved in MeCN (5.0 mL) in an oven-dried round bottom-flask and cooled to 0 ˚C. Pyrrolidine (0.10 mL, 1.20 mmol, 1.2 equiv.) was added followed immediately by DBU (0.18 mL, 1.20 mmol, 1.2 equiv.). The reaction was stirred at 0 ˚C for 2 h and then diluted with EtOAc (20 mL) and quenched with sat. aq. NaCl solution (20 mL). The aqueous phase was separated and extracted with EtOAc (3 × 20 mL). The combined organic layers were dried over anhydrous Na_2SO_4, filtered and concentrated *in vacuo*. Purification by flash column chromatography (SiO_2, EtOAc/MeOH, 1:0 to 10:1) afforded sulfondiimidamide **9** as an off-white solid (132 mg, 50%).

***R*$_f$**= 0.12 (EtOAc/MeOH 10:1); **m.p.** 94 – 96 ˚C (EtOAc); **^{1}H NMR** (500 MHz, $CDCl_3$): δ 8.10 – 8.04 (m, 2H, Ar*H*), 7.46 – 7.39 (m, 1H, Ar*H*), 7.38 – 7.31 (m, 2H, Ar*H*), 5.16 (s, 2H, N*H*$_2$), 3.56 – 3.47 (m, 1H, C*H*), 3.29 (ddd, 1H, *J* = 13.8, 8.9, 5.0 Hz, C*H*), 3.15 (dt, 1H, *J* = 13.6, 5.2 Hz, C*H*), 2.79 (dt, 1H, *J* = 12.9, 5.2 Hz, C*H*), 2.64 – 2.48 (m, 4H, NC*H*$_2$), 1.74 (td, 4H, *J* = 5.5, 2.7 Hz, alkylC*H*$_2$); **^{13}C NMR** (126 MHz, $CDCl_3$): δ 178.1, 136.8, 131.0, 128.7, 127.9, 54.0, 49.5, 45.7, 23.6; **IR** (ν_{max}, cm^{-1}) 1592, 1540, 1336, 1297, 1172, 1136, 1025, 716; **HRMS** (ESI): *m/z* calcd for $C_{13}H_{20}N_3OS^+$: 266.1322 $[M+H]^+$; found 266.1324.

Β-amino sulfondiimidamide 11

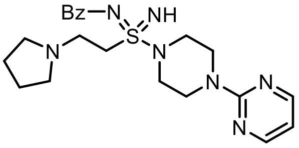

An oven-dried round-bottom flask containing β-amino sulfinamidine **9** (39.8 mg, 0.15 mmol, 1.0 equiv.) and $PhI(OAc)_2$ (72.5 mg, 0.225 mmol, 1.5 equiv.) was sealed and flushed with N_2 and anhydrous toluene (1.5 mL) was added. Et_3N (62 µL, 0.45 mmol, 3.0 equiv.) was added to

the solution, followed immediately by the addition of 1-(2-Pyrimidyl)piperazine **10** (36.9 mg, 0.225, 1.5 equiv.). The reaction mixture was stirred at rt for 30 min then diluted with EtOAc (10 mL) and quenched with sat. aq. NaCl solution (10 mL). The aqueous phase was separated and extracted with EtOAc (3 × 10 mL). The combined organic layers were dried over anhydrous Na_2SO_4, filtered and concentrated *in vacuo*. Purification by flash column chromatography (SiO_2, EtOAc/MeOH, 10:1) afforded **11** as a colourless oil (52.2 mg, 81%).

***R*$_f$**= 0.17 (EtOAc/MeOH 10:1); **^{1}H NMR** (400 MHz, $CDCl_3$): δ 8.30 (t, 2H, *J* = 5.0 Hz, Ar*H*), 8.11 (dd, 2H, *J* = 17.5, 7.8 Hz, Ar*H*), 7.57 – 7.15 (m, 3H, Ar*H*), 6.50 (q, 1H, *J* = 6.2 Hz, Ar*H*), 4.10 – 3.64 (m, 5H, NC*H*$_2$, C*H*), 3.41 (p, 5H, *J* = 11.5 Hz, NC*H*$_2$, C*H*), 3.11 (ddp, 2H, *J* = 27.3, 11.2, 5.3 Hz, C*H*$_2$), 2.59 (d, 4H, *J* = 6.2 Hz, NC*H*$_2$), 1.91 – 1.69 (m, 4H, alkylC*H*$_2$); **^{13}C NMR** (126 MHz, $CDCl_3$): δ 174.8, 161.4, 157.8, 136.0, 131.9, 129.1, 128.1, 110.6, 54.2, 51.4, 49.3, 46.3, 43.9, 23.6; **IR** (ν_{max}, cm^{-1}) 1585, 1550, 1499, 1448, 1357, 1317, 1293, 1262, 1139, 1025, 983, 953, 903, 799, 716; **HRMS** (ESI): *m/z* calcd for $C_{21}H_{30}N_7OS^+$: 428.2227 $[M+H]^+$; found 428.2234.

B-amino sulfondiimidamide 12

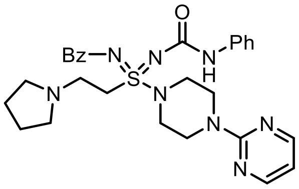

N-H sulfondiimidamide **11** (42.8 mg, 0.10 mmol, 1.0 equiv.), was dissolved in MeCN (1.0 mL). DBU (22 µL, 0.15 mmol, 1.5 equiv.) was added to the stirred solution, followed by *p*-tolyl isocyanate (16 µL, 0.15 mmol, 1.5 equiv.). The reaction mixture was stirred at rt for 30 min then diluted with EtOAc (10 mL) and quenched with sat. aq. NaCl solution (10 mL). The aqueous phase was separated and extracted with EtOAc (3 × 10 mL). The combined organic layers were dried over anhydrous Na_2SO_4, filtered and concentrated *in vacuo*. Purification by flash column chromatography (SiO_2, EtOAc/MeOH, 20:1) afforded sulfondiimidamide **12** as a white solid (18.5 mg, 34%).

***R*$_f$**= 0.30 (EtOAc/MeOH 10:1); **^{1}H NMR** (400 MHz, DMSO-*d*^{6}): δ 9.47 (s, 1H, N*H*), 8.40 (d, 2H, d, *J* = 4.8 Hz, Ar*H*), 8.05 – 7.98 (m, 2H, Ar*H*), 7.59 – 7.52 (m, 1H, Ar*H*), 7.47 (dt, 4H, *J* = 15.0, 7.8 Hz, Ar*H*), 7.19 (t, 2H, *J* = 7.8 Hz, Ar*H*), 6.90 (t, 1H, *J* = 7.3 Hz, Ar*H*), 6.69 (t, 1H, *J* = 4.7 Hz, Ar*H*), 4.12 (t, 2H, *J* = 7.0 Hz, SC*H*$_2$), 3.91 (t, 4H, *J* = 5.1 Hz, NC*H*$_2$), 3.52 (q, 4H, *J* = 4.6 Hz, NC*H*$_2$), 2.88 (td,

2H, J = 7.3, 2.5 Hz, SCH_2CH_2), 2.46 (d, 4H, J = 5.6 Hz, NCH_2), 1.60 (q, 4H, J = 3.3 Hz, alkylCH_2); **^{13}C NMR** (101 MHz, $CDCl_3$): δ 171.2, 161.0, 158.1, 155.8, 140.3, 135.9, 132.1, 128.9, 128.4, 128.2, 121.8, 118.2, 110.7, 53.4, 49.1, 48.3, 45.3, 43.3, 23.2; **IR** (ν_{max}, cm^{-1}) 1585, 1550, 1499, 1448, 1357, 1317, 1293, 1262, 1139, 1025, 983, 953, 903, 799, 716; **HRMS** (ESI): *m/z* calcd for $C_{28}H_{35}N_8O_2S^+$: 547.2598 [M+H]$^+$; found 547.2589.

Vinyl sulfondiimidamide 13

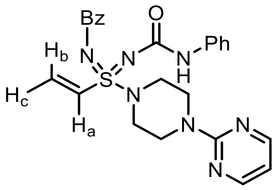

Sulfondiimidamide **12** (71.1 mg, 0.13 mmol, 1.0 equiv.) was dissolved in CH_2Cl_2 (2.6 mL) under a N_2 atmosphere and cooled to 0 ˚C. Peroxyacetic acid (25 µL, 0.13 mmol, 1.0 equiv.) was then added and the mixture stirred at 0 ˚C for 30 minutes. After this time the reaction was quenched with $Na_2S_2O_4$ (10 mL) and diluted with EtOAc (10 mL). The aqueous phase was separated and extracted with EtOAc (3 × 10 mL). The combined organic layers were dried over anhydrous Na_2SO_4, filtered and concentrated *in vacuo*. Purification by flash column chromatography (SiO_2, PE/EtOAc, 1:1) afforded vinyl sulfondiimidamide **13** as a white solid (29.2 mg, 47%).

***R*$_f$**= 0.83 (EtOAc); **^{1}H NMR** (500 MHz, $CDCl_3$): δ 8.31 (d, 2H, J = 4.7 Hz, Ar*H*), 8.19 – 8.12 (m, 2H, Ar*H*), 7.54 – 7.46 (m, 1H, Ar*H*), 7.40 (td, 4H, J = 6.7, 3.3 Hz, Ar*H*), 7.29 – 7.20 (m, 2H, Ar*H*), 7.04 – 6.91 (m, 2H, Ar*H*, CH_a), 6.74 (d, 1H, J = 16.2 Hz, CH_b), 6.53 (t, 1H, J = 4.8 Hz, Ar*H*), 6.33 (d, 1H, J = 9.4 Hz, CH_c), 4.01 (t, 4H, J = 5.2 Hz, NCH_2), 3.60 – 3.46 (m, 4H, $SNCH_2$); **^{13}C NMR** (126 MHz, $CDCl_3$): δ 172.7, 161.3, 157.9, 155.7, 138.9, 135.7, 133.2, 132.4, 130.6, 129.7, 129.0, 128.2, 123.3, 118.9, 110.7, 45.9, 43.4; **IR** (ν_{max}, cm^{-1}) 1634, 1584, 1551, 1500, 1440, 1358, 1312, 1286, 1263, 1222, 1122, 951, 917, 798, 756, 713; **HRMS** (ESI): *m/z* calcd for $C_{24}H_{26}N_7O_2S^+$: 476.1863 [M+H]$^+$; found 476.1873.

2.4 General procedure E – conjugate addition reactions with amino acid derivatives

Procedure adapted from the literature.[3] The respective sulfondiimidamide **6** (1.0 equiv.) was dissolved in anhydrous MeCN (0.1 M) in an oven dried microwave vial. Anhydrous triethylamine (2.0 equiv.) was added, followed by the amino acid (1.0 equiv.). The reaction mixture was stirred at rt for 18 h and then concentrated *in vacuo*. Purification by flash column chromatography with the appropriate solvent system afforded the desired product.

Sulfondiimidamide-cysteine adduct S1a

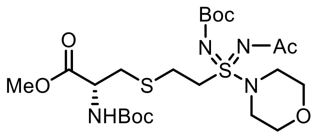

Prepared according to general procedure E, using sulfondiimidamide **6i** (17.9 mg, 0.057 mmol, 1.0 equiv.), triethylamine (16 µL, 0.113 mmol, 2.0 equiv.), Boc-Cys-OMe (12 µL, 0.057 mmol, 1.0 equiv.) and MeCN (0.57 mL). Purification by flash column chromatography (SiO_2, petrol/EtOAc, 1:1) afforded **S1a** as a mixture of diastereomers as a colourless oil (21.6 mg, 70%).

R*$_f$**= 0.43 (Petrol/EtOAc 1:3); **^{1}H NMR** (400 MHz, $CDCl_3$): δ 5.30 (s, 1H, N*H*), 4.51 (s, 1H, C*H*), 3.90 – 3.70 (m, 9H, OC*H*$_2$, C(O)OC*H*$_3$, alkylC*H*$_2$), 3.37 (dd, 4H, *J* = 5.7, 3.8 Hz, NC*H*$_2$), 3.06 – 2.79 (m, 4H, alkyl*H*), 2.13 (d, 3H, *J* = 2.2 Hz, C(O)C*H*$_3$), 1.46 (s, 9H, C(C*H*$_3$)$_3$), 1.44 (s, 9H, C(C*H*$_3$)$_3$); **^{13}C NMR** (101 MHz, $CDCl_3$): δ 178.5, 171.3, 156.7, 155.3, 81.0, 80.6, 66.6, 53.4, 52.9, 51.4, 46.5, 35.30, 35.27, 28.4, 28.2, 27.1, 25.3 (note: for cysteine CH_2SC***H_2CH, 2 peaks were seen instead of 1 due to the loss of symmetry caused by the stereogenic sulfur centre); **IR** (ν_{max}, cm^{-1}) 2929, 1746, 1710, 1667, 1517, 1456, 1392, 1366, 1279, 1246, 1162, 1113, 1045, 925, 864, 786; **HRMS** (ESI): *m/z* calcd for $C_{22}H_{41}N_4O_8S_2^+$: 553.2360 [M+H]$^+$; found 553.2384.

Note: The product was isolated as a mixture of diastereomers where the dr was not determined due to the overlap of signals.

Sulfondiimidamide-lysine adduct S1b

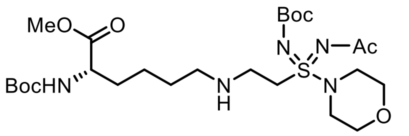

Prepared according to general procedure E, using sulfondiimidamide **6i** (15.9 mg, 0.05 mmol, 1.0 equiv.), triethylamine (14 µL, 0.10 mmol, 2.0 equiv.), Boc-Lys-OMe.HCl (14.8 mg, 0.05 mmol, 1.0 equiv.) and MeCN (0.5 mL). Purification by flash column chromatography (SiO_2, EtOAc/MeOH 1:0 to 10:1) afforded **S1b** a mixture of diastereomers as a colourless oil (20.4 mg, 72%).

***R*f**= 0.20 (EtOAc/MeOH 10:1); **^{1}H NMR** (400 MHz, $CDCl_3$): δ 5.12 (d, 1H, *J* = 8.5 Hz, N*H*), 4.34 – 4.19 (m, 1H, C*H*), 3.88 (dtd, 1H, *J* = 12.7, 6.5, 3.8 Hz, alkyl*H*), 3.81 – 3.65 (m, 8H, OCH_2, C(O)OCH_3, alkyl*H*), 3.42 – 3.35 (m, 4H, NCH_2), 3.30 (t, 1H, *J* = 4.7 Hz, alkyl*H*), 3.13 (h, 2H, *J* = 6.5 Hz, alkyl*H*), 2.72 (br.s, 1H, N*H*), 2.64 (dd, 2H, *J* = 7.6, 6.1 Hz, alkyl*H*), 2.12 (s, 3H, C(O)CH_3), 1.77 (dt, 1H, *J* = 10.9, 5.4 Hz, alkyl*H*), 1.69 – 1.57 (m, 2H, alkyl*H*), 1.56 – 1.48 (m, 2H, alkyl*H*), 1.45 (s, 9H, C(CH_3)$_3$), 1.43 (d, 9H, *J* = 1.5 Hz, C(CH_3)$_3$); **^{13}C NMR** (151 MHz, $CDCl_3$): δ 173.0, 170.9, 159.3, 155.4, 80.1, 79.9, 66.8, 53.1, 52.4, 50.0, 48.8, 46.6, 41.3, 41.3, 32.7, 29.7, 28.32, 28.26, 28.0, 22.6, 21.4 (note: 2 additional peaks found from diastereomers); **IR** (ν_{max}, cm^{-1}) 2928, 2860, 1743, 1709, 1647, 1522, 1456, 1392, 1366, 1285, 1252, 1162, 1113, 1066, 924, 862, 741; **HRMS** (ESI): *m/z* calcd for $C_{25}H_{48}N_5O_8S^+$: 578.3218 $[M+H]^+$; found 578.3211.

Note: The product was isolated as a mixture of diastereomers where the dr was not determined due to the overlap of signals.

Sulfondiimidamide-histidine adduct S1c

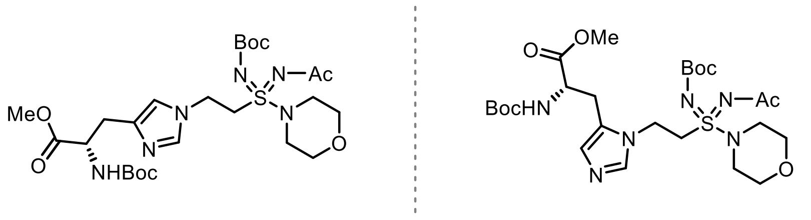

Prepared according to general procedure E, using sulfondiimidamide **6i** (15.9 mg, 0.05 mmol, 1.0 equiv.), triethylamine (14 µL, 0.10 mmol, 2.0 equiv.), Boc-His-OMe (13.5 mg, 0.05 mmol,

1.0 equiv.) and MeCN (0.5 mL). Purification by flash column chromatography (SiO_2, EtOAc/MeOH 10:1) afforded **S1c** as a mixture of regioisomers (1.3:1) and diastereomers (14.4 mg, 49%).

***R*f**= 0.14 (EtOAc); **^{1}H NMR** (600 MHz, $CDCl_3$): δ 7.51 (d, 1H, *J* = 4.0 Hz, Ar*H*), 7.42 (d, 1H, *J* = 1.4 Hz, Ar*H*), 6.80 (s, 1H, Ar*H*), 6.71 (s, 1H, Ar*H*), 5.79 (d, 1H, *J* = 8.2 Hz, N*H*), 5.24 (d, 1H, *J* = 8.0 Hz, N*H*), 4.55 – 4.48 (m, 2H, C*H*), 4.37 (dt, 4H, *J* = 17.0, 6.8 Hz, OCH_2), 3.99 – 3.93 (m, 1H, alkyl*H*), 3.94 – 3.82 (m, 3H, alkyl*H*), 3.75 (d, 3H, *J* = 1.4 Hz, C(O)OCH_3), 3.72 – 3.61 (m, 12H, C(O)OCH_3, alkyl*H*), 3.29 (dq, 8H, *J* = 23.7, 4.7 Hz, alkyl*H*), 3.18 – 3.08 (m, 2H, alkyl*H*), 3.02 (ddd, 3H, *J* = 19.5, 13.5, 5.1 Hz, alkyl*H*), 2.13 (d, 4H, *J* = 1.2 Hz, alkyl*H*), 1.47 (s, 18H, C$(CH_3)_3$), 1.42 (s, 9H, C$(CH_3)_3$), 1.39 (d, 9H, *J* = 3.7 Hz, C$(CH_3)_3$); **^{13}C NMR** (151 MHz, $CDCl_3$): δ 178.74, 178.73, 178.6, 172.6, 171.8, 171.7, 156.5, 156.45, 156.44, 155.7, 155.3, 138.84, 138.82, 138.2, 138.1, 137.44, 137.39, 128.90, 128.85, 126.3, 126.1, 116.42, 116.38, 81.4, 81.32, 81.30, 80.6, 79.9, 66.40, 66.38, 66.35, 53.5, 53.1, 53.0, 52.8, 52.4, 51.29, 51.25, 50.68, 50.66, 46.5, 46.4, 40.93, 40.91, 38.7, 38.6, 30.4, 28.5, 28.4, 28.2, 27.2, 27.1, 27.03, 26.99, 26.9 (note: additional peaks found from regioisomers and diastereomers); **IR** (ν_{max}, cm^{-1}) 2977, 2928, 2860, 1744, 1702, 1499, 1455, 1439, 1392, 1366, 1277, 1247, 1158, 1112, 1066, 1046, 922, 861, 735; **HRMS** (ESI): *m/z* calcd for $C_{25}H_{43}N_6O_8S^+$: 587.2858 $[M+H]^+$; found 587.2856.

Note: The product was isolated as a mixture of regioisomers and diastereomers where the dr was not determined due to the overlap of signals.

Sulfondiimidamide-tyrosine adduct S1d

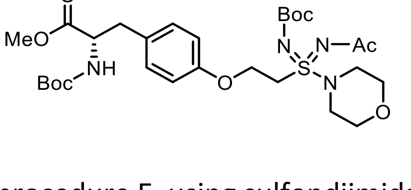

Prepared according to general procedure E, using sulfondiimidamide **6i** (15.9 mg, 0.05 mmol, 1.0 equiv.), triethylamine (14 µL, 0.10 mmol, 2.0 equiv.), Boc-Tyr-OMe (14.7 mg, 0.05 mmol, 1.0 equiv.) and MeCN (0.5 mL). Purification by flash column chromatography (SiO_2, EtOAc) afforded **S1d** a mixture of diastereomers as a colourless oil (5.7 mg, 19%).

***R*f**= 0.83 (Petrol/EtOAc 1:3); **^{1}H NMR** (400 MHz, $CDCl_3$): δ 7.06 (d, 2H, *J* = 8.2 Hz, Ar*H*), 6.81 (d, 2H, *J* = 8.3 Hz, Ar*H*), 4.96 (d, 1H, *J* = 8.3 Hz, N*H*), 4.54 (d, 1H, *J* = 7.3 Hz, C*H*), 4.30 (q, 2H, *J* = 5.2 Hz, alkyl*H*), 4.14 (ddd, 1H, *J* = 14.8, 5.7, 4.2 Hz, alkyl*H*), 4.02 (ddd, 1H, *J* = 14.9, 6.8,

4.6 Hz, alkyl*H*), 3.82 – 3.68 (m, 7H, OCH_2, C(O)OCH_3), 3.44 (dd, 4H, *J* = 5.9, 3.0 Hz, NCH_2), 3.13 – 2.89 (m, 2H, alkyl*H*), 2.15 (s, 3H, C(O)CH_3), 1.47 (d, 9H, *J* = 1.2 Hz, C(CH_3)$_3$), 1.42 (s, 9H, C(CH_3)$_3$); **^{13}C NMR** (151 MHz, $CDCl_3$): δ 178.7, 172.5, 156.8, 156.5, 155.2, 130.8, 129.5, 114.6, 81.0, 80.1, 66.6, 61.1, 54.6, 52.4, 51.4, 46.2, 37.6, 28.5, 28.2, 27.2; **IR** (ν_{max}, cm^{-1}) 1698, 1509, 1457, 1367, 1279, 1161, 1113, 1047, 923, 741, 650, 611; **HRMS** (ESI): *m/z* calcd for $C_{28}H_{45}N_4O_9S^+$: 613.2902 [M+H]$^+$; found 613.2885.

Note: The product was isolated as a mixture of diastereomers where the dr was not determined due to the overlap of signals.

Sulfondiimidamide-cysteine adduct S1e

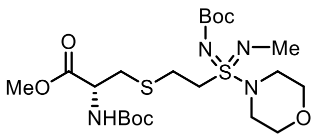

Prepared according to general procedure E, using sulfondiimidamide **6k** (14.5 mg, 0.05 mmol, 1.0 equiv.), triethylamine (14 µL, 0.10 mmol, 2.0 equiv.), Boc-Cys-OMe (12 µL, 0.05 mmol, 1.0 equiv.) and MeCN (0.5 mL). Purification by flash column chromatography (SiO_2, petrol/EtOAc 1:1) afforded **S2a** a mixture of diastereomers as a colourless oil (20.1 mg, 77%).

***R*$_f$**= 0.54 (Petrol/EtOAc 1:3); **^{1}H NMR** (600 MHz, $CDCl_3$): δ 5.37 (d, 1H, *J* = 8.0 Hz, N*H*), 4.53 (q, 1H, *J* = 6.3 Hz, C*H*), 3.81 – 3.68 (m, 7H, C(O)OCH_3, OCH_2), 3.50 (dddd, 1H, *J* = 13.7, 11.2, 5.3, 3.2 Hz, alkyl*H*), 3.43 – 3.35 (m, 1H, alkyl*H*), 3.32 (ddd, 2H, *J* = 12.5, 6.0, 3.3 Hz, NCH_2), 3.25 (ddt, 2H, *J* = 12.1, 6.0, 2.9 Hz, NCH_2), 3.04 – 2.85 (m, 4H, alkyl*H*), 2.77 (d, 3H, *J* = 3.6 Hz, NCH_3), 1.46 (d, 9H, *J* = 1.0 Hz, C(CH_3)$_3$), 1.44 (s, 9H, C(CH_3)$_3$); **^{13}C NMR** (151 MHz, $CDCl_3$): δ 171.41, 171.40, 158.4, 155.3, 80.5, 79.7, 66.9, 53.52, 53.45, 52.8, 51.9, 46.3, 35.2, 35.1, 30.42, 30.40, 28.4, 28.3, 26.2 (note: 3 additional peaks found from diastereomers); **IR** (ν_{max}, cm^{-1}) 2928, 2859, 1748, 1715, 1656, 1520, 1455, 1366, 1280, 1254, 1183, 1114, 1064, 1018, 928, 870, 744; **HRMS** (ESI): *m/z* calcd for $C_{22}H_{41}N_4O_8S_2^+$: 553.2360 [M+H]$^+$; found 553.2384.

Note: The product was isolated as a mixture of diastereomers where the dr was not determined due to the overlap of signals.

Sulfondiimidamide-lysine adduct S1f

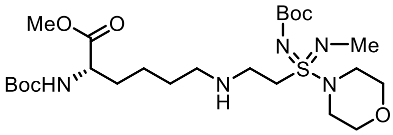

Prepared according to general procedure E, using sulfondiimidamide **6k** (14.5 mg, 0.05 mmol, 1.0 equiv.), triethylamine (14 µL, 0.10 mmol, 2.0 equiv.), Boc-Lys-OMe.HCl (14.8 mg, 0.05 mmol, 1.0 equiv.) and MeCN (0.5 mL). Purification by flash column chromatography (SiO_2, EtOAc/MeOH 1:0 to 10:1) afforded **S2b** a mixture of diastereomers as a colourless oil (18.2 mg, 65%).

***R*f**= 0.18 (EtOAc/MeOH 10:1); **^{1}H NMR** (600 MHz, $CDCl_3$): δ 5.14 (d, 1H, *J* = 8.3 Hz, N*H*), 4.29 (d, 1H, *J* = 7.0 Hz, C*H*), 4.00 (s, 1H, alkyl*H*), 3.81 – 3.68 (m, 7H, OCH_2, C(O)OCH_3), 3.67 – 3.58 (m, 1H, alkyl*H*), 3.47 – 3.36 (m, 4H, alkyl*H,* NCH_2), 3.24 (ddd, 2H, *J* = 12.0, 6.4, 3.0 Hz, NCH_2), 2.95 – 2.77 (m, 2H, alkyl*H*), 2.72 (s, 3H, NCH_3), 1.84 (t, 1H, *J* = 7.9 Hz, alkyl*H*), 1.76 (q, 2H, *J* = 7.4 Hz, alkyl*H*), 1.71 – 1.60 (m, 1H, alkyl*H*), 1.47 (s, 11H, C(CH_3)$_3$, alkyl*H*), 1.43 (d, 9H, *J* = 1.1 Hz, C(CH_3)$_3$); **^{13}C NMR** (151 MHz, $CDCl_3$): δ 173.1, 158.6, 158.6, 155.6, 80.5, 80.1, 66.7, 53.3, 52.5, 48.1, 47.9, 47.8, 46.1, 43.64, 43.62, 32.4, 29.8, 29.48, 29.45, 28.5, 28.3, 26.8, 22.8, 22.7 (note: additional peaks found from diastereomers); **IR** (ν_{max}, cm^{-1}) 2930, 2860, 1744, 1712, 1656, 1521, 1456, 1392, 1366, 1283, 1254, 1182, 1113, 1066, 1019, 930, 868, 746; **HRMS** (ESI): *m/z* calcd for $C_{24}H_{48}N_5O_7S^+$: 550.3269 $[M+H]^+$; found 550.3255.

Note: The product was isolated as a mixture of diastereomers where the dr was not determined due to the overlap of signals.

2.5 Procedure for competition reactions of Lys- and Cys- derivatives

Procedure adapted from literature.[4] The respective sulfondiimidamide (19.0 mg, 0.06 mmol, 1.0 equiv.) was added to an oven-dried microwave vial and flushed with N_2. Anhydrous MeCN (0.3 mL) was added followed by anhydrous Et_3N (17 µL, 0.12 mmol, 2.0 equiv.). Boc-Cys-OMe (12 µL, 0.06 mmol, 1.0 equiv.) and Boc-Lys-OMe.HCl (17.8 mg, 0.06 mmol, 1.0 equiv.) were dissolved in MeCN (0.3 mL) and then added. The reaction mixture was stirred at rt for 18 h and then concentrated *in vacuo*. Purification by flash column chromatography (SiO_2,

Petrol/EtOAc/MeOH 1:1:0 to 0:1:0 to 0:10:1 to afford Cys-adduct **S1a** (21 mg, 63%) and Lys-adduct **S1b** (5.0 mg, 15%) with matching analytical data to those reported above.

2.6 General procedure F – determination of half-life for the conjugate addition of Boc-Cys-OMe

The rate determination was conducted using nuclear magnetic resonance (NMR) spectroscopy. ^{1}H NMR spectra were obtained on a Brunker AVIIIHD 500 (500 MHz) using 5 mm NMR tubes at 20 ˚C. The spectra were recorded using zg60 pulse program with a spectral width of 10000 Hz and a relaxation time of 1 second. For the majority of substrates, each acquisition consisted of 4 scans with increasing time inverval from 0 seconds to 600 seconds for a total elapsed time of 3 h. Consuption of starting material was determined by monitoring the disappearance of alkene proton signals (6.0 to 6.5 ppm) relative to a dibromomethane internal standard.

Sulfondiimidamide (0.05 mmol, 1.0 equiv.) was dissolved in MeCN-d^3 (0.4 mL) in an NMR tube. Dibromomethane (0.1 mmol, 2.0 equiv.) was then added as an internal standard. Locking and shimming of the reaction mixture was performed, and a initial spectrum was obtained. The NMR tube was then removed from the magnet and Boc-Cys-OMe (1.0 or 10.0 equiv.) in a 0.1 mL solution of MeCN-d^3 and Et_3N (0.05 mmol, 1.0 equiv.) were added and the mixture shaken. Acquisition of subsequent NMR spectra was started directly after the addition of the nucleophile. For substrates that were treated with 1.0 equiv. of Boc-Cys-OMe, the consumption of starting material was plotted as the inverse against time and the half-life was caluclated by second order kinetics. For substrates that were treated with 10.0 equiv. of Boc-Cys-OMe, the concentration of starting material was plotted as the natural log against time and the half-life was calculated by psudo-first order kinetics. In both cases, the half-life was calculated through the equation obtained from the line-of-best fit. Example plots are shown below for compound **8b** and **8d**.

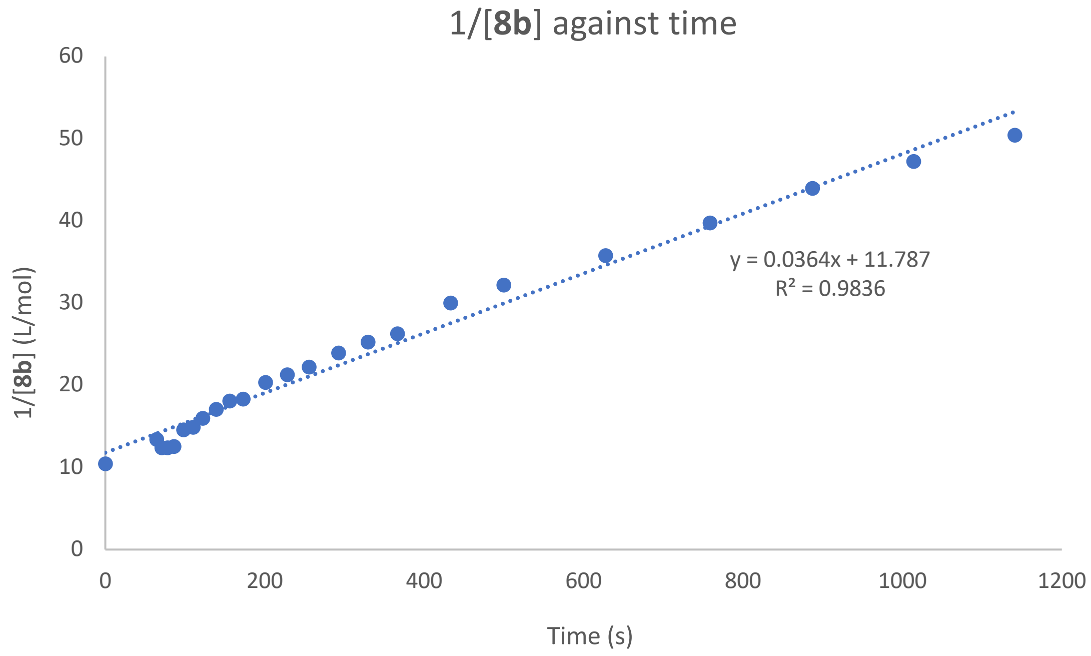

***Figure 5-1** 1/[**8b**] against time.*

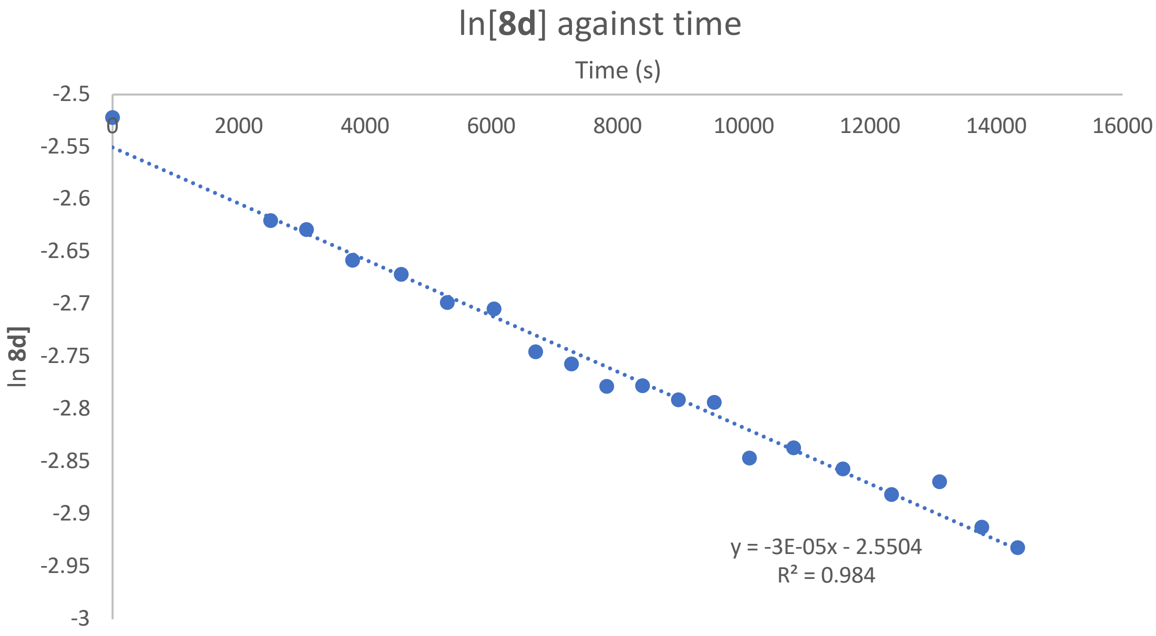

***Figure 5-2** Ln[**8d**] against time.*

Sulfondiimidamide-Cysteine Adduct S2a

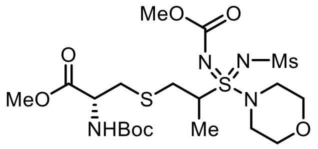

Prepared according to general procedure F, using **8c** (16.3 mg, 0.05 mmol, 1.0 equiv.), *N*-(*tert*-Butoxycarbonyl)-L-cysteine methyl ester (11.8 mg, 0.05 mmol, 1.0 equiv.), Et_3N (7 µL, 0.05 mmol, 1.0 equiv.) and MeCN-d^3 (0.5 mL, 0.1 M conc.). The half-life calculated was calculated by second order kinetics as 25 seconds.

HRMS (ESI): *m/z* calcd for $C_{19}H_{37}N_4O_9S_3^+$: 561.1717 $[M+H]^+$; found 561.1724.

Sulfondiimidamide-Cysteine Adduct S2b

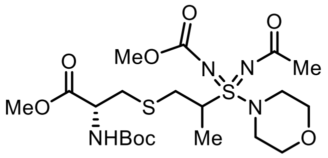

Prepared according to general procedure F, using **8a** (14.5 mg, 0.05 mmol, 1.0 equiv.), *N*-(*tert*-Butoxycarbonyl)-L-cysteine methyl ester (11.8 mg, 0.05 mmol, 1.0 equiv.), Et_3N (7 µL, 0.05 mmol, 1.0 equiv.) and MeCN-d^3 (0.5 mL, 0.1 M conc.). The half-life calculated from two experiments by second order kinetics were 207 seconds and 228 seconds respectively, the average half-life calculated was 218 seconds.

HRMS (ESI): *m/z* calcd for $C_{20}H_{37}N_4O_8S_2^+$: 525.2047 $[M+H]^+$; found 525.2056.

Sulfondiimidamide-Cysteine Adduct S2c

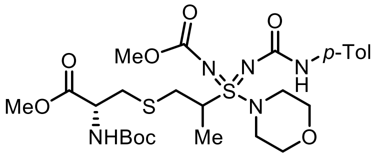

Prepared according to general procedure F, using **8b** (19.0 mg, 0.05 mmol, 1.0 equiv.), *N*-(*tert*-Butoxycarbonyl)-L-cysteine methyl ester (11.8 mg, 0.05 mmol, 1.0 equiv.), Et_3N (7 µL, 0.05 mmol, 1.0 equiv.) and MeCN-d^3 (0.5 mL, 0.1 M conc.). The half-life calculated by second order kinetics was 287 seconds.

HRMS (ESI): *m/z* calcd for $C_{26}H_{42}N_5O_8S_2^+$: 616.2469 $[M+H]^+$; found 616.2484.

Sulfondiimidamide-Cysteine Adduct S2d

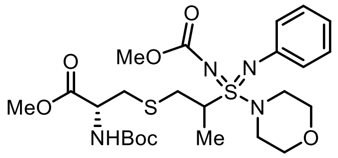

Prepared according to general procedure F, using **8e** (16.2 mg, 0.05 mmol, 1.0 equiv.), *N*-(*tert*-Butoxycarbonyl)-L-cysteine methyl ester (118 mg, 0.50 mmol, 10.0 equiv.), Et_3N (7 µL, 0.05 mmol, 1.0 equiv.) and MeCN-d^3 (0.5 mL, 0.1 M conc.). The half-life calculated by pseudo-first order kinetics was 7567 seconds. Purification by flash column chromatography (SiO_2, petrol/EtOAc, 1:1) afforded **S2d** as a mixture of diastereomers as a colourless oil.

^{1}H NMR (600 MHz, CD_3CN): δ 7.27 – 7.19 (m, 2H, Ar*H*), 7.09 (ddt, 2H, *J* = 8.1, 4.7, 1.2 Hz, Ar*H*), 6.97 (td, 1H, *J* = 7.4, 1.1 Hz, Ar*H*), 5.78 (t, 1H, *J* = 11.3 Hz, N*H*), 4.49 – 4.34 (m, 1H, C*H*), 4.19 –- 3.91 (m, 1H, alkyl*H*), 3.74 – 3.67 (m, 3H, C(O)OC*H*$_3$), 3.62 (dtd, 2H, *J* = 12.4, 6.5, 3.0 Hz, OC*H*$_2$), 3.59 – 3.51 (m, 5H, OC*H*$_2$, C(O)C*H*$_3$), 3.51 – 3.44 (m, 2H, NC*H*$_2$), 3.38 – 3.33 (m, 2H, NC*H*$_2$), 3.32 – 3.16 (m, 0.5H, alkyl*H*), 3.04 (ddt, 1H, *J* = 26.3, 14.0, 4.9 Hz, alkyl*H*), 2.90 (dt, 0.5H, *J* = 14.4, 7.4 Hz, alkyl*H*), 2.86 – 2.77 (m, 1H, alkyl*H*), 2.76 – 2.65 (m, 1H, alkyl*H*), 1.56 – 1.46 (m, 3H, C*H*$_3$), 1.41 (d, 9H, *J* = 2.7 Hz, C(C*H*$_3$)$_3$); **^{13}C NMR** (151 MHz, CD_3CN): δ 172.6, 172.55, 172.47, 172.4, 159.62, 159.60, 159.4, 156.5, 144.78, 144.76, 144.74, 144.72, 130.01, 129.99, 129.96, 124.6, 124.5, 123.2, 123.1, 80.4, 80.3, 67.39, 67.37, 63.4, 63.2, 62.8, 54.8, 54.7, 54.2, 54.1, 53.4, 53.3, 53.08, 53.07, 53.05, 48.23, 48.16, 47.98, 47.96, 46.95, 35.6, 35.3, 35.0, 34.9, 34.5, 34.3, 33.8 (note: additional peaks found from diastereomers); **HRMS** (ESI): *m/z* calcd for $C_{24}H_{39}N_4O_7S_2^+$: 559.2255 [M+H]$^+$; found 559.2242.

Note: The product was isolated as a mixture of diastereomers where the dr was not determined due to the overlap of signals.

Sulfondiimidamide-Cysteine Adduct S2e

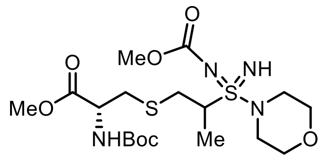

Prepared according to general procedure F, using **7a** (12.4 mg, 0.05 mmol, 1.0 equiv.), *N*-(*tert*-Butoxycarbonyl)-L-cysteine methyl ester (11.8 mg, 0.05 mmol, 1.0 equiv.) or (118 mg,

0.50 mmol, 10.0 equiv.), Et_3N (7 µL, 0.05 mmol, 1.0 equiv.) and MeCN-d^3 (0.5 mL, 0.1 M conc.). The half-life calculated by second order kinetics was 52859 seconds. The half-life calculated from two experiments by pseudo-first order kinetics was 8682 and 7715 seconds, the average half-life calculated was 8199 seconds.

HRMS (ESI): *m/z* calcd for $C_{18}H_{35}N_4O_7S_2^+$: 483.1942 [M+H]$^+$; found 483.1929.

Sulfondiimidamide-Cysteine Adduct S2f

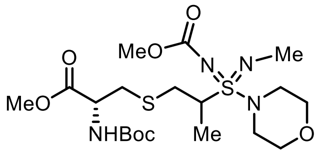

Prepared according to general procedure F, using **8d** (13.1 mg, 0.05 mmol, 1.0 equiv.), *N*-(*tert*-Butoxycarbonyl)-L-cysteine methyl ester (118 mg, 0.50 mmol, 10.0 equiv.), Et_3N (7 µL, 0.05 mmol, 1.0 equiv.) and MeCN-d^3 (0.5 mL, 0.1 M conc.). The half-life calculated by pseudo first order kinetics was 25945 seconds.

HRMS (ESI): *m/z* calcd for $C_{19}H_{37}N_4O_7S_2^+$: 497.2098 [M+H]$^+$; found 497.2085.

Sulfondiimidamide-Cysteine Adduct S2g

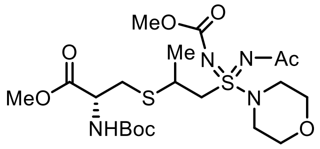

Prepared according to general procedure F, using **8f** (*E:Z* = 3.5:1) (14.5 mg, 0.05 mmol, 1.0 equiv.), *N*-(*tert*-Butoxycarbonyl)-L-cysteine methyl ester (11.8 mg, 0.05 mmol, 1.0 equiv.), Et_3N (7 µL, 0.05 mmol, 1.0 equiv.) and MeCN-d^3 (0.5 mL, 0.1 M conc.). The half-life calculated from two experiments by second order kinetics were 25.2 seconds and 28.5 seconds respectively, the average half-life calculated was 27 seconds.

HRMS (ESI): *m/z* calcd for $C_{20}H_{37}N_4O_8S_2^+$: 525.2047 [M+H]$^+$; found 525.2037.

Sulfondiimidamide-Cysteine Adduct S2h

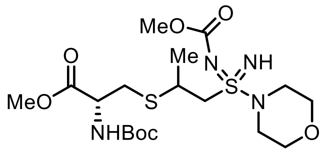

Prepared according to general procedure F, using **7b** (*E:Z* = 10:1) (12.4 mg, 0.05 mmol, 1.0 equiv.), *N*-(*tert*-Butoxycarbonyl)-L-cysteine methyl ester (118 mg, 0.50 mmol, 10.0 equiv.), Et_3N (7 µL, 0.05 mmol, 1.0 equiv.) and MeCN-d^3 (0.5 mL, 0.1 M conc.). The half-life calculated by pseudo first order kinetics was 775 seconds.

HRMS (ESI): *m/z* calcd for $C_{18}H_{35}N_4O_7S_2^+$: 483.1942 [M+H]$^+$; found 483.1946.

Sulfondiimidamide-Cysteine Adduct S2i

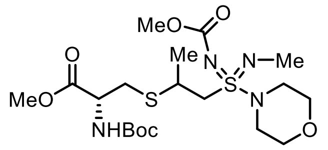

Prepared according to general procedure F, using **8g** (*E:Z* = >20:1) (13.1 mg, 0.05 mmol, 1.0 equiv.), *N*-(*tert*-Butoxycarbonyl)-L-cysteine methyl ester (118 mg, 0.50 mmol, 10.0 equiv.), Et_3N (7 µL, 0.05 mmol, 1.0 equiv.) and MeCN-d^3 (0.5 mL, 0.1 M conc.). The half-life calculated by pseudo first order kinetics was 1561 seconds.

HRMS (ESI): *m/z* calcd for $C_{19}H_{37}N_4O_7S_2^+$: 497.2098 [M+H]$^+$; found 497.2123.

Acrylamide-Cysteine Adduct S2j

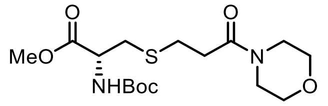

Prepared according to general procedure F, using 4-acryloylmorpholine **14** (7.1 mg, 0.05 mmol, 1.0 equiv.), *N*-(*tert*-Butoxycarbonyl)-L-cysteine methyl ester (11.8 mg, 0.05 mmol, 1.0 equiv.), Et_3N (7 µL, 0.05 mmol, 1.0 equiv.) and MeCN-d^3 (0.5 mL, 0.1 M conc.). The half-life calculated by second order kinetics was 15720 seconds.

HRMS (ESI): *m/z* calcd for $C_{16}H_{29}N_2O_6S^+$: 377.1741 [M+H]$^+$; found 377.1732.

Sulfonamide-Cysteine Adduct S2k

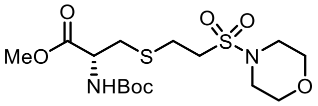

Prepared according to general procedure F, using 4-(vinylsulfonyl)morpholine **13** (8.9 mg, 0.05, 1.0 equiv.), *N*-(*tert*-Butoxycarbonyl)-L-cysteine methyl ester (11.8 mg, 0.05 mmol, 1.0 equiv.), Et_3N (7 µL, 0.05 mmol, 1.0 equiv.) and MeCN-d^3 (0.5 mL, 0.1 M conc.). The half-life calculated by second order kinetics was 562 seconds.

HRMS (ESI): *m/z* calcd for $C_{15}H_{29}N_2O_7S_2^+$: 413.1411 [M+H]$^+$; found 413.1404.

Sulfondiimidamide-Cysteine Adduct S2l

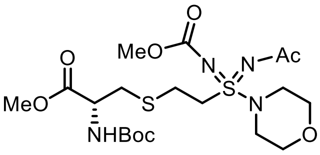

Prepared according to general procedure F, using **6o** (13.8 mg, 0.05 mmol, 1.0 equiv.), *N*-(*tert*-Butoxycarbonyl)-L-cysteine methyl ester (11.8 mg, 0.05 mmol, 1.0 equiv.), Et_3N (7 µL, 0.05 mmol, 1.0 equiv.) and MeCN-d^3 (0.5 mL, 0.1 M conc.). The reaction was too fast to accurately determine the half-life.

HRMS (ESI): *m/z* calcd for $C_{19}H_{35}N_4O_8S_2^+$: 511.1891 [M+H]$^+$; found 511.1883.

Sulfondiimidamide-Cysteine Adduct S2m

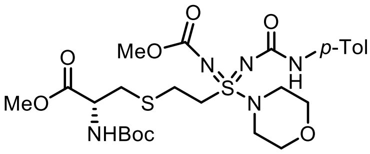

Prepared according to general procedure F, using **6l** (18.3 mg, 0.05 mmol, 1.0 equiv.), *N*-(*tert*-Butoxycarbonyl)-L-cysteine methyl ester (11.8 mg, 0.05 mmol, 1.0 equiv.), Et_3N (7 µL, 0.05 mmol, 1.0 equiv.) and MeCN-d^3 (0.5 mL, 0.1 M conc.). The reaction was too fast to accurately determine the half-life. Purification by flash column chromatography (SiO_2, petrol/EtOAc, 1:1) afforded **S2m** as a mixture of diastereomers as a colourless oil.

^{1}H NMR (600 MHz, $CDCl_3$): δ 7.30 (d, 2H, *J* = 7.9 Hz, Ar*H*), 7.14 – 7.11 (m, 1H, N*H*), 7.07 (d, 2H, *J* = 8.2 Hz, Ar*H*), 5.34 (t, 1H, *J* = 8.2 Hz, N*H*), 4.54 (s, 1H, C*H*), 3.96 (s, 1H, alkyl*H*), 3.84 (q, 1H, *J* = 7.8 Hz, alkyl*H*), 3.81 – 3.77 (m, 4H, OCH_2), 3.76 (d, 3H, *J* = 2.9 Hz, C(O)CH_3), 3.69 (s, 3H, C(O)CH_3), 3.43 (dtt, 4H, *J* = 17.3, 12.2, 7.2 Hz, NCH_2), 3.05 (td, 1H, *J* = 13.5, 5.2 Hz, alkyl*H*), 2.95 (dq, 3H, *J* = 13.5, 7.3 Hz, alkyl*H*), 2.28 (s, 3H, ArCH_3), 1.44 (s, 9H, C(CH_3)$_3$); **^{13}C NMR** (151 MHz, CD_3CN): δ 171.4, 158.5, 155.5, 155.3, 136.3, 132.9, 129.5, 119.0, 80.6, 66.6, 53.5, 53.4, 52.9, 51.8, 51.7, 46.6, 35.3, 35.2, 28.4, 25.4, 25.3, 20.9 (note: additional peaks found from diastereomers); **HRMS** (ESI): *m/z* calcd for $C_{25}H_{40}N_5O_8S_2^+$: 602.2313 [M+H]$^+$; found 602.2298.

Note: The product was isolated as a mixture of diastereomers where the dr was not determined due to the overlap of signals.

Sulfondiimidamide-Cysteine Adduct S2n

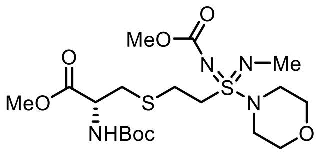

Prepared according to general procedure F, using **6r** (12.4 mg, 0.05 mmol, 1.0 equiv.), *N*-(*tert*-Butoxycarbonyl)-L-cysteine methyl ester (11.8 mg, 0.05 mmol, 1.0 equiv.), Et_3N (7 µL, 0.05 mmol, 1.0 equiv.) and MeCN-d^3 (0.5 mL, 0.1 M conc.). The reaction was too fast to accurately determine the half-life.

HRMS (ESI): *m/z* calcd for $C_{19}H_{35}N_4O_8S_2^+$: 511.1891 [M+H]$^+$; found 511.1883.

3. Single Crystal X-ray Diffraction

Single-crystal X-ray diffraction data were collected using either a Rigaku Oxford Diffraction SuperNova diffractometer equipped with a Cu Kα microfocus source (λ = 1.5418 Å) and an Atlas CCD area detector, or a Rigaku Oxford Diffraction Synergy DW diffractometer equipped with a MicroMax-007 HF microfocus rotating-anode X-ray generator and a HyPix-Arc 150° pixel detector. Individual crystals were selected under Paratone-N oil, mounted on MiTeGen loops, and cooled to 150 K using an Oxford Cryosystems nitrogen-flow device.[5]

Data acquisition, indexing, integration, and multi-scan scaling were performed with CrysAlisPro.[6] Unit-cell constants were refined against all suitable reflections during processing.

Structure solution methods were selected according to data quality: compound 6m and 6o was solved using SHELXT,[7] while compound 8d was solved using ShelXS.[8] Final refinements for all structures were carried out against F^2 using the CRYSTALS refinement suite.[9, 10] Complete crystallographic information, including refinement details and atomic coordinates, is provided in the accompanying CIF files.

Crystallographic data have been deposited with the Cambridge Crystallographic Data Centre under deposition numbers CCDC 2522888-2522890. These files are available free of charge from the CCDC via www.ccdc.cam.ac.uk/data_request/cif.

Table S1. Crystal data and structure refinement for 6m.

CCDC code	2522888	
Empirical formula	$C_9H_{17}N_3O_5S_2$	
Formula weight	311.38	
Temperature	100 K	
Wavelength	1.54184 Å	
Crystal system	Monoclinic	
Space group	P 21/c	
Unit cell dimensions	a = 8.78770(10) Å	a= 90°.
	b = 14.61610(10) Å	b= 96.0740(10)°.
	c = 10.44070(10) Å	g = 90°.
Volume	1333.49(2) $Å^3$	
Z	4	
Density (calculated)	1.551 Mg/m^3	
Absorption coefficient	3.839 mm^{-1}	
F(000)	656	
Crystal size	0.388 x 0.197 x 0.053 mm^3	
Theta range for data collection	5.061 to 75.915°.	
Index ranges	-10<=h<=10, -18<=k<=16, -13<=l<=13	
Reflections collected	46946	
Independent reflections	2748 [R(int) = 0.041]	
Completeness to theta = 74.397°	99.5 %	
Absorption correction	Semi-empirical from equivalents	
Max. and min. transmission	0.82 and 0.43	
Refinement method	Full-matrix least-squares on F^2	
Data / restraints / parameters	2748 / 0 / 172	
Goodness-of-fit on F^2	1.0009	
Final R indices [I>2sigma(I)]	R1 = 0.0256, wR2 = 0.0686	
R indices (all data)	R1 = 0.0263, wR2 = 0.0691	
Largest diff. peak and hole	0.36 and -0.42 e.$Å^{-3}$	

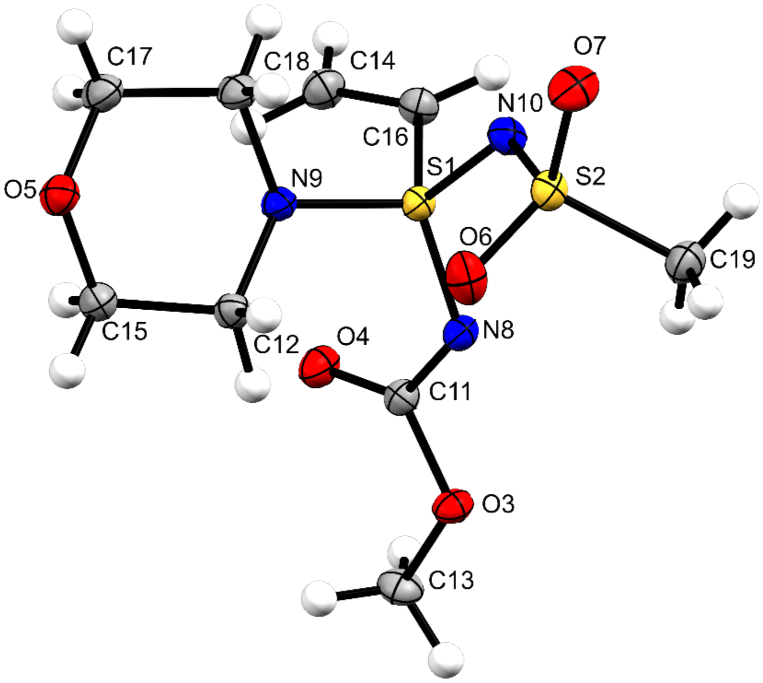

Table S2. Crystal data and structure refinement for 6o.

CCDC code	2522889	
Empirical formula	$C_{10}H_{17}N_3O_4S$	
Formula weight	275.33	
Temperature	150 K	
Wavelength	1.54184 Å	
Crystal system	Triclinic	
Space group	P -1	
Unit cell dimensions	a = 8.27083(19) Å	α= 75.5232(19)°.
	b = 8.6041(2) Å	β= 88.7798(18)°.
	c = 9.6895(2) Å	γ = 78.4972(19)°.
Volume	653.93(3) $Å^3$	
Z	2	
Density (calculated)	1.398 Mg/m^3	
Absorption coefficient	2.329 mm^{-1}	
F(000)	292	
Crystal size	0.227 x 0.137 x 0.106 mm^3	
Theta range for data collection	4.716 to 74.377°.	
Index ranges	-10<=h<=10, -10<=k<=10, -12<=l<=12	
Reflections collected	39834	
Independent reflections	2666 [R(int) = 0.027]	
Completeness to theta = 74.377°	99.7 %	
Absorption correction	Semi-empirical from equivalents	
Max. and min. transmission	0.78 and 0.49	
Refinement method	Full-matrix least-squares on F^2	
Data / restraints / parameters	2666 / 0 / 163	
Goodness-of-fit on F^2	0.9776	
Final R indices [I>2sigma(I)]	R1 = 0.0274, wR2 = 0.0757	
R indices (all data)	R1 = 0.0287, wR2 = 0.0768	
Largest diff. peak and hole	0.42 and -0.35 e.$Å^{-3}$	

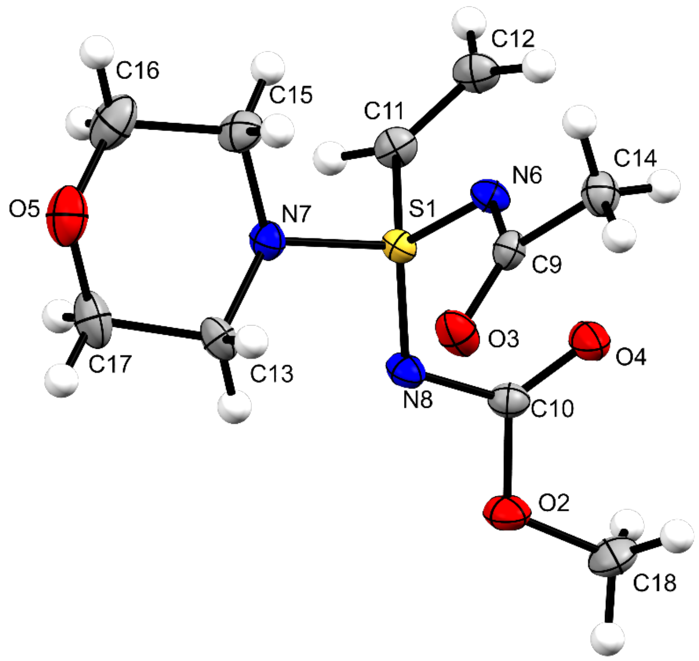

Table S1. Crystal data and structure refinement for 8d.

CCDC code	2522890	
Empirical formula	$C_{10}H_{19}N_3O_3S$	
Formula weight	261.35	
Temperature	150 K	
Wavelength	1.54184 Å	
Crystal system	Monoclinic	
Space group	C 1 c 1	
Unit cell dimensions	a = 9.8439(2) Å	α= 90°.
	b = 17.7917(4) Å	β= 91.518(2)°.
	c = 7.30163(18) Å	γ = 90°.
Volume	1278.36(5) $Å^3$	
Z	4	
Density (calculated)	1.358 Mg/m^3	
Absorption coefficient	2.288 mm^{-1}	
F(000)	560	
Crystal size	0.204 x 0.114 x 0.078 mm^3	
Theta range for data collection	4.972 to 76.134°.	
Index ranges	-12<=h<=12, -22<=k<=22, -9<=l<=8	
Reflections collected	13235	
Independent reflections	2549 [R(int) = 0.034]	
Completeness to theta = 76.134°	99.8 %	
Absorption correction	Semi-empirical from equivalents	
Max. and min. transmission	0.84 and 0.49	
Refinement method	Full-matrix least-squares on F^2	
Data / restraints / parameters	2549 / 2 / 154	
Goodness-of-fit on F^2	1.0112	
Final R indices [I>2sigma(I)]	R1 = 0.0237, wR2 = 0.0609	
R indices (all data)	R1 = 0.0245, wR2 = 0.0620	
Absolute structure parameter	0.011(5)	
Largest diff. peak and hole	0.14 and -0.19 e.$Å^{-3}$	

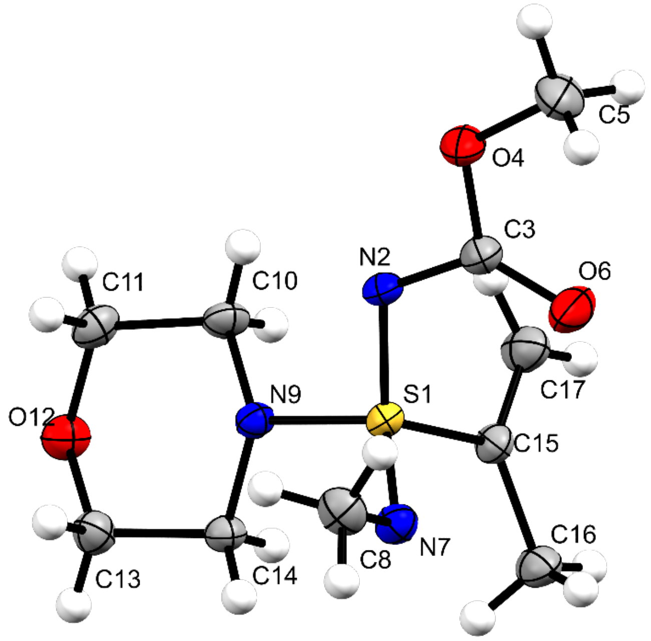

4. References

1. M. Ding, Z.-X. Zhang, T. Q. Davies and M. C. Willis, *Org. Lett.*, 2022, **24**, 1711–1715.
2. Z.-X. Zhang, C. Bell, M. Ding and M. C. Willis, *J. Am. Chem. Soc.*, 2022, **144**, 11851–11858.
3. H. Chen, R. Huang, Z. Li, W. Zhu, J. Chen, Y. Zhan and B. Jiang, *Org. Biomol. Chem.*, 2017, **15**, 7339–7345.
4. Y. T. Wong, C. Bell and M. C. Willis, *Chem. Sci.*, 2025, **16**, 12860–12866.
5. J. Cosier and A. M. Glazer, *J. Appl. Crystallogr.*, 1986, **19**, 105–107.
6. **N.d.**
7. G. Sheldrick, *Acta Crystallogr. Sect. Found. Adv.*, 2015, **71**, 3–8.
8. G. Sheldrick, SHELXS86 (1986) Program for Crystal Structure solution. Acta Crystallographica, A24, 351-359. - References - Scientific Research Publishing," can be found under, https://www.scirp.org/reference/referencespapers?referenceid=1398541, (accessed 14 January, 2026).
9. P. W. Betteridge, J. R. Carruthers, R. I. Cooper, K. Prout and D. J. Watkin, *J. Appl. Crystallogr.*, 2003, **36**, 1487–1487.
10. P. Parois, R. I. Cooper and A. L. Thompson, *Chem. Cent. J.*, 2015, **9**, 30.

5. NMR Spectra

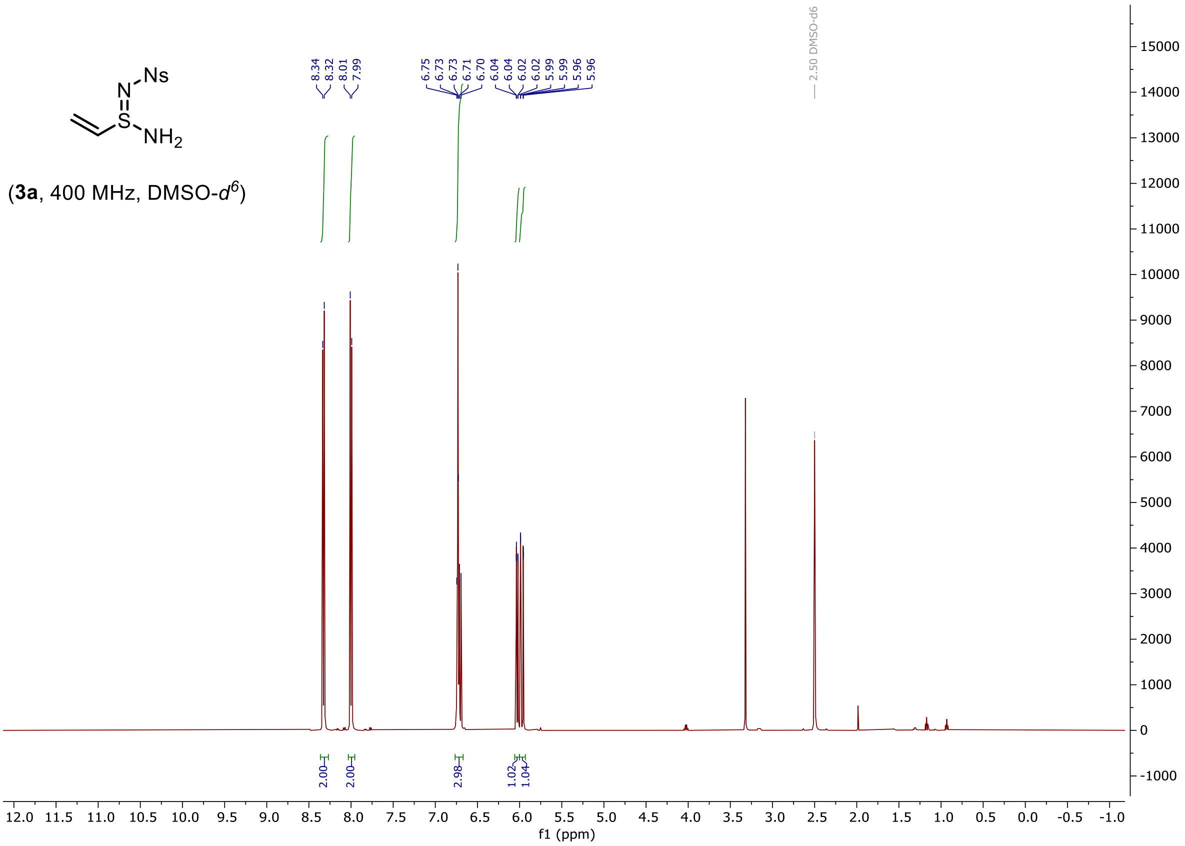

(**3a**, 400 MHz, DMSO-d^6)

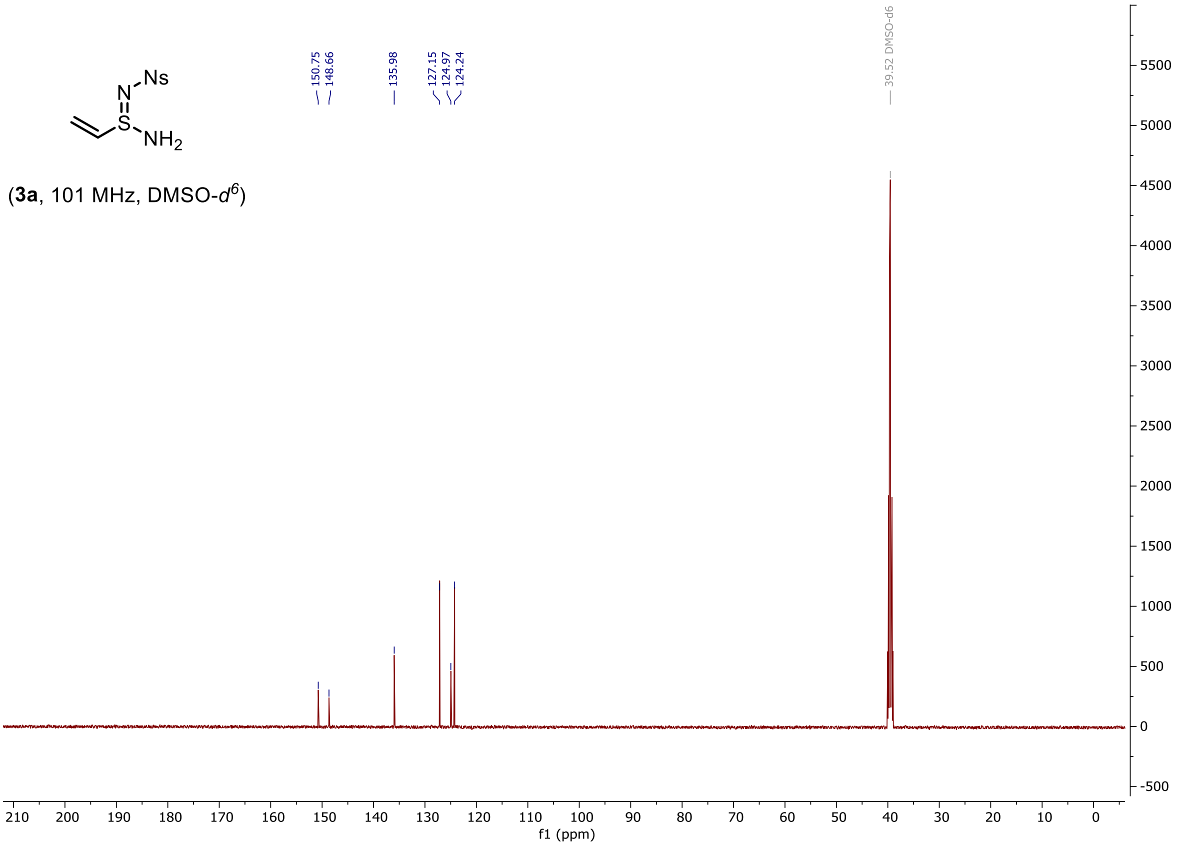

(**3a**, 101 MHz, DMSO-d^6)

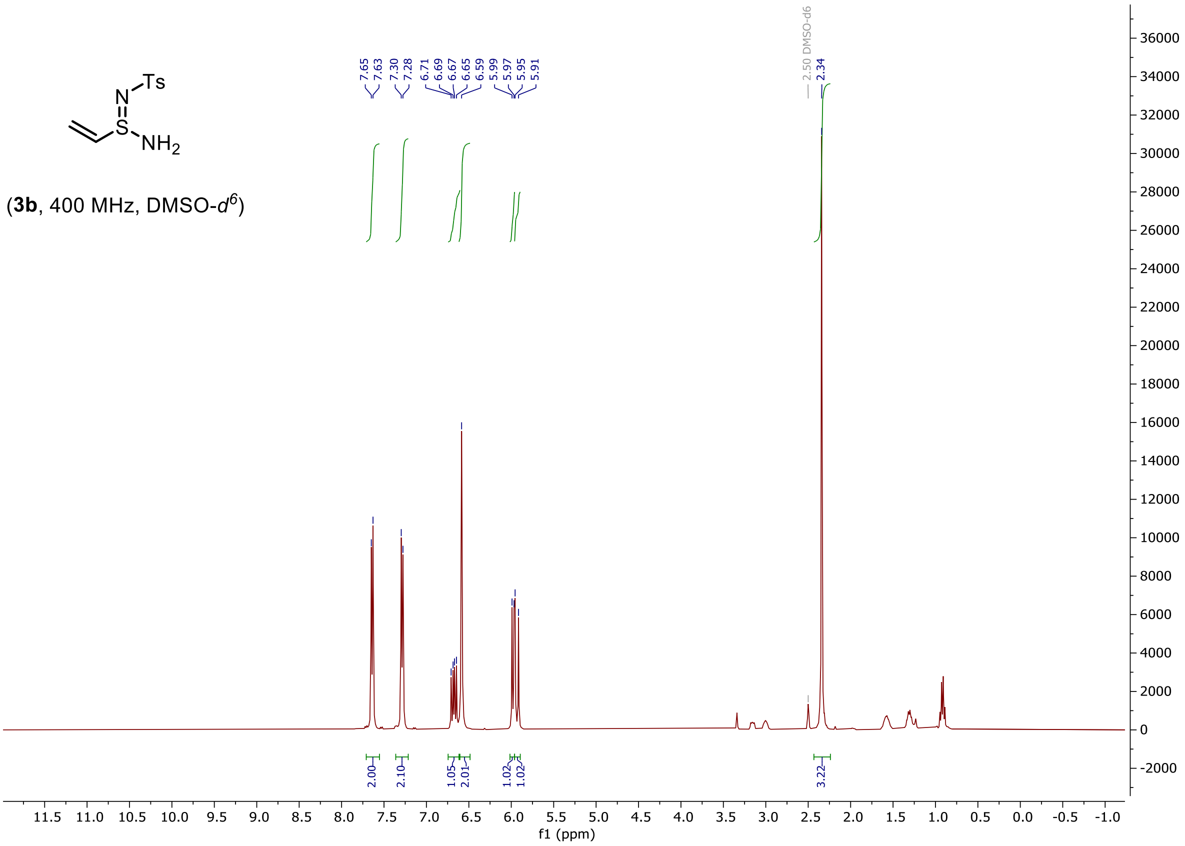

(**3b**, 400 MHz, DMSO-d^6)

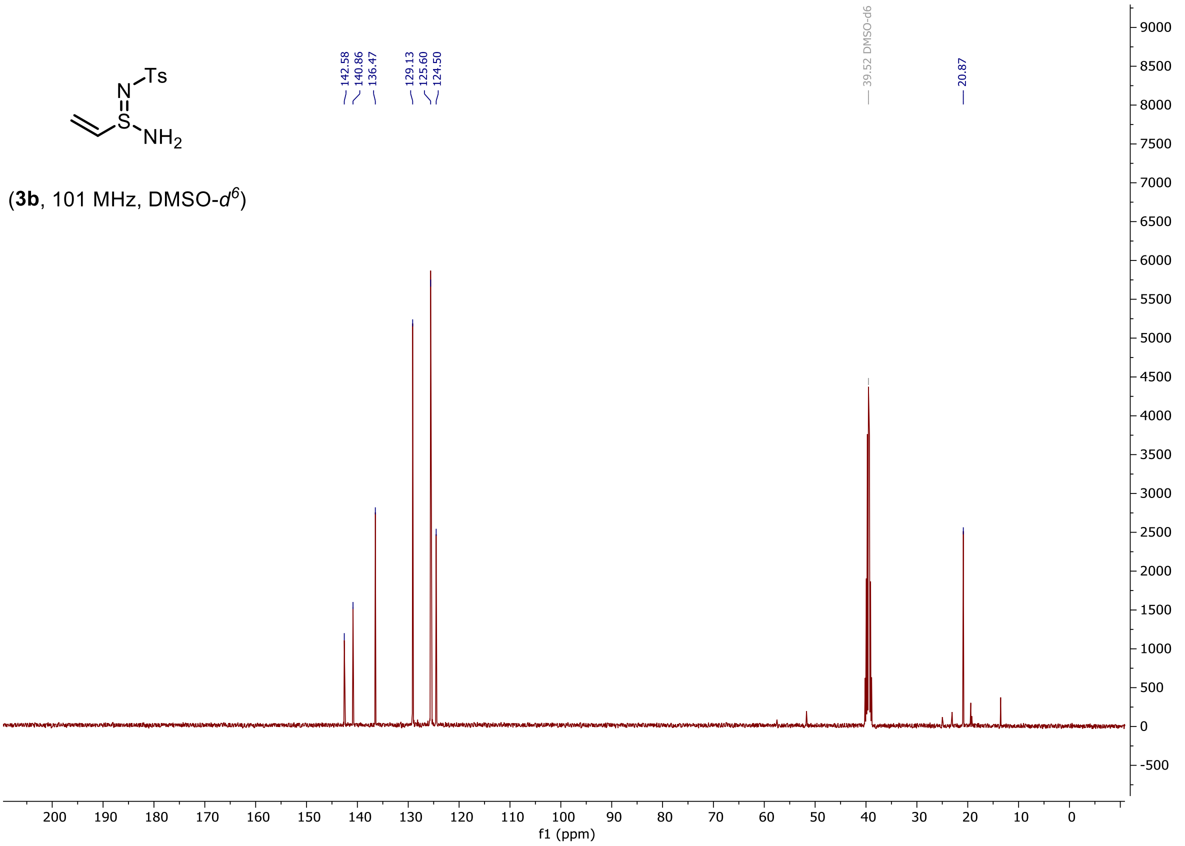

(**3b**, 101 MHz, DMSO-d^6)

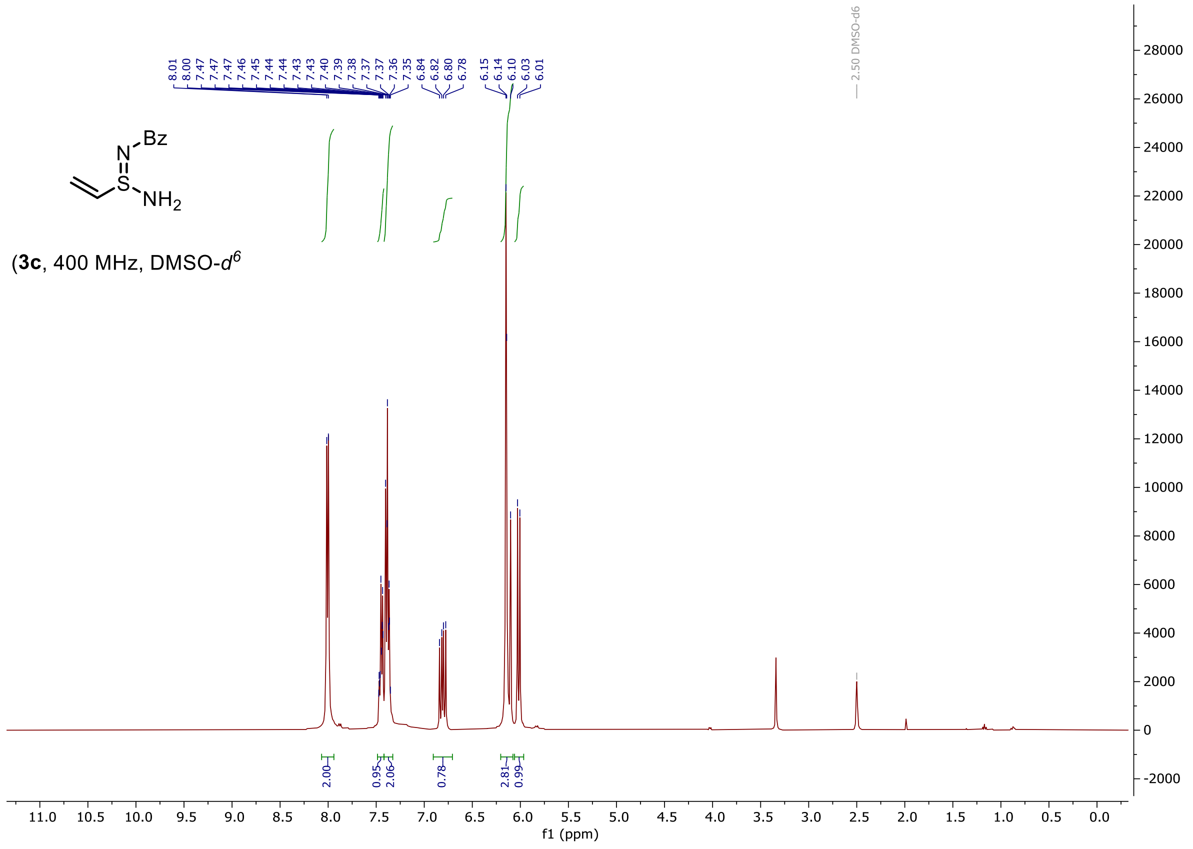

(3c, 400 MHz, DMSO-d6
8.01 8.00 7.47 7.47 7.47 7.46 7.45 7.44 7.44 7.43 7.43 7.40 7.39 7.38 7.37 7.37 7.36 7.35 6.84 6.82 6.80 6.78 6.15 6.14 6.10 6.03 6.01
2.50 DMSO-d6
2.00 0.95 2.06 0.78 2.81 0.99
f1 (ppm)

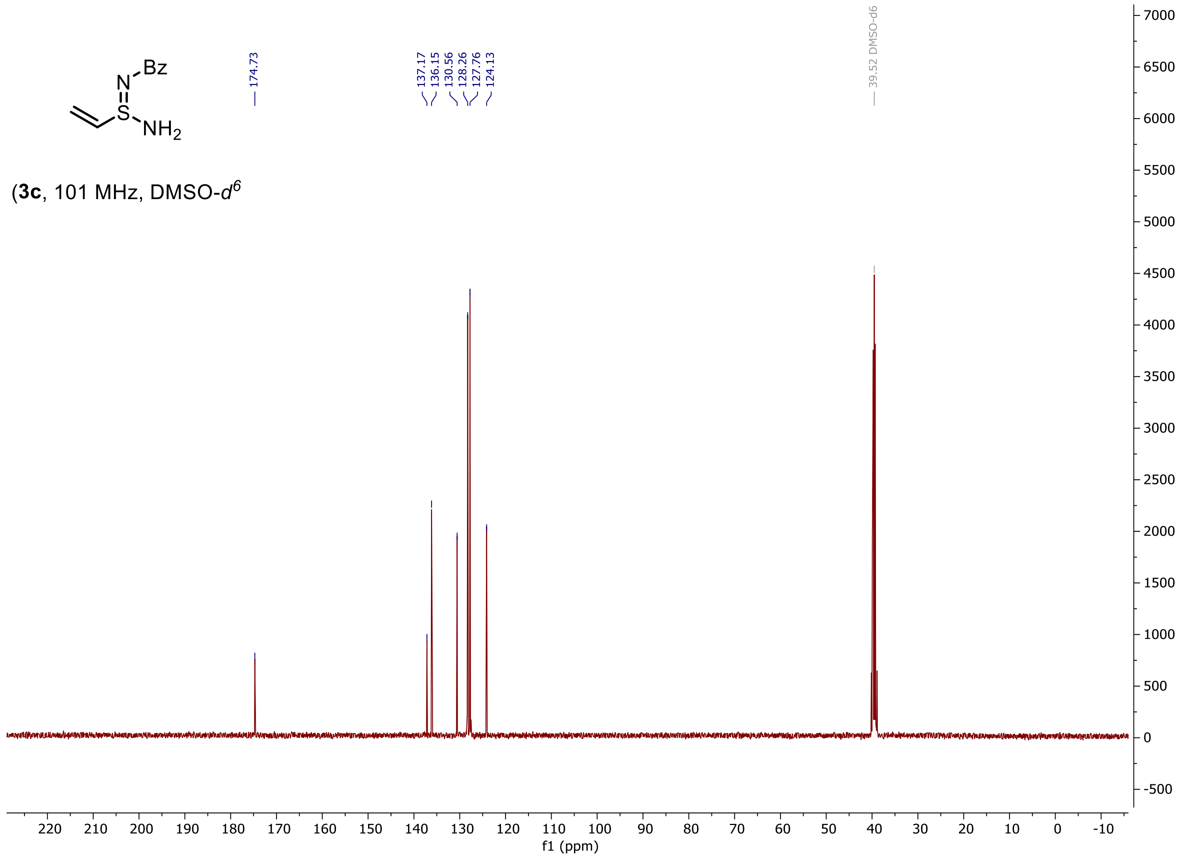

(3c, 101 MHz, DMSO-d6
174.73
137.17 136.15 130.56 128.26 127.76 124.13
39.52 DMSO-d6
f1 (ppm)

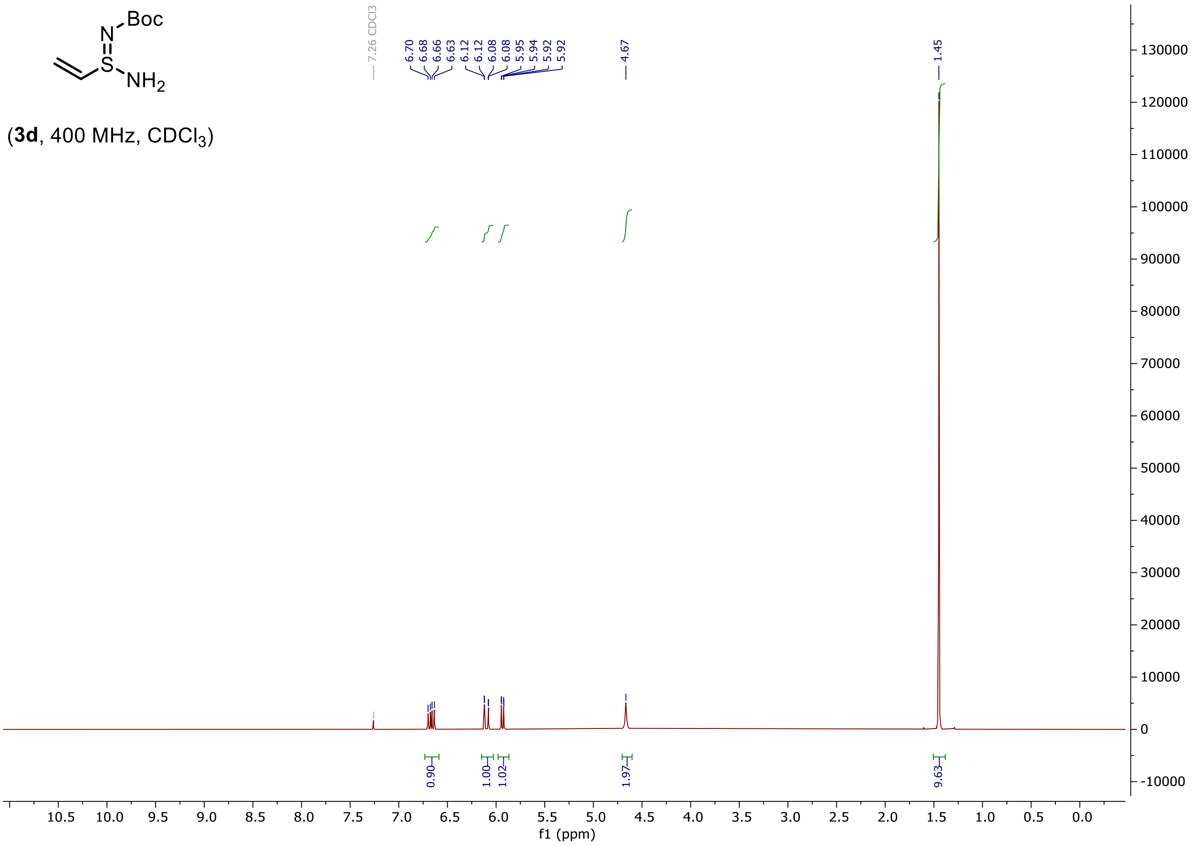
(3d, 400 MHz, CDCl3)

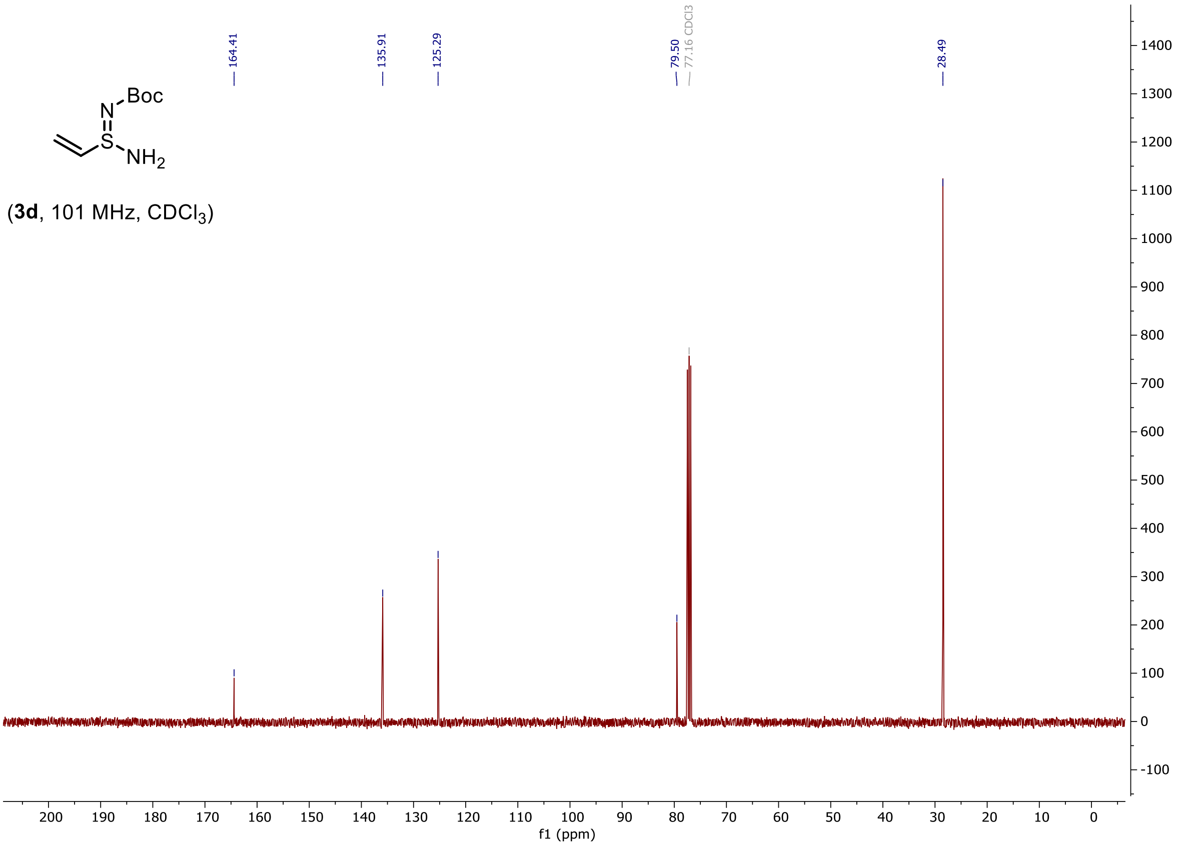
(3d, 101 MHz, CDCl3)

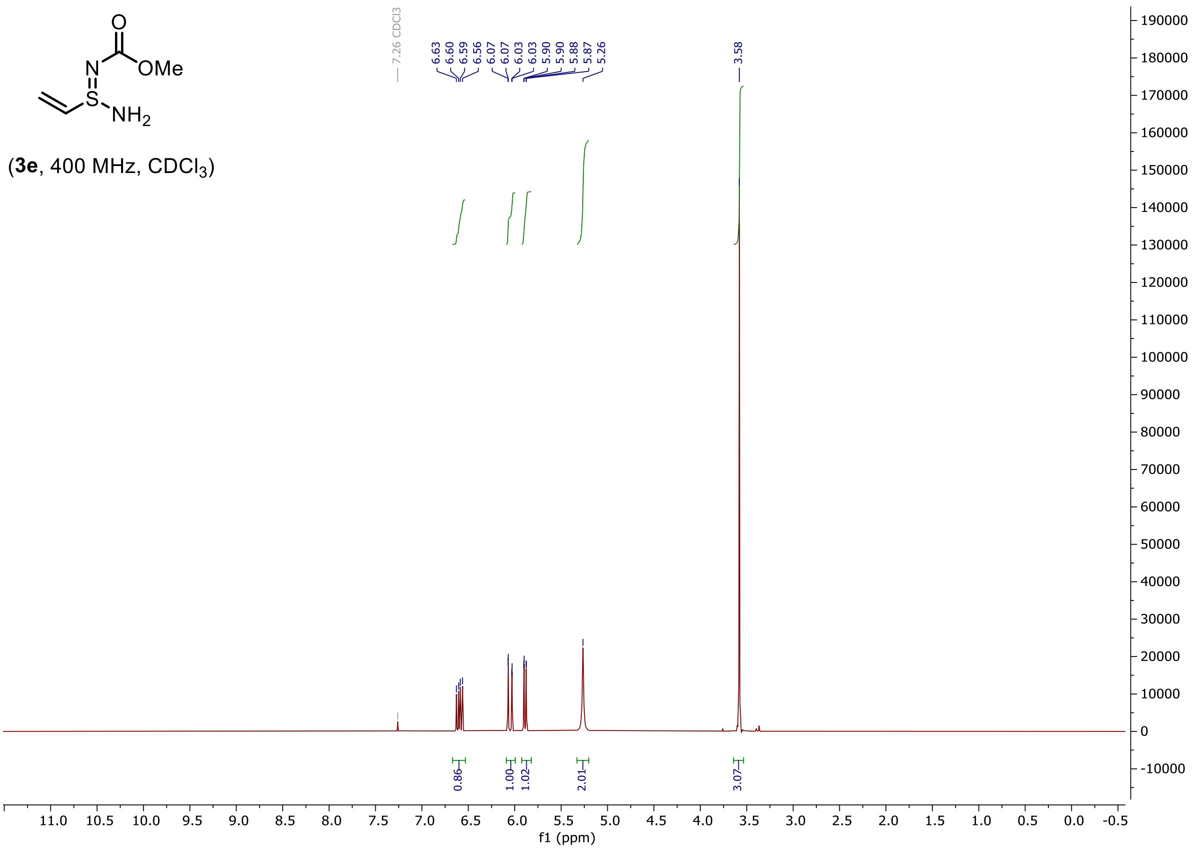
(3e, 400 MHz, CDCl3)
7.26 CDCl3
6.63
6.60
6.59
6.56
6.07
6.07
6.03
6.03
5.90
5.90
5.88
5.87
5.26
3.58
0.86
1.00
1.02
2.01
3.07
f1 (ppm)

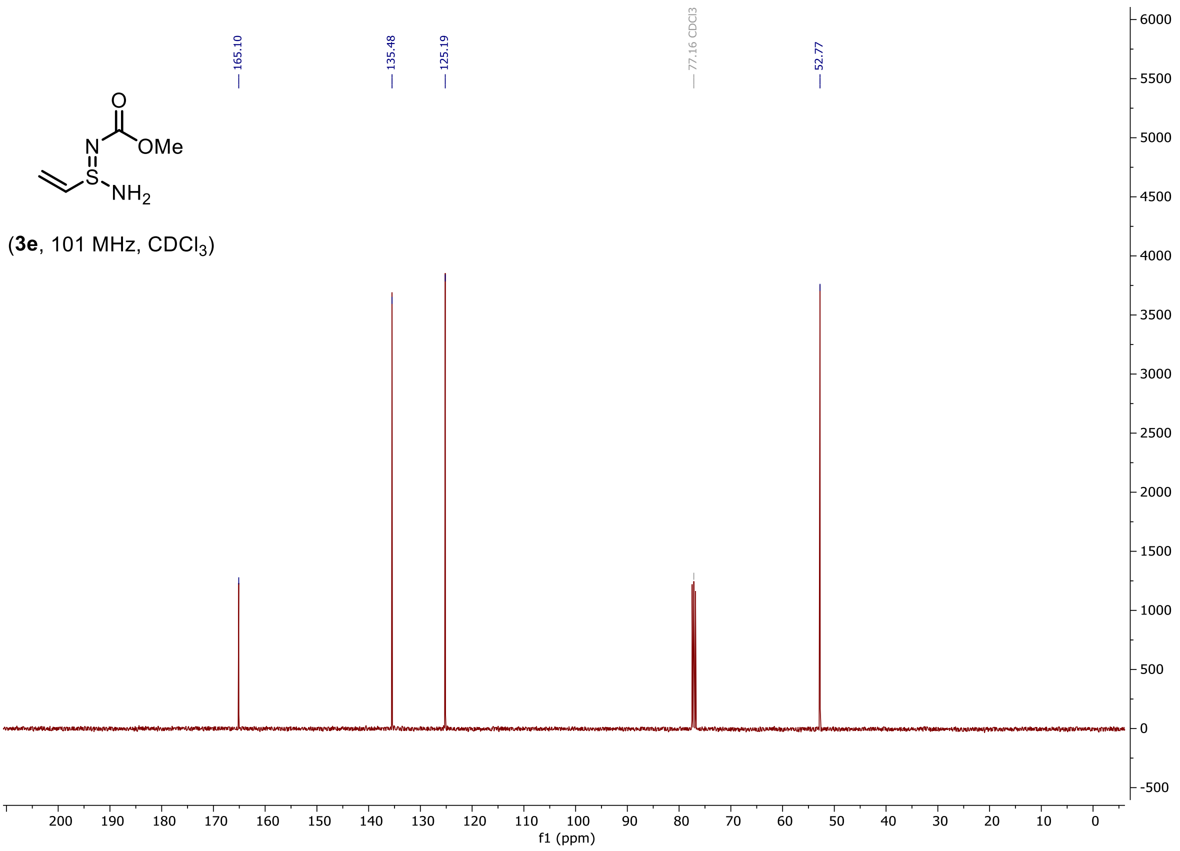
(3e, 101 MHz, CDCl3)
165.10
135.48
125.19
77.16 CDCl3
52.77
f1 (ppm)

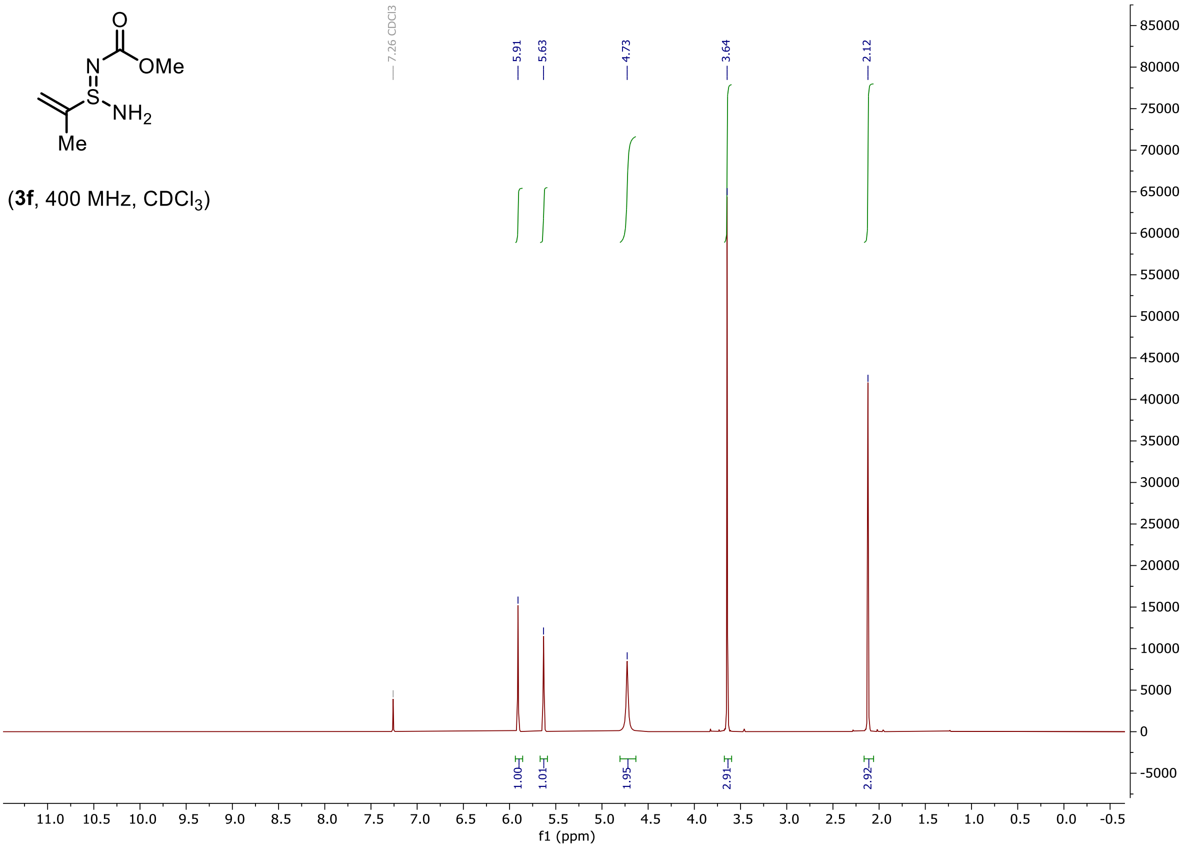

(**3f**, 400 MHz, $CDCl_3$)

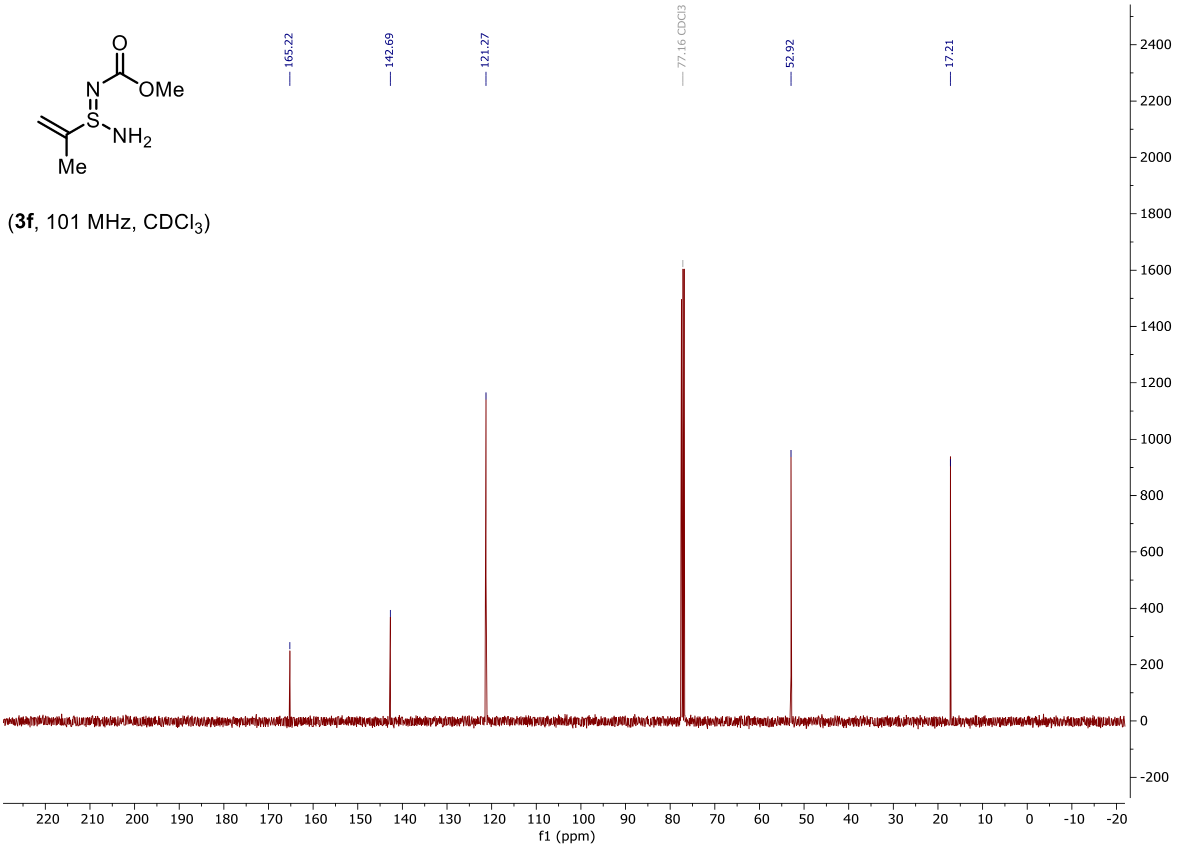

(**3f**, 101 MHz, $CDCl_3$)

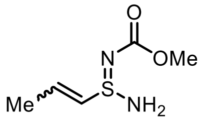

(**3g**, 400 MHz, $CDCl_3$)

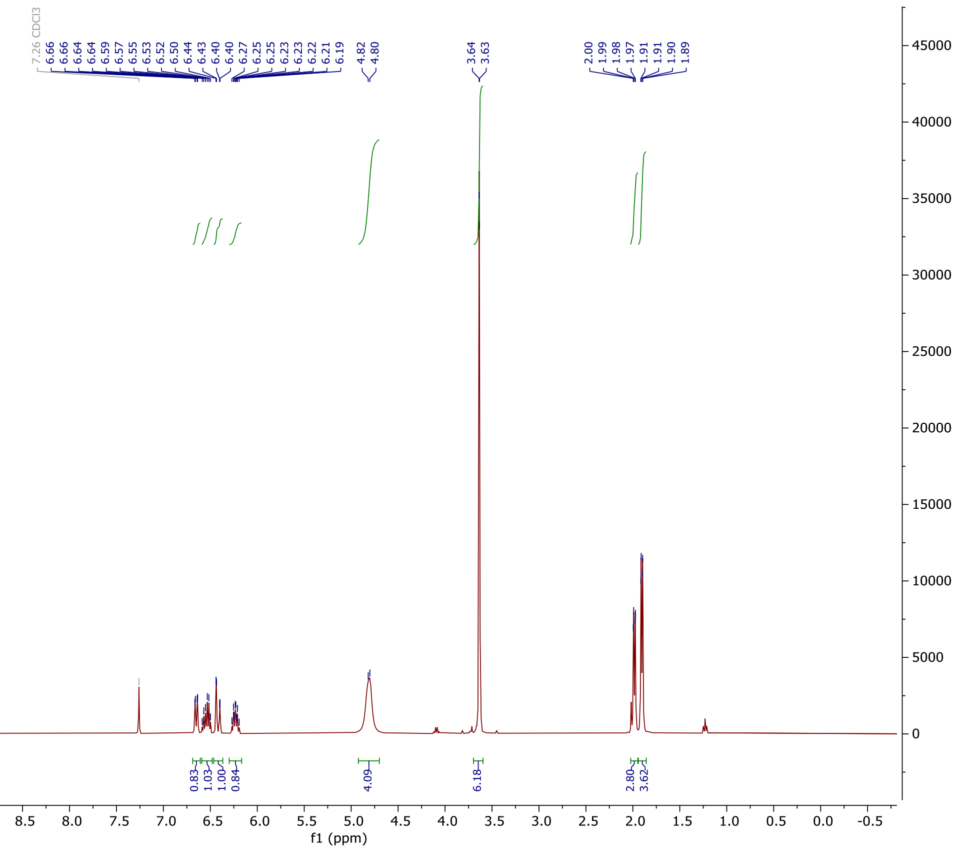

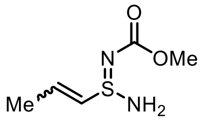

(**3g**, 101 MHz, $CDCl_3$)

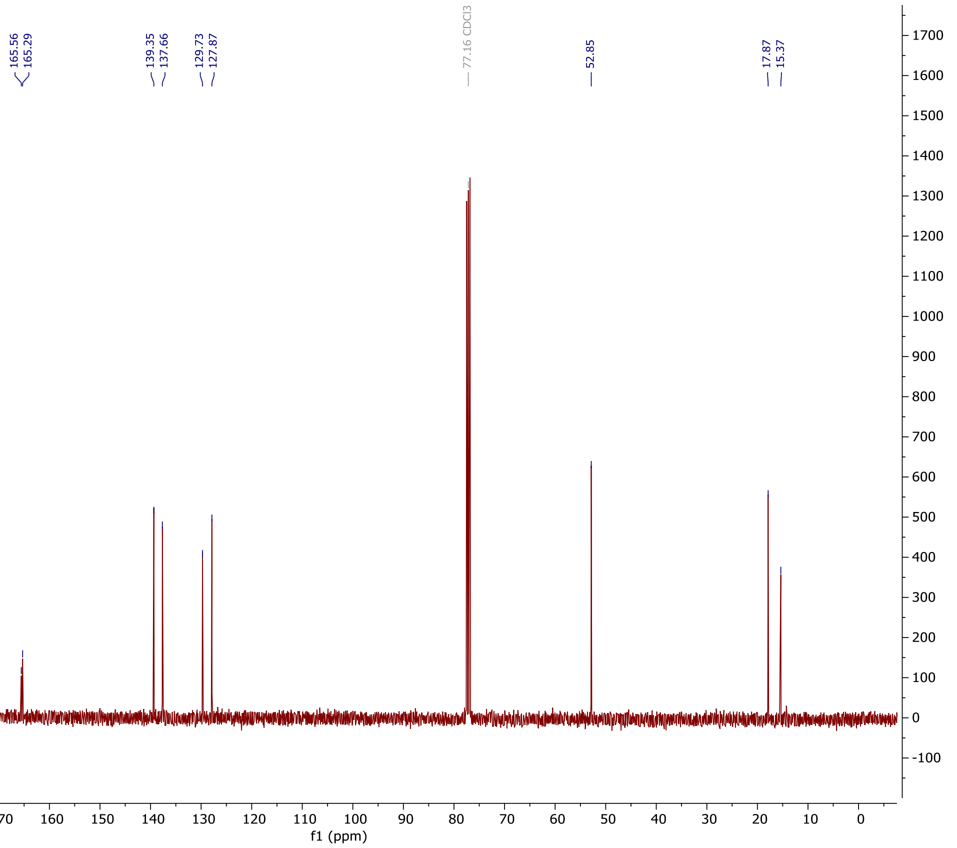

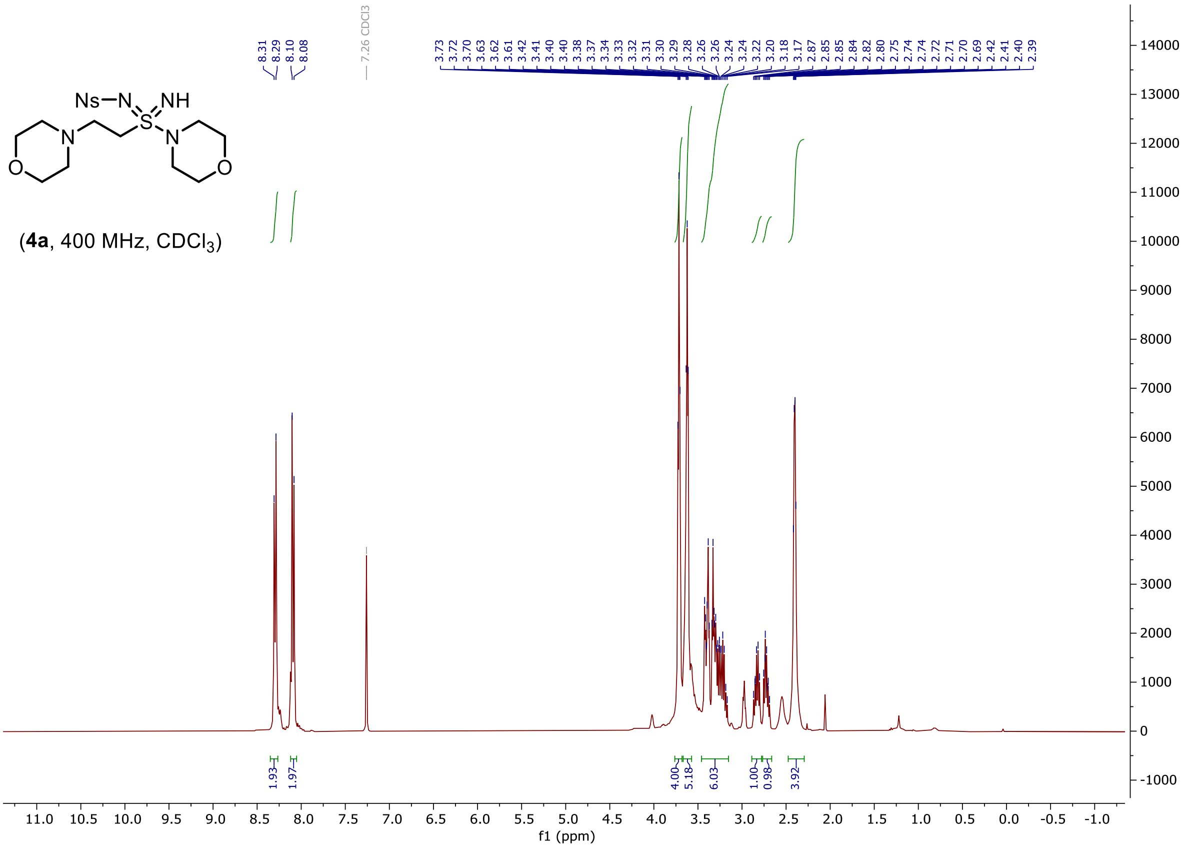
8.31
8.29
8.10
8.08
7.26 CDCl3
3.73 3.72 3.70 3.63 3.62 3.61 3.42 3.41 3.40 3.40 3.38 3.37 3.34 3.33 3.32 3.31 3.30 3.29 3.28 3.26 3.26 3.24 3.24 3.24 3.22 3.20 3.18 3.17 2.87 2.85 2.85 2.84 2.82 2.80 2.75 2.74 2.74 2.72 2.71 2.70 2.69 2.42 2.41 2.40 2.39
Ns–N
NH
S
N
O
(4a, 400 MHz, CDCl3)
1.93
1.97
4.00
5.18
6.03
1.00
0.98
3.92
f1 (ppm)

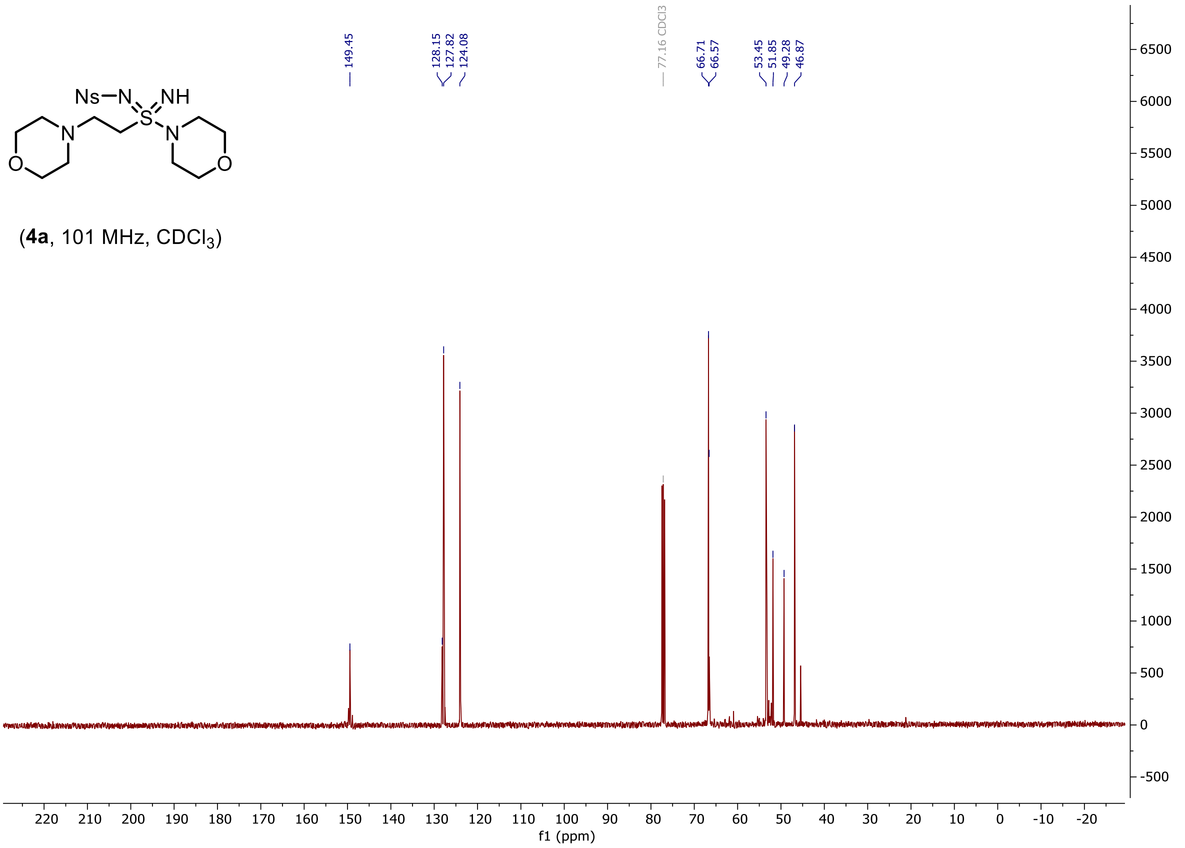
149.45
128.15
127.82
124.08
77.16 CDCl3
66.71
66.57
53.45
51.85
49.28
46.87
Ns–N
NH
S
N
O
(4a, 101 MHz, CDCl3)
f1 (ppm)

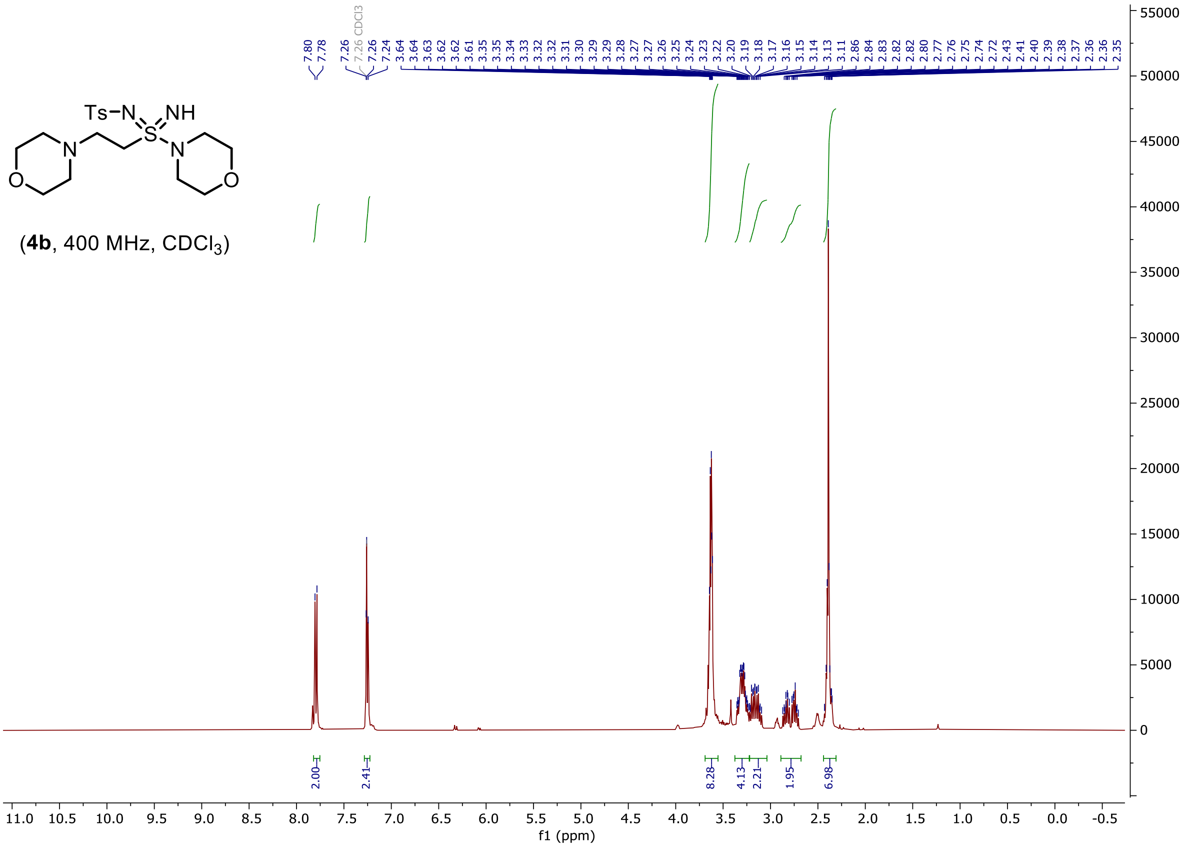

(**4b**, 400 MHz, $CDCl_3$)

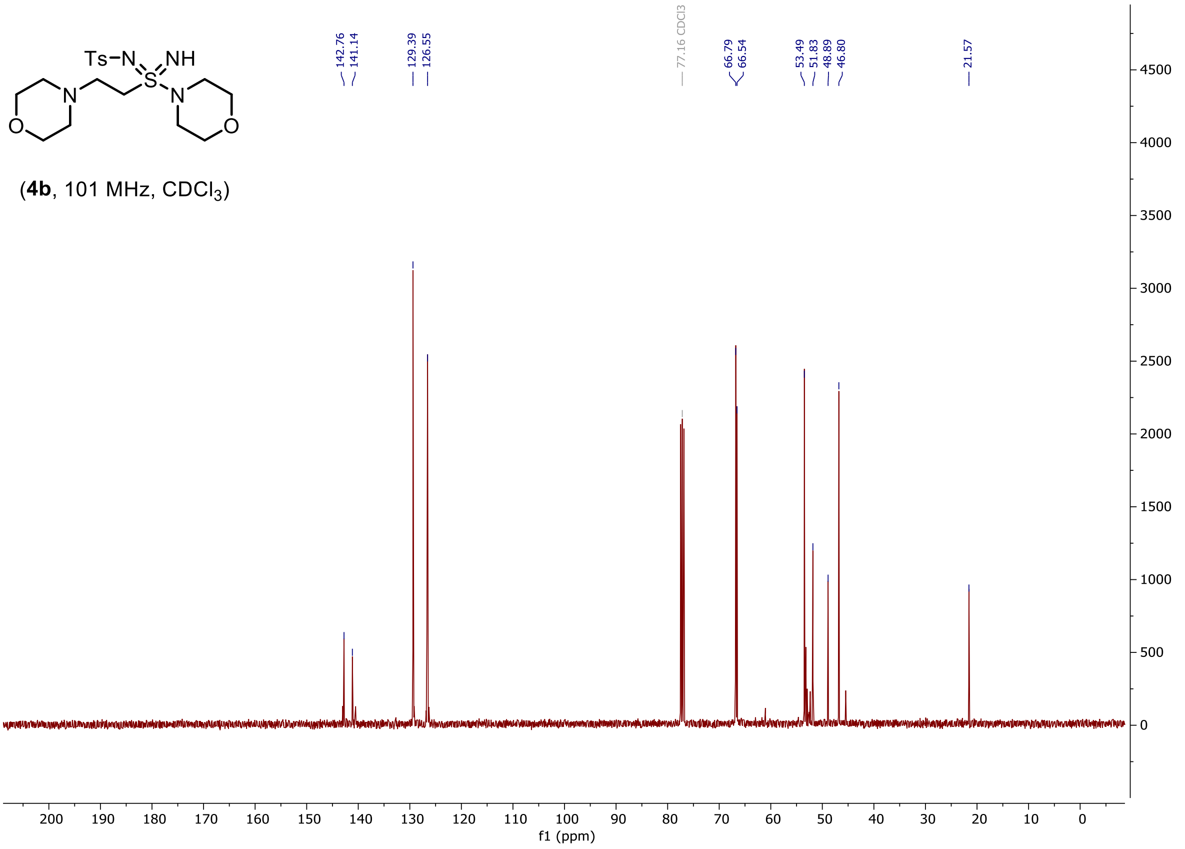

(**4b**, 101 MHz, $CDCl_3$)

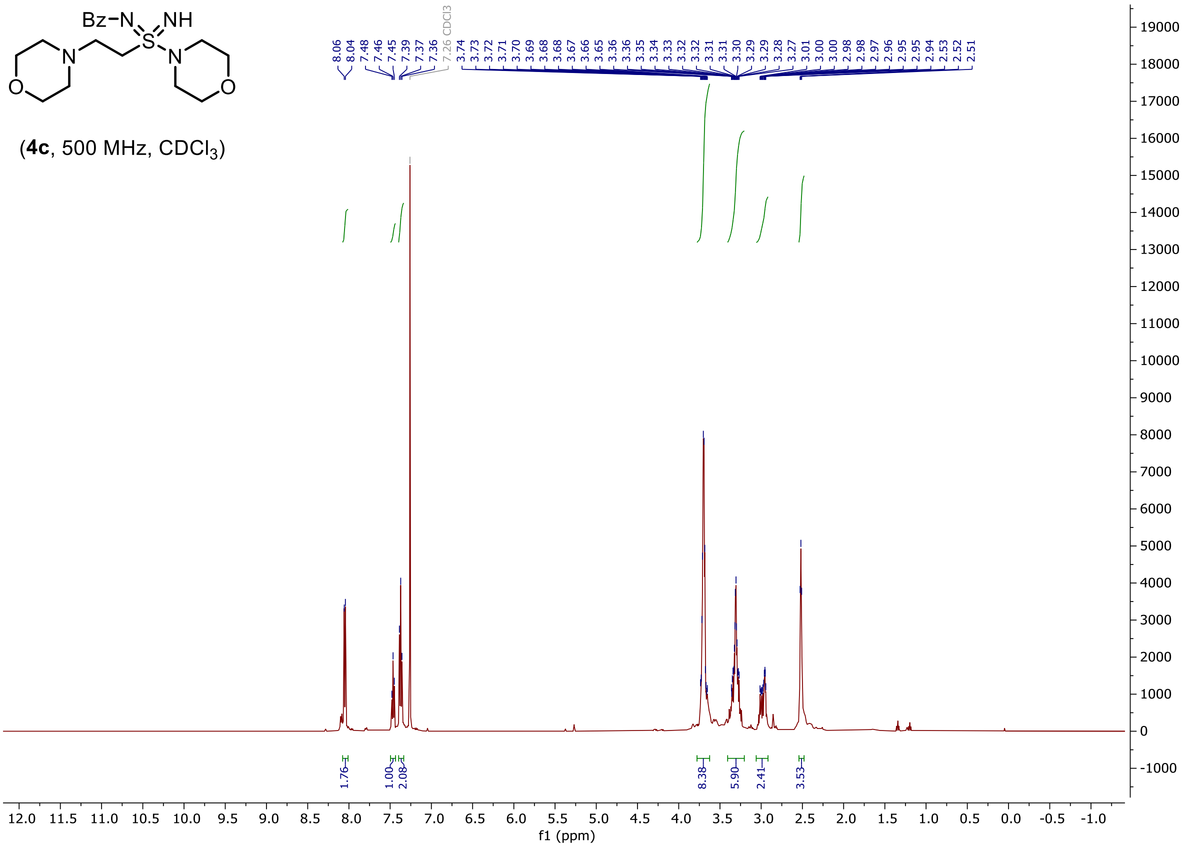

(**4c**, 500 MHz, $CDCl_3$)

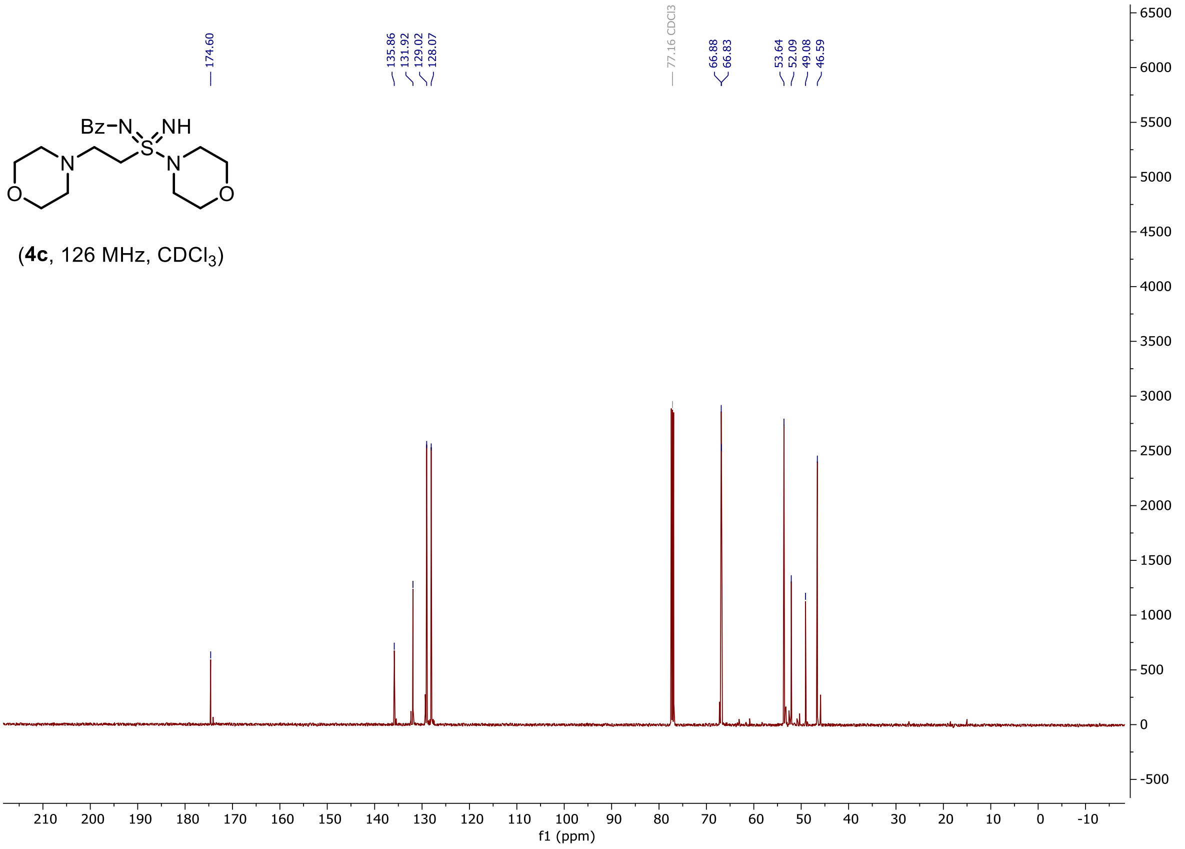

(**4c**, 126 MHz, $CDCl_3$)

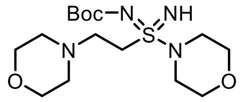

(**4d**, 400 MHz, $CDCl_3$)

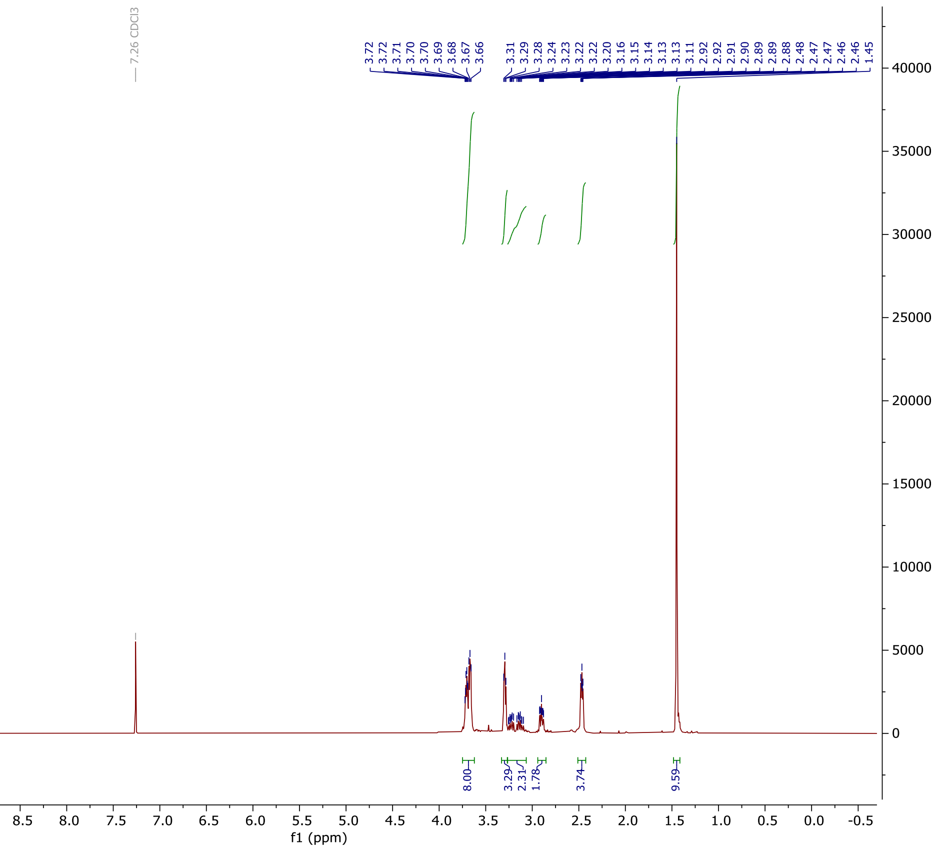

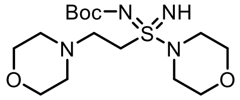

(**4d**, 101 MHz, $CDCl_3$)

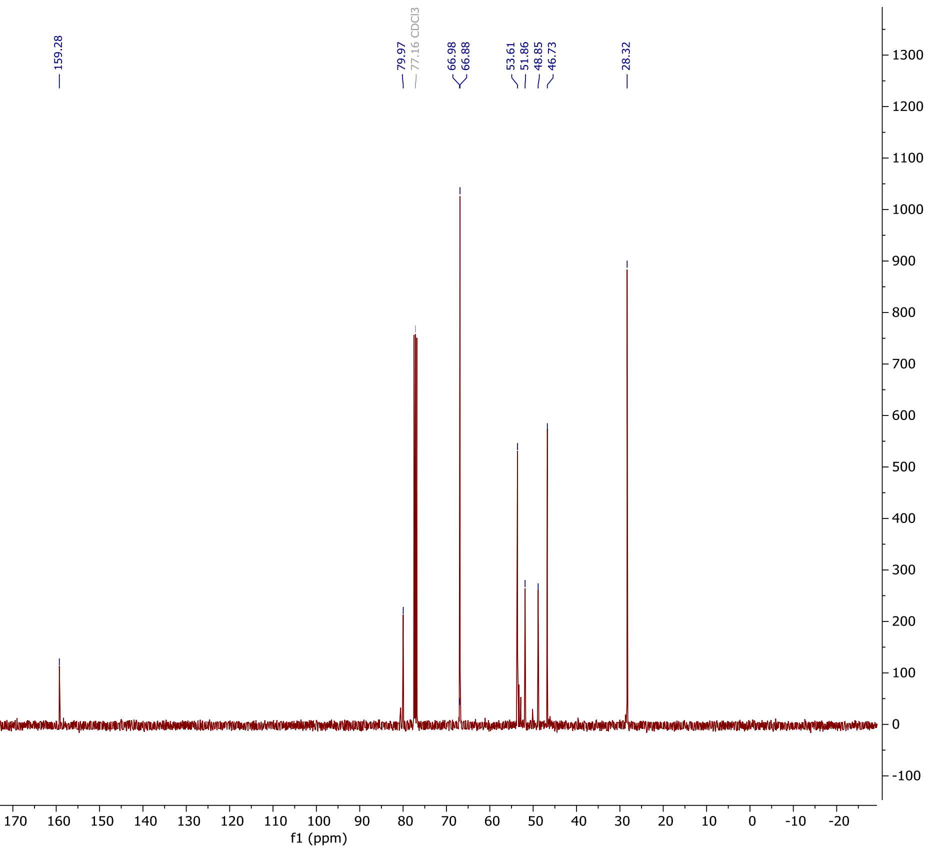

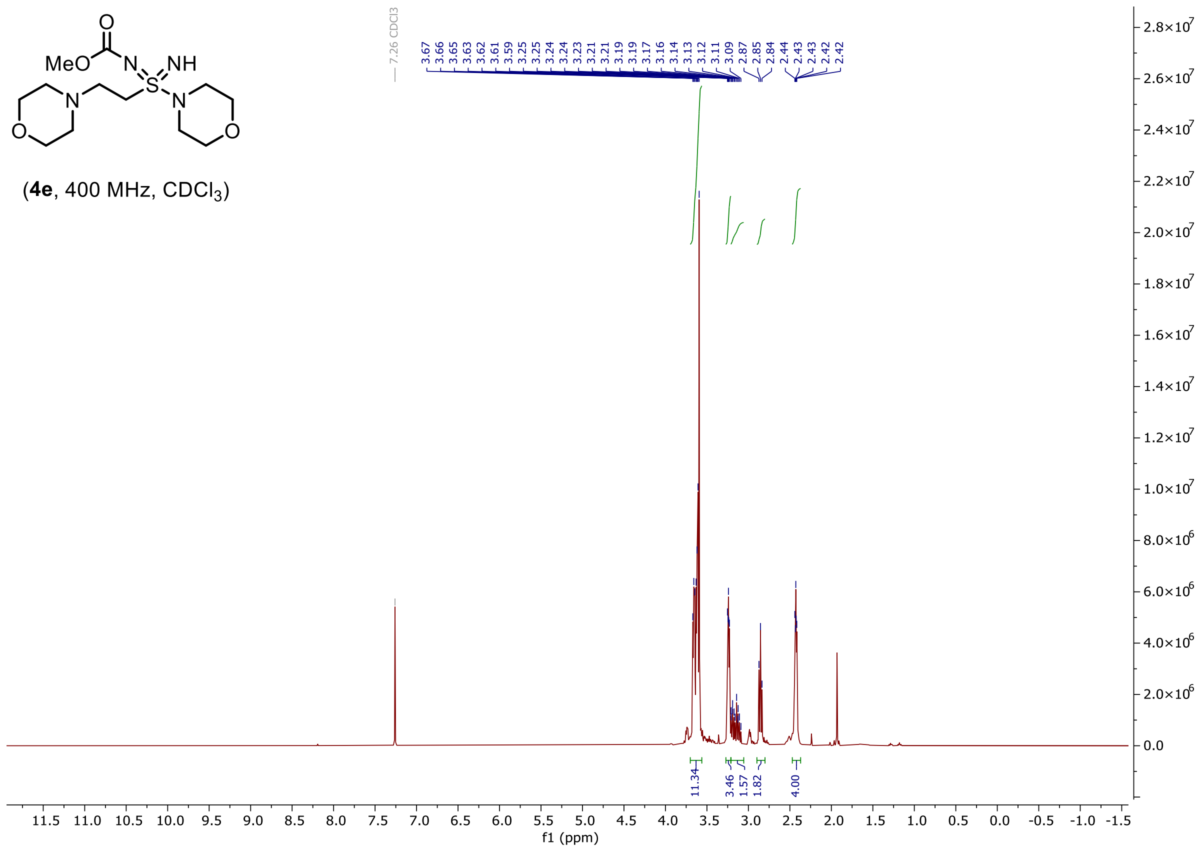

(**4e**, 400 MHz, $CDCl_3$)

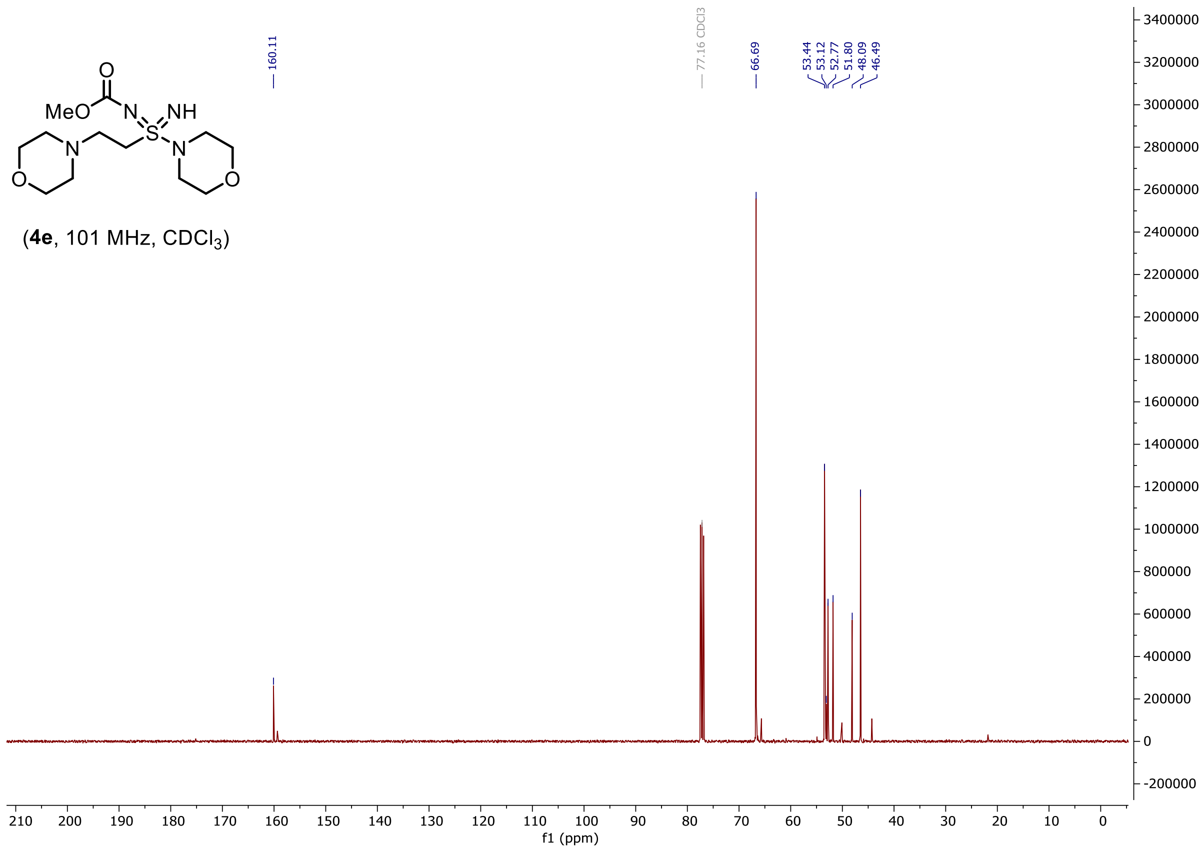

(**4e**, 101 MHz, $CDCl_3$)

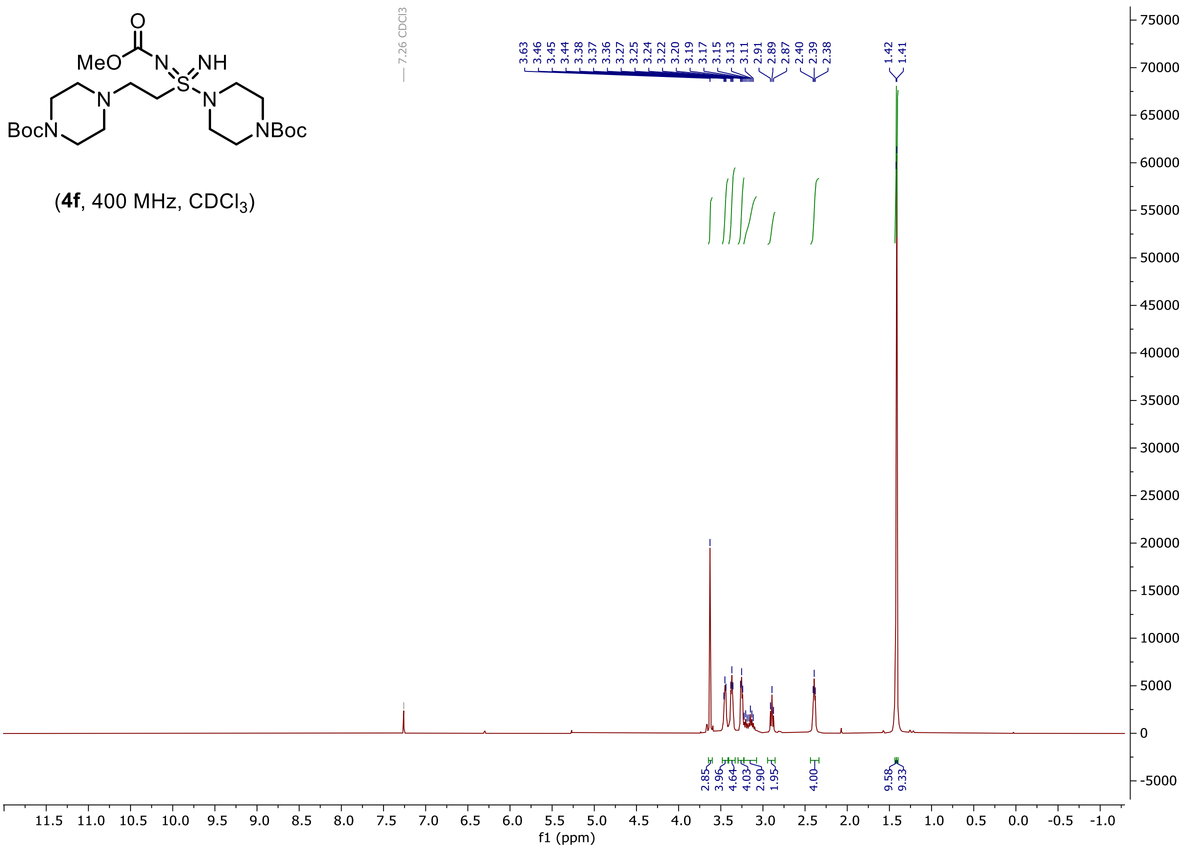

(**4f**, 400 MHz, $CDCl_3$)

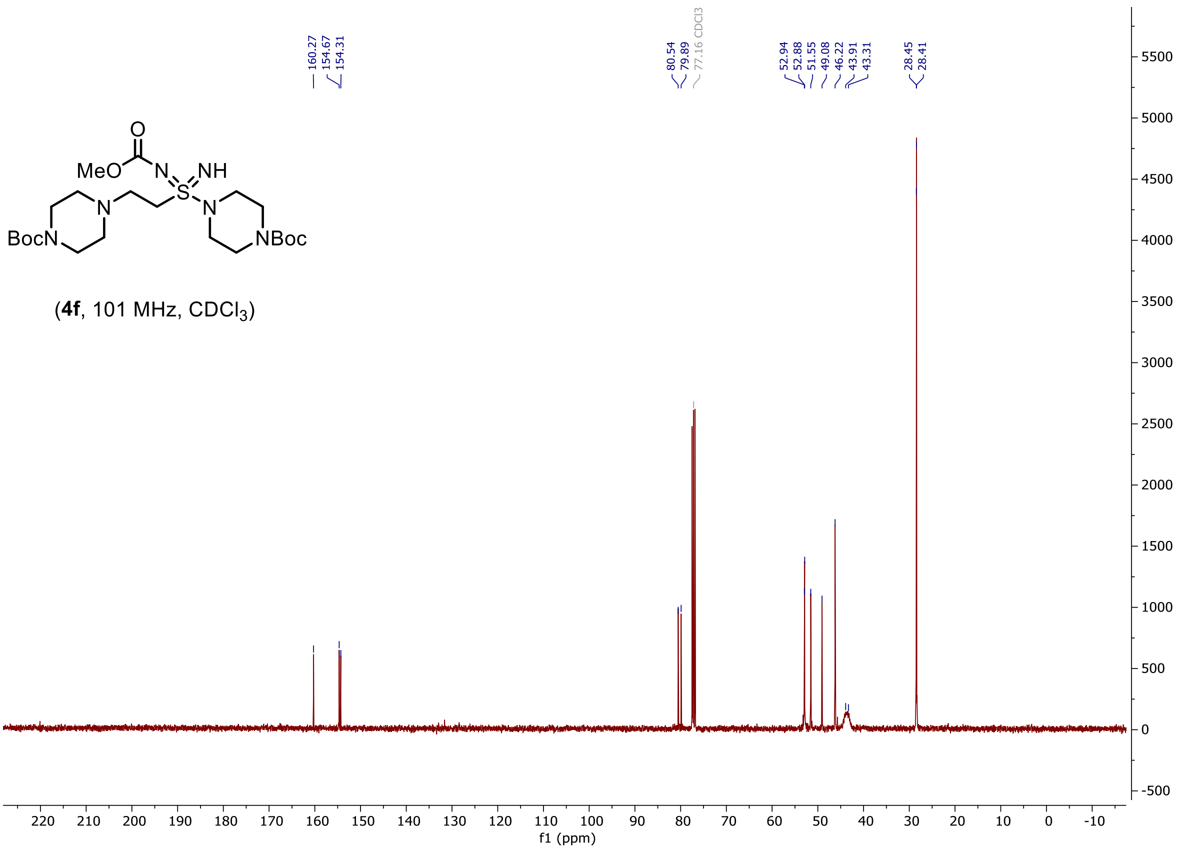

(**4f**, 101 MHz, $CDCl_3$)

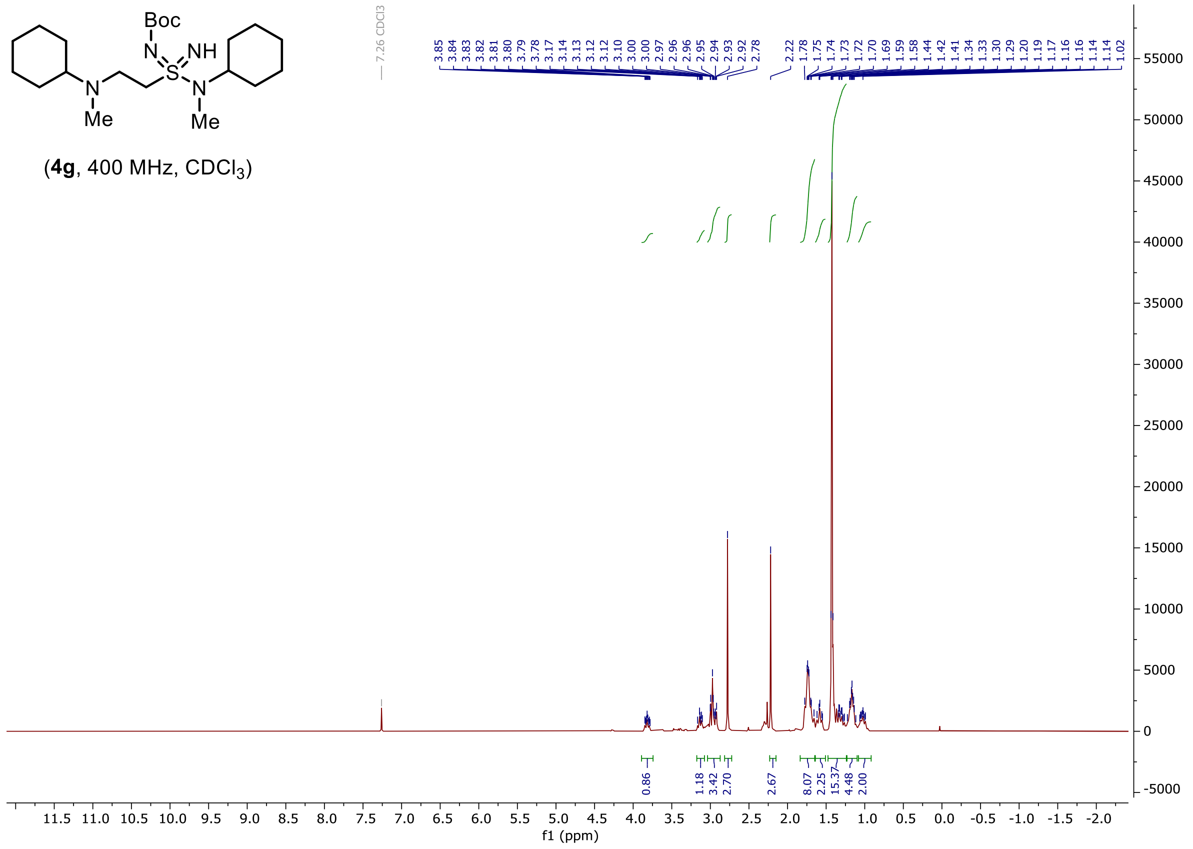

(**4g**, 400 MHz, $CDCl_3$)

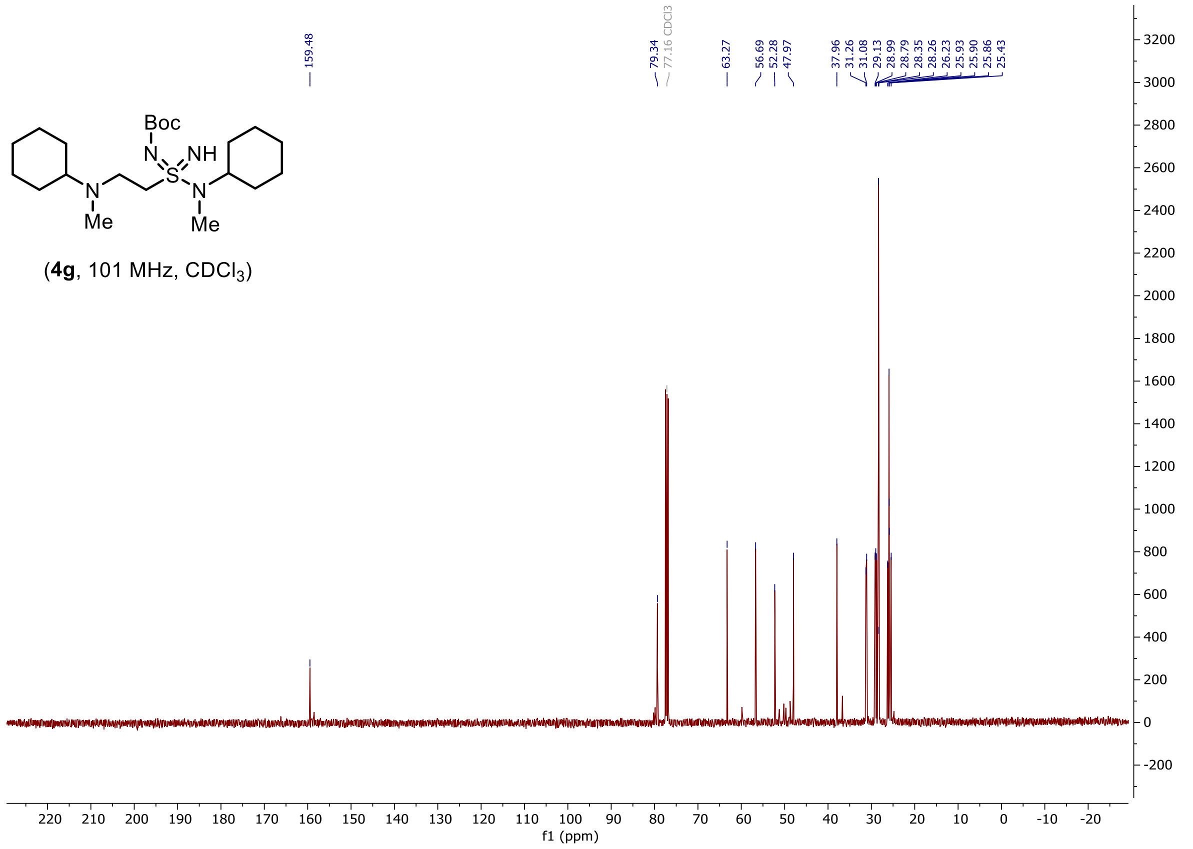

(**4g**, 101 MHz, $CDCl_3$)

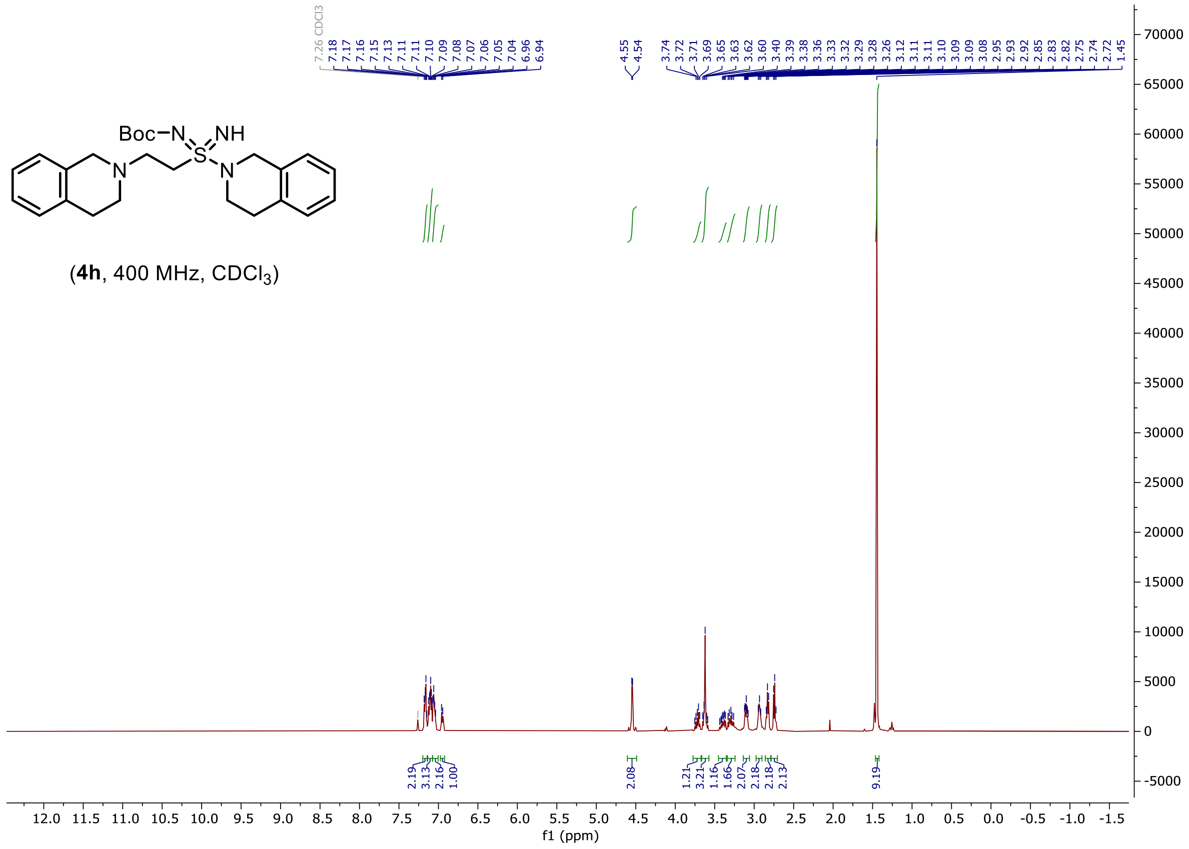
7.26 CDCl3
7.18 7.17 7.16 7.15 7.13 7.11 7.11 7.10 7.09 7.08 7.07 7.06 7.05 7.04 6.96 6.94
4.55 4.54
3.74 3.72 3.71 3.69 3.65 3.63 3.62 3.60 3.40 3.39 3.38 3.36 3.33 3.32 3.29 3.28 3.26 3.12 3.11 3.11 3.10 3.09 3.09 3.08 2.95 2.93 2.92 2.85 2.83 2.82 2.75 2.74 2.72 1.45
Boc−N
NH
S
N
N
(**4h**, 400 MHz, CDCl$_3$)
2.19 3.13 2.16 1.00
2.08
1.21 3.21 1.16 1.66 2.07 2.18 2.18 2.13
9.19
f1 (ppm)

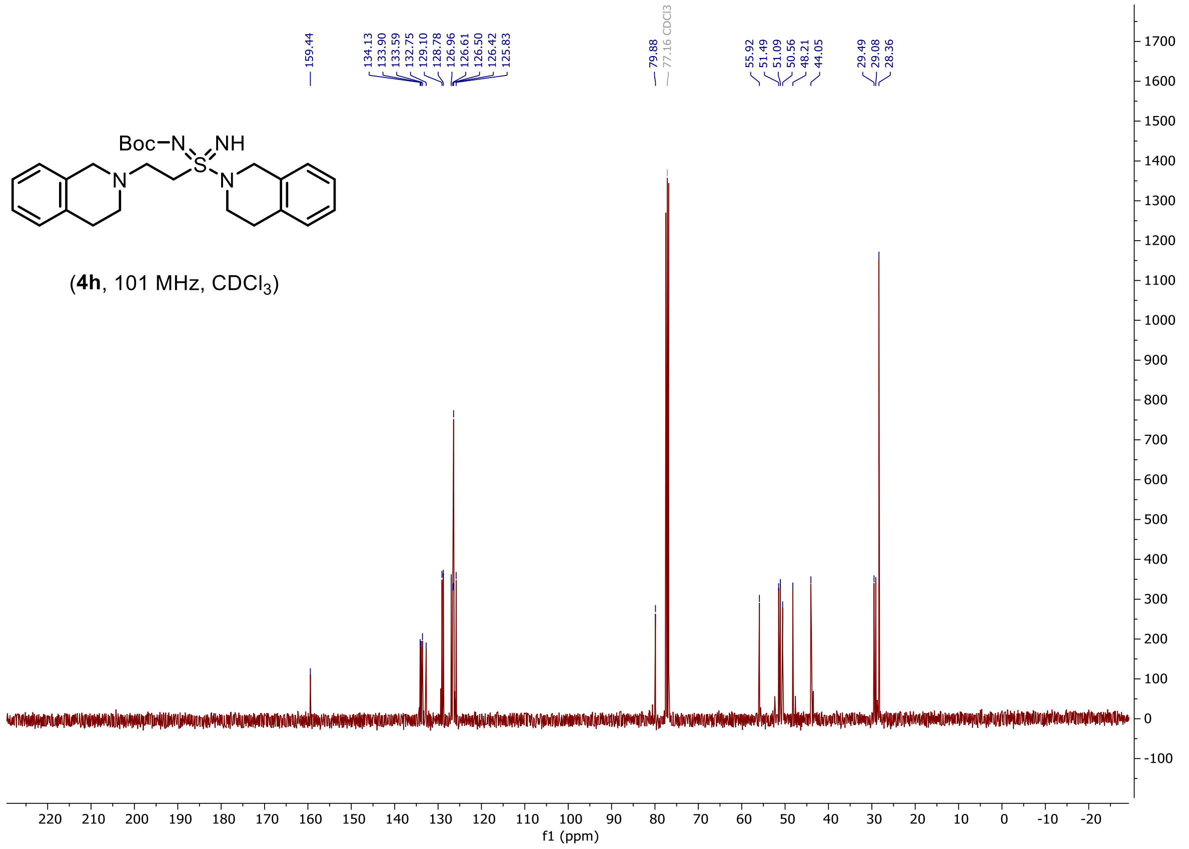
159.44
134.13 133.90 133.59 132.75 129.10 128.78 126.96 126.61 126.50 126.42 125.83
79.88
77.16 CDCl3
55.92 51.49 51.09 50.56 48.21 44.05
29.49 29.08 28.36
Boc−N
NH
S
N
N
(**4h**, 101 MHz, CDCl$_3$)
f1 (ppm)

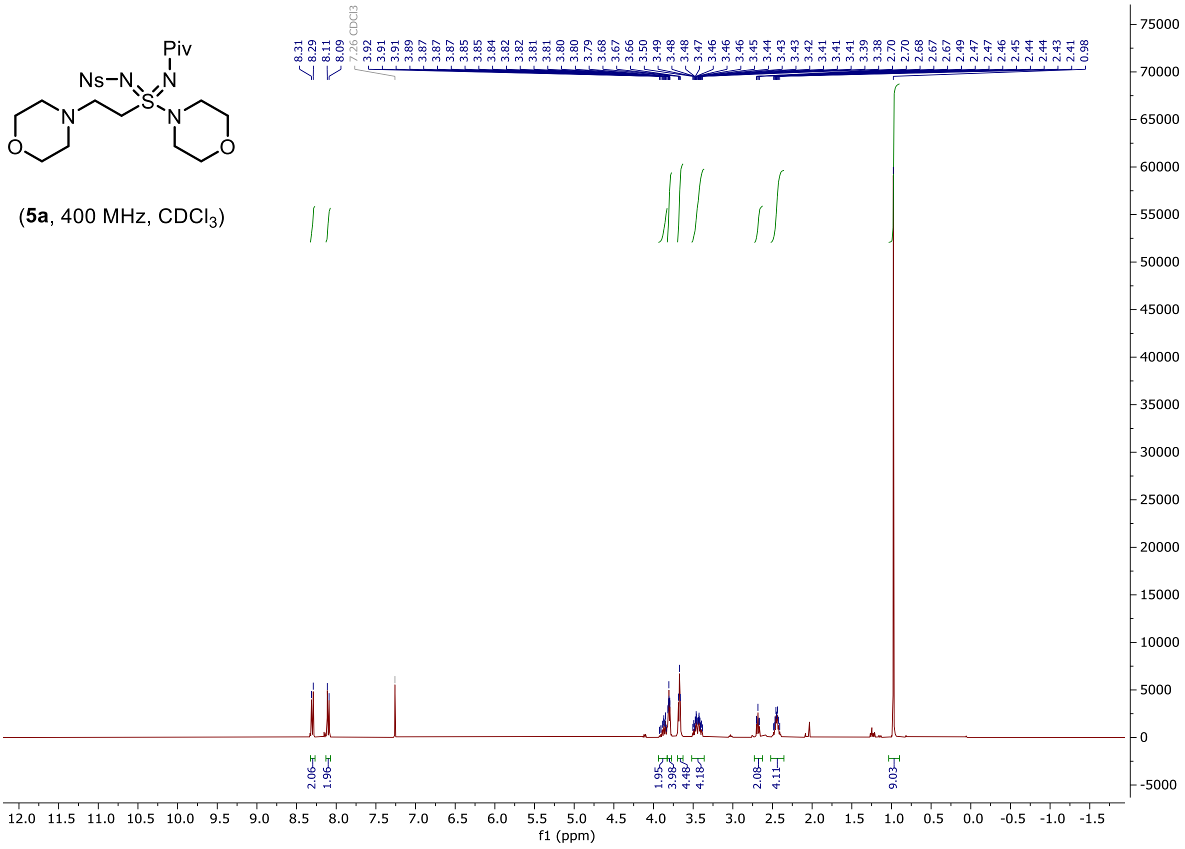

(**5a**, 400 MHz, $CDCl_3$)

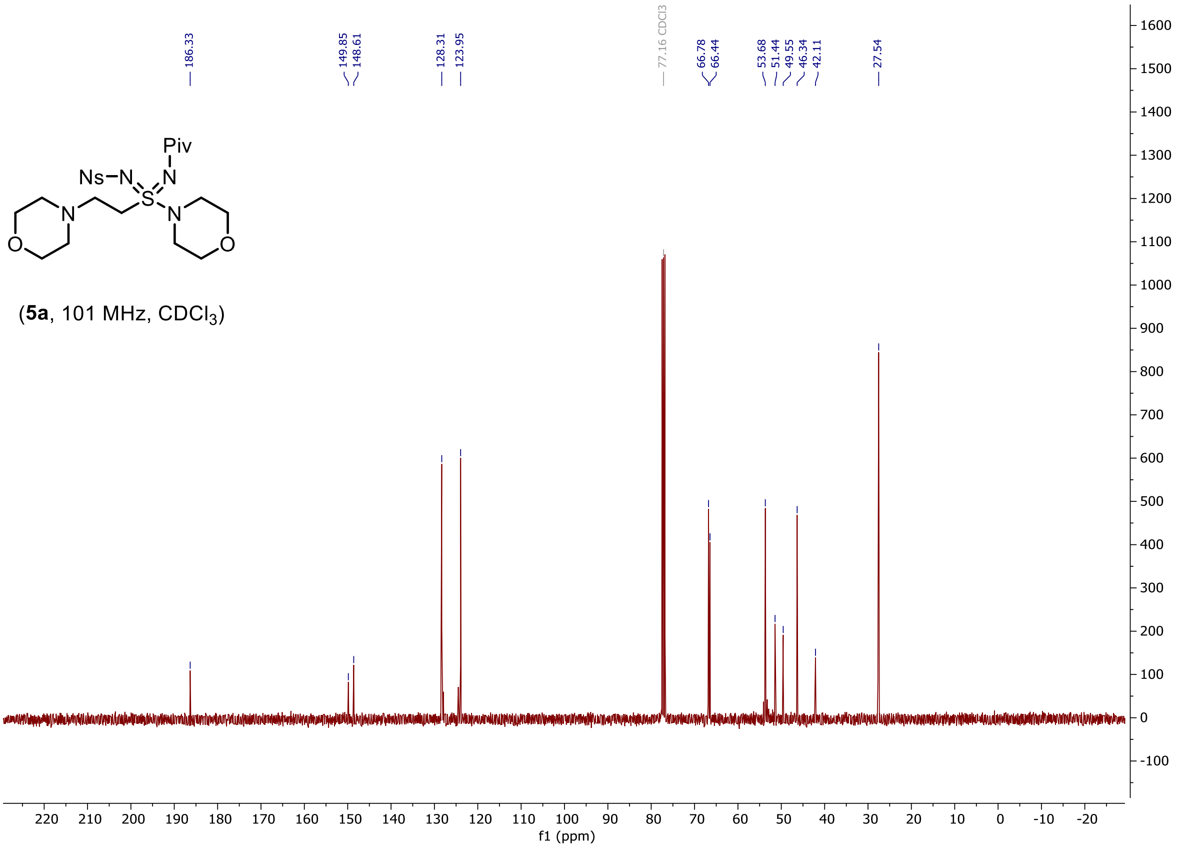

(**5a**, 101 MHz, $CDCl_3$)

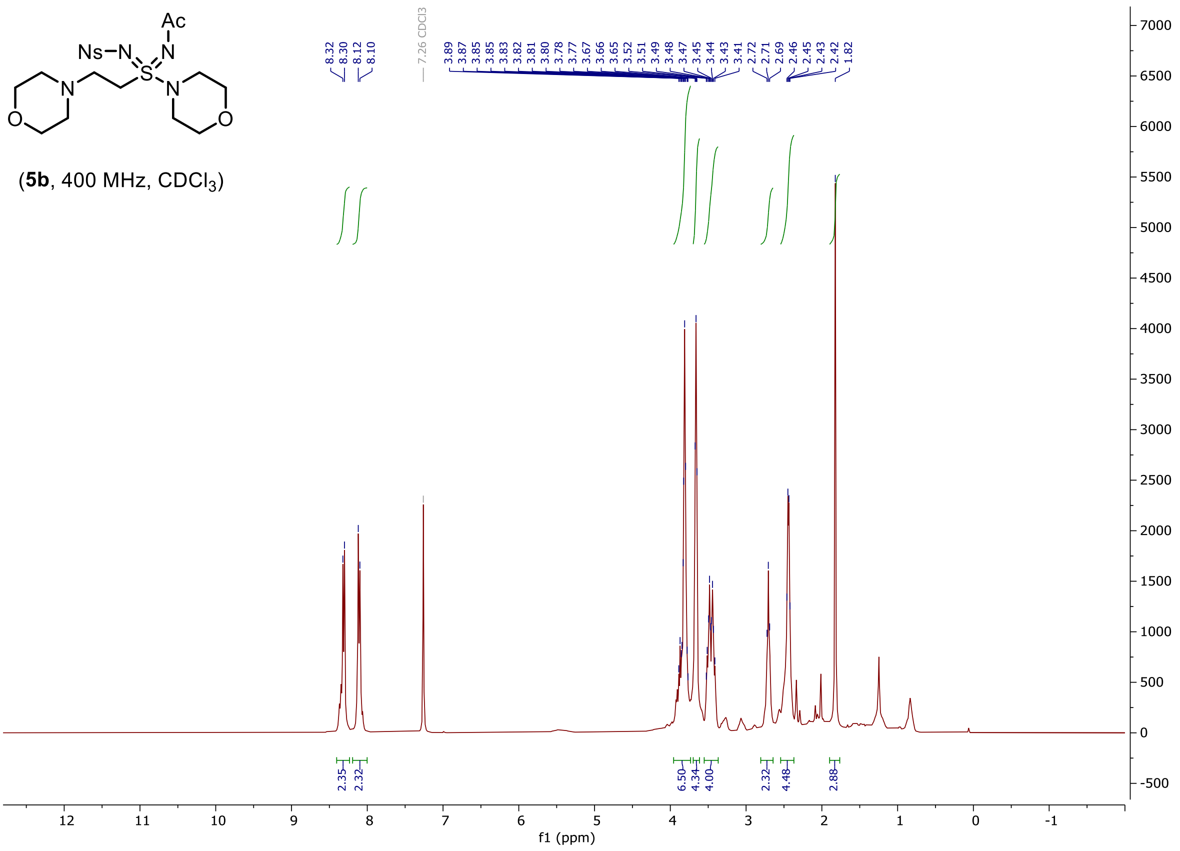

(**5b**, 400 MHz, $CDCl_3$)

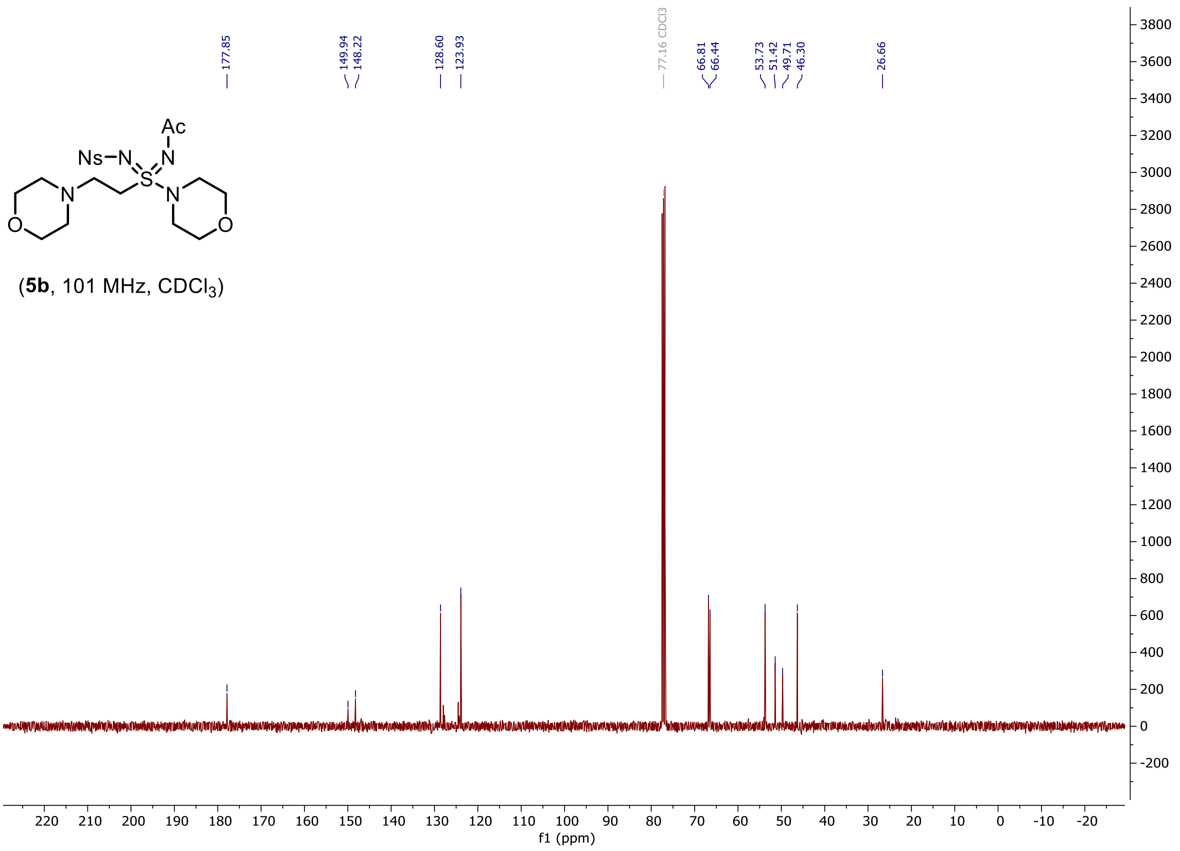

(**5b**, 101 MHz, $CDCl_3$)

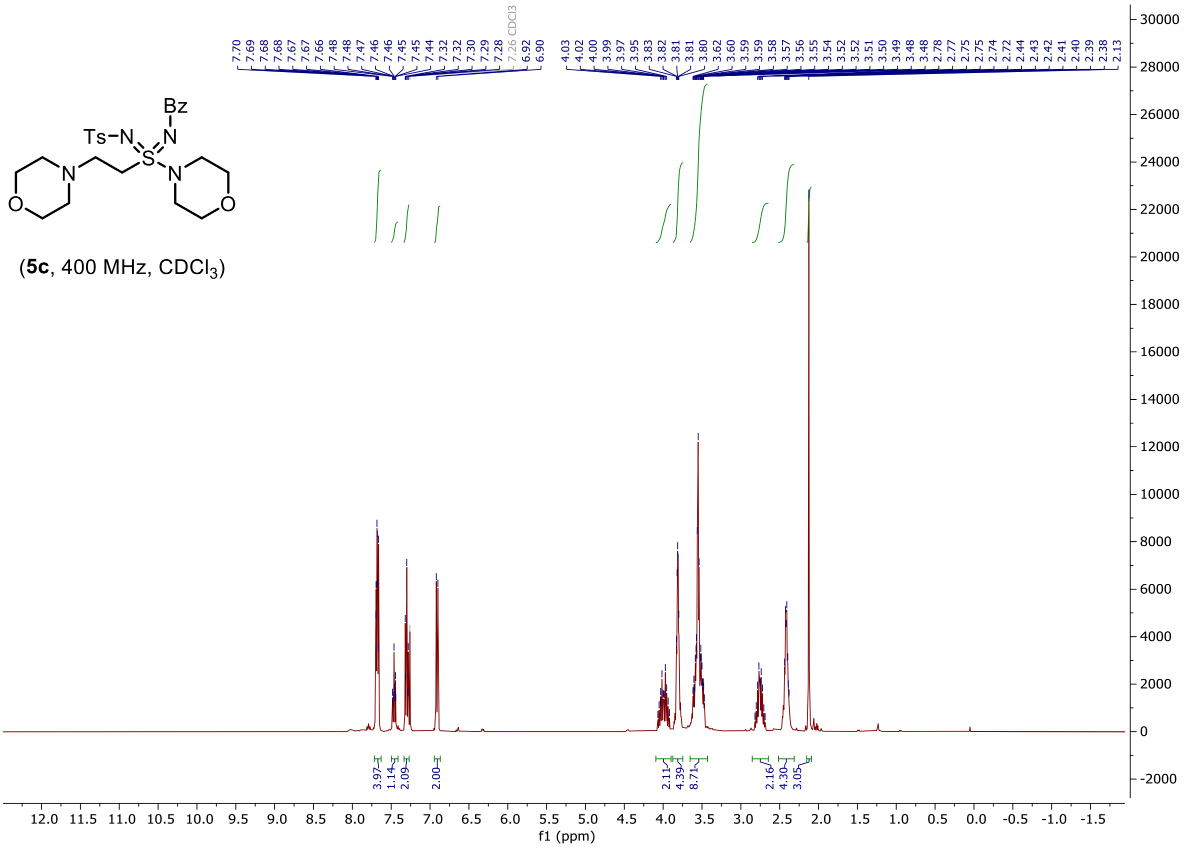

(**5c**, 400 MHz, $CDCl_3$)

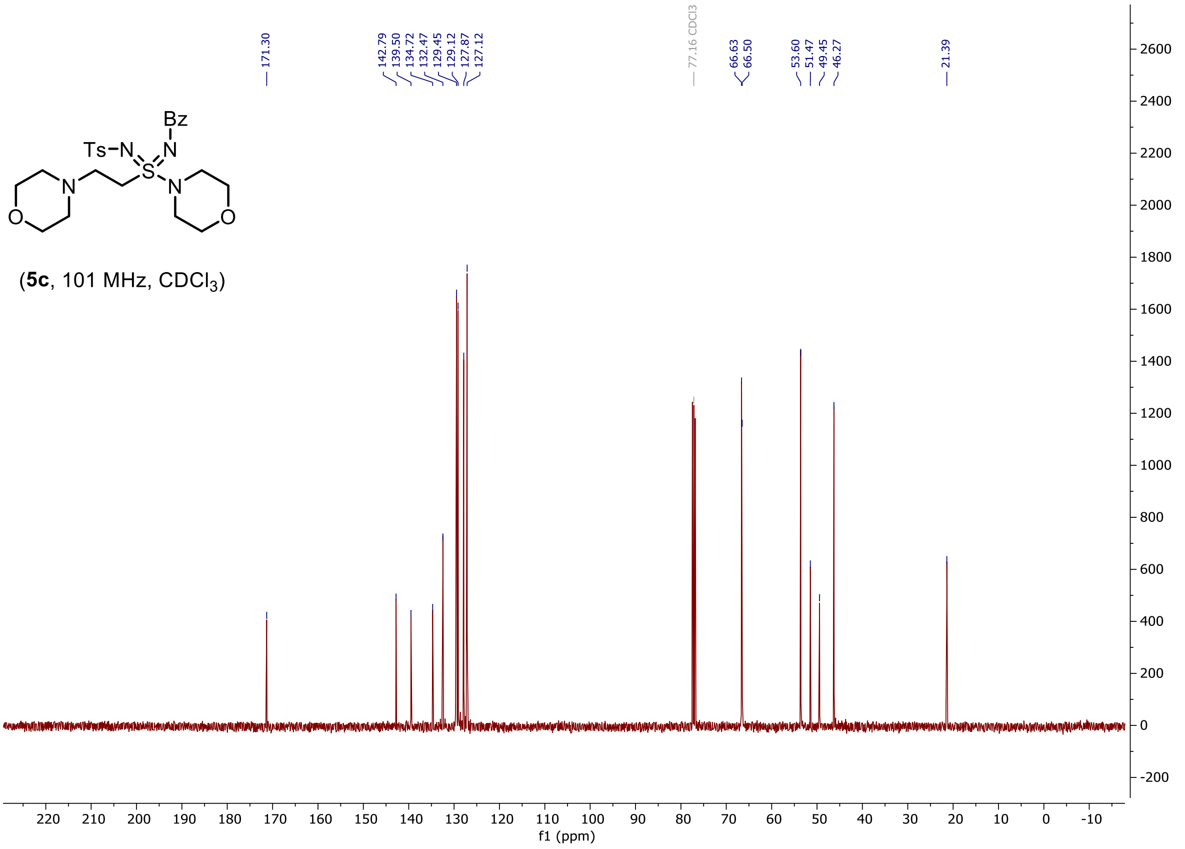

(**5c**, 101 MHz, $CDCl_3$)

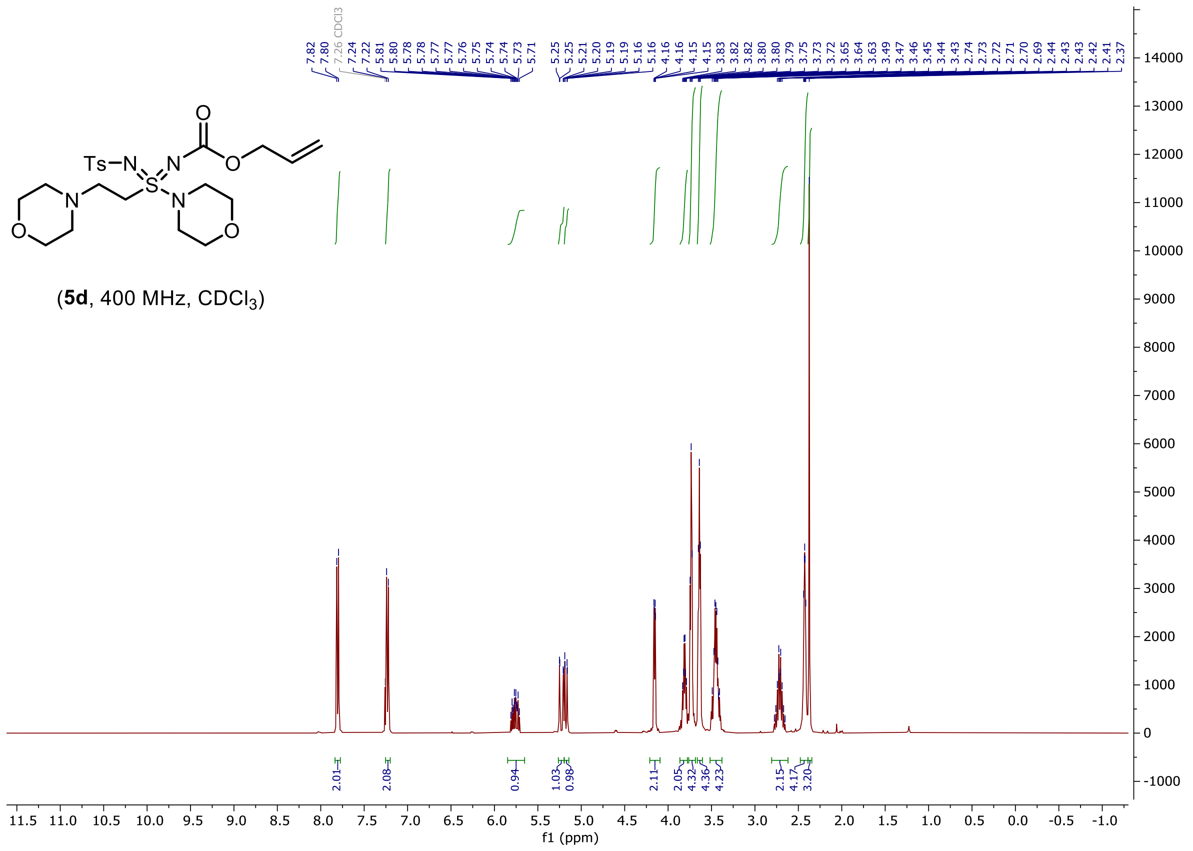
(5d, 400 MHz, CDCl3)
f1 (ppm)

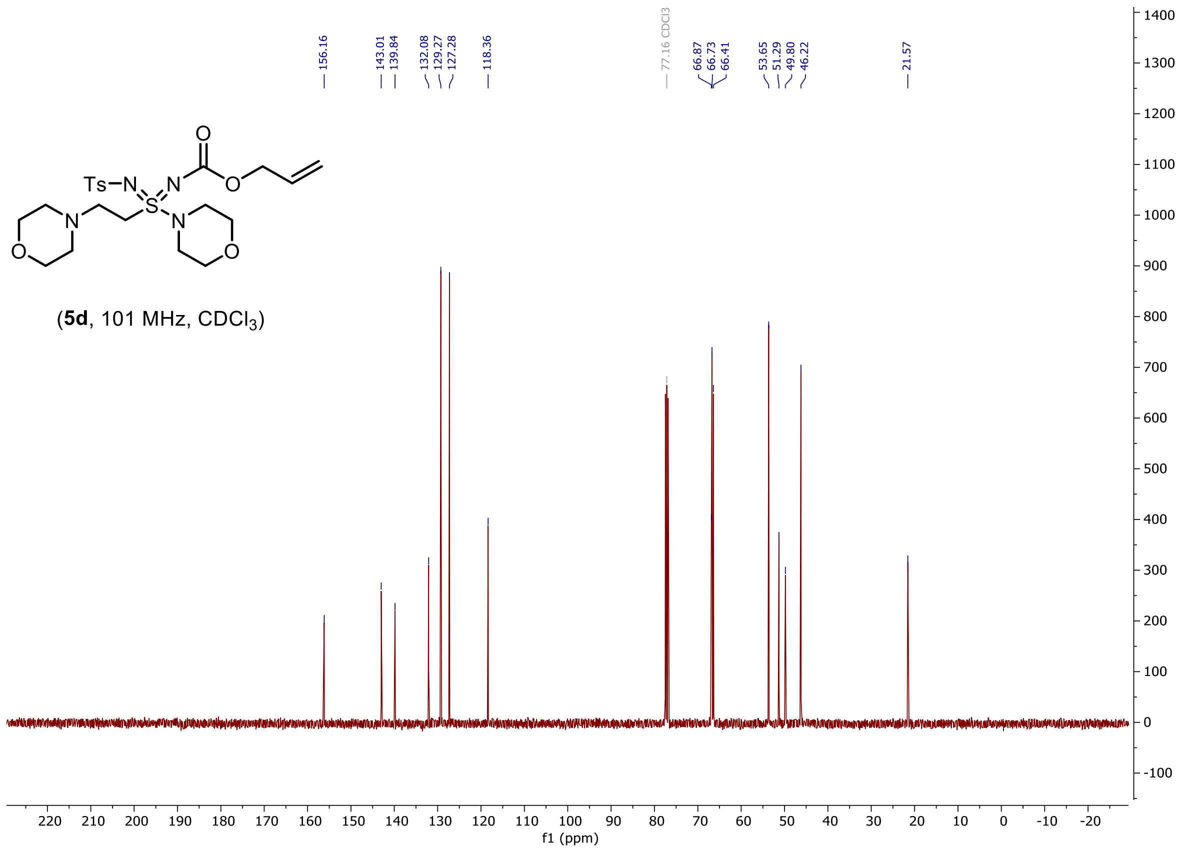
(5d, 101 MHz, CDCl3)
156.16
143.01
139.84
132.08
129.27
127.28
118.36
77.16 CDCl3
66.87
66.73
66.41
53.65
51.29
49.80
46.22
21.57
f1 (ppm)

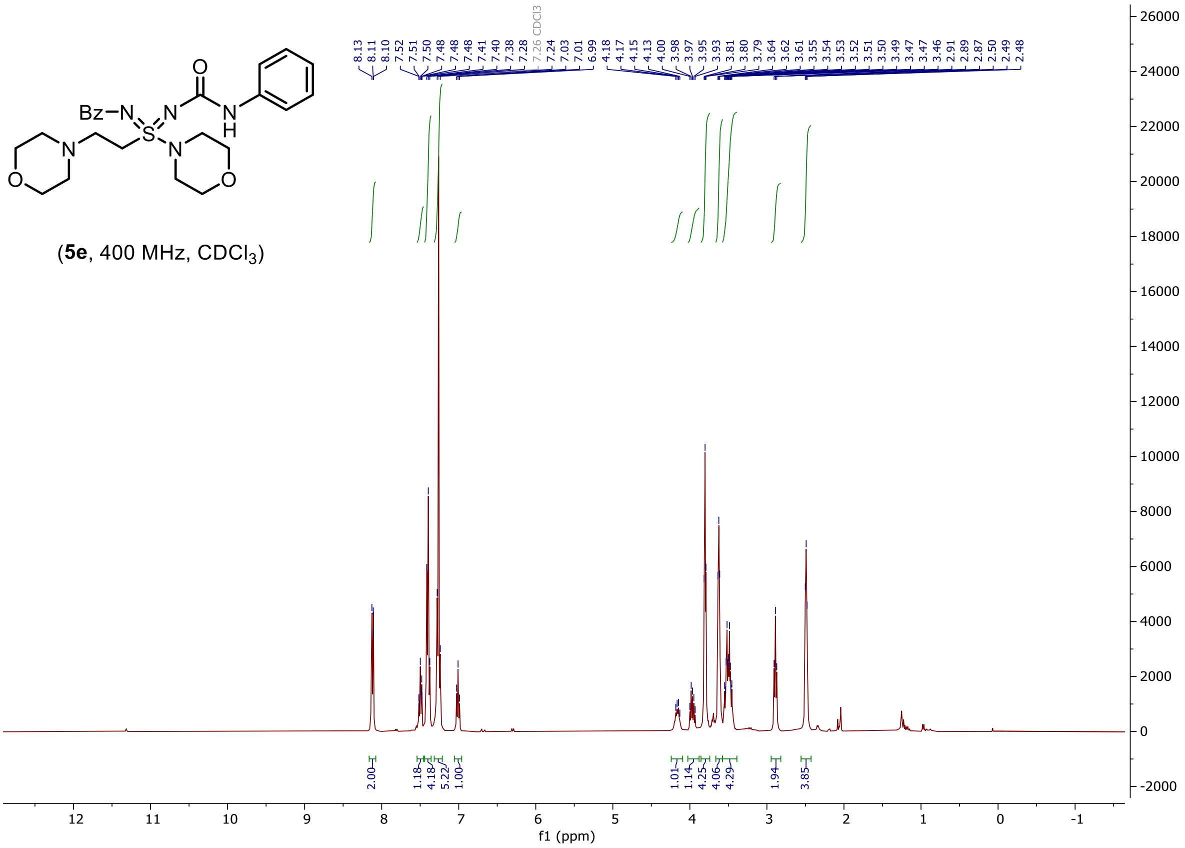

(**5e**, 400 MHz, $CDCl_3$)

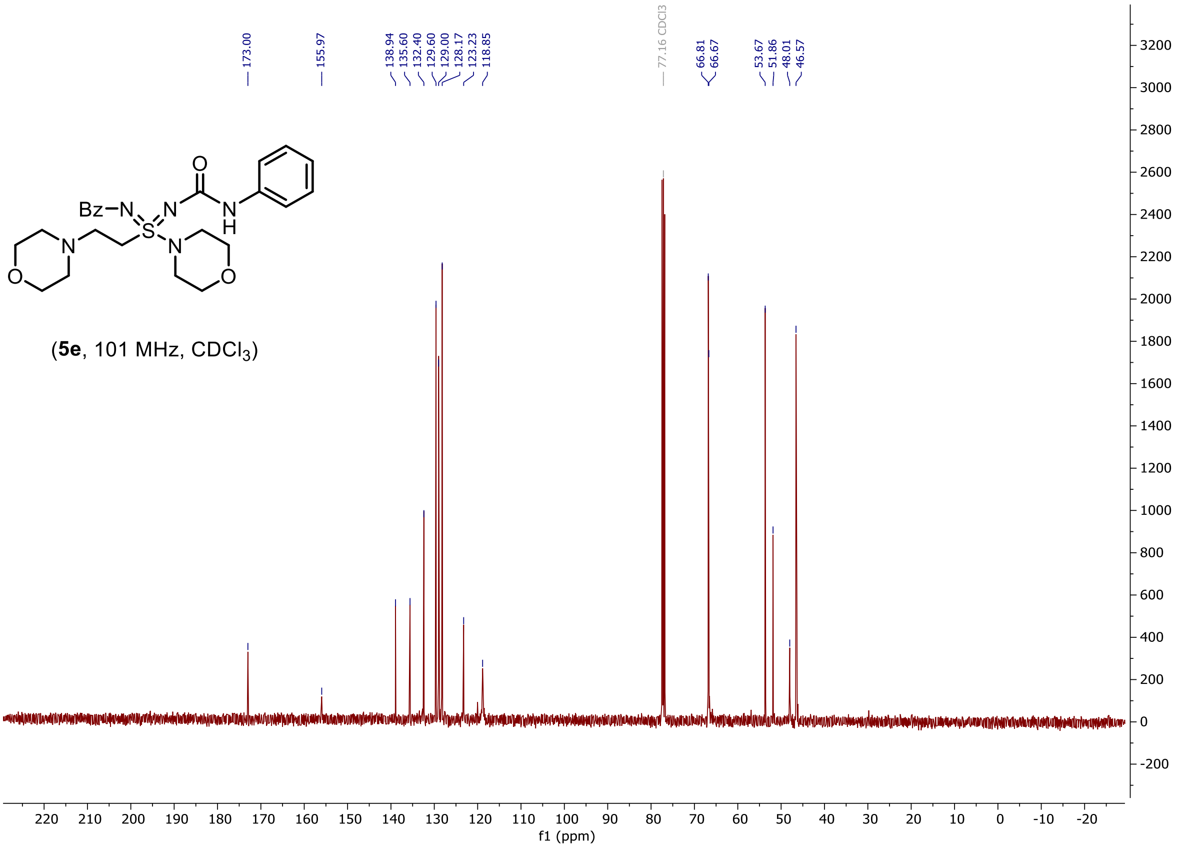

(**5e**, 101 MHz, $CDCl_3$)

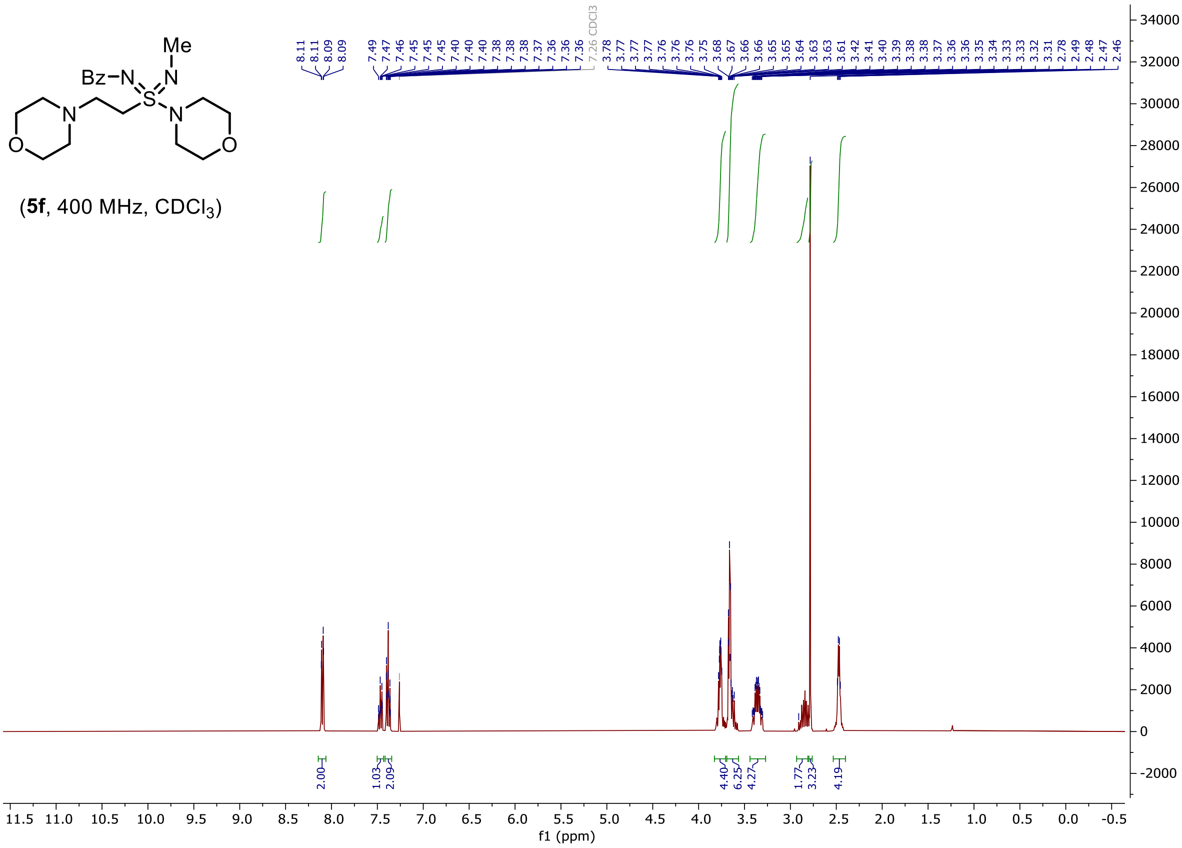
Me
Bz−N
N
S
N
O
N
O
(5f, 400 MHz, CDCl$_3$)
8.11 8.11 8.09 8.09
7.49 7.47 7.46 7.45 7.45 7.45 7.40 7.40 7.40 7.38 7.38 7.38 7.37 7.36 7.36 7.36
7.26 CDCl3
3.78 3.77 3.77 3.77 3.76 3.76 3.76 3.75 3.68 3.67 3.66 3.66 3.65 3.65 3.64 3.63 3.63 3.61 3.42 3.41 3.40 3.39 3.38 3.38 3.37 3.36 3.36 3.35 3.34 3.33 3.33 3.32 3.31 2.78 2.49 2.48 2.47 2.46
2.00
1.03 2.09
4.40 6.25 4.27
1.77 3.23
4.19
f1 (ppm)

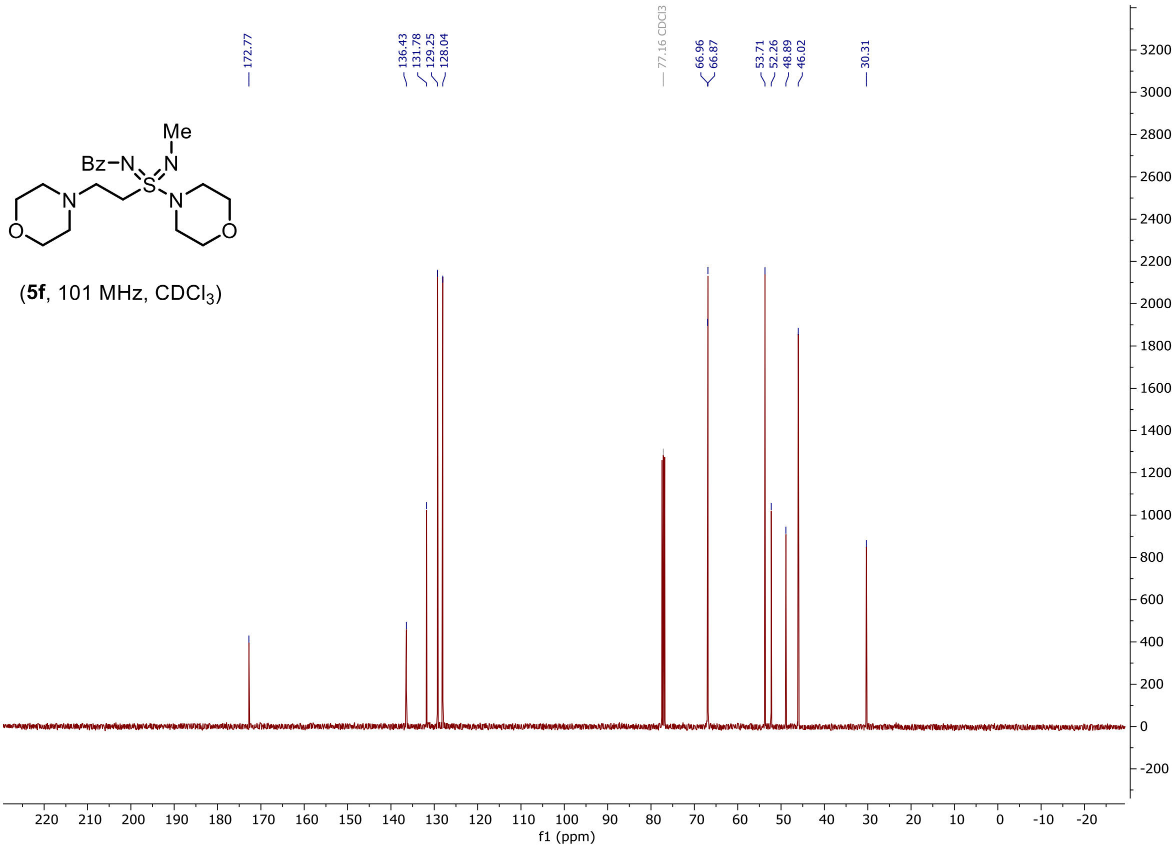
172.77
136.43 131.78 129.25 128.04
77.16 CDCl3
66.96 66.87
53.71 52.26 48.89 46.02
30.31
Me
Bz−N
N
S
N
O
N
O
(5f, 101 MHz, CDCl$_3$)
f1 (ppm)

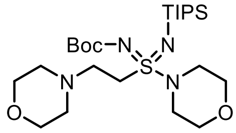

($\mathbf{5g}$, 400 MHz, $CDCl_3$)

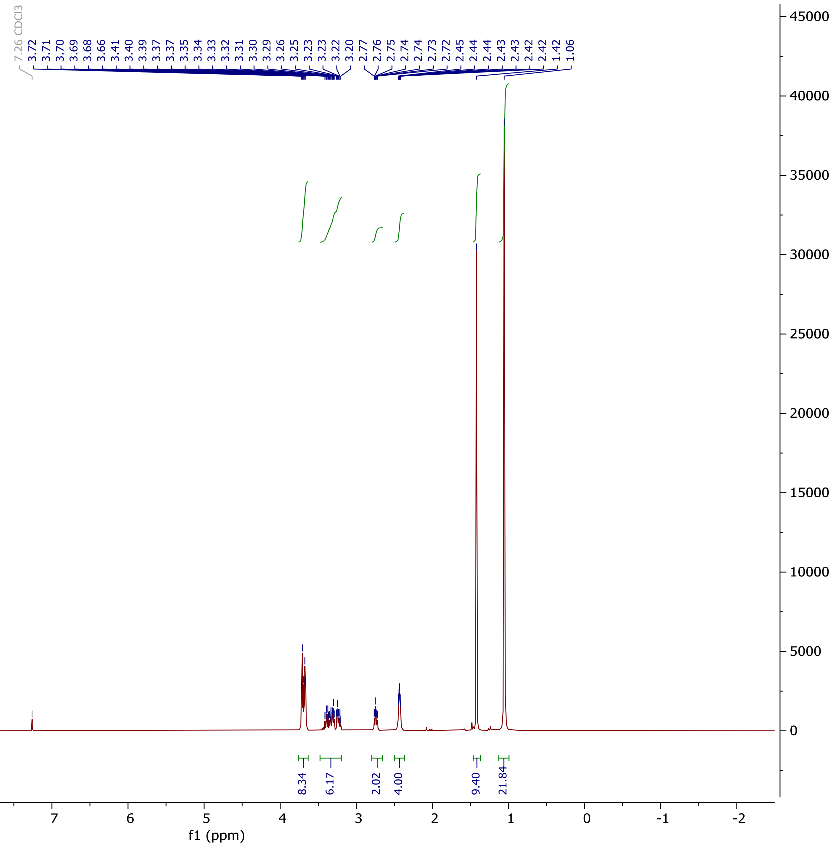

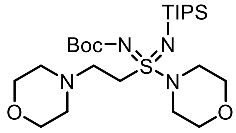

($\mathbf{5g}$, 101 MHz, $CDCl_3$)

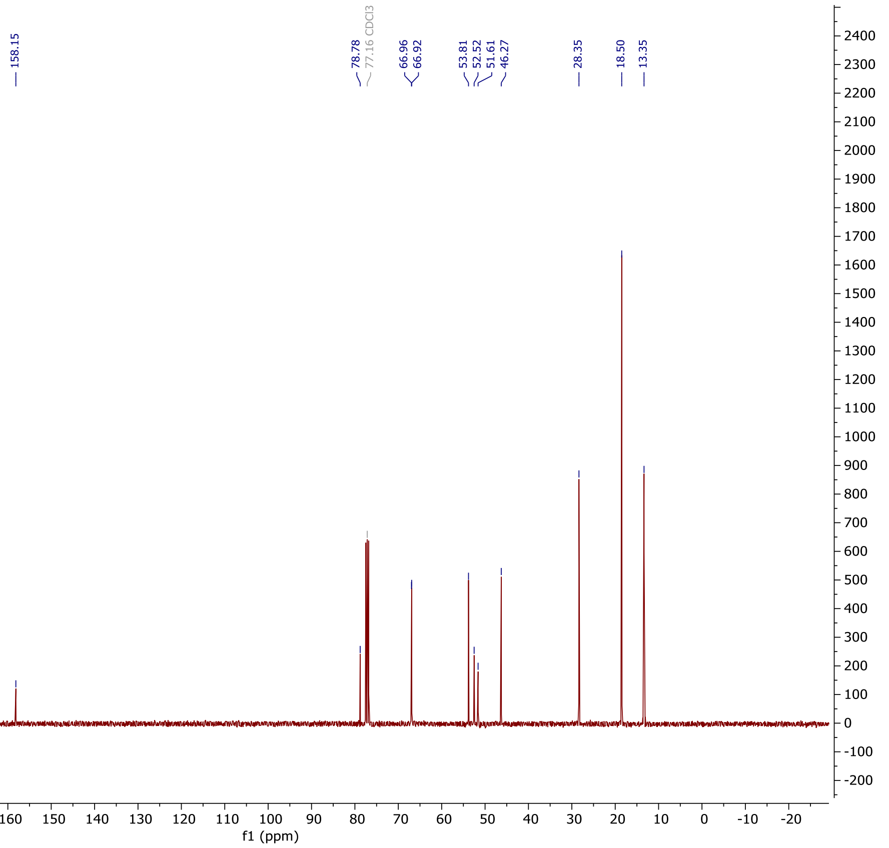

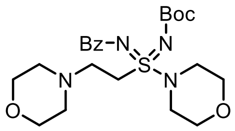

(**5h**, 400 MHz, $CDCl_3$)

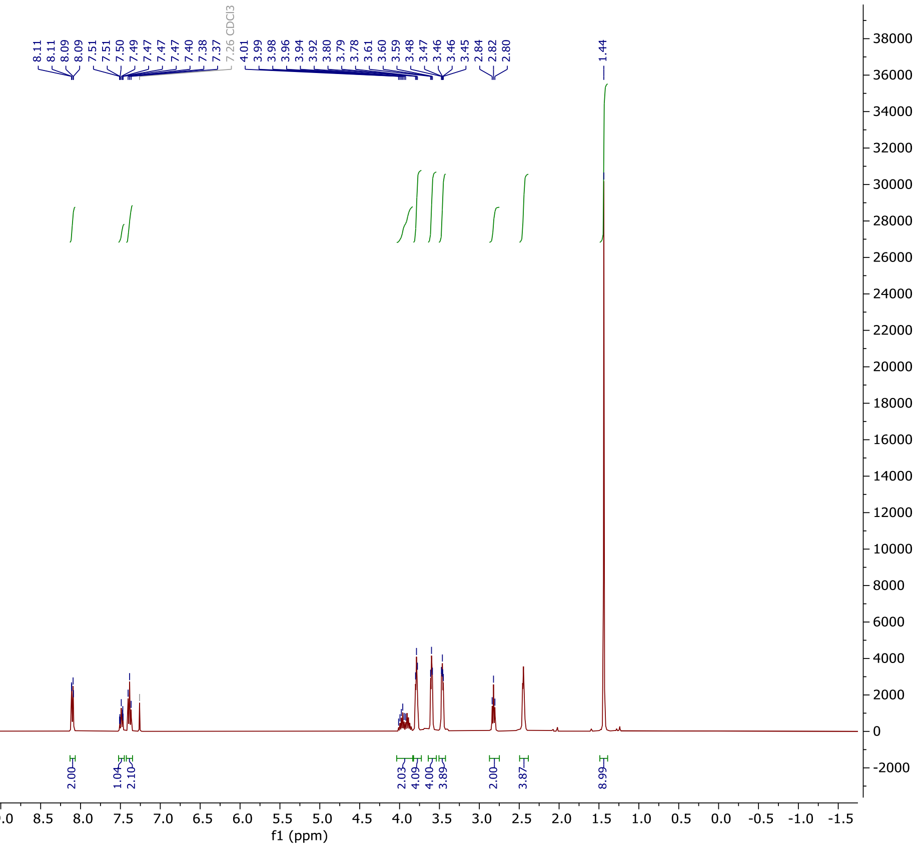

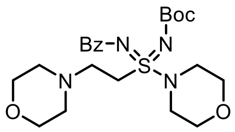

(**5h**, 101 MHz, $CDCl_3$)

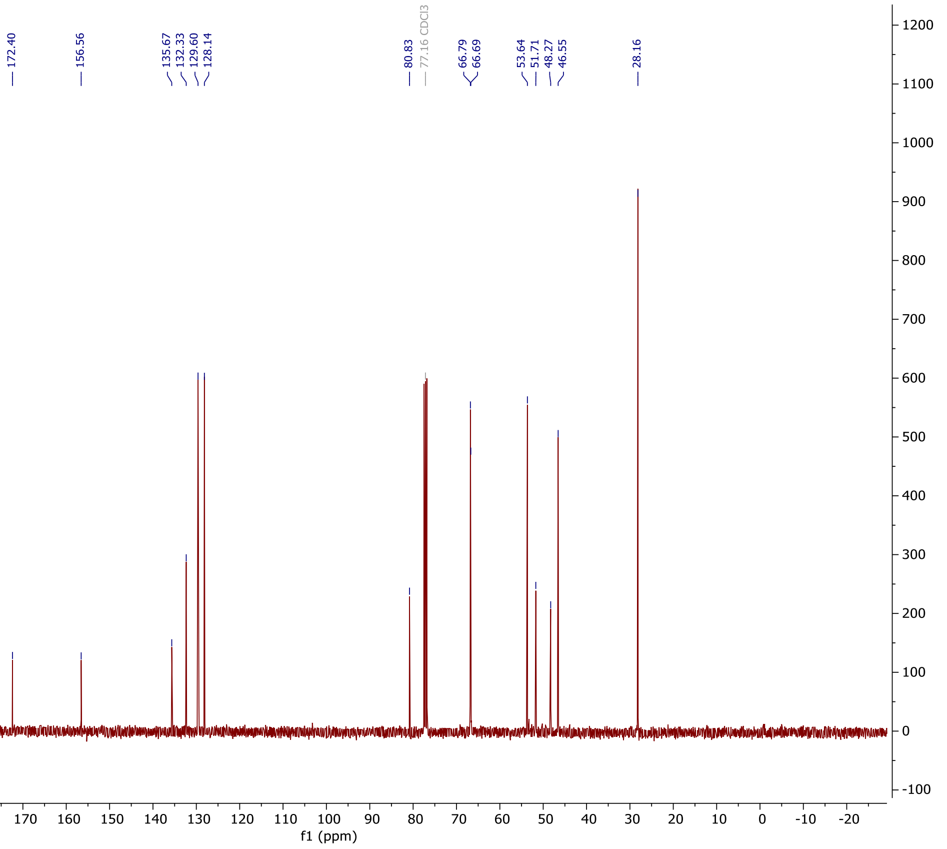

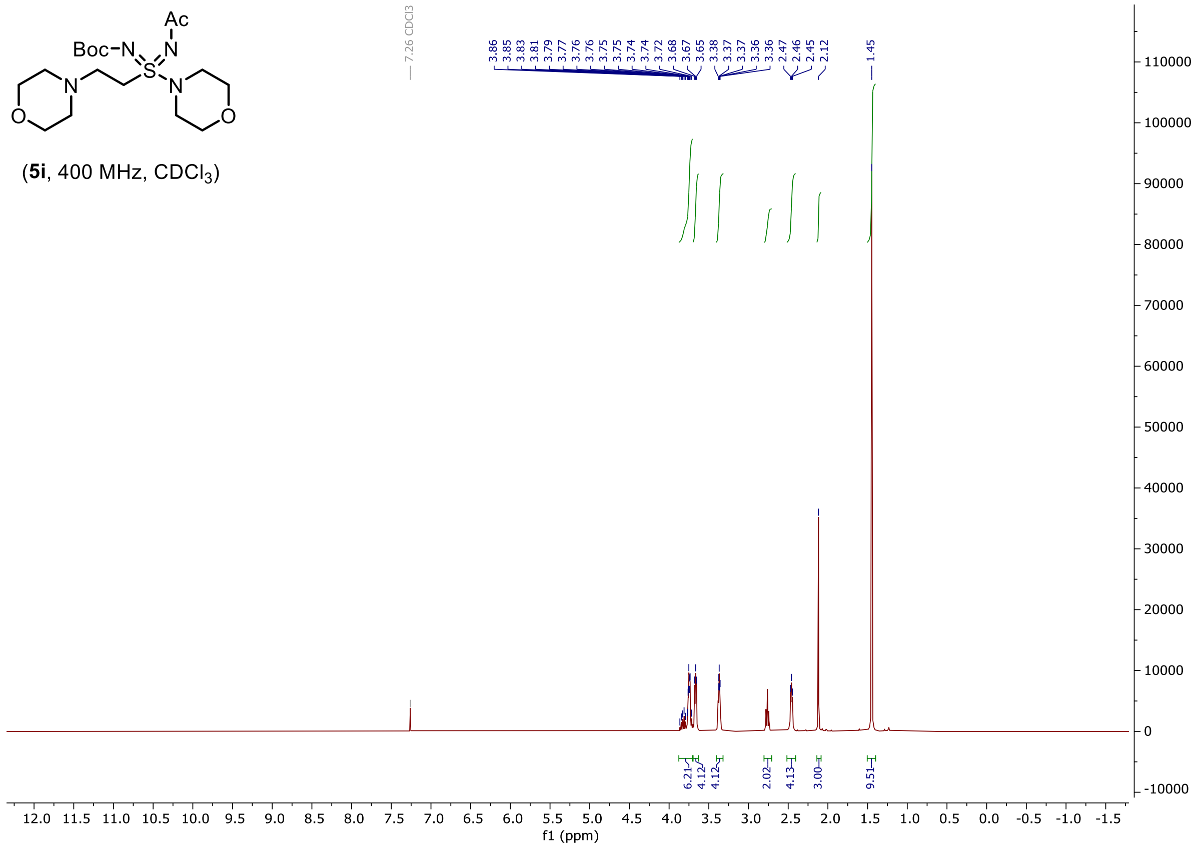

(**5i**, 400 MHz, $CDCl_3$)

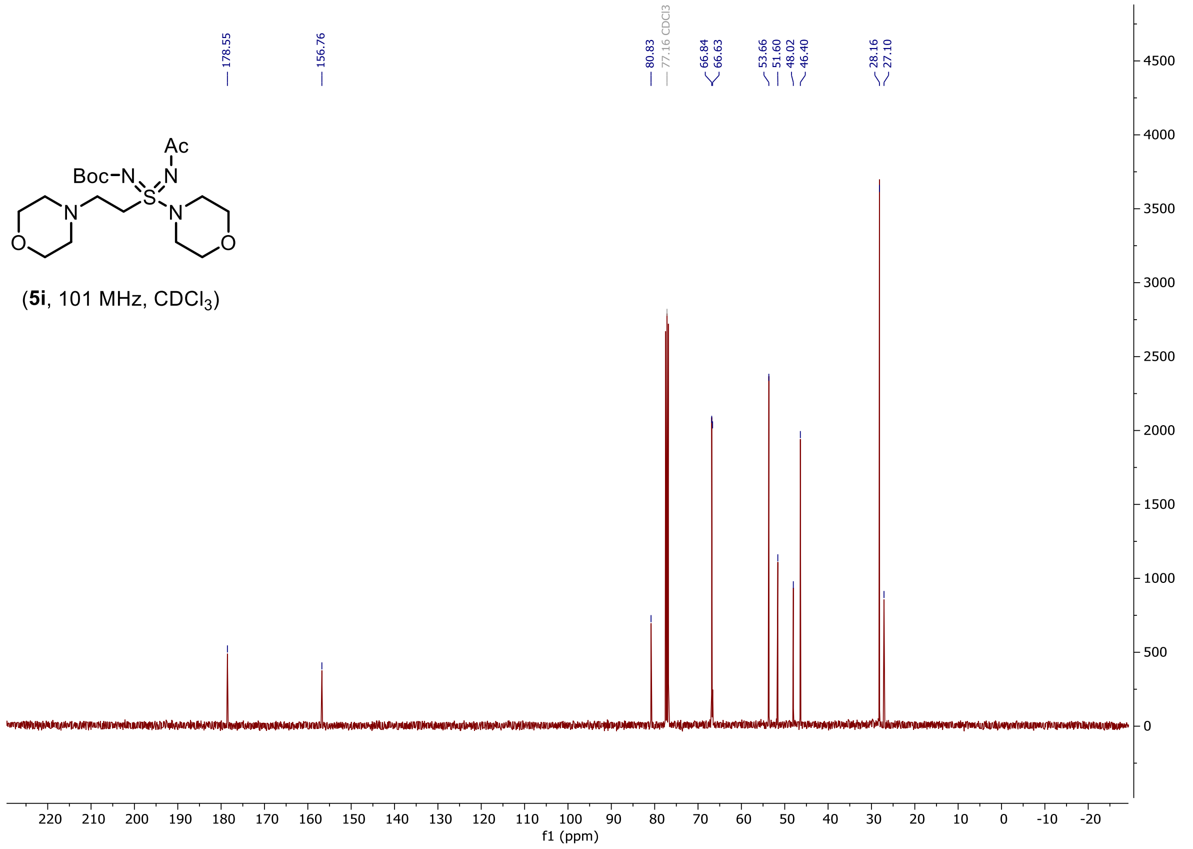

(**5i**, 101 MHz, $CDCl_3$)

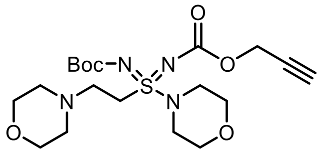

(**5j**, 400 MHz, $CDCl_3$)

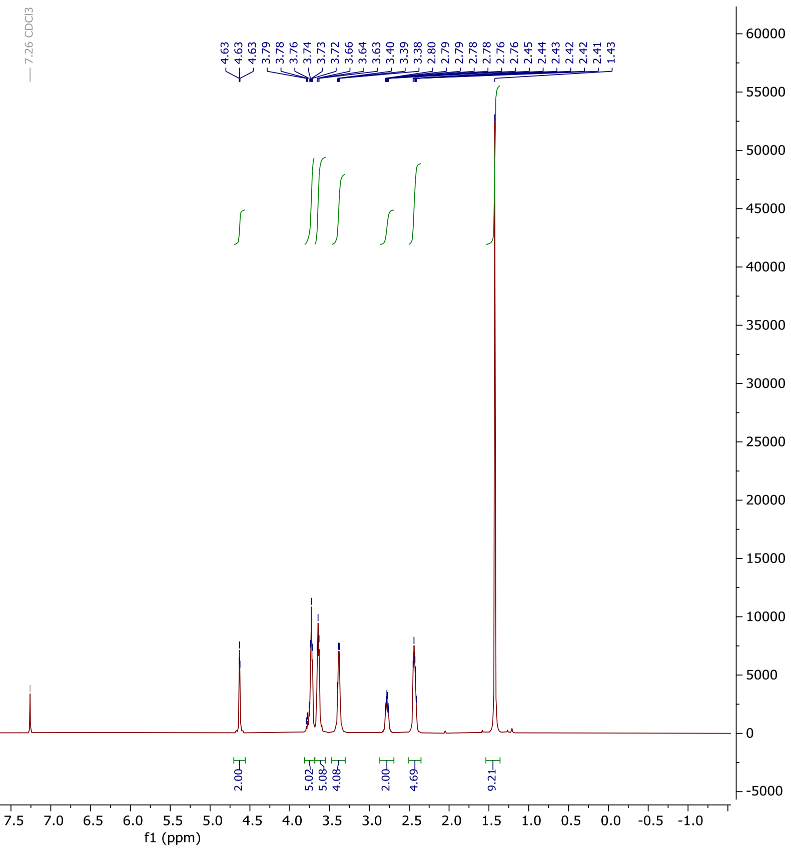

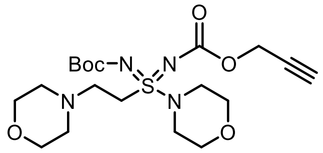

(**5j**, 101 MHz, $CDCl_3$)

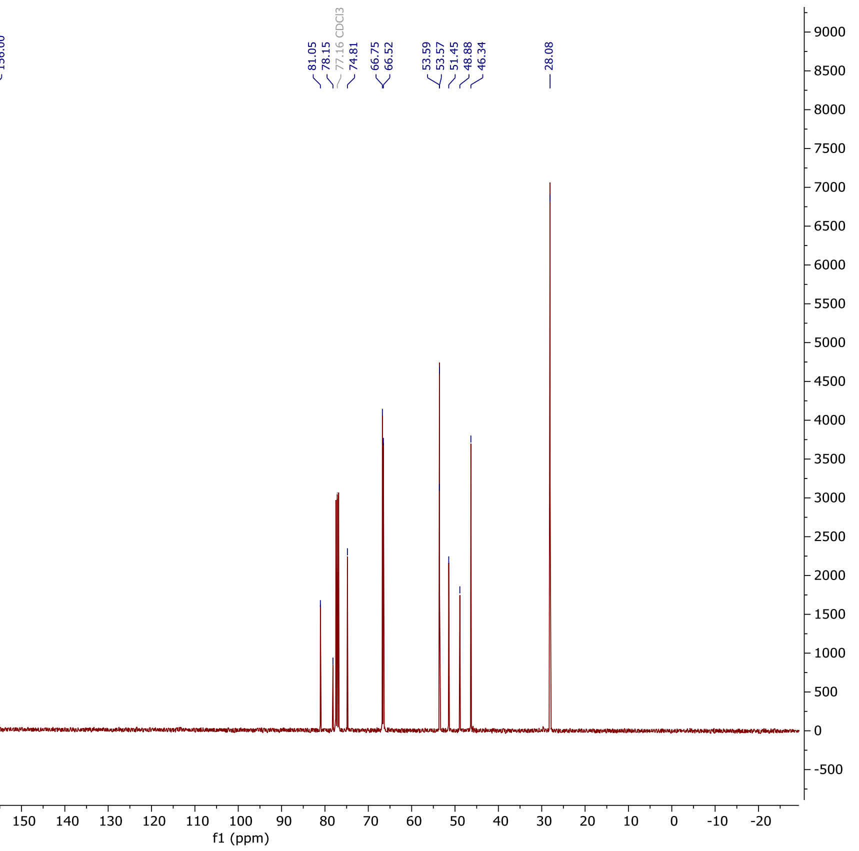

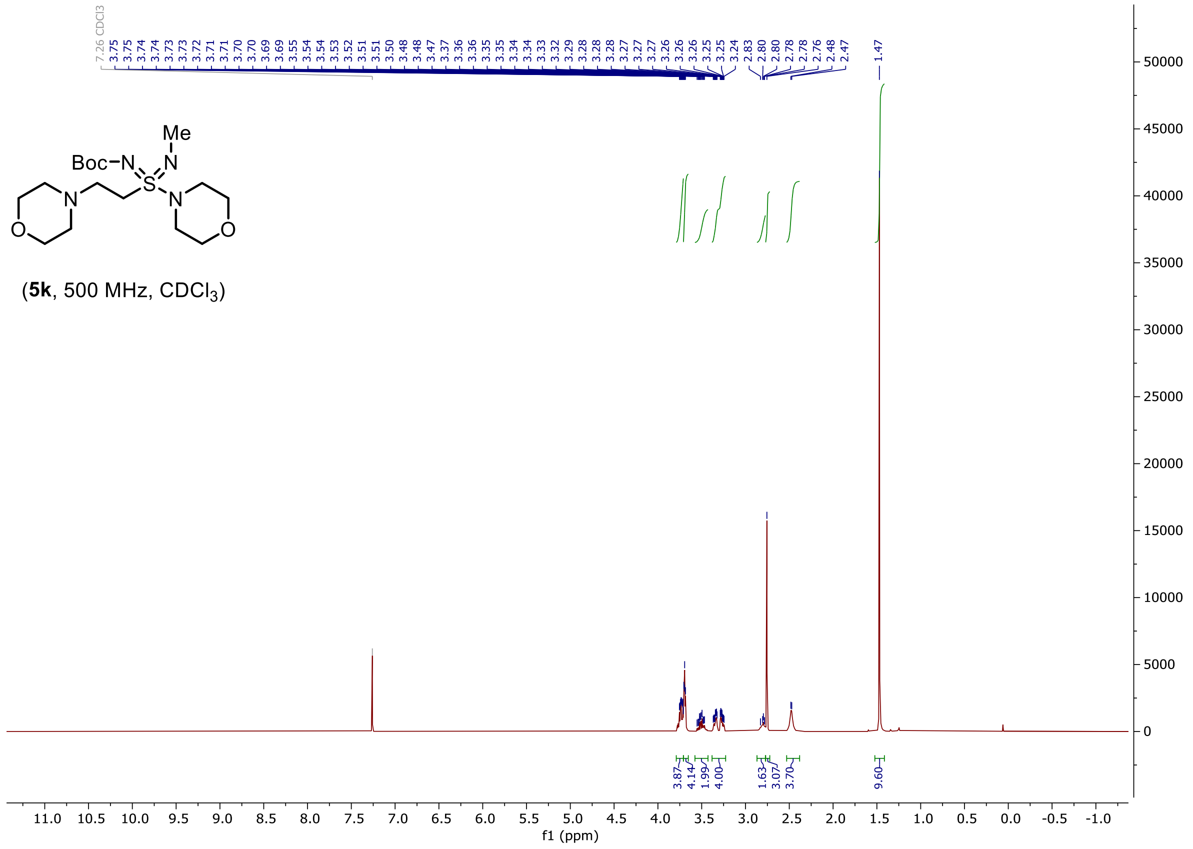
7.26 CDCl3
3.75 3.75 3.74 3.74 3.73 3.73 3.72 3.71 3.71 3.70 3.69 3.69 3.55 3.54 3.54 3.53 3.52 3.51 3.51 3.50 3.48 3.48 3.47 3.37 3.36 3.36 3.35 3.35 3.34 3.34 3.33 3.32 3.29 3.28 3.28 3.28 3.27 3.27 3.27 3.26 3.26 3.26 3.25 3.25 3.25 3.24 2.83 2.80 2.80 2.78 2.78 2.76 2.48 2.47
1.47
Me
Boc
N
S
O
(**5k**, 500 MHz, $CDCl_3$)
3.87 4.14 1.99 4.00 1.63 3.07 3.70 9.60
f1 (ppm)

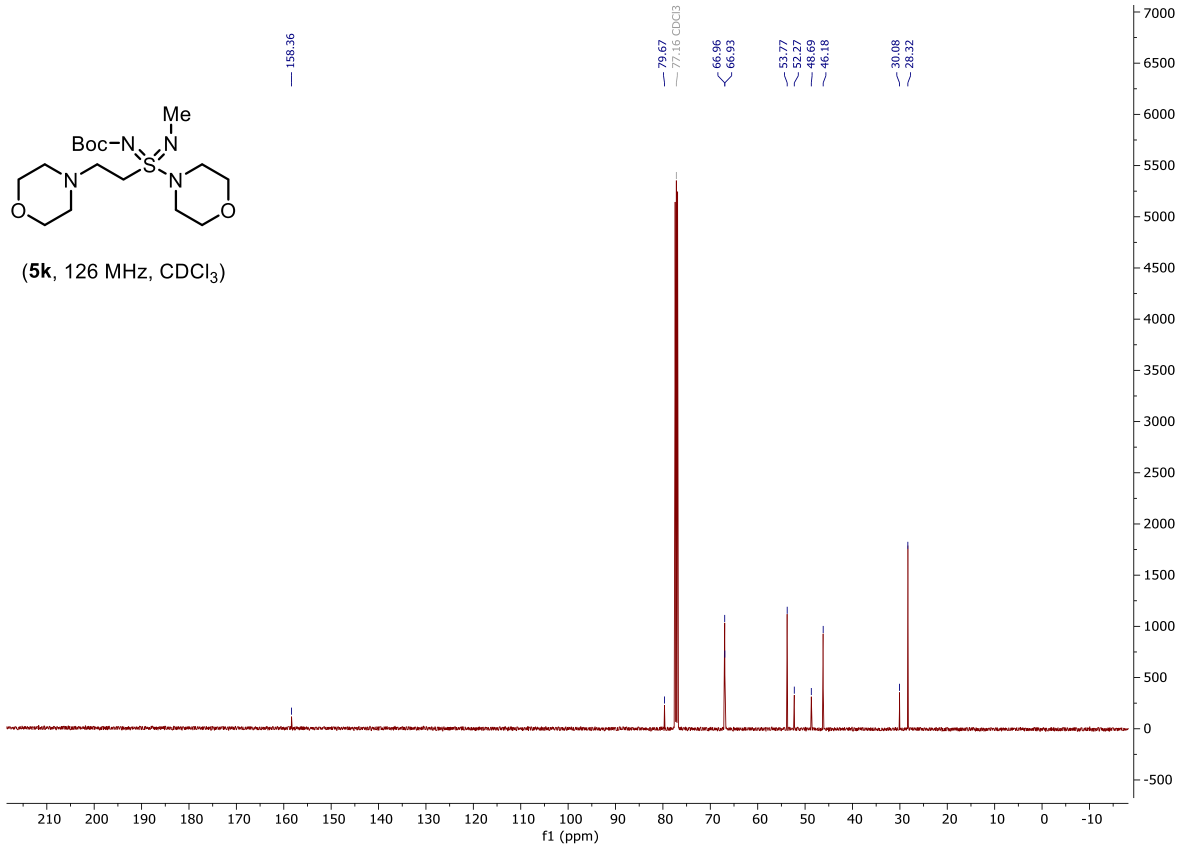
158.36
79.67
77.16 CDCl3
66.96 66.93
53.77 52.27 48.69 46.18
30.08 28.32
Me
Boc
N
S
O
(**5k**, 126 MHz, $CDCl_3$)
f1 (ppm)

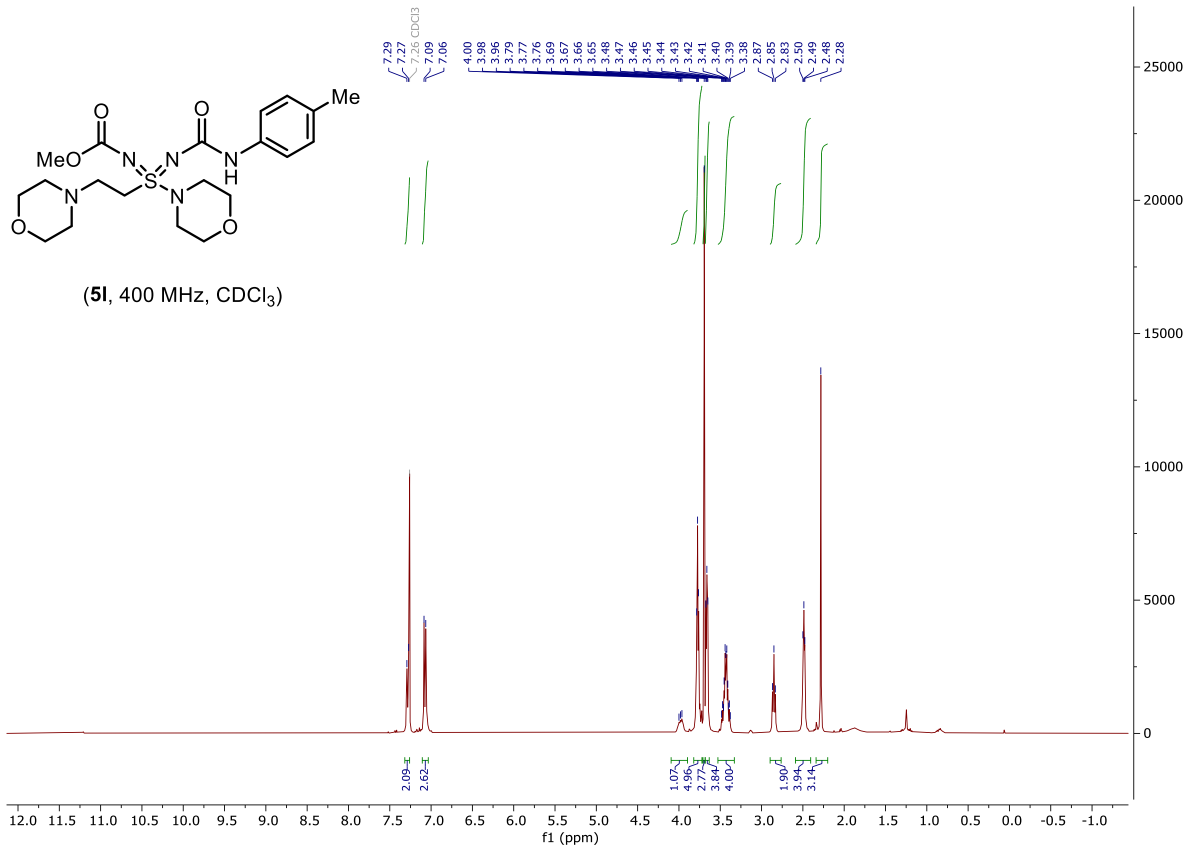

(**5l**, 400 MHz, $CDCl_3$)

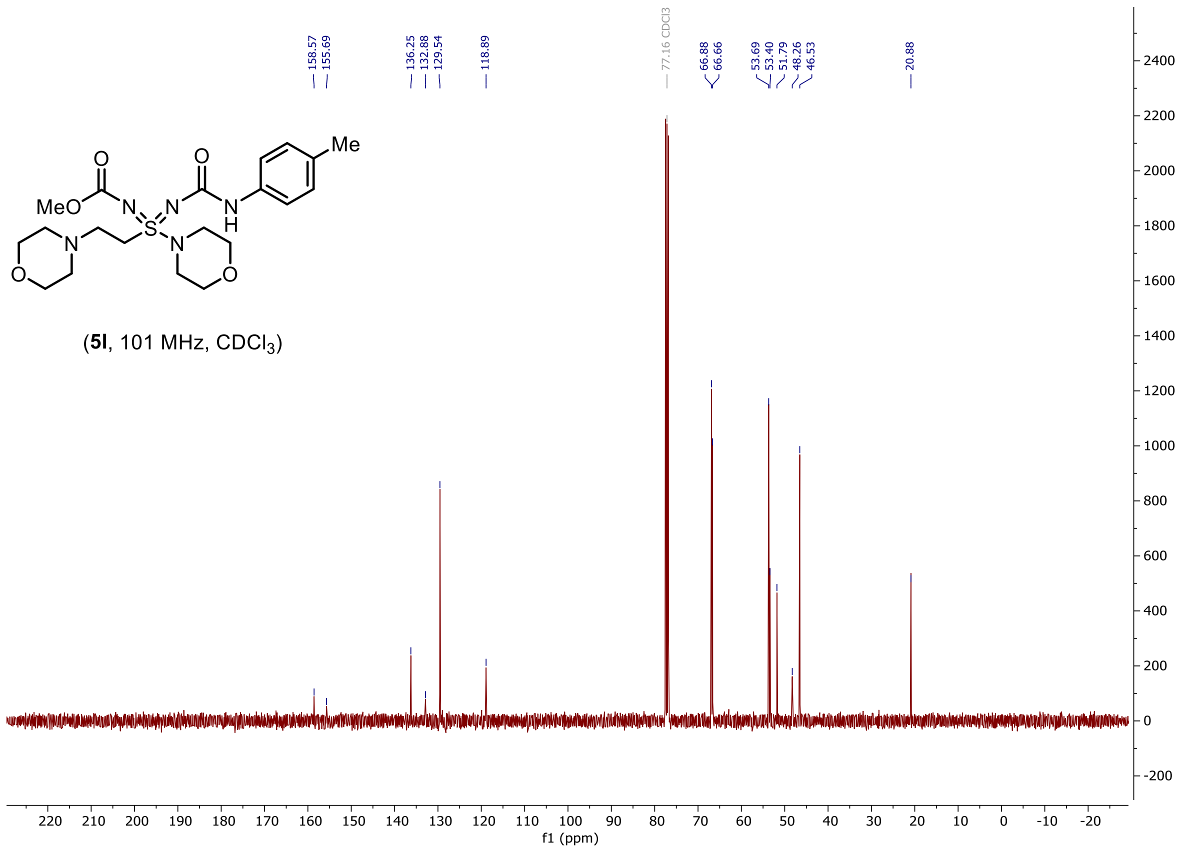

(**5l**, 101 MHz, $CDCl_3$)

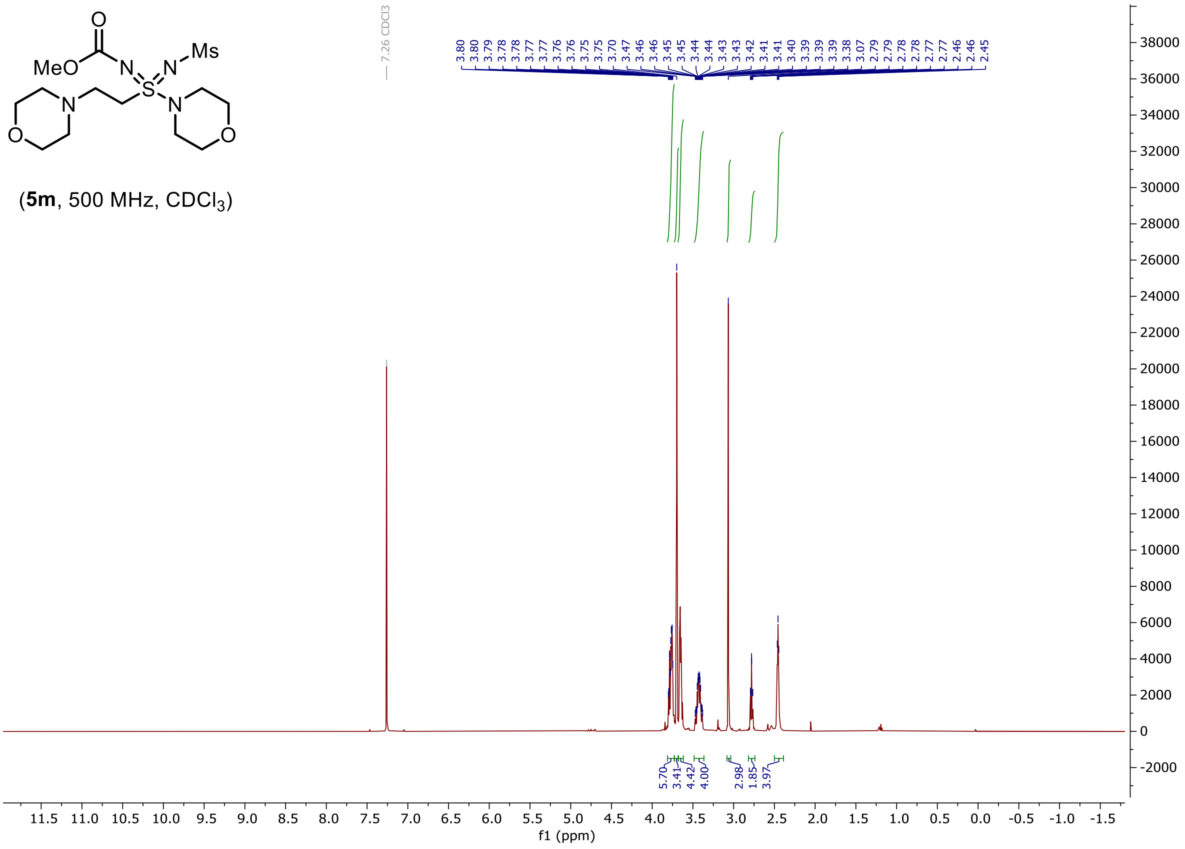

(**5m**, 500 MHz, $CDCl_3$)

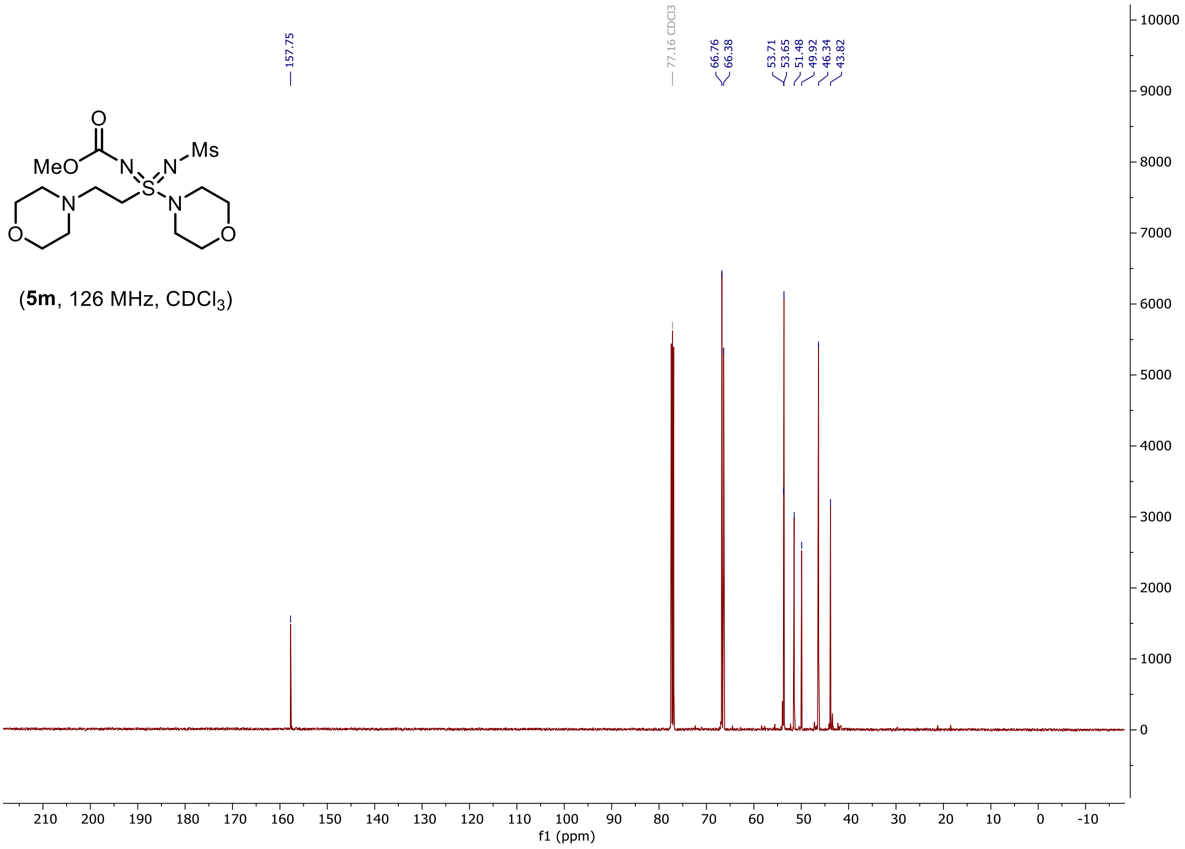

(**5m**, 126 MHz, $CDCl_3$)

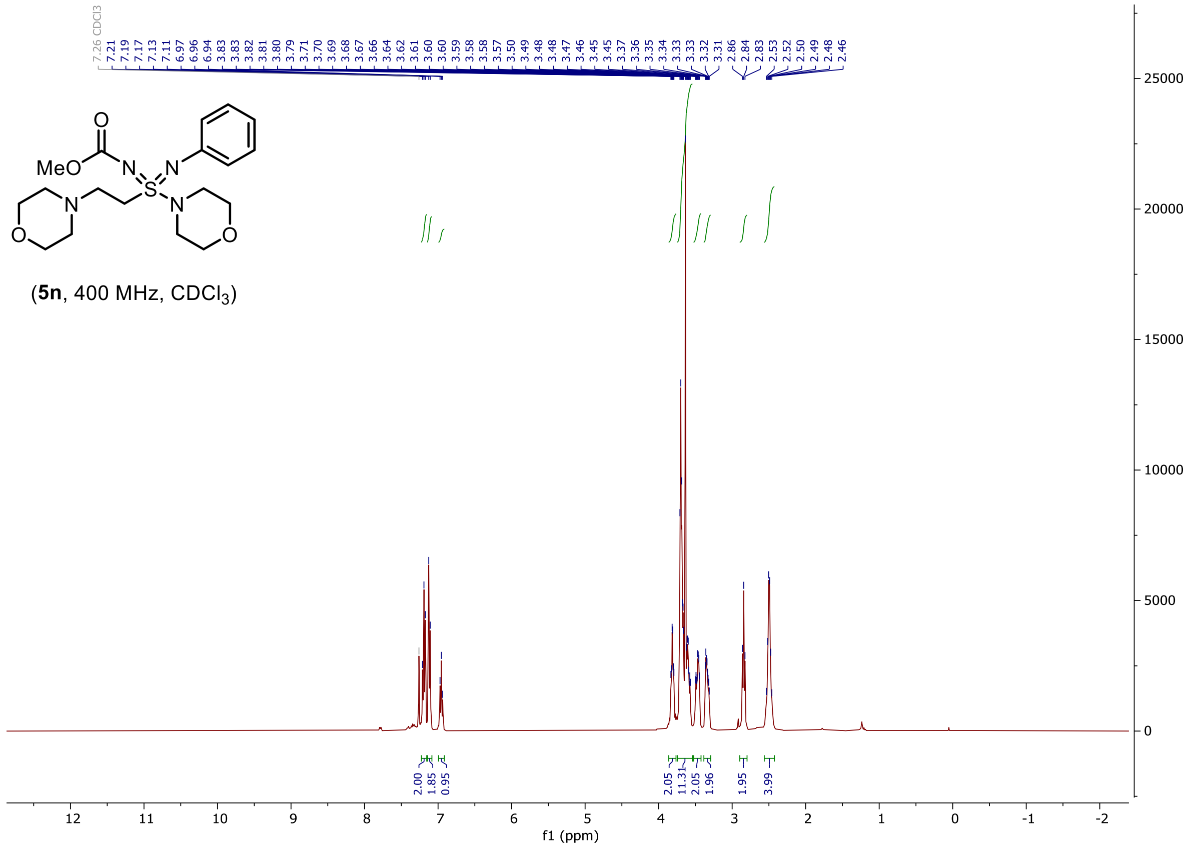

(**5n**, 400 MHz, $CDCl_3$)

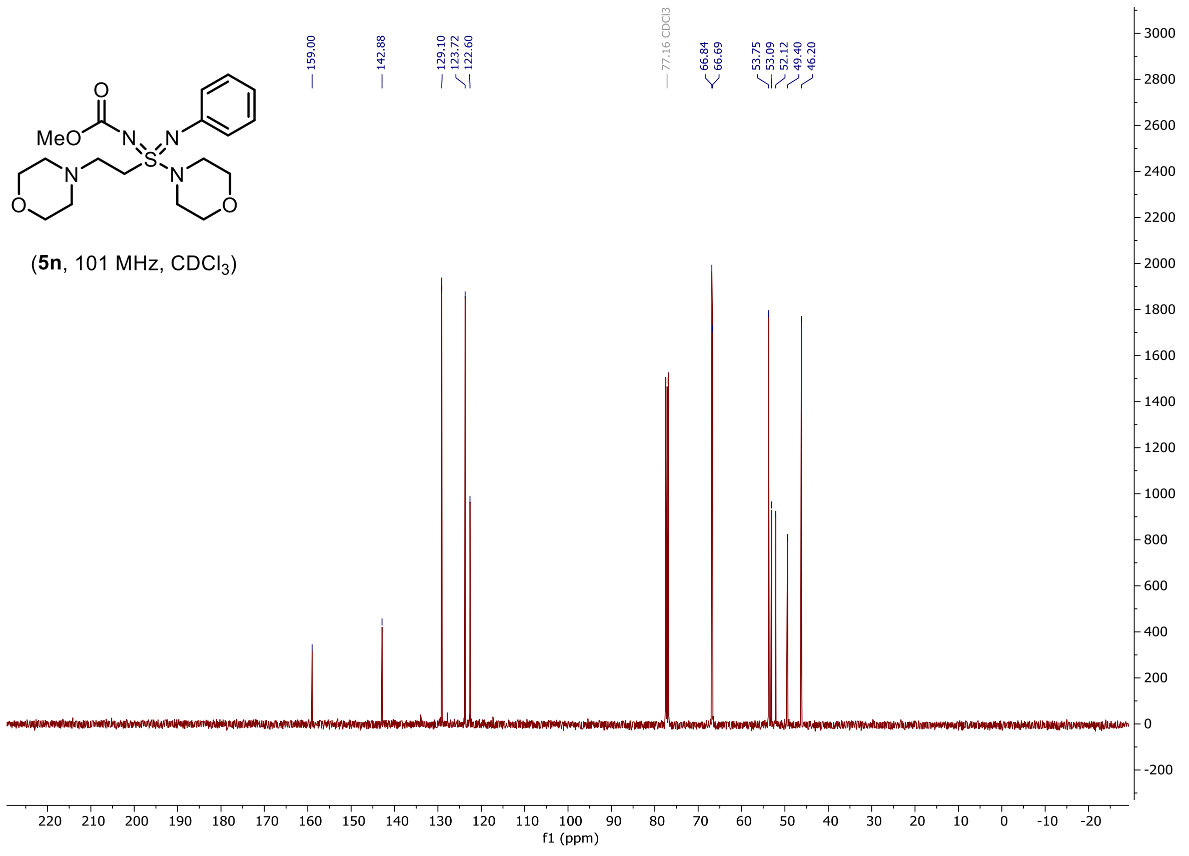

(**5n**, 101 MHz, $CDCl_3$)

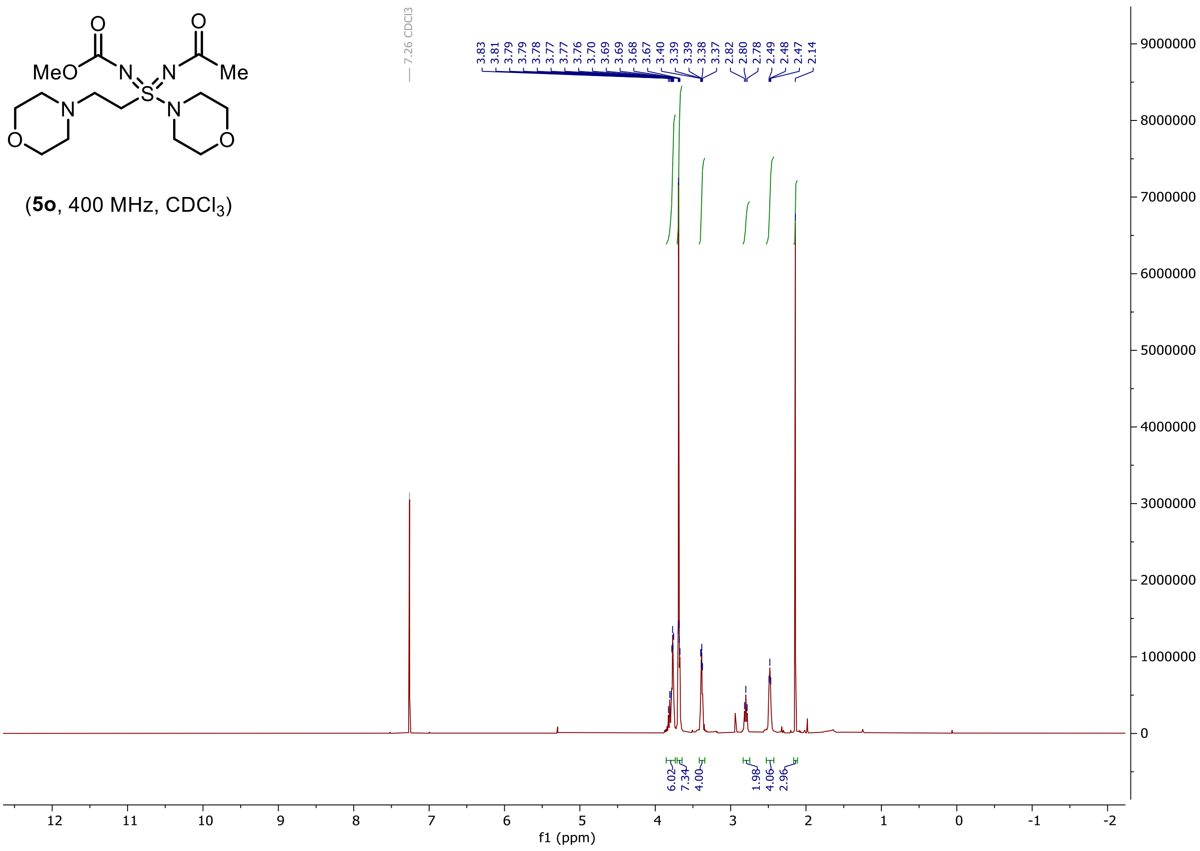

(**5o**, 400 MHz, $CDCl_3$)

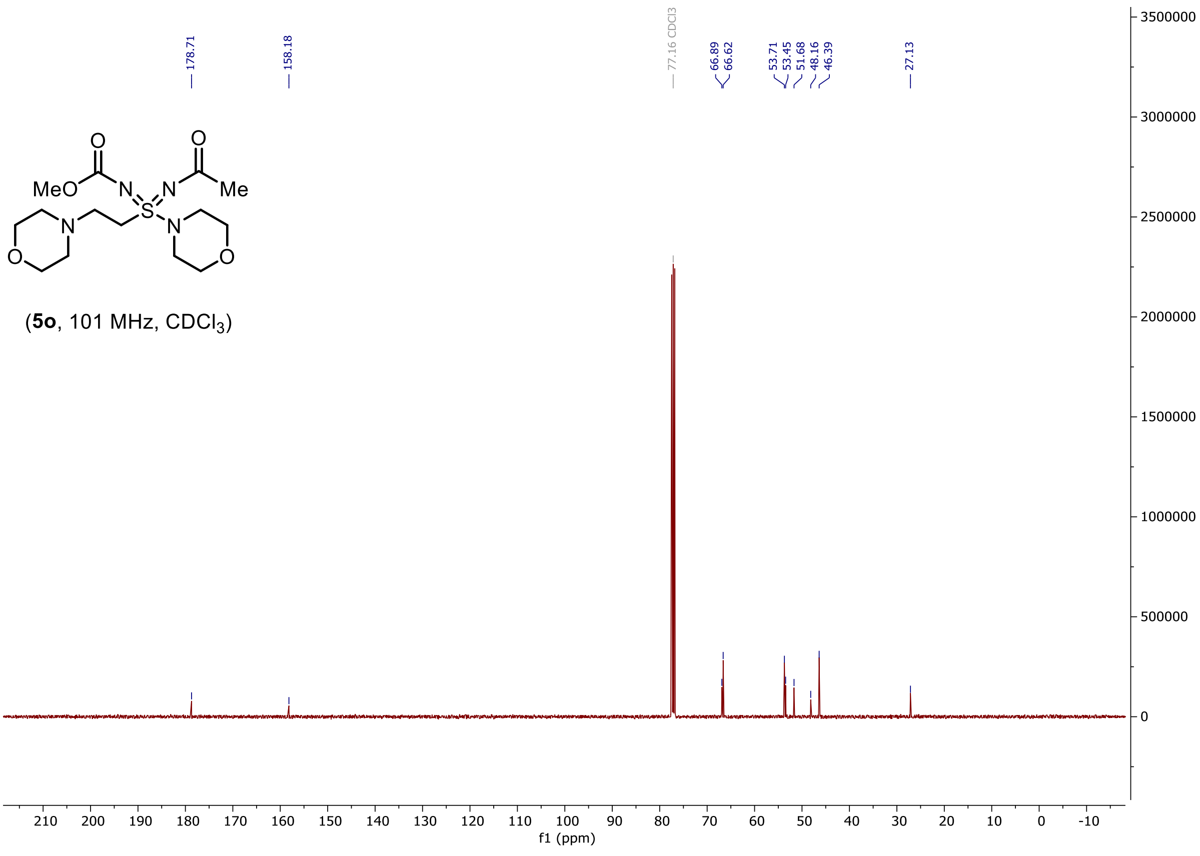

(**5o**, 101 MHz, $CDCl_3$)

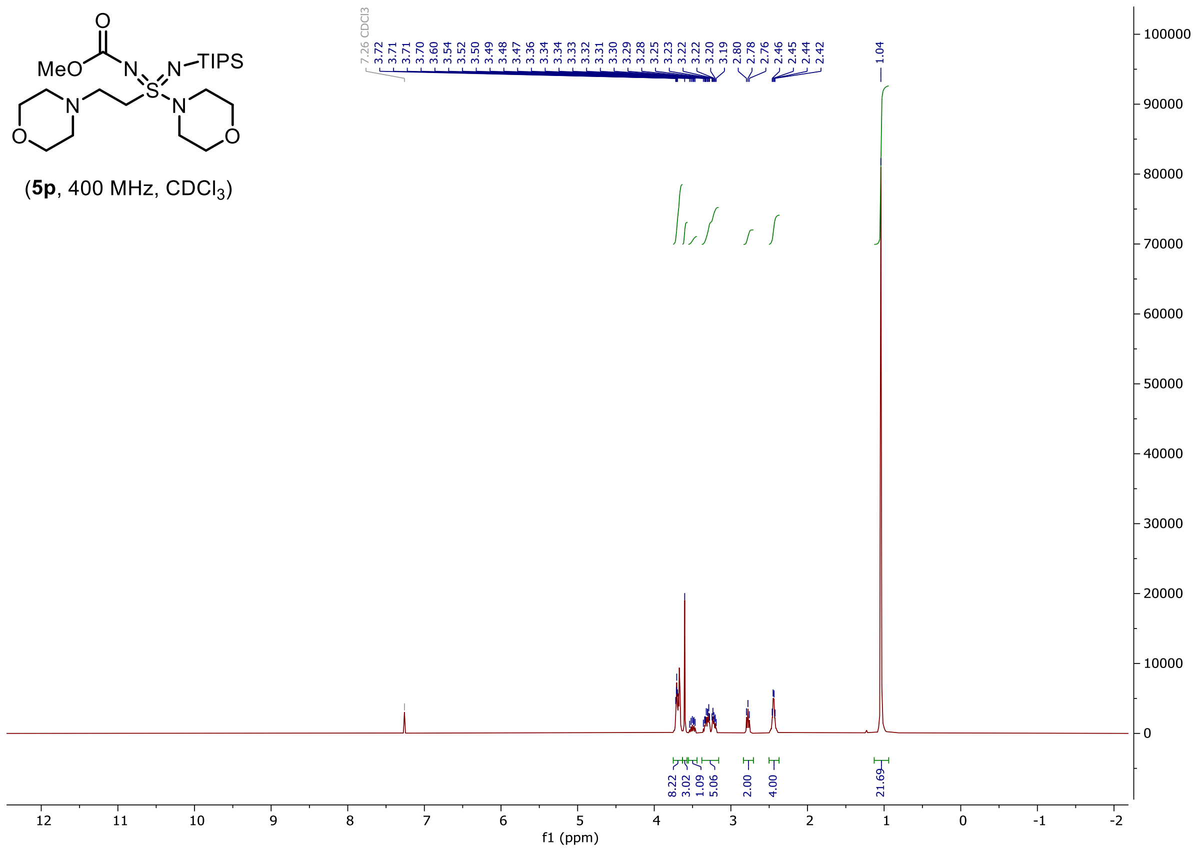
MeO
N
N–TIPS
S
N
O
(5p, 400 MHz, CDCl3)
7.26 CDCl3
3.72
3.71
3.71
3.70
3.60
3.54
3.52
3.50
3.49
3.48
3.47
3.36
3.34
3.34
3.33
3.32
3.31
3.30
3.29
3.28
3.25
3.23
3.22
3.22
3.20
3.19
2.80
2.78
2.76
2.46
2.45
2.44
2.42
1.04
8.22
3.02
1.09
5.06
2.00
4.00
21.69
f1 (ppm)

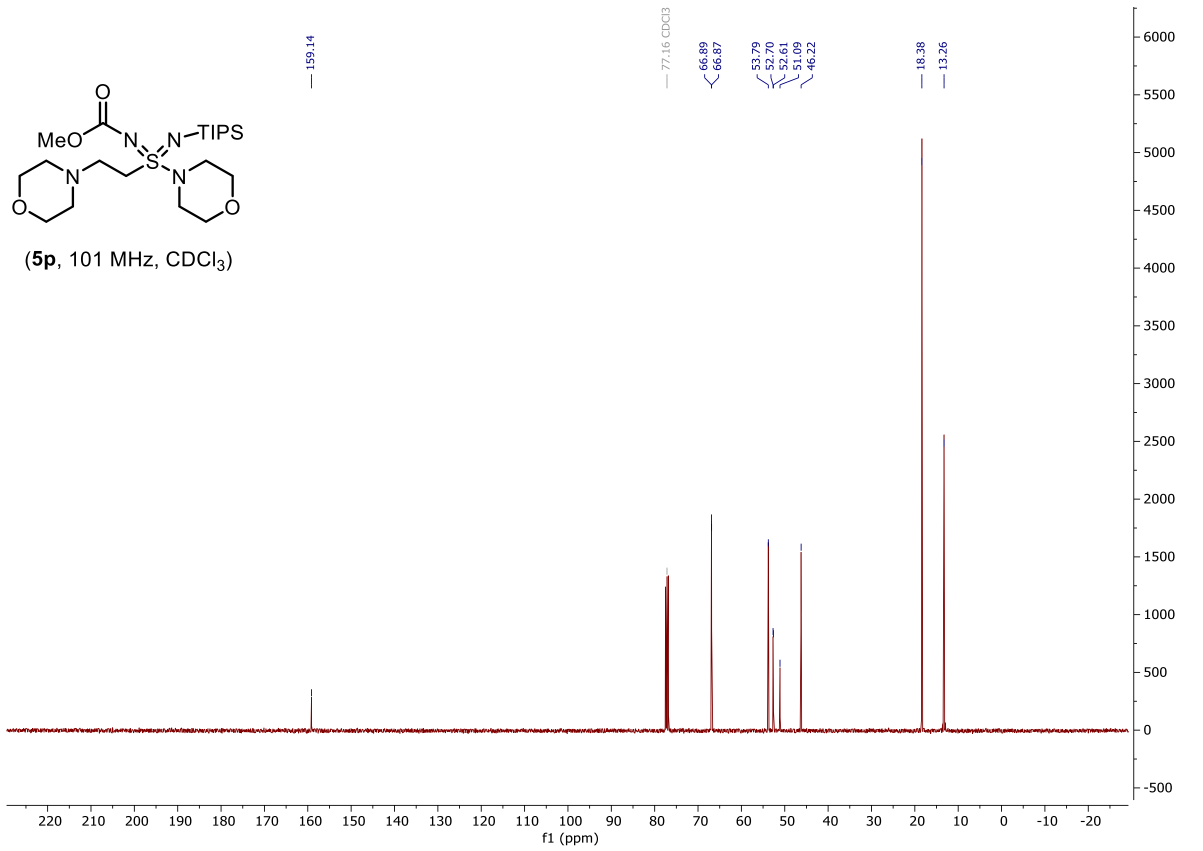
MeO
N
N–TIPS
S
N
O
(5p, 101 MHz, CDCl3)
159.14
77.16 CDCl3
66.89
66.87
53.79
52.70
52.61
51.09
46.22
18.38
13.26
f1 (ppm)

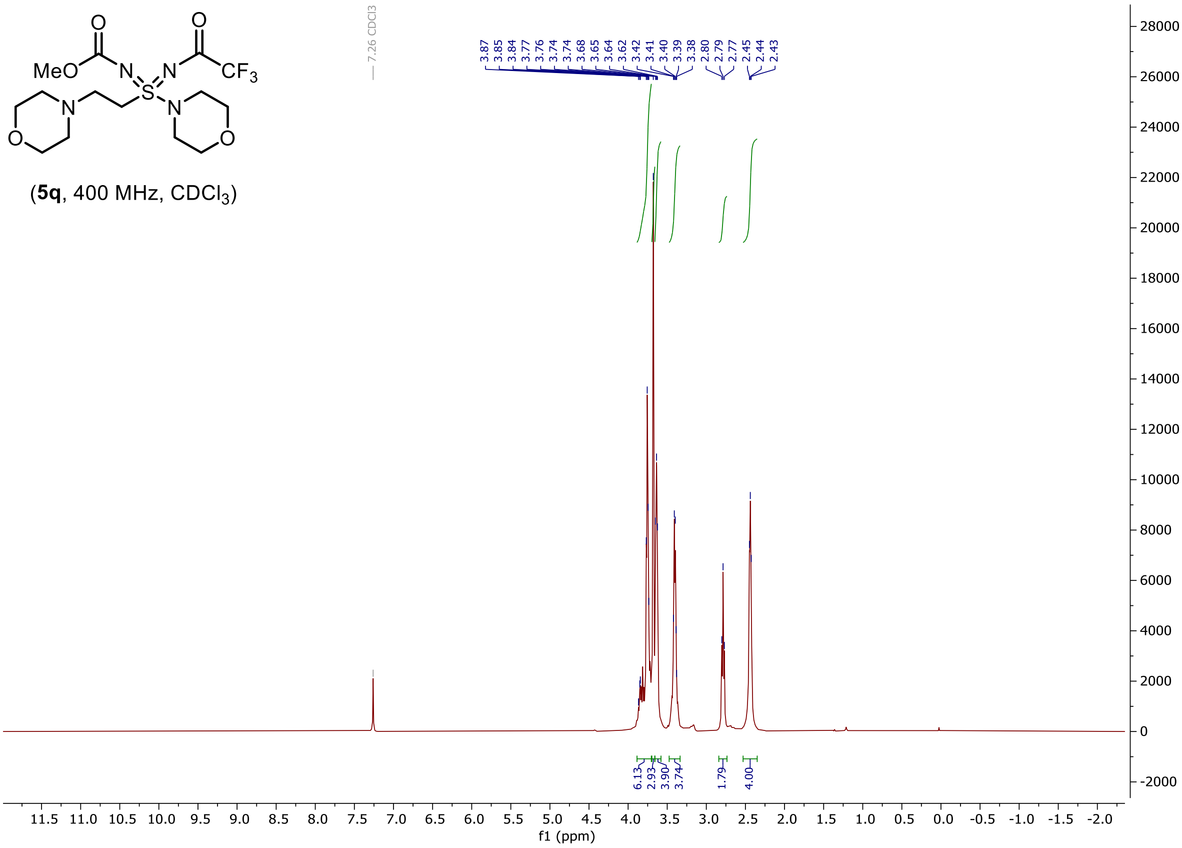

(**5q**, 400 MHz, $CDCl_3$)

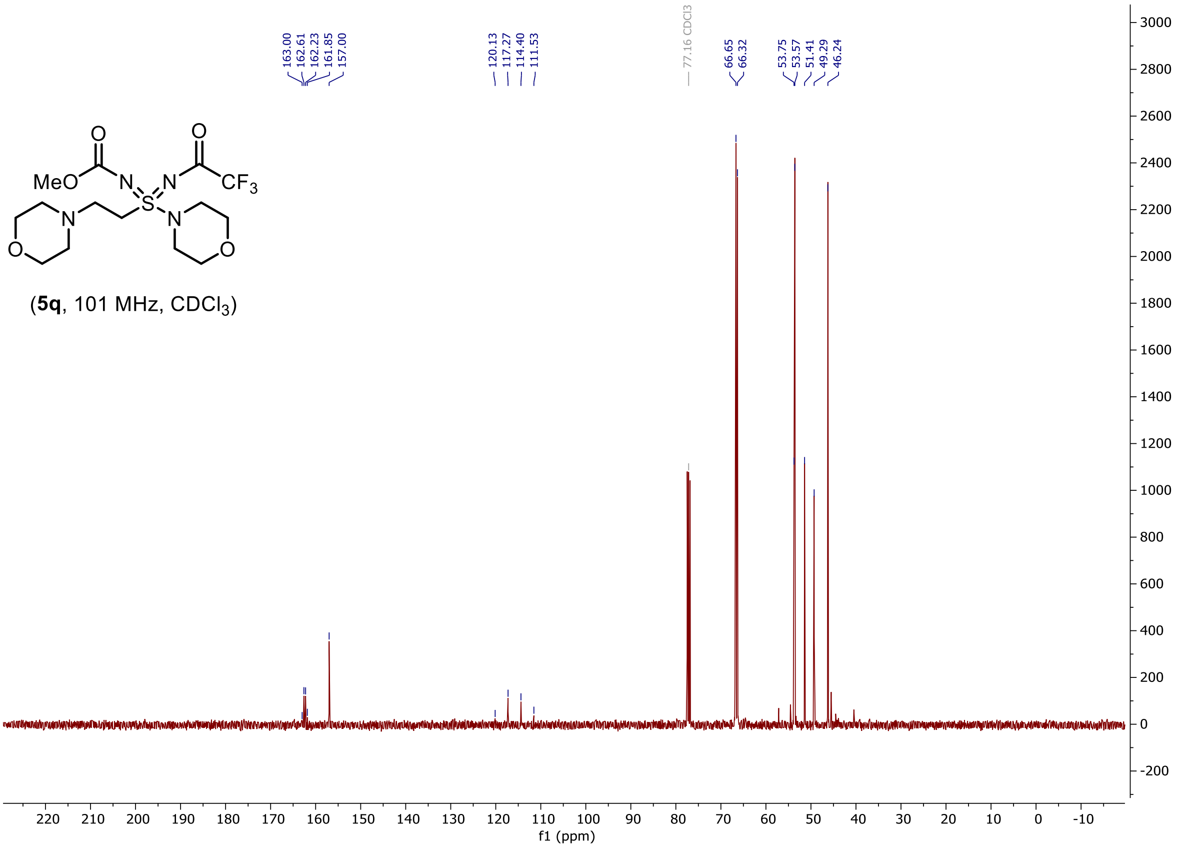

(**5q**, 101 MHz, $CDCl_3$)

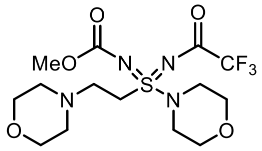

(**5q**, 377 MHz, $CDCl_3$)

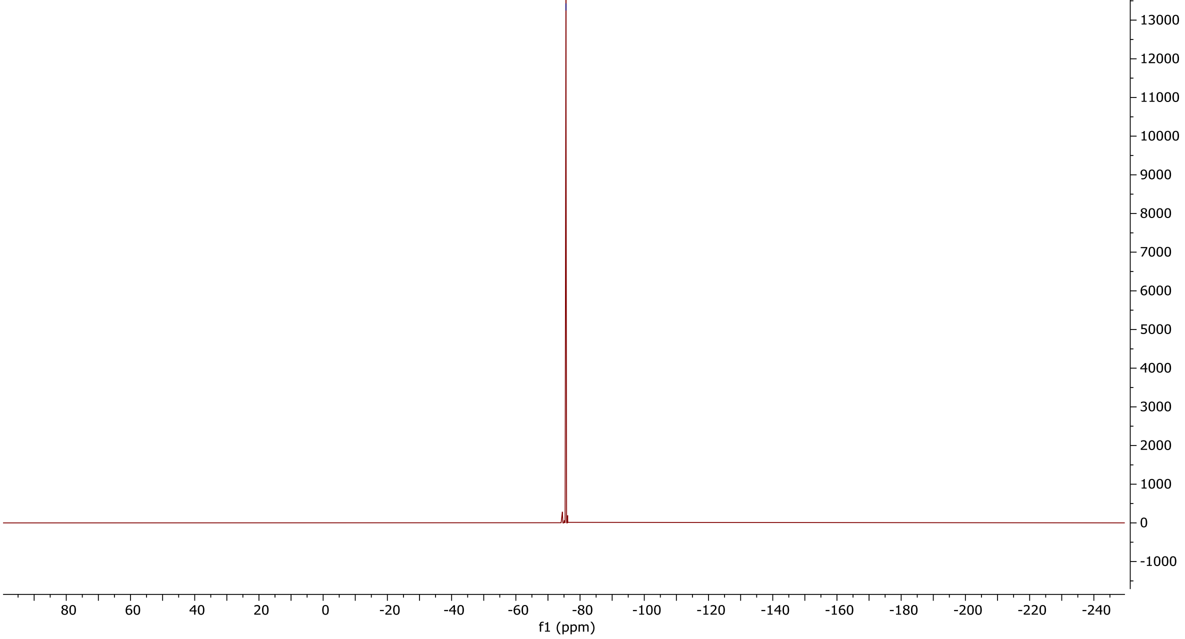

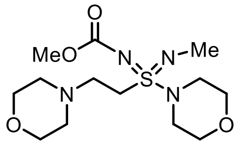

(**5r**, 400 MHz, CDCl$_3$)

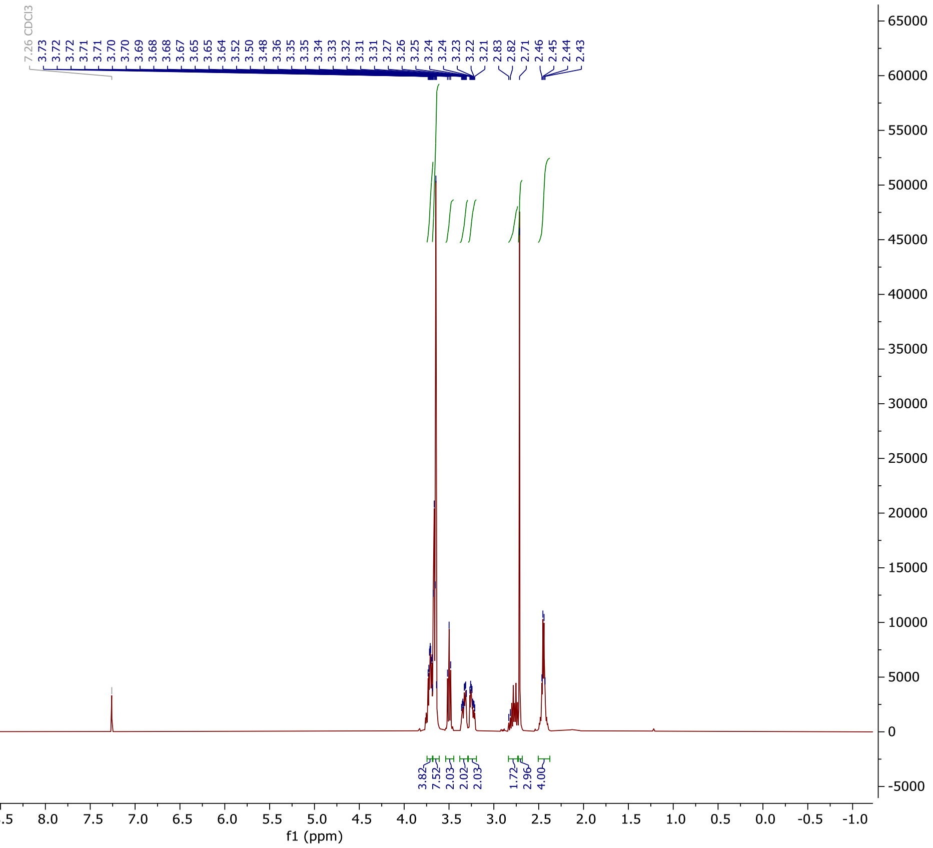

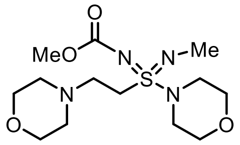

(**5r**, 101 MHz, CDCl$_3$)

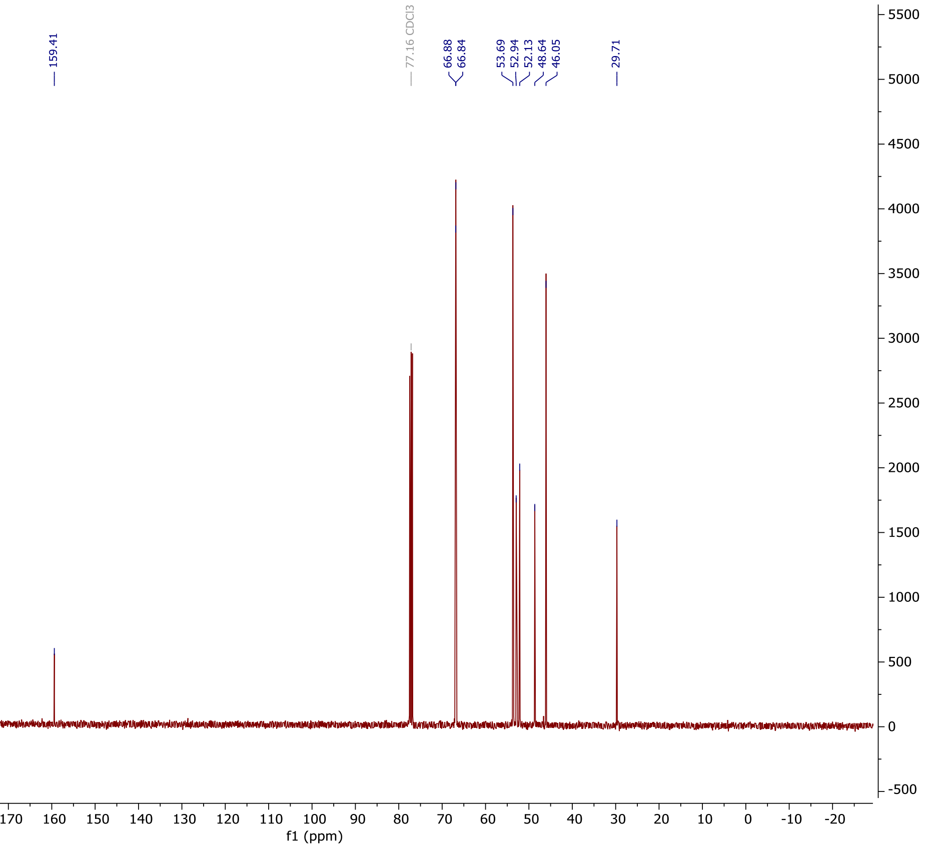

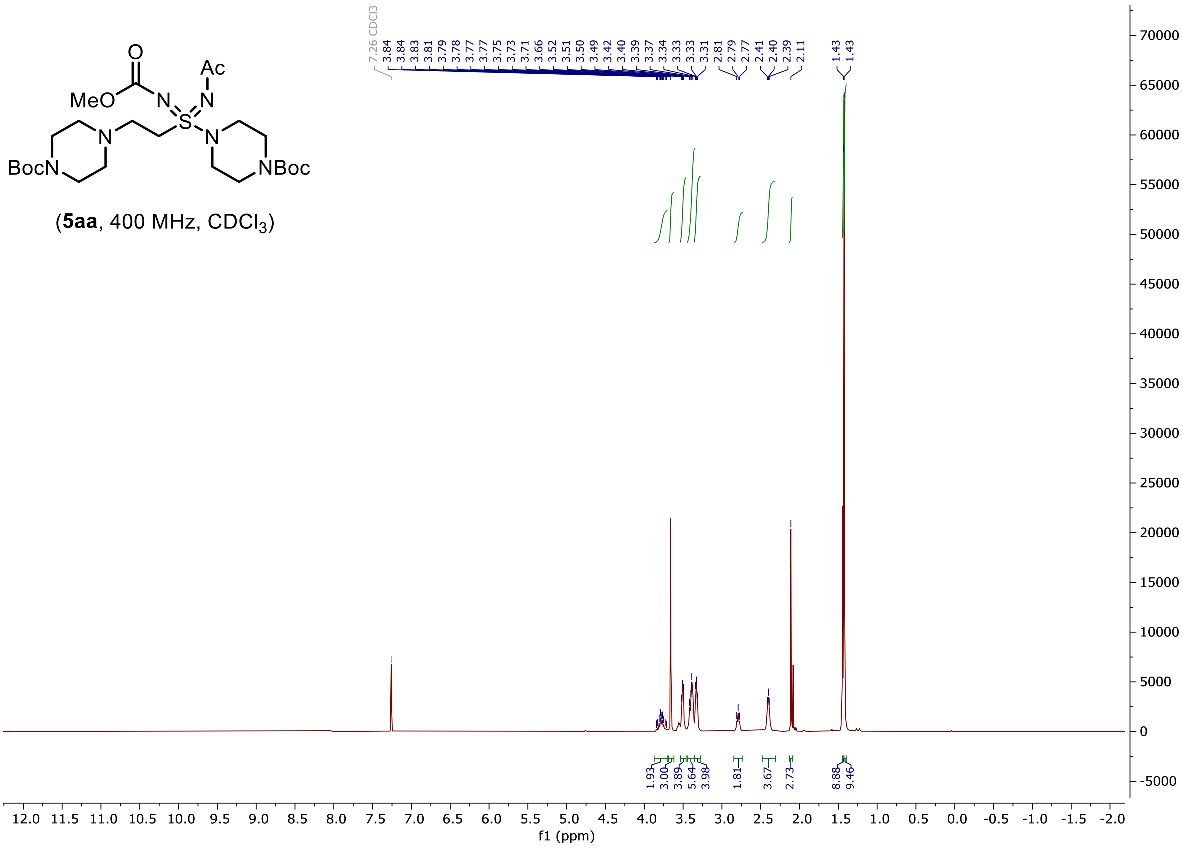

(**5aa**, 400 MHz, $CDCl_3$)

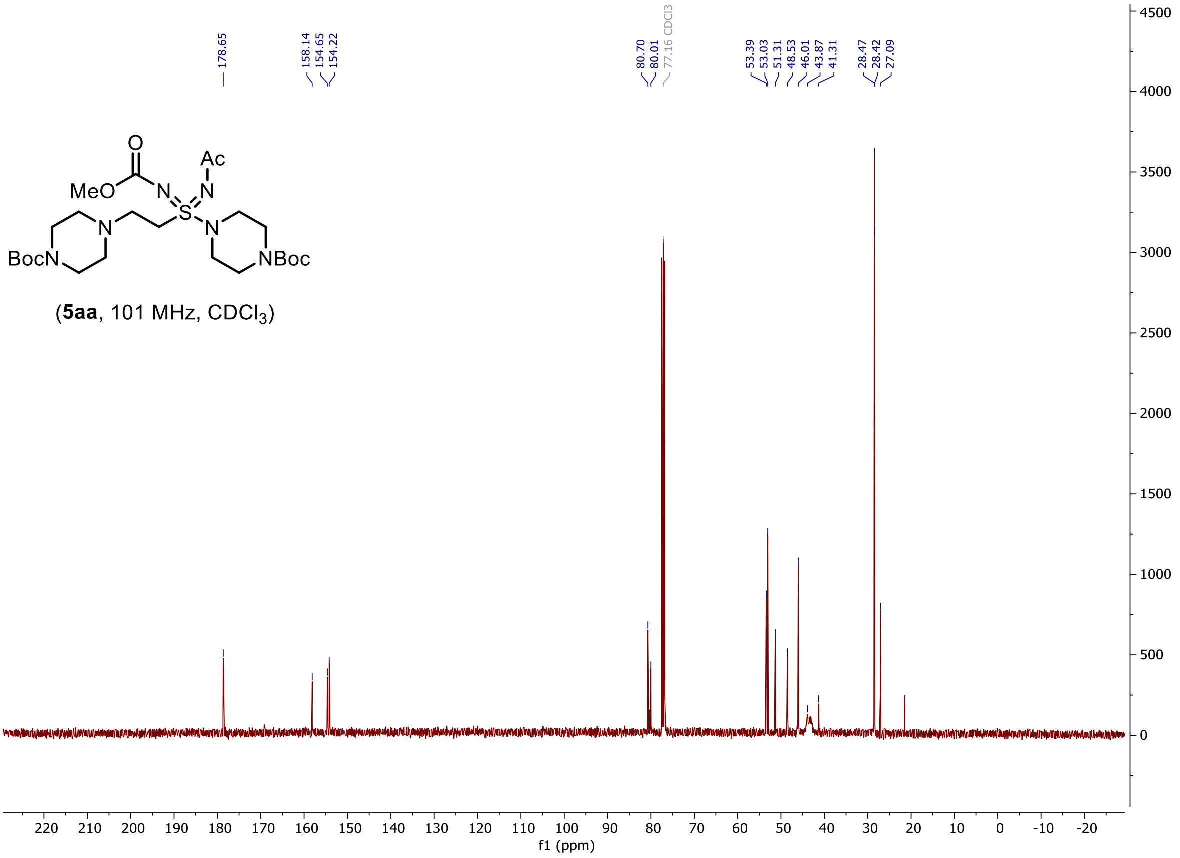

(**5aa**, 101 MHz, $CDCl_3$)

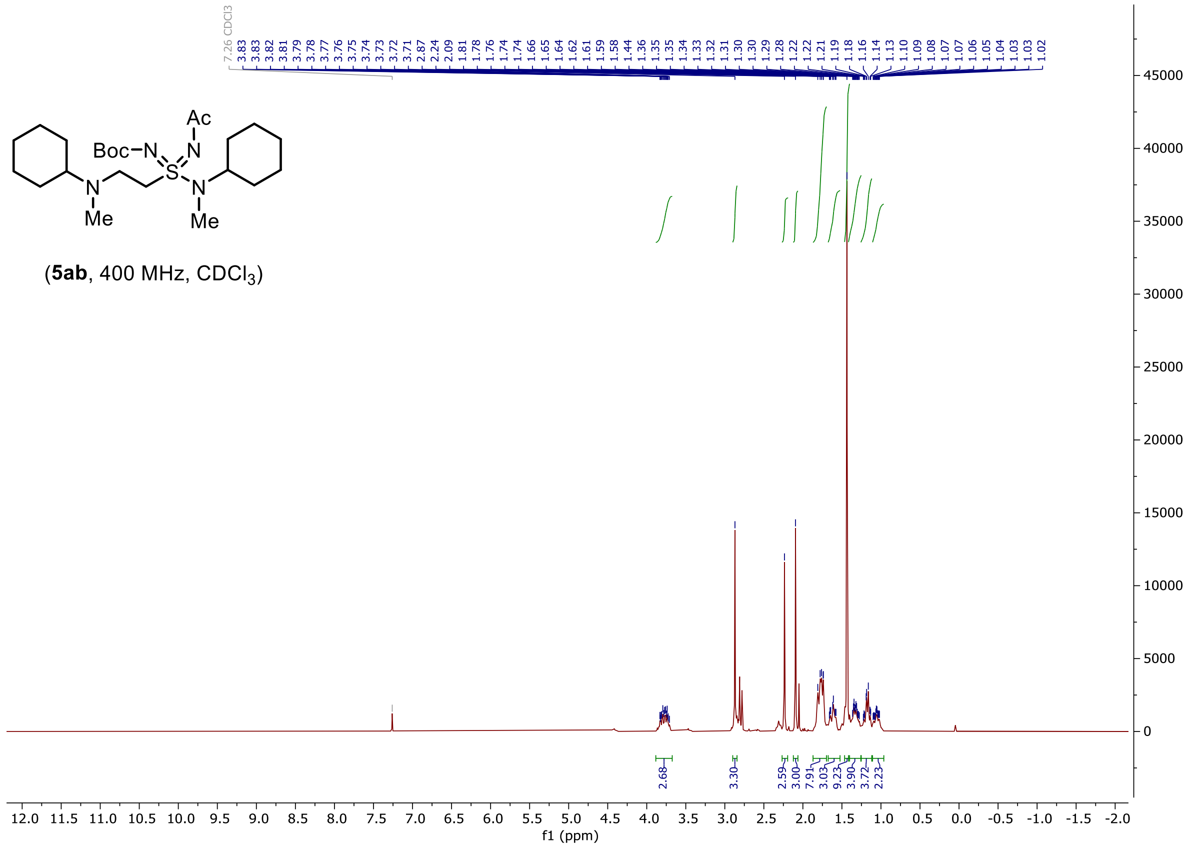

(**5ab**, 400 MHz, $CDCl_3$)

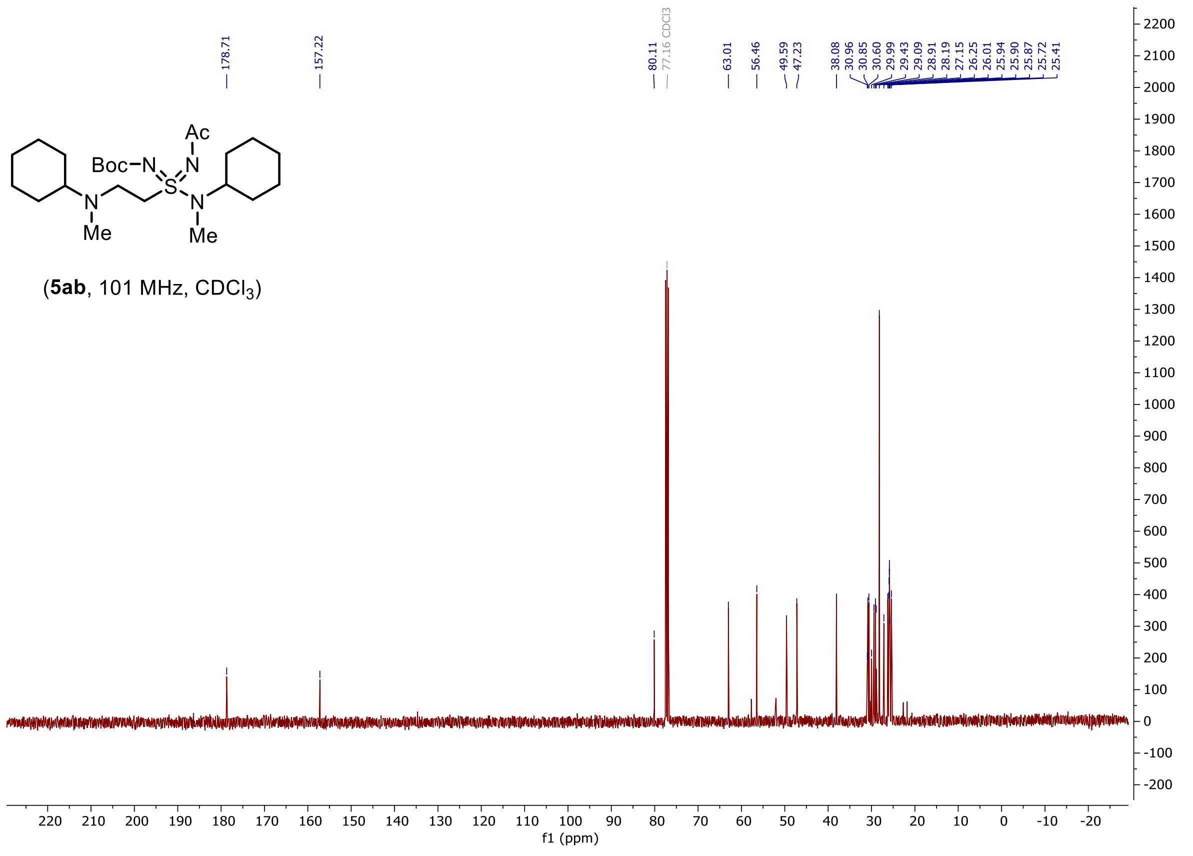

(**5ab**, 101 MHz, $CDCl_3$)

(**5ac**, 400 MHz, $CDCl_3$)

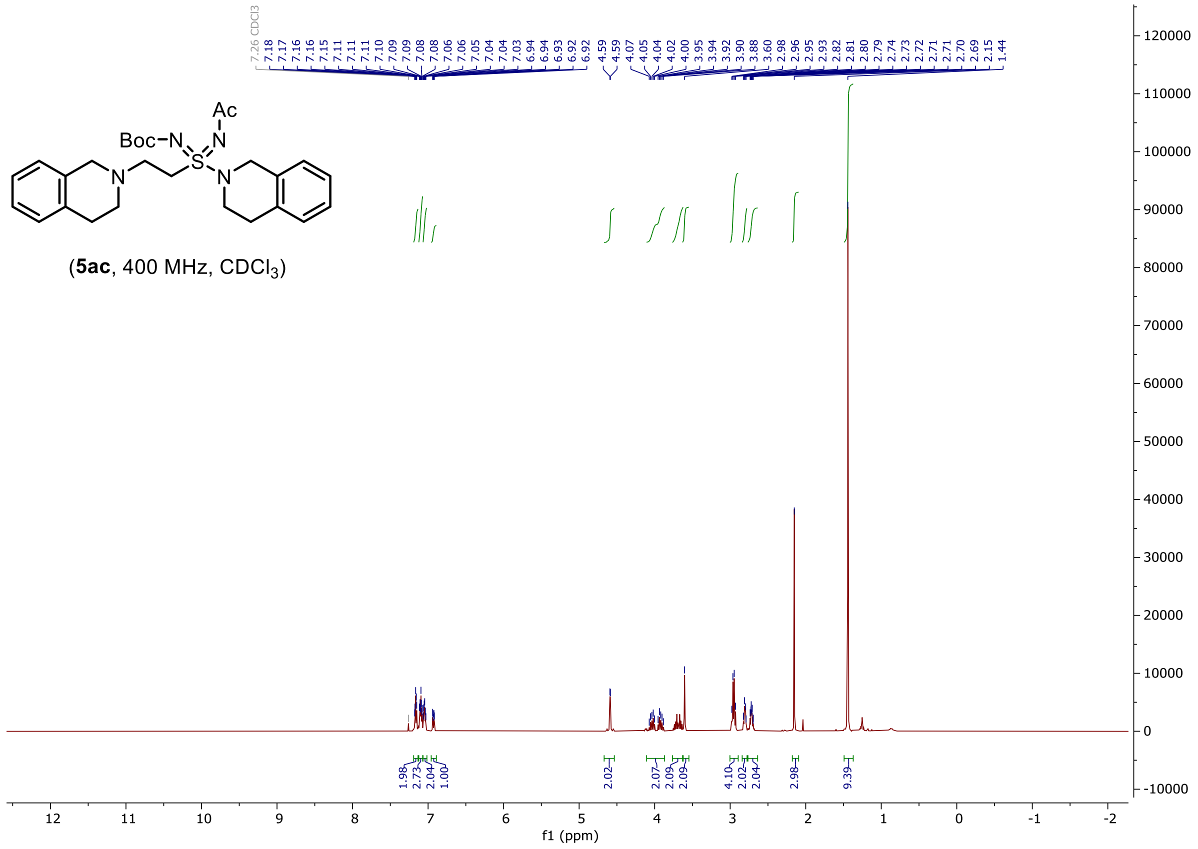

(**5ac**, 101 MHz, $CDCl_3$)

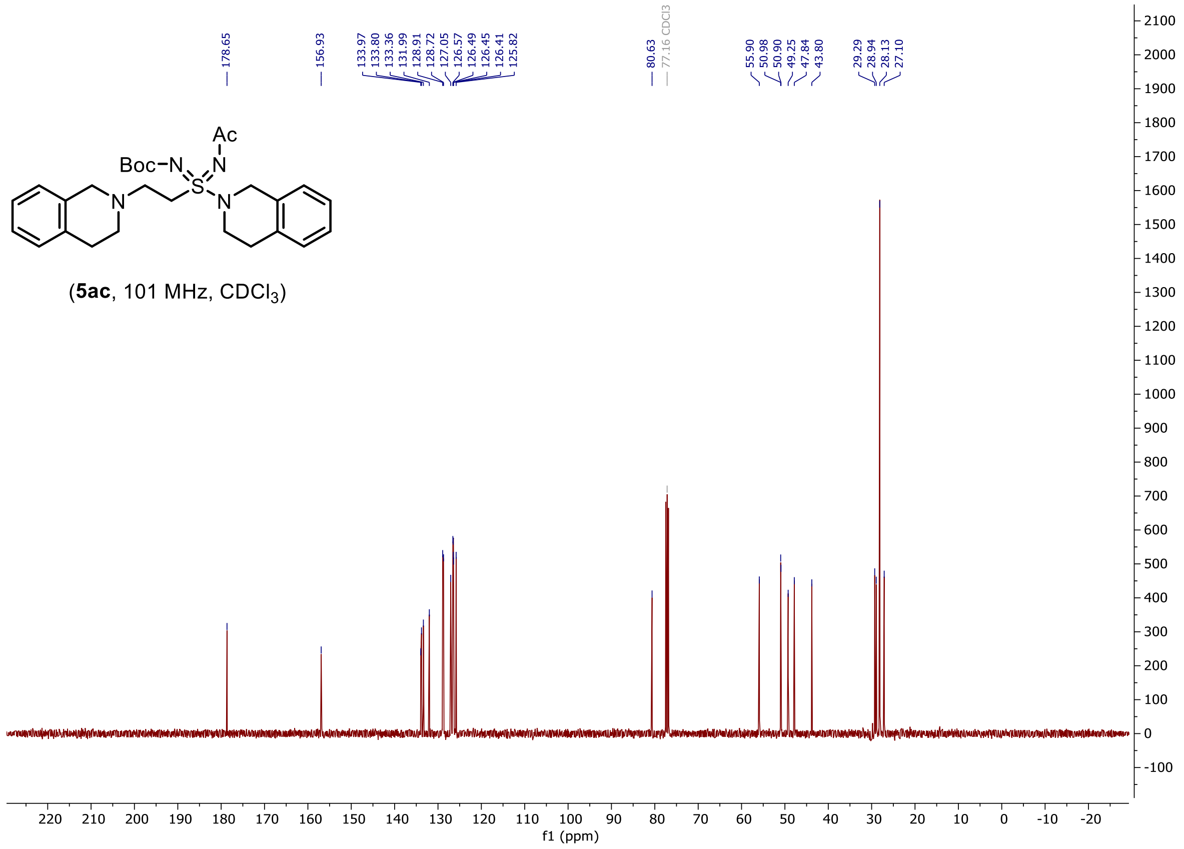

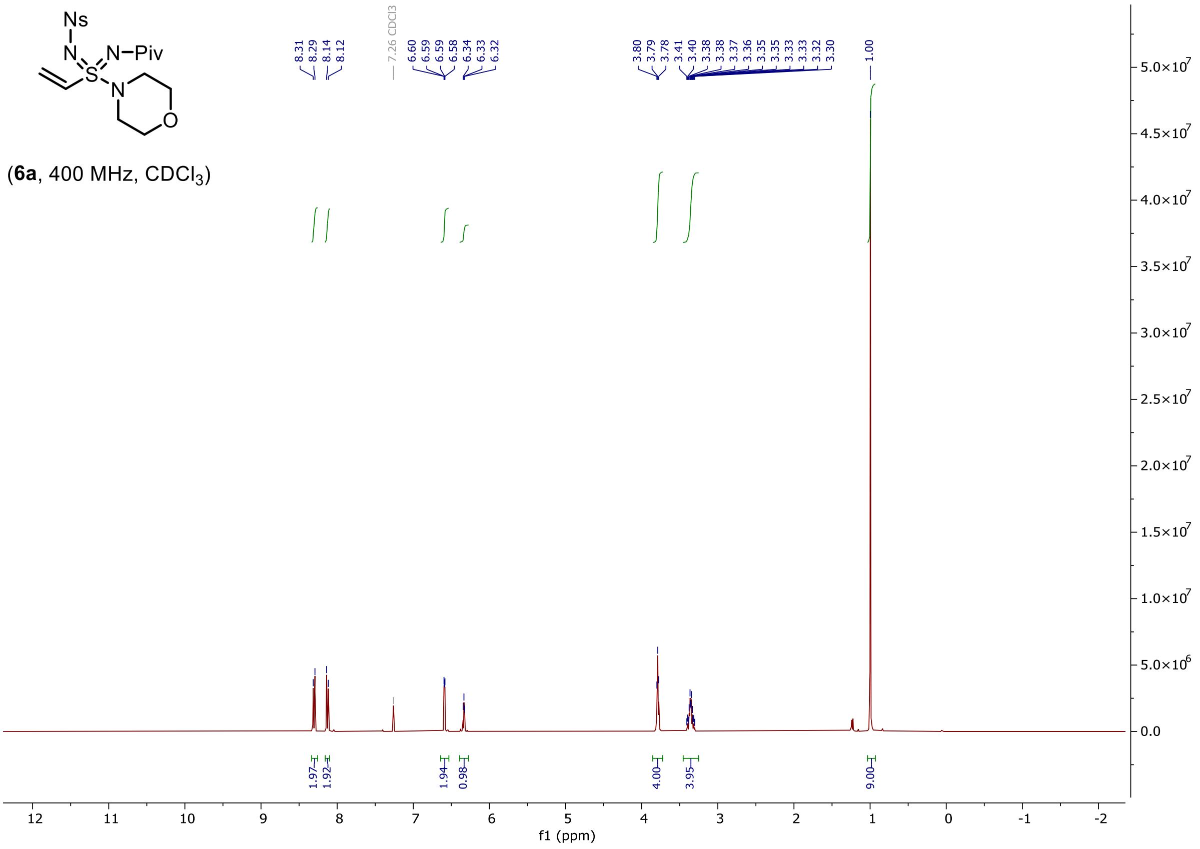
Ns
N
N−Piv
S
N
O
(6a, 400 MHz, CDCl3)
8.31 8.29 8.14 8.12
7.26 CDCl3
6.60 6.59 6.59 6.58 6.34 6.33 6.32
3.80 3.79 3.78 3.41 3.40 3.38 3.38 3.37 3.36 3.35 3.35 3.33 3.33 3.32 3.30
1.00
1.97 1.92 1.94 0.98 4.00 3.95 9.00
f1 (ppm)

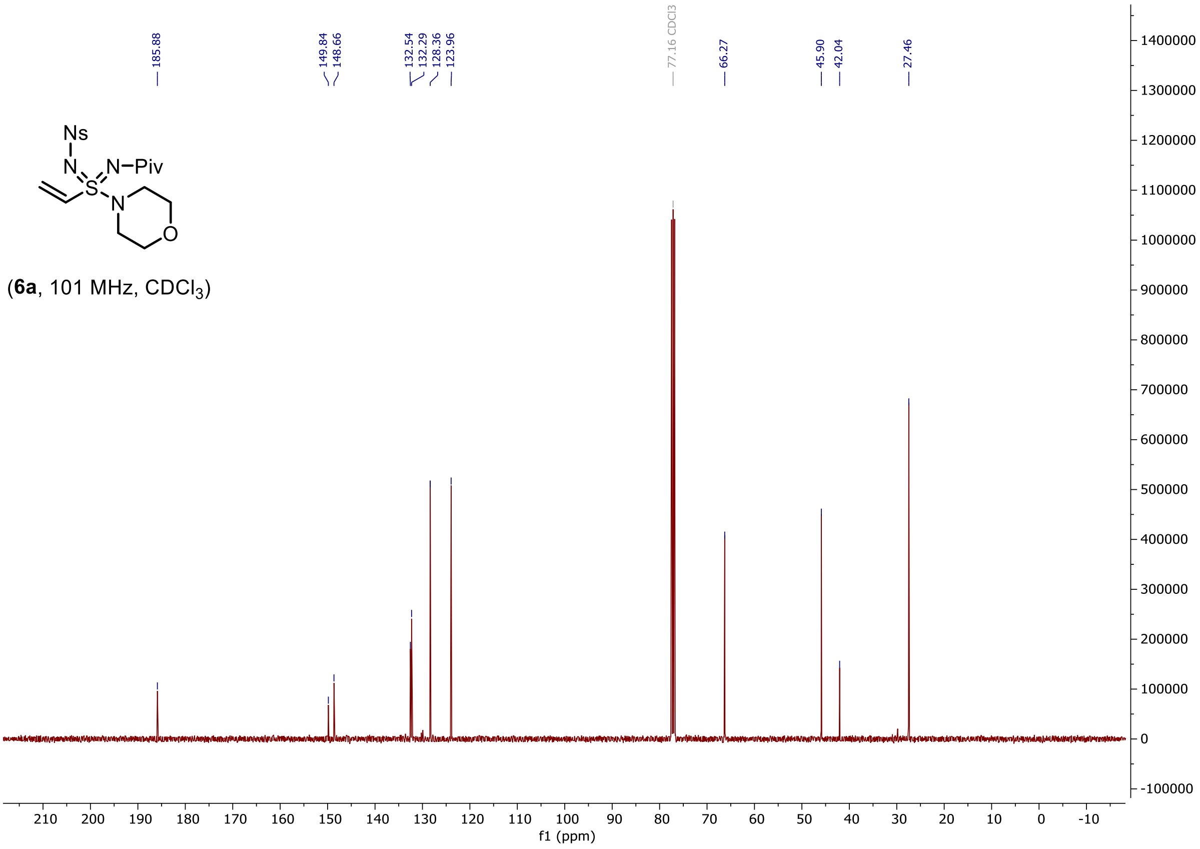
Ns
N
N−Piv
S
N
O
(6a, 101 MHz, CDCl3)
185.88
149.84 148.66
132.54 132.29 128.36 123.96
77.16 CDCl3
66.27
45.90 42.04
27.46
f1 (ppm)

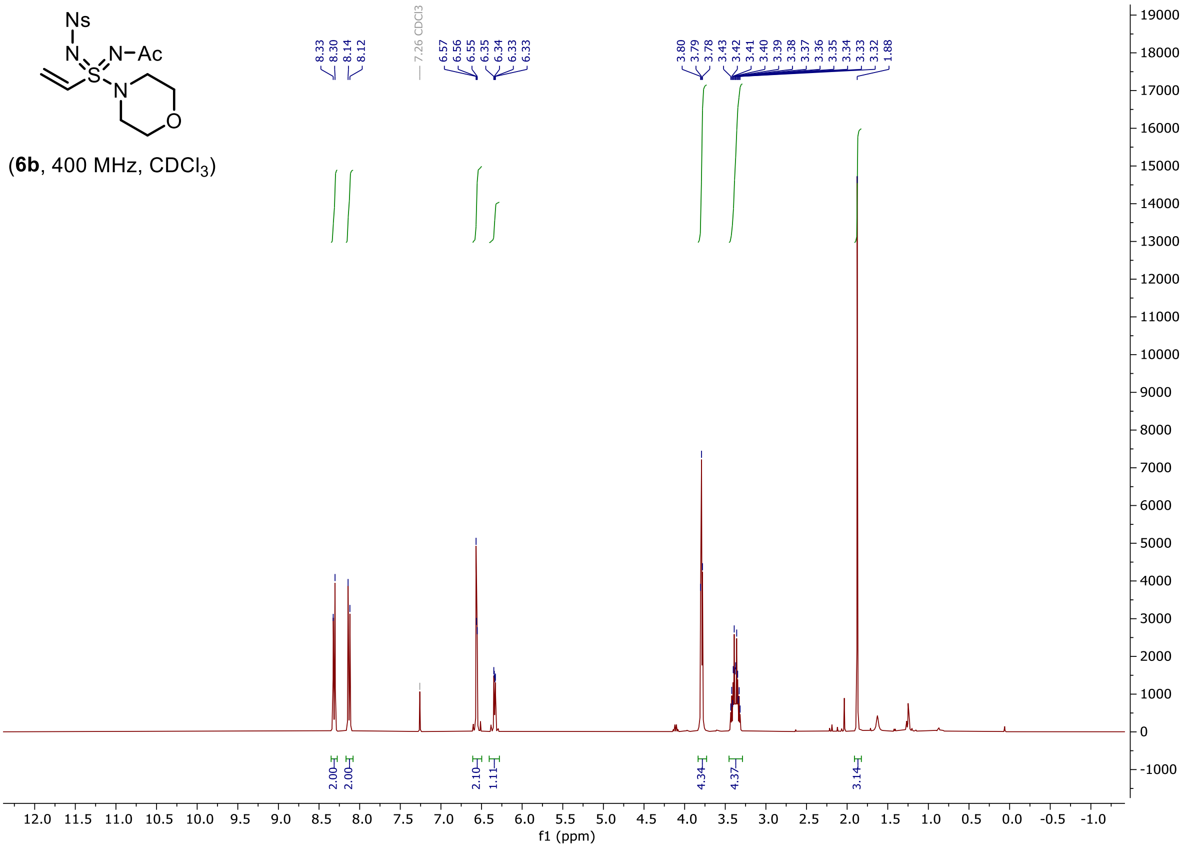

(**6b**, 400 MHz, $CDCl_3$)

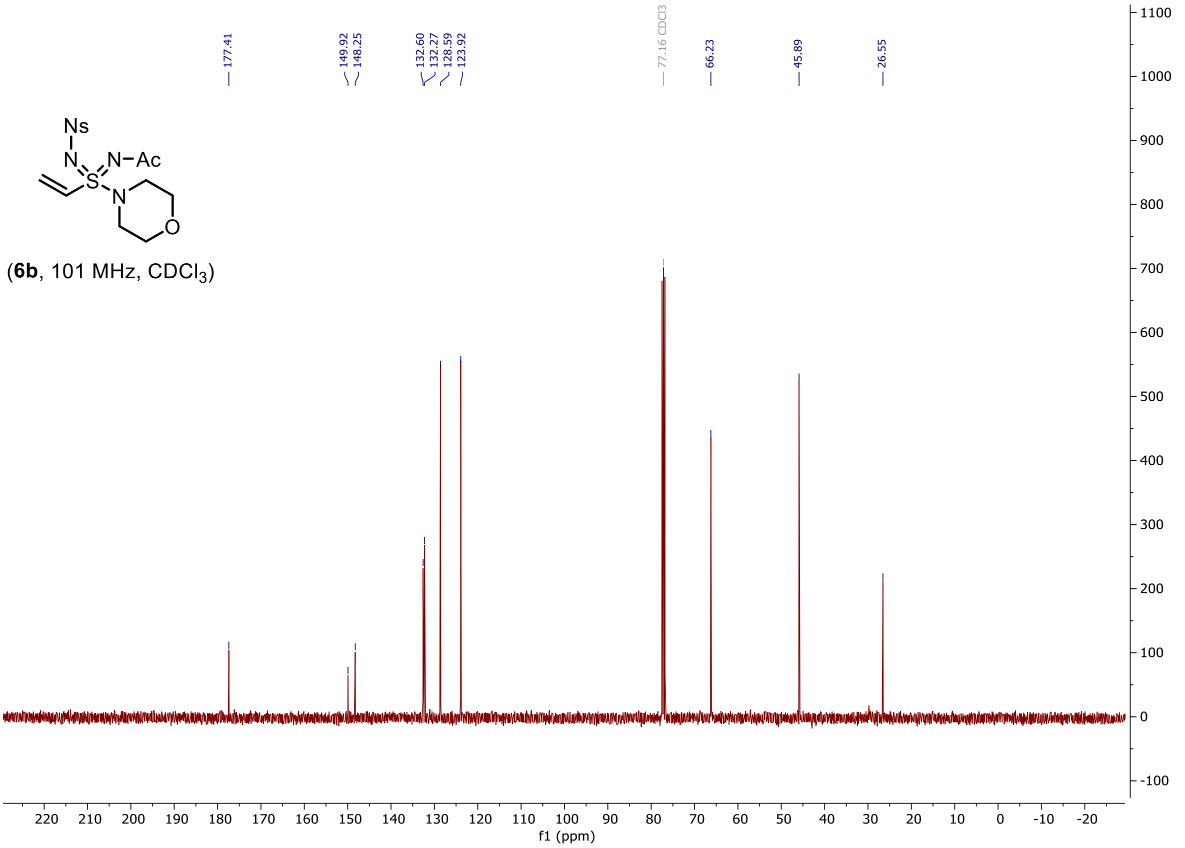

(**6b**, 101 MHz, $CDCl_3$)

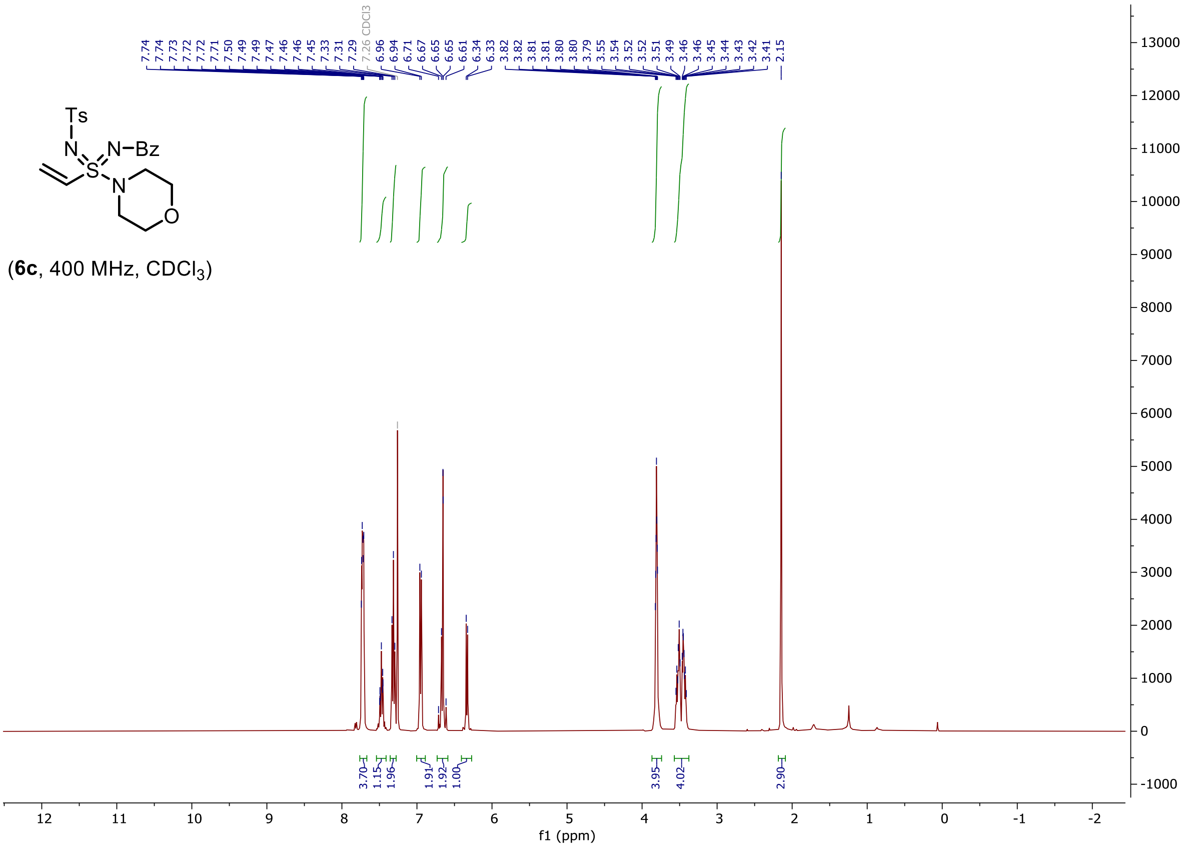

(**6c**, 400 MHz, $CDCl_3$)

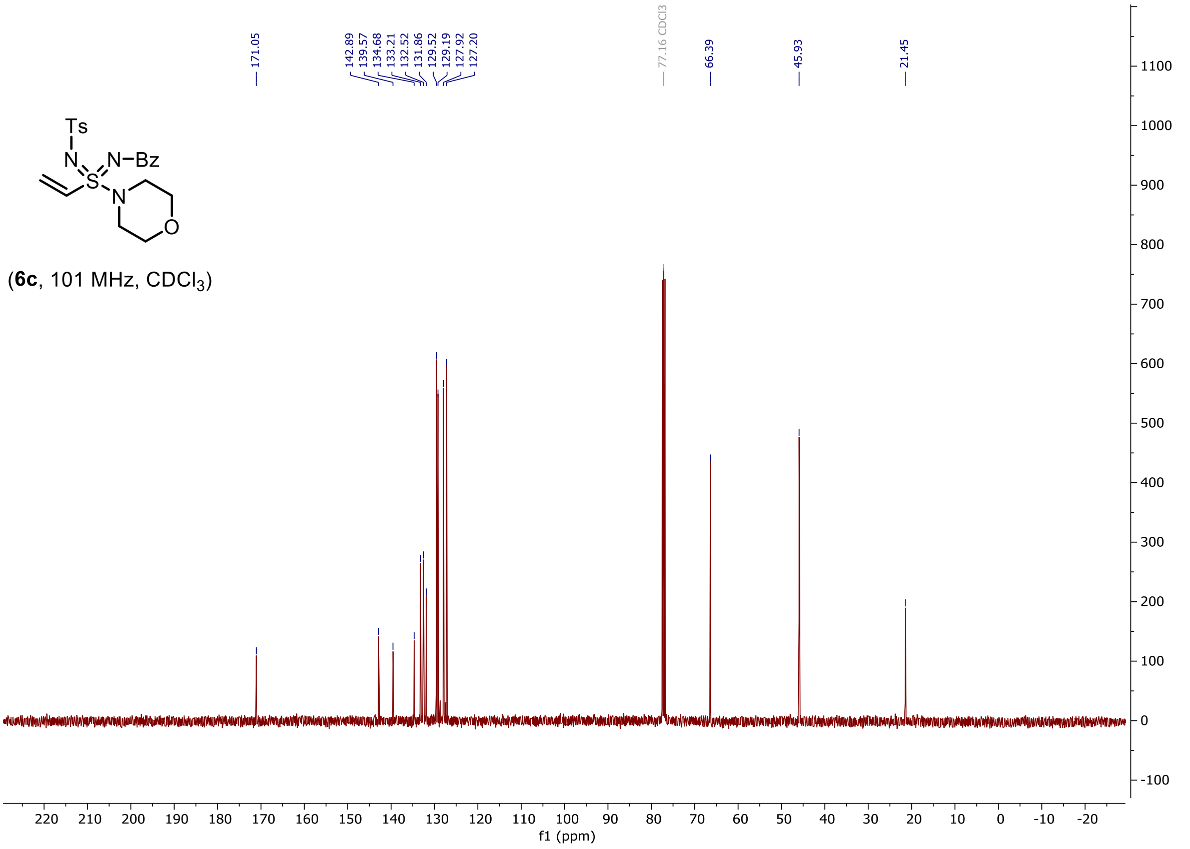

(**6c**, 101 MHz, $CDCl_3$)

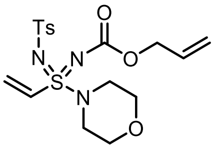

(**6d**, 400 MHz, $CDCl_3$)

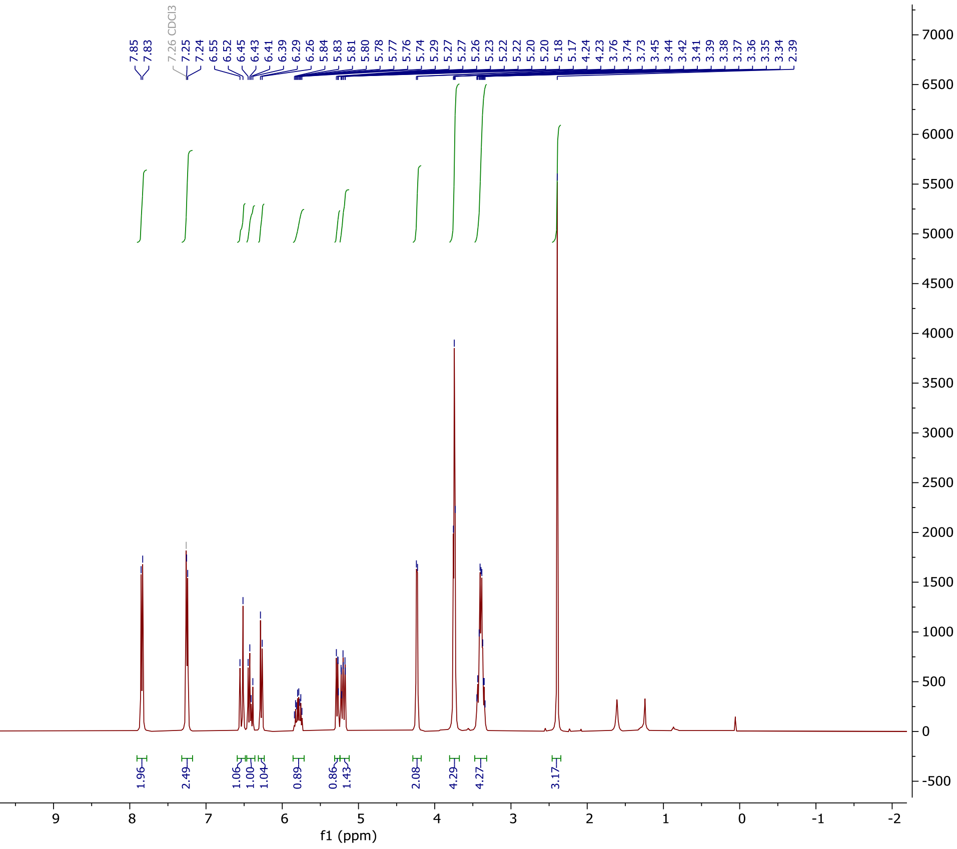

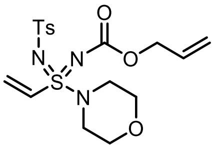

(**6d**, 101 MHz, $CDCl_3$)

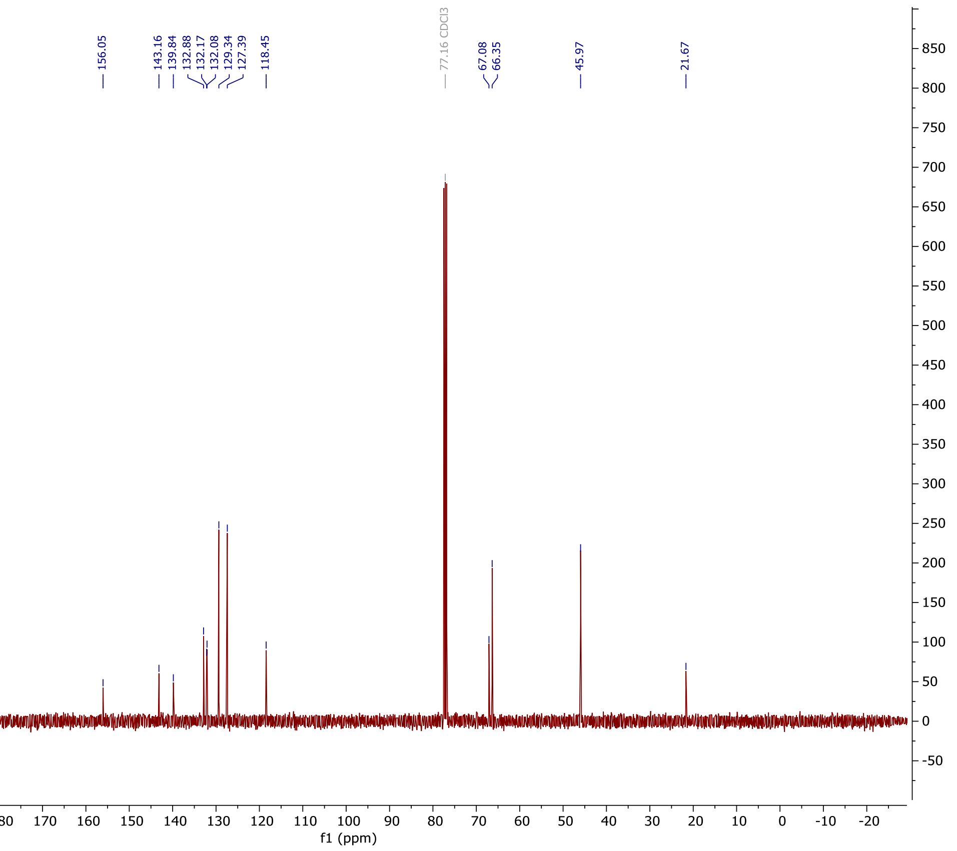

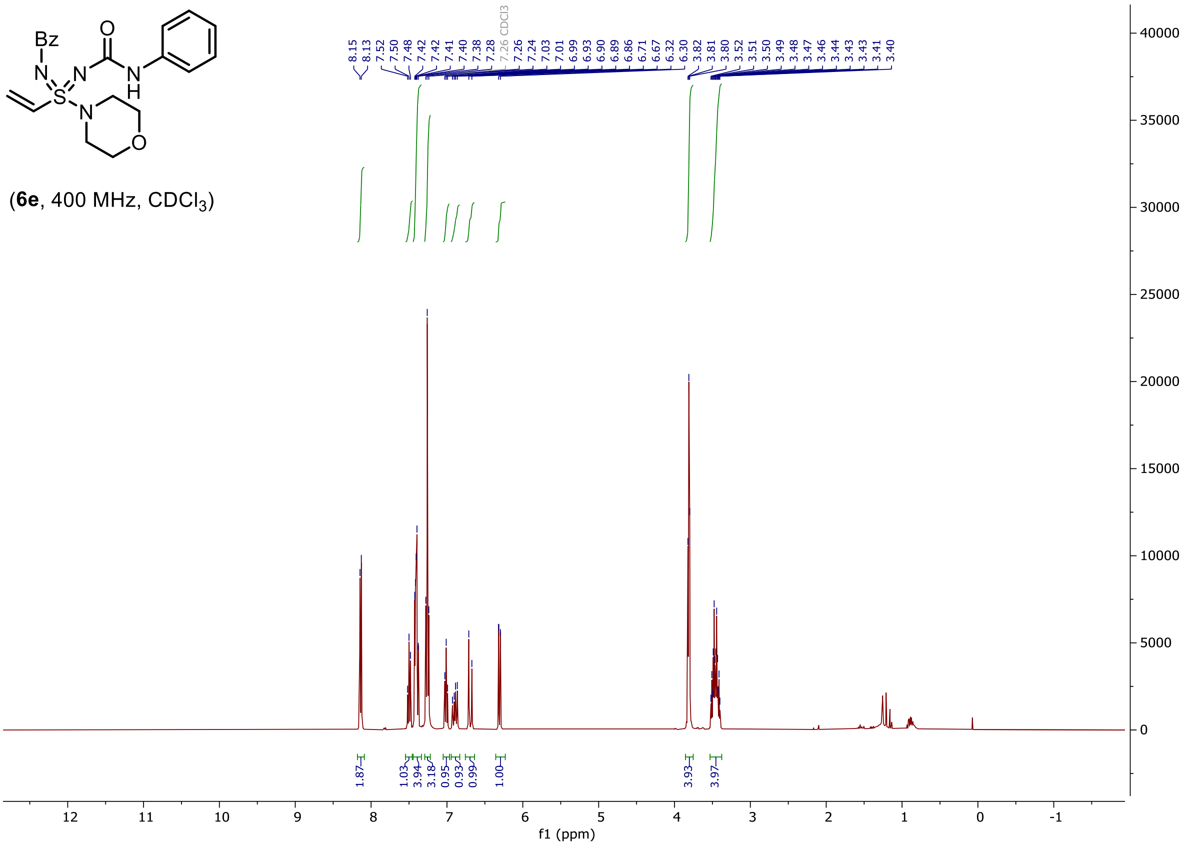

(**6e**, 400 MHz, $CDCl_3$)

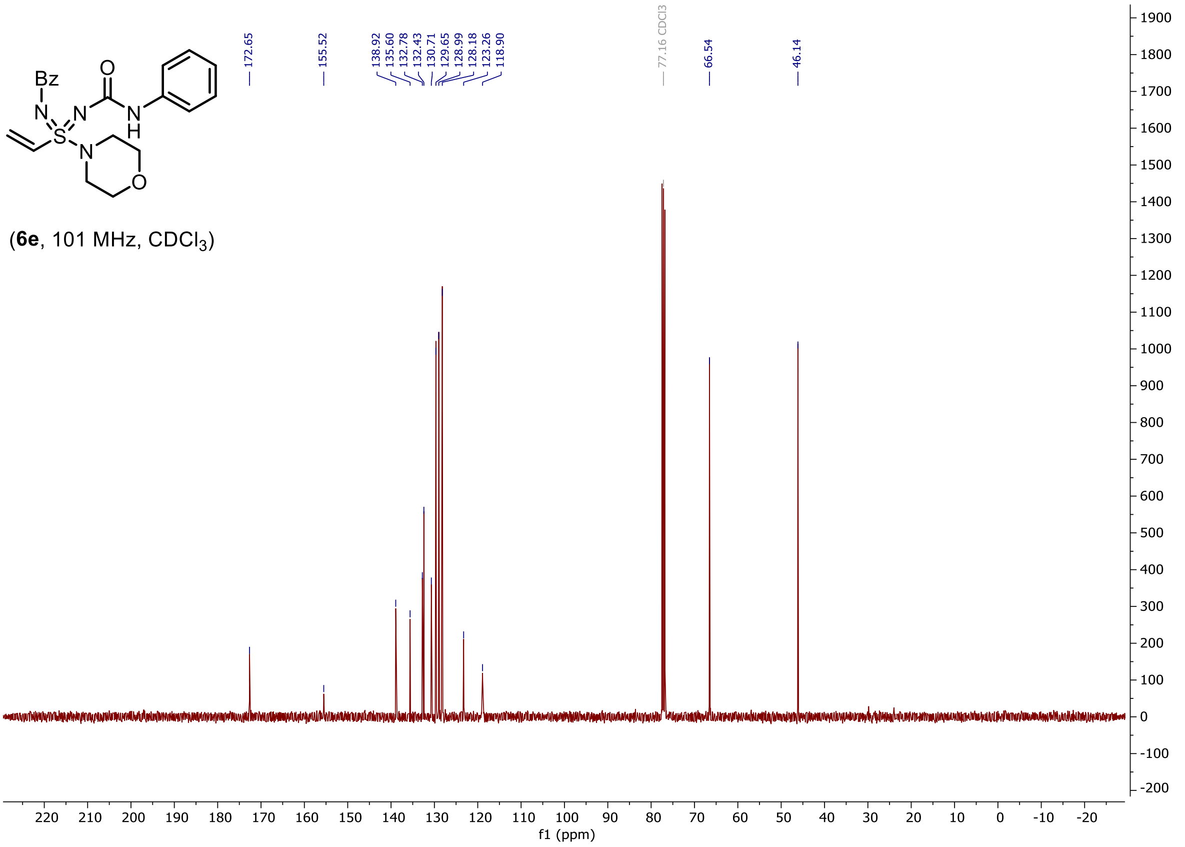

(**6e**, 101 MHz, $CDCl_3$)

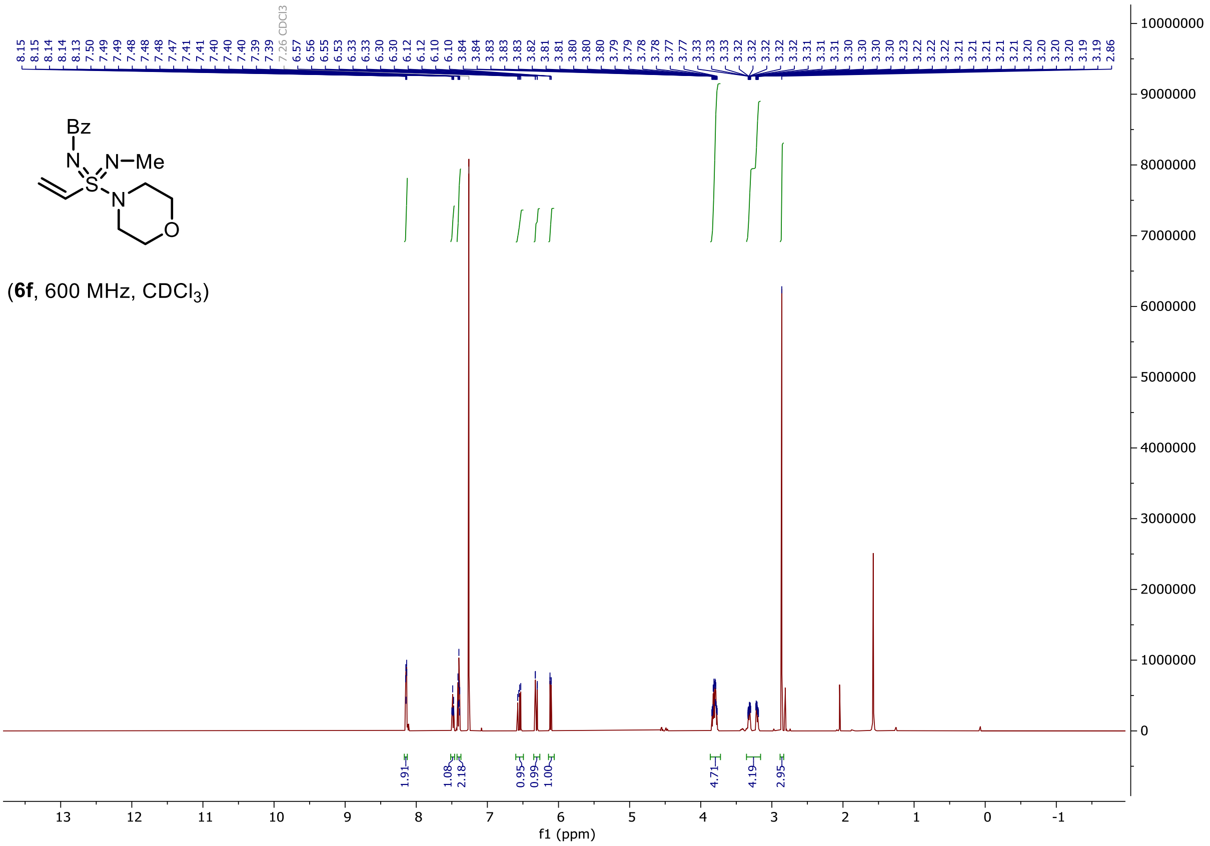

(**6f**, 600 MHz, $CDCl_3$)

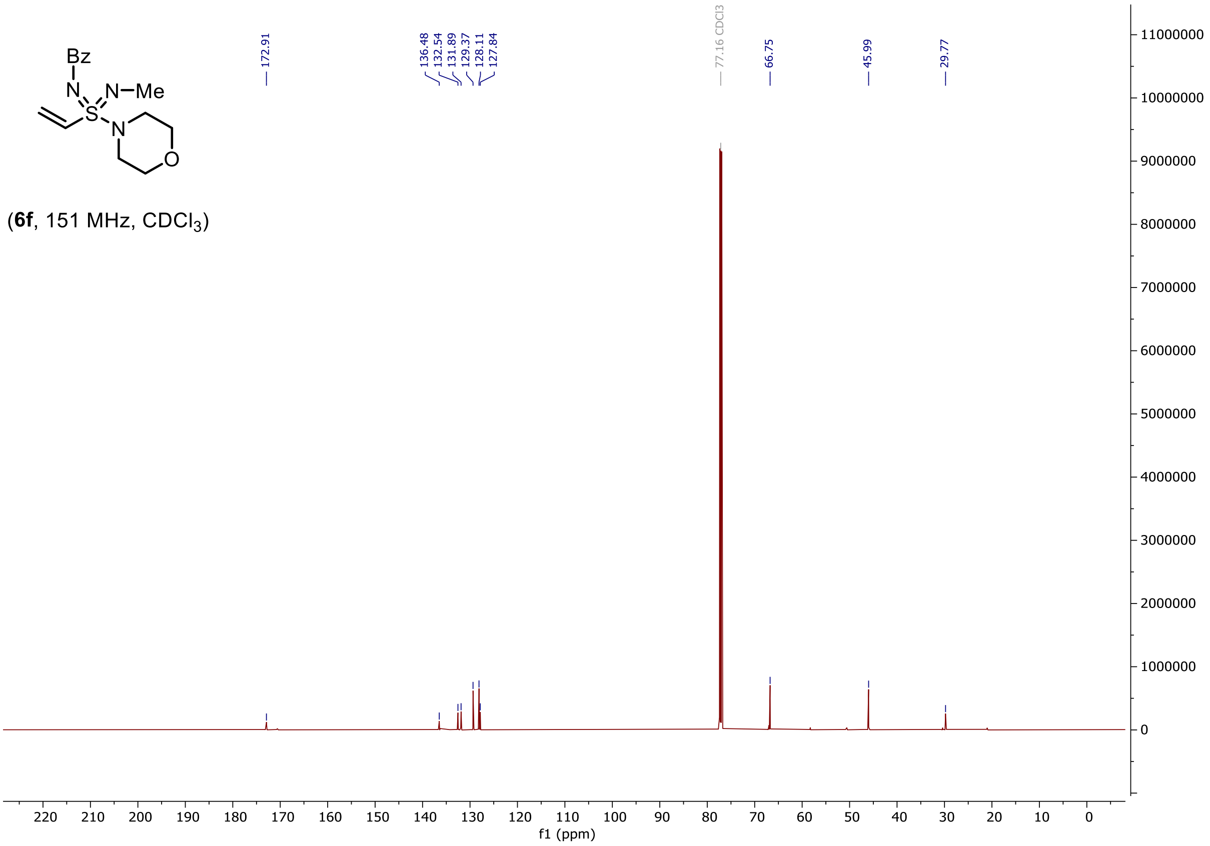

(**6f**, 151 MHz, $CDCl_3$)

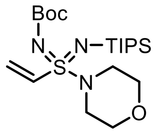

(**6g**, 400 MHz, $CDCl_3$)

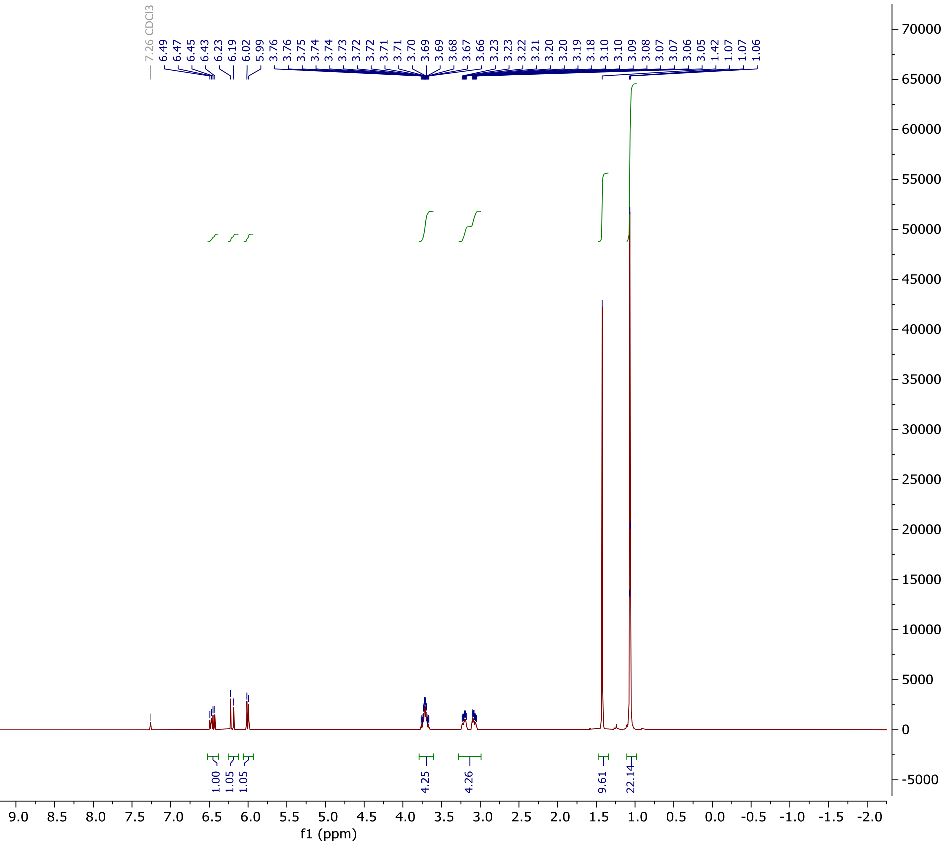

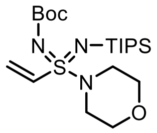

(**6g**, 101 MHz, $CDCl_3$)

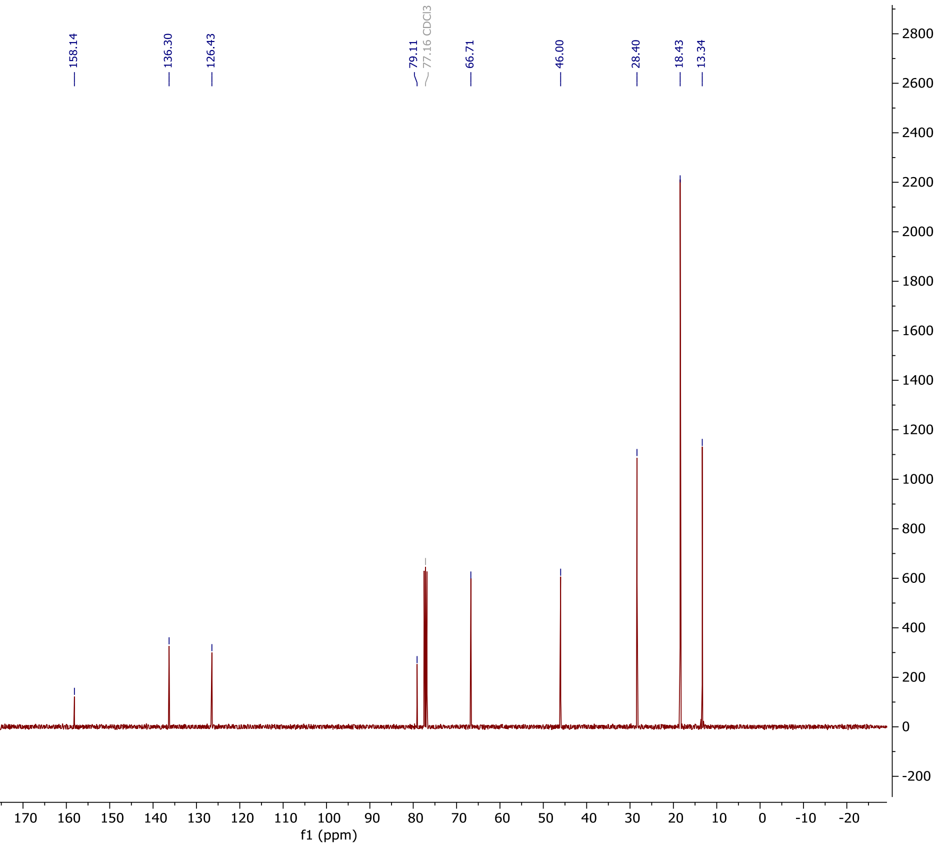

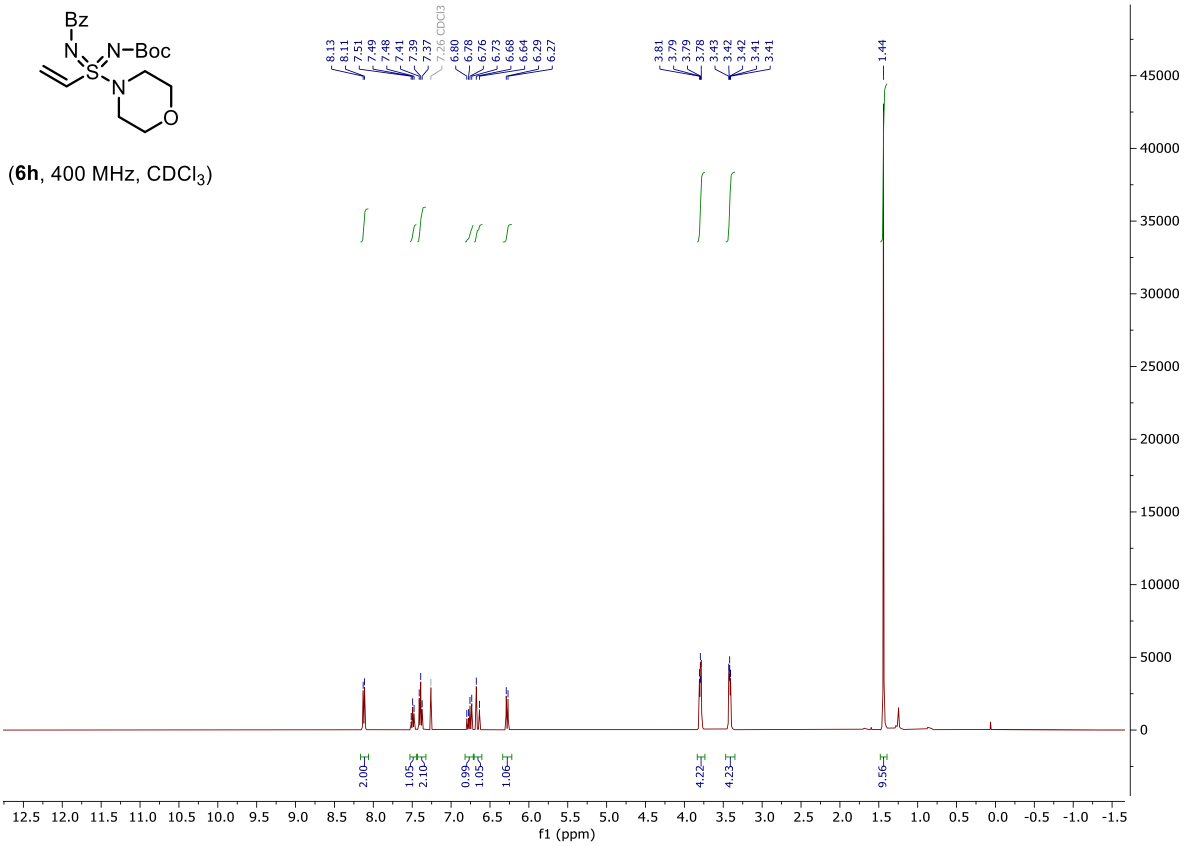

(**6h**, 400 MHz, $CDCl_3$)

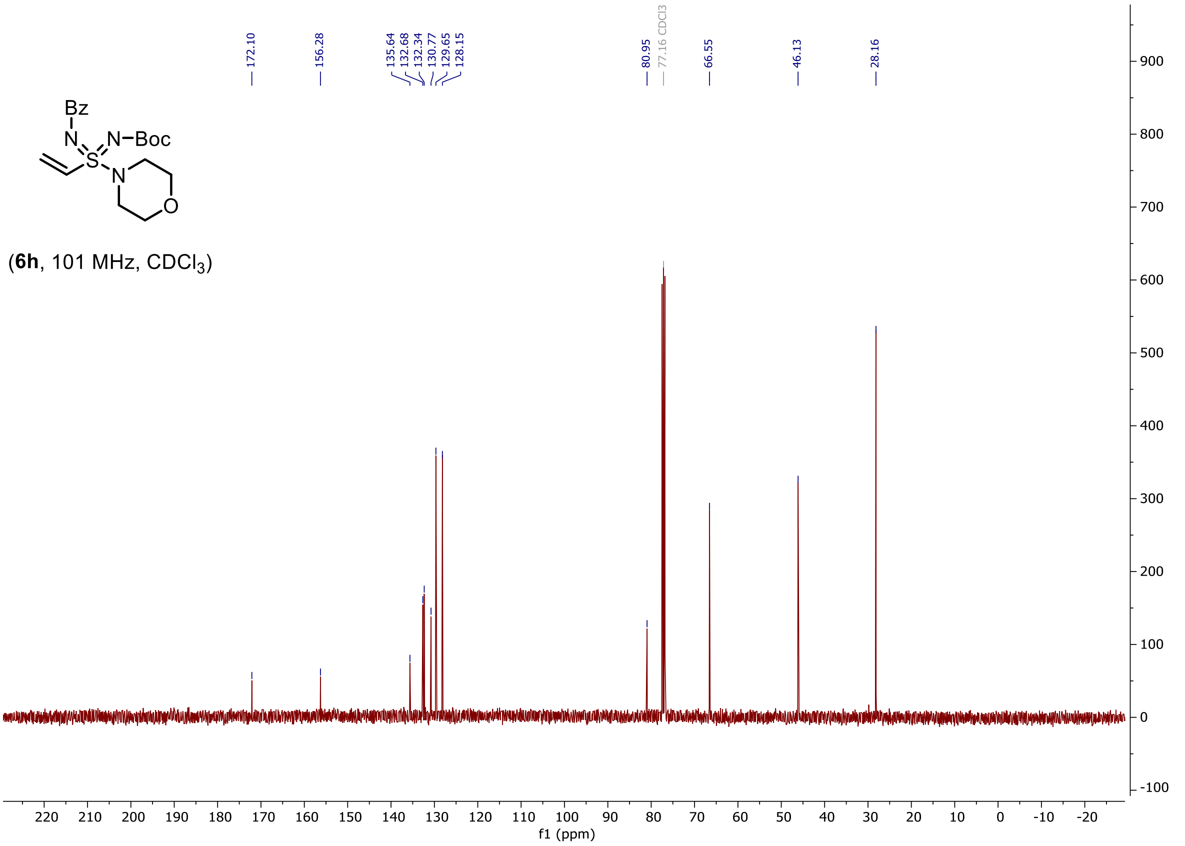

(**6h**, 101 MHz, $CDCl_3$)

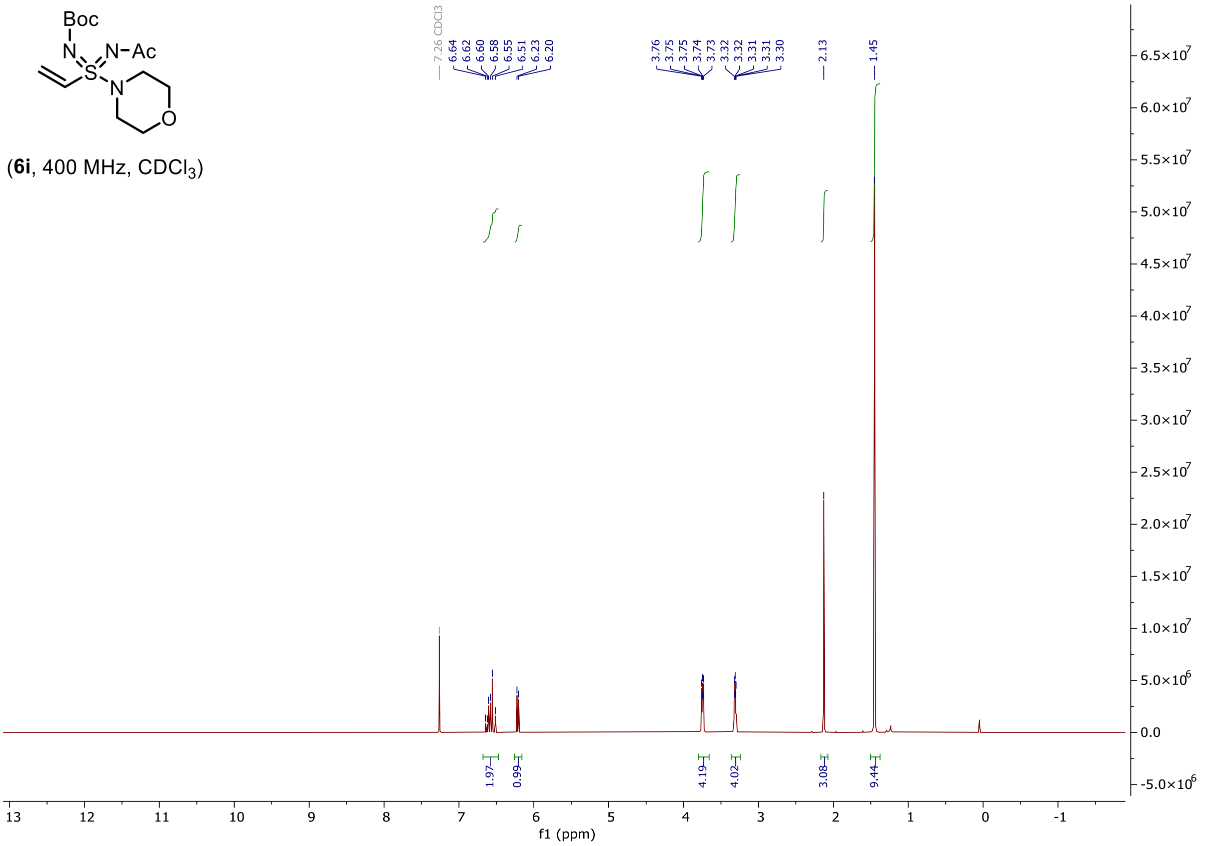

(**6i**, 400 MHz, $CDCl_3$)

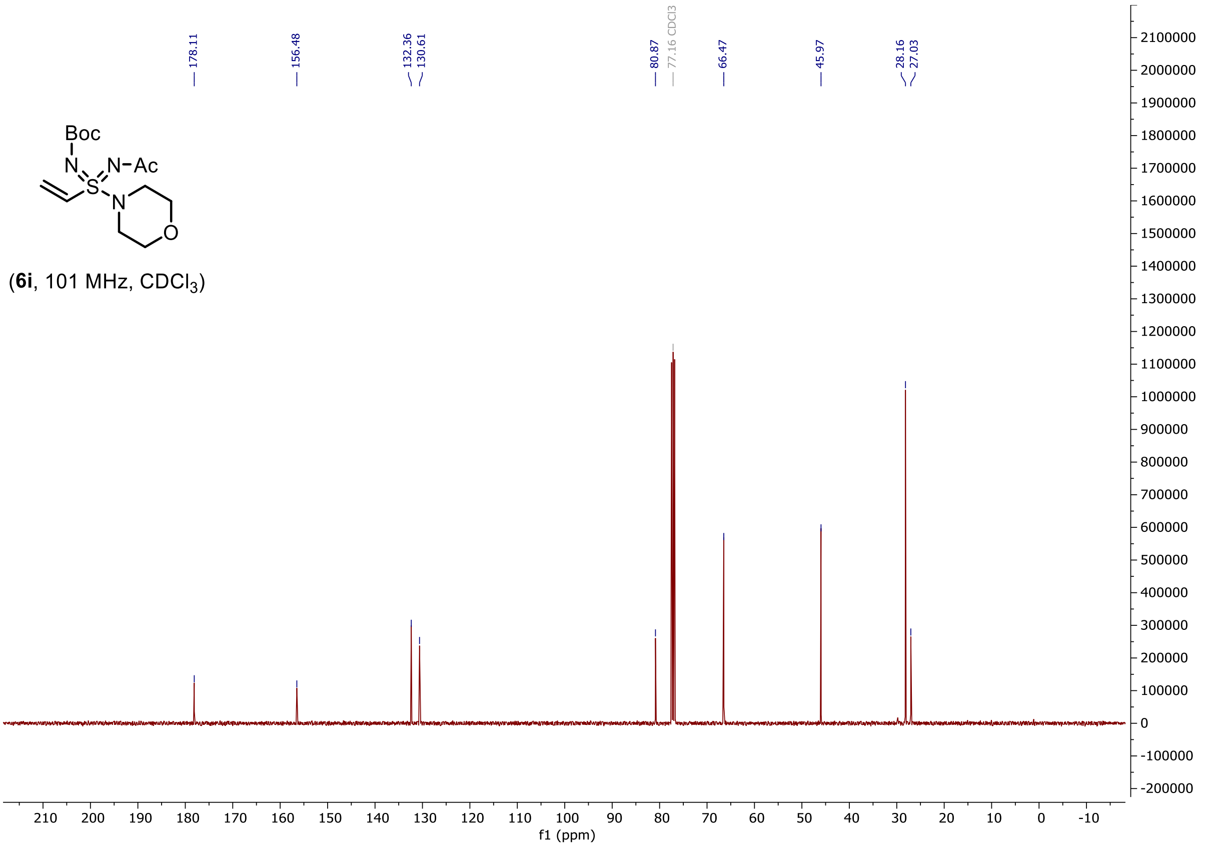

(**6i**, 101 MHz, $CDCl_3$)

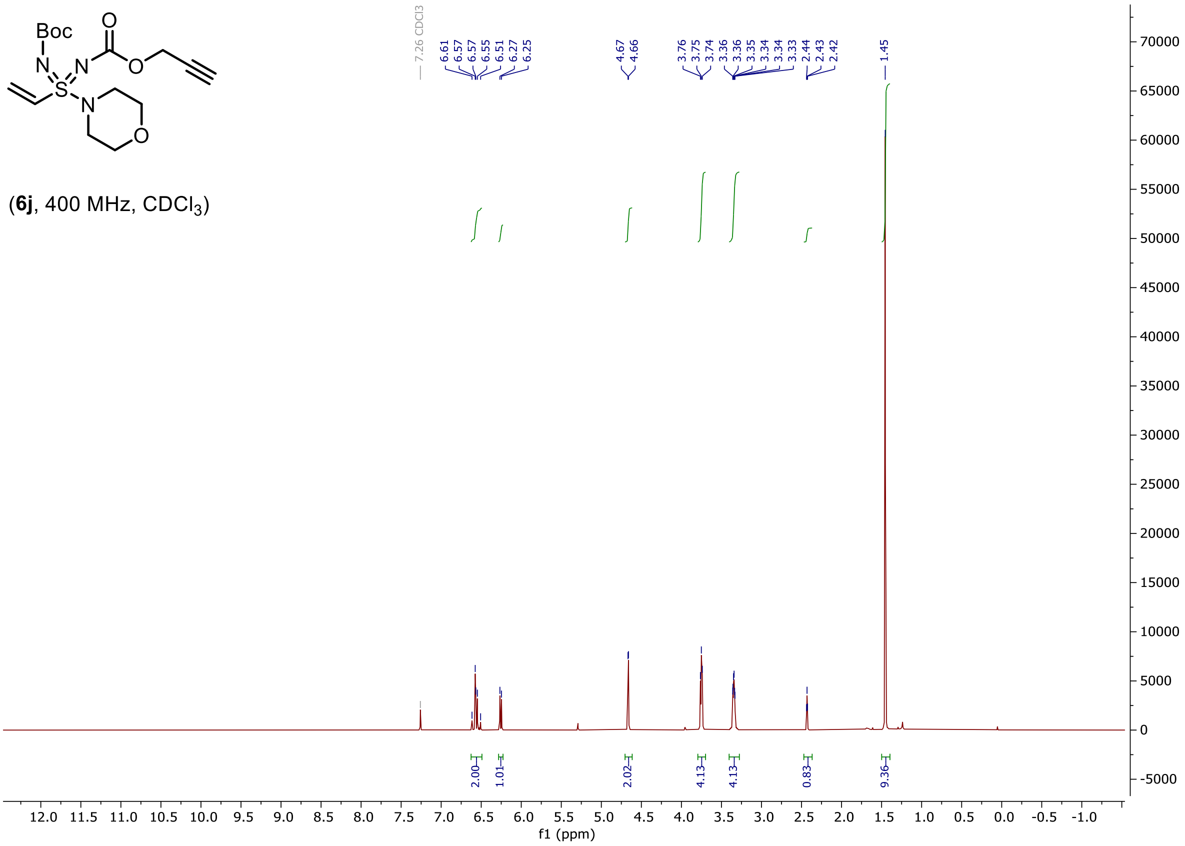

(**6j**, 400 MHz, $CDCl_3$)

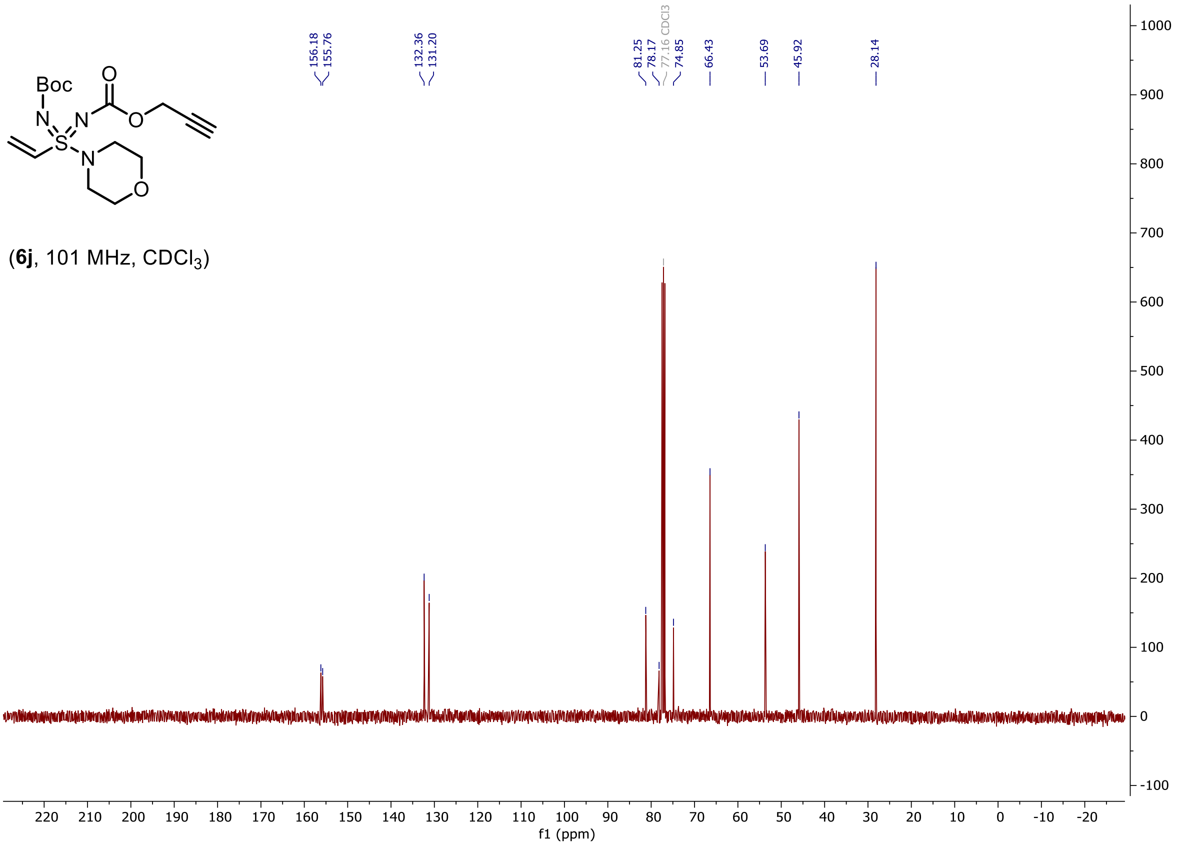

(**6j**, 101 MHz, $CDCl_3$)

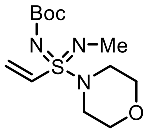

(**6k**, 400 MHz, $CDCl_3$)

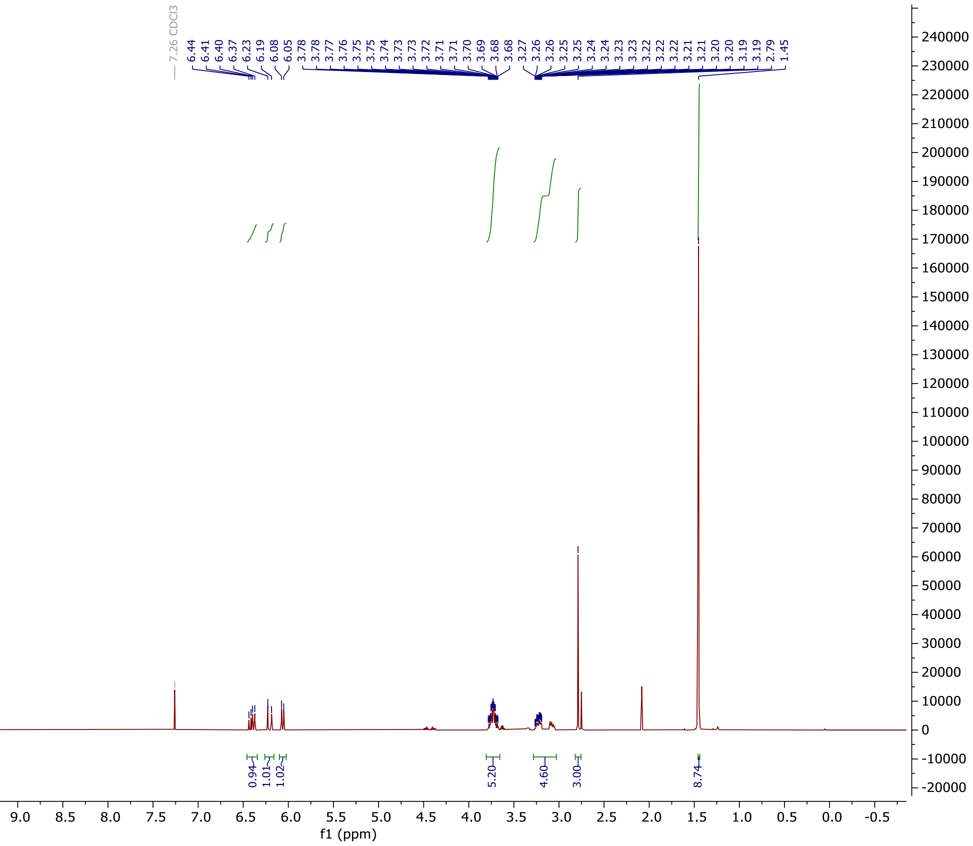

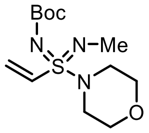

(**6k**, 101 MHz, $CDCl_3$)

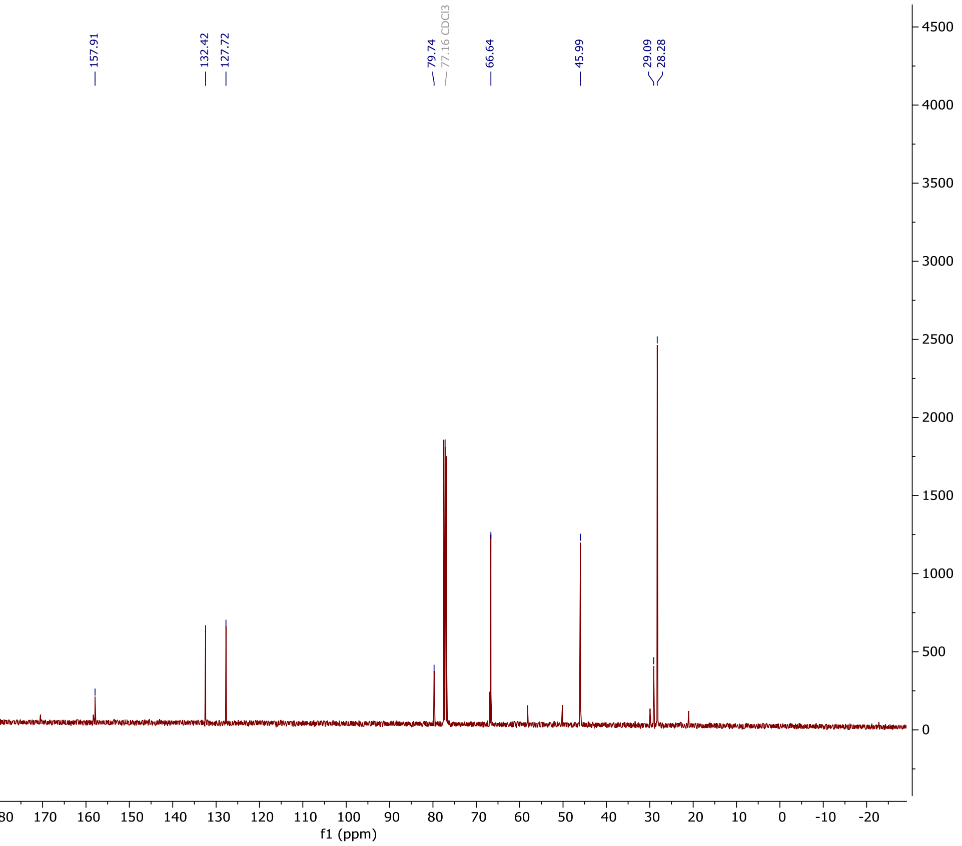

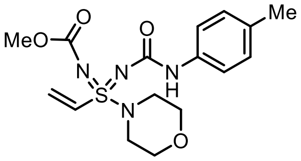

(**6l**, 400 MHz, $CDCl_3$)

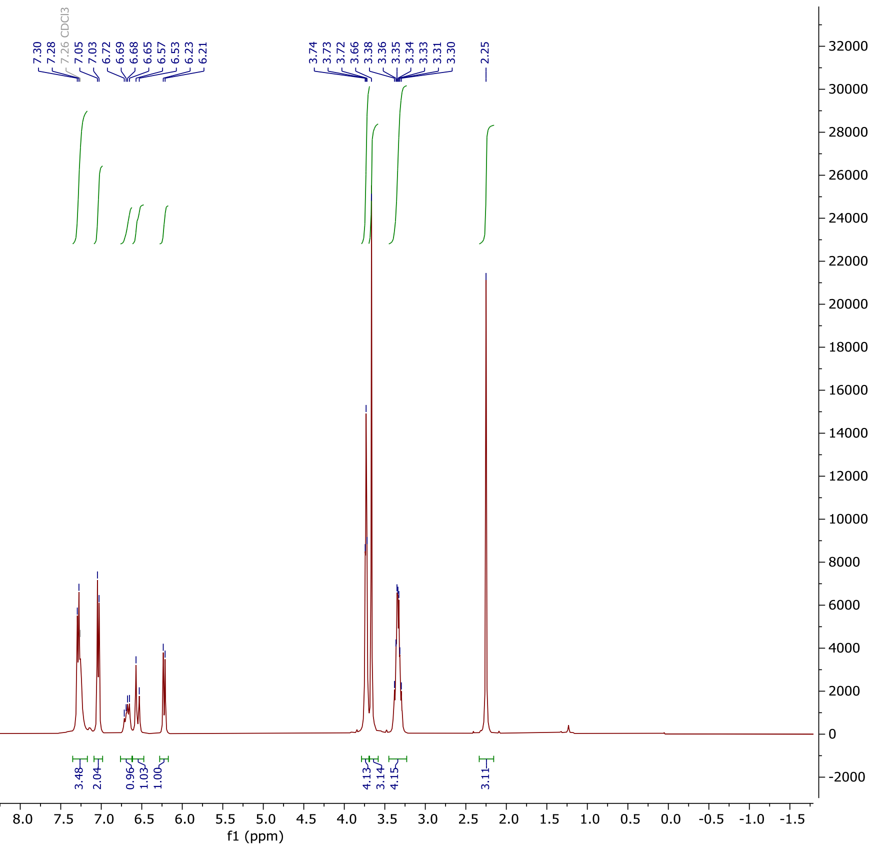

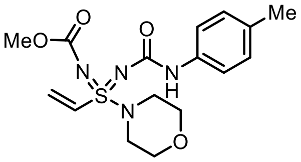

(**6l**, 101 MHz, $CDCl_3$)

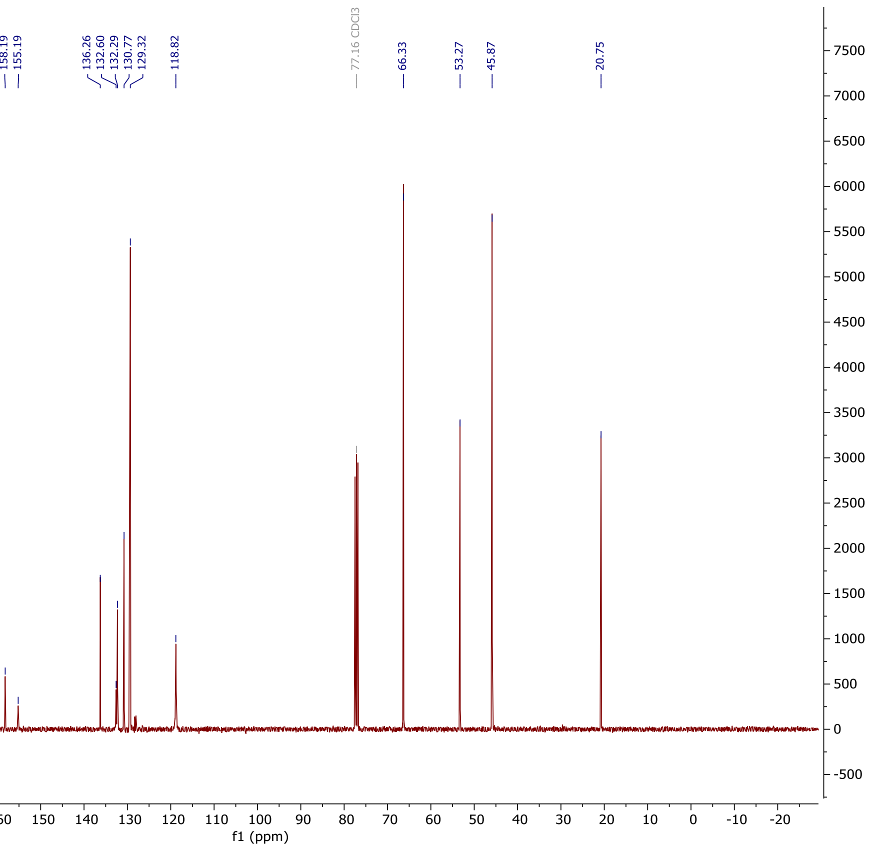

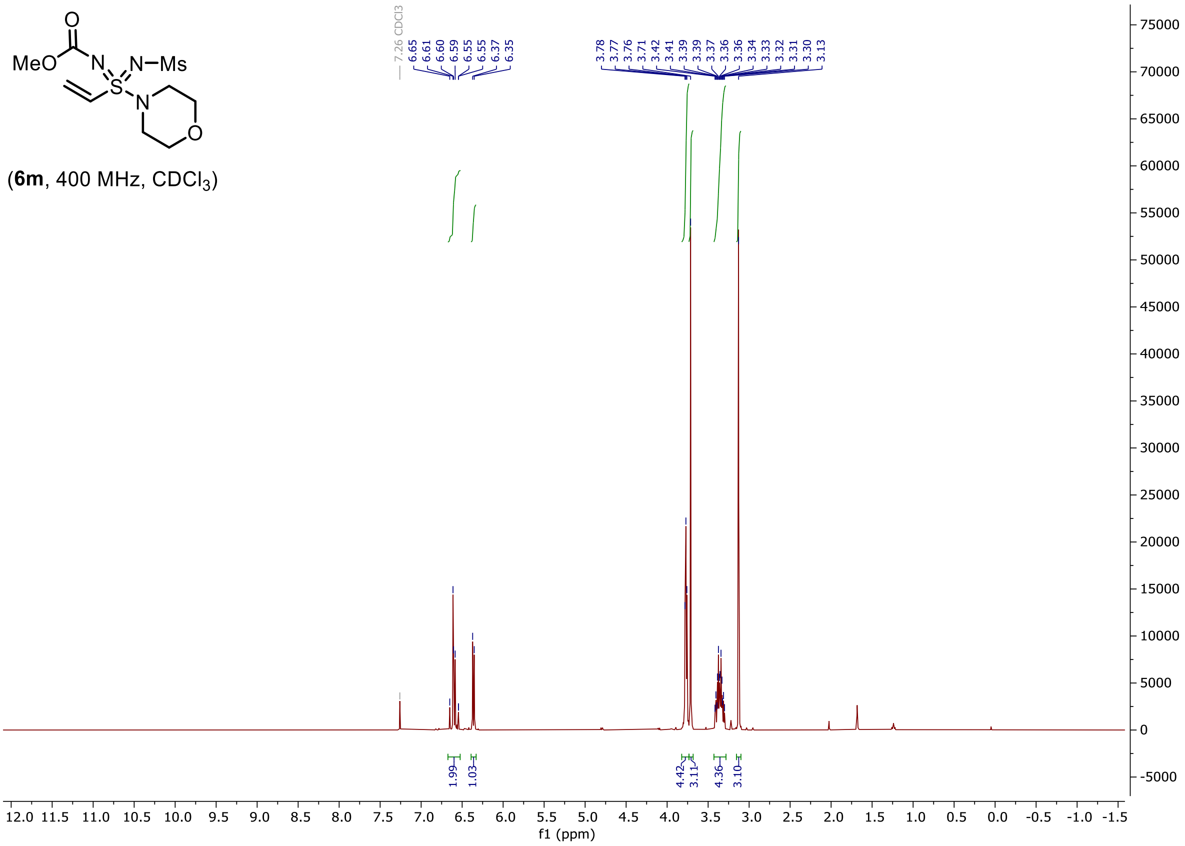

MeO
N
N−Ms
S
N
O
(6m, 400 MHz, CDCl3)
7.26 CDCl3
6.65
6.61
6.60
6.59
6.55
6.55
6.55
6.37
6.35
3.78
3.77
3.76
3.71
3.42
3.41
3.39
3.39
3.37
3.36
3.36
3.34
3.33
3.32
3.31
3.30
3.13
1.99
1.03
4.42
3.11
4.36
3.10
f1 (ppm)

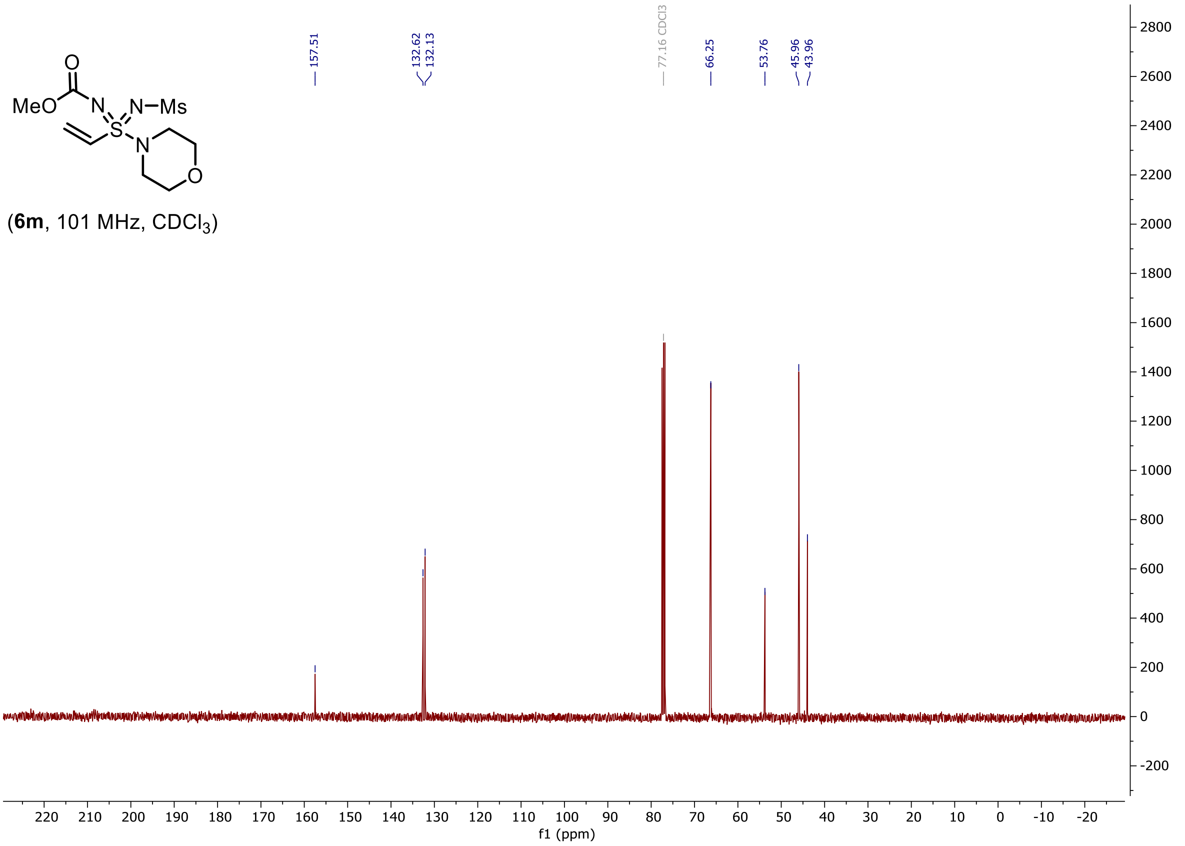

MeO
N
N−Ms
S
N
O
(6m, 101 MHz, CDCl3)
157.51
132.62
132.13
77.16 CDCl3
66.25
53.76
45.96
43.96
f1 (ppm)

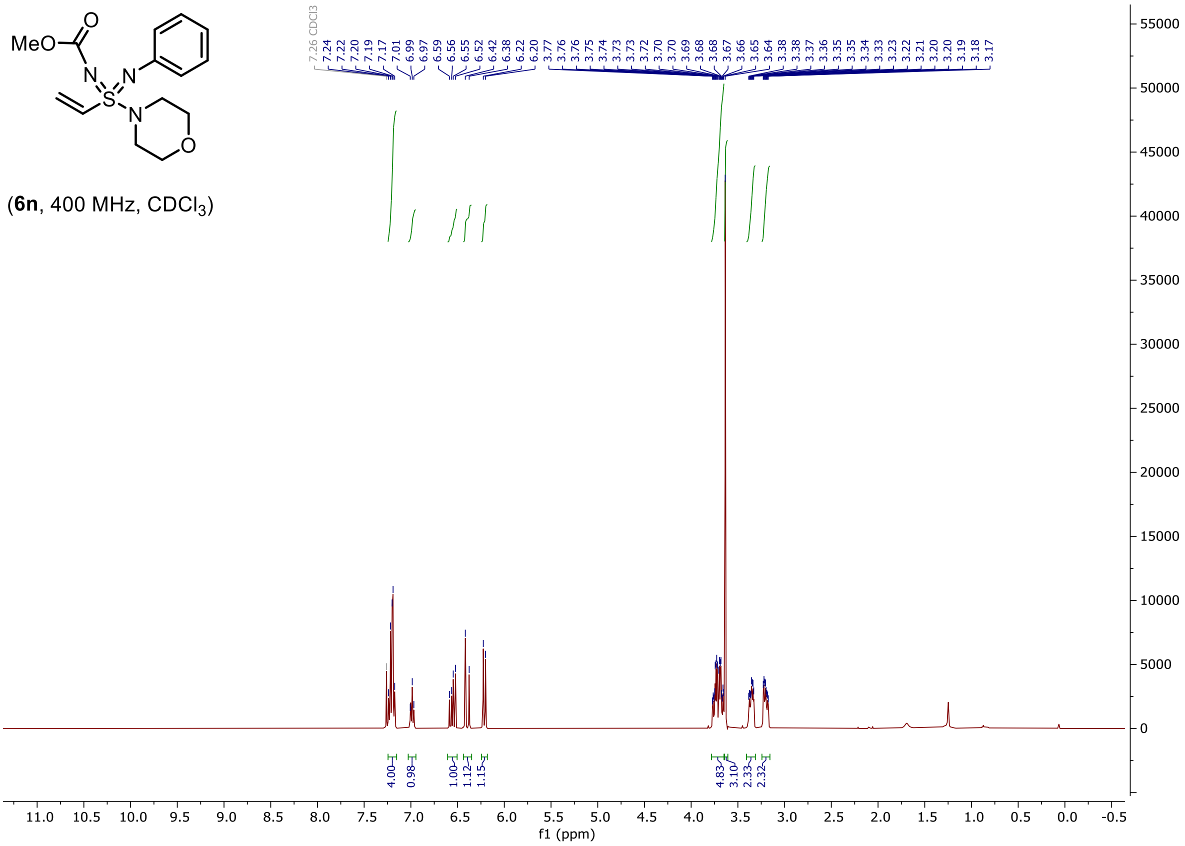

(6n, 400 MHz, CDCl3)
MeO
O
N
N
S
N
O
f1 (ppm)

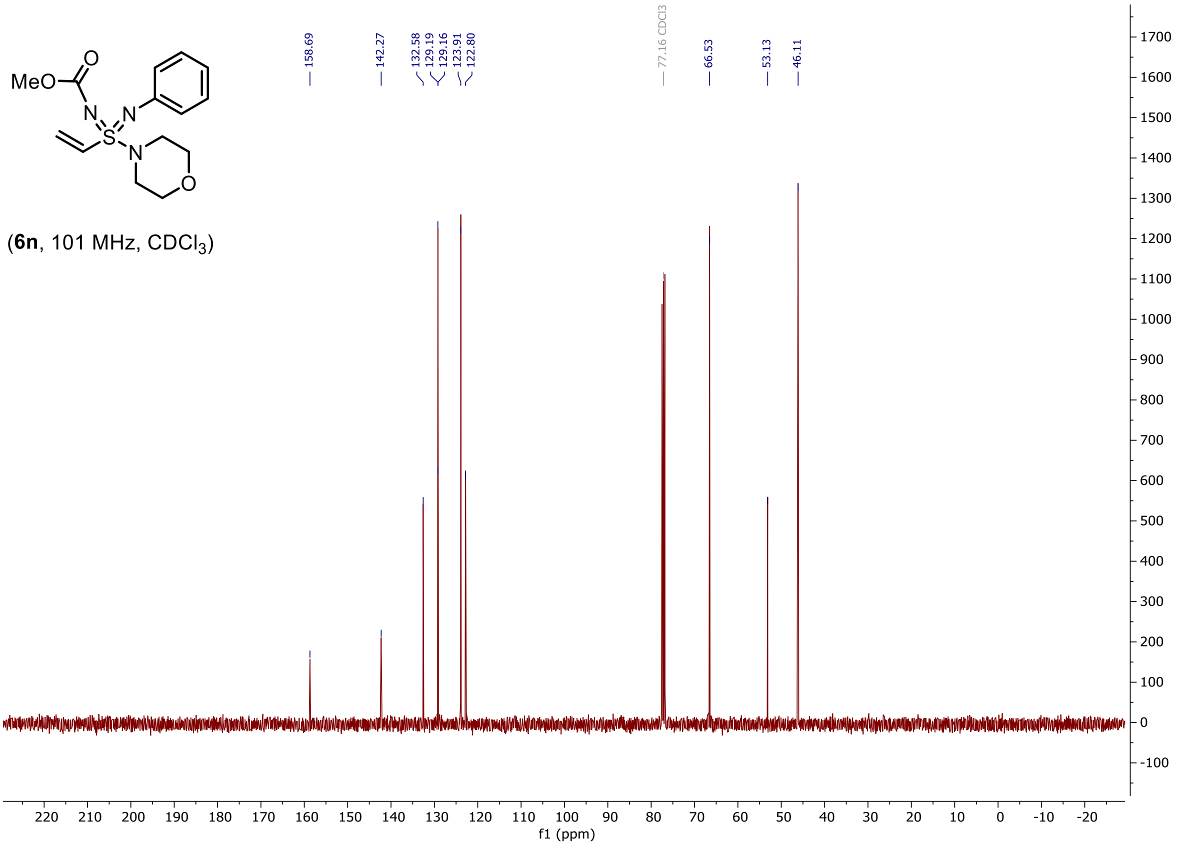

(6n, 101 MHz, CDCl3)
158.69
142.27
132.58
129.19
129.16
123.91
122.80
77.16 CDCl3
66.53
53.13
46.11
f1 (ppm)

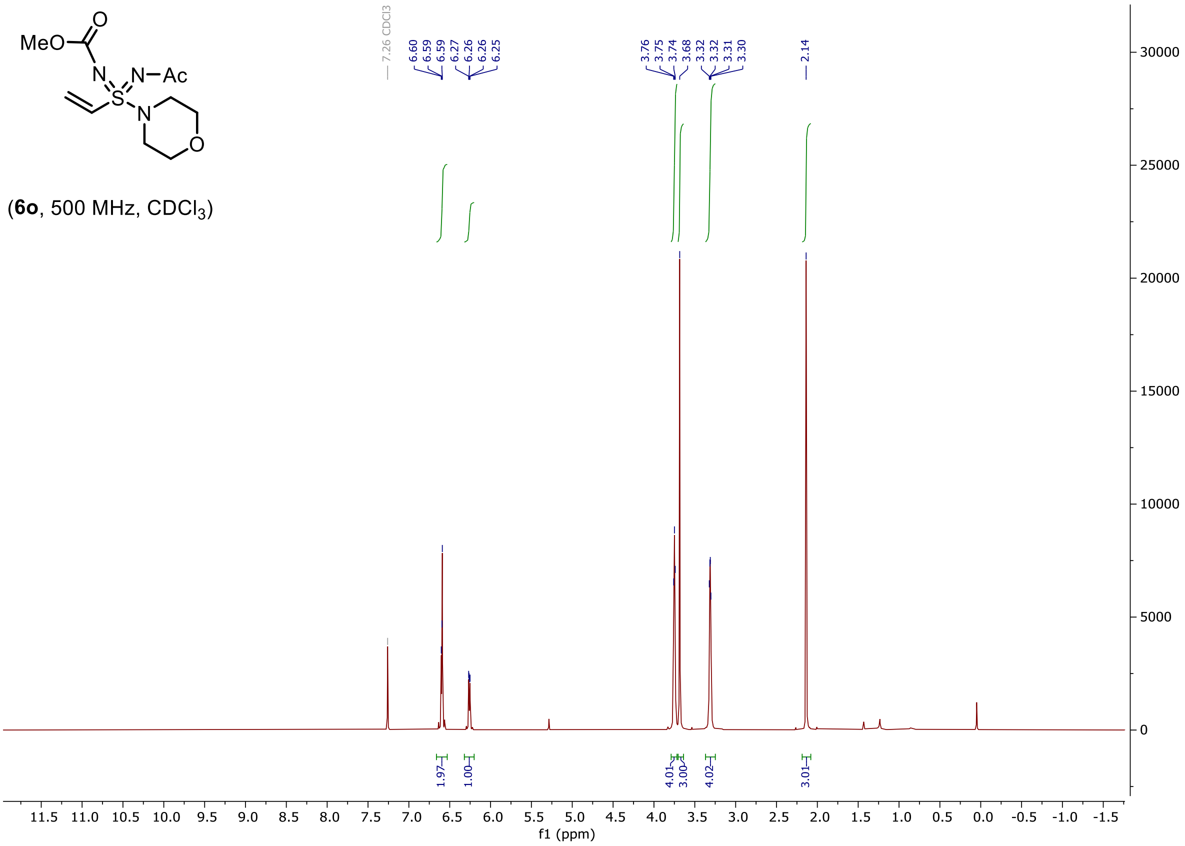

(**6o**, 500 MHz, $CDCl_3$)

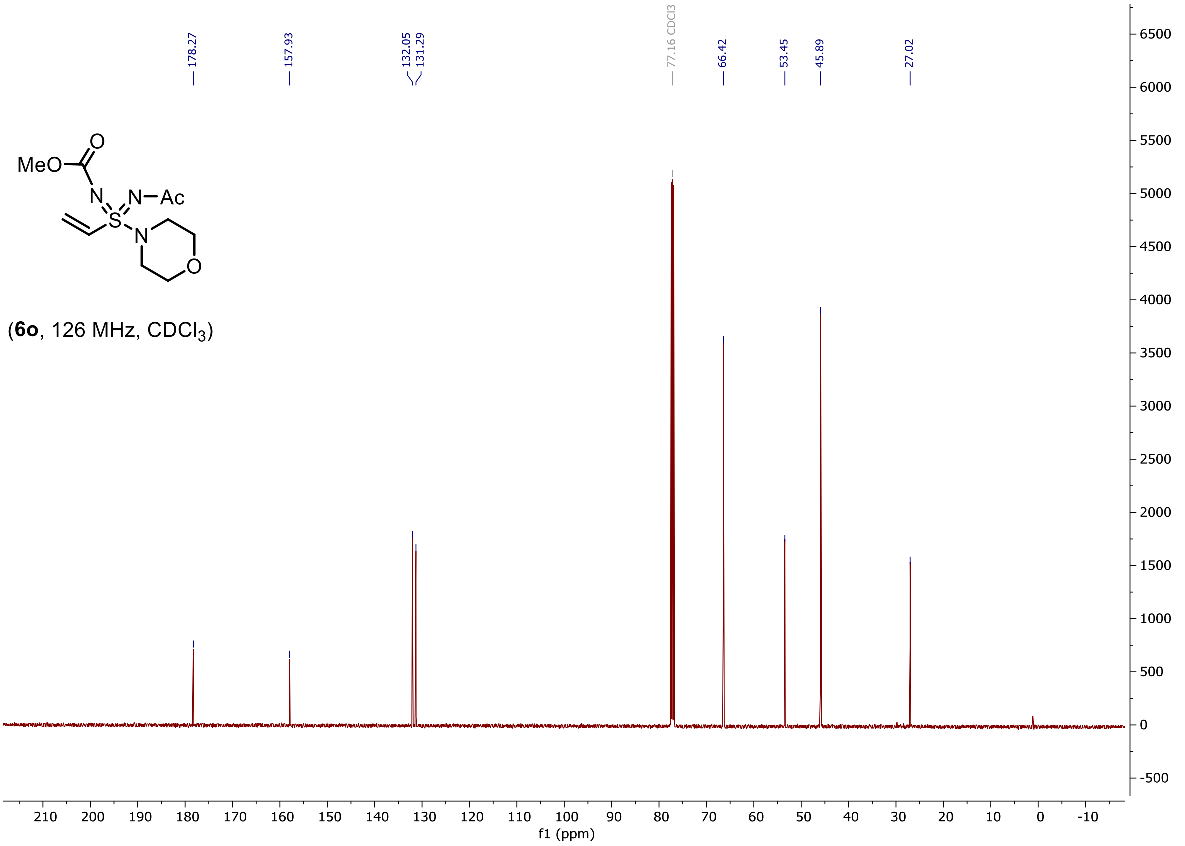

(**6o**, 126 MHz, $CDCl_3$)

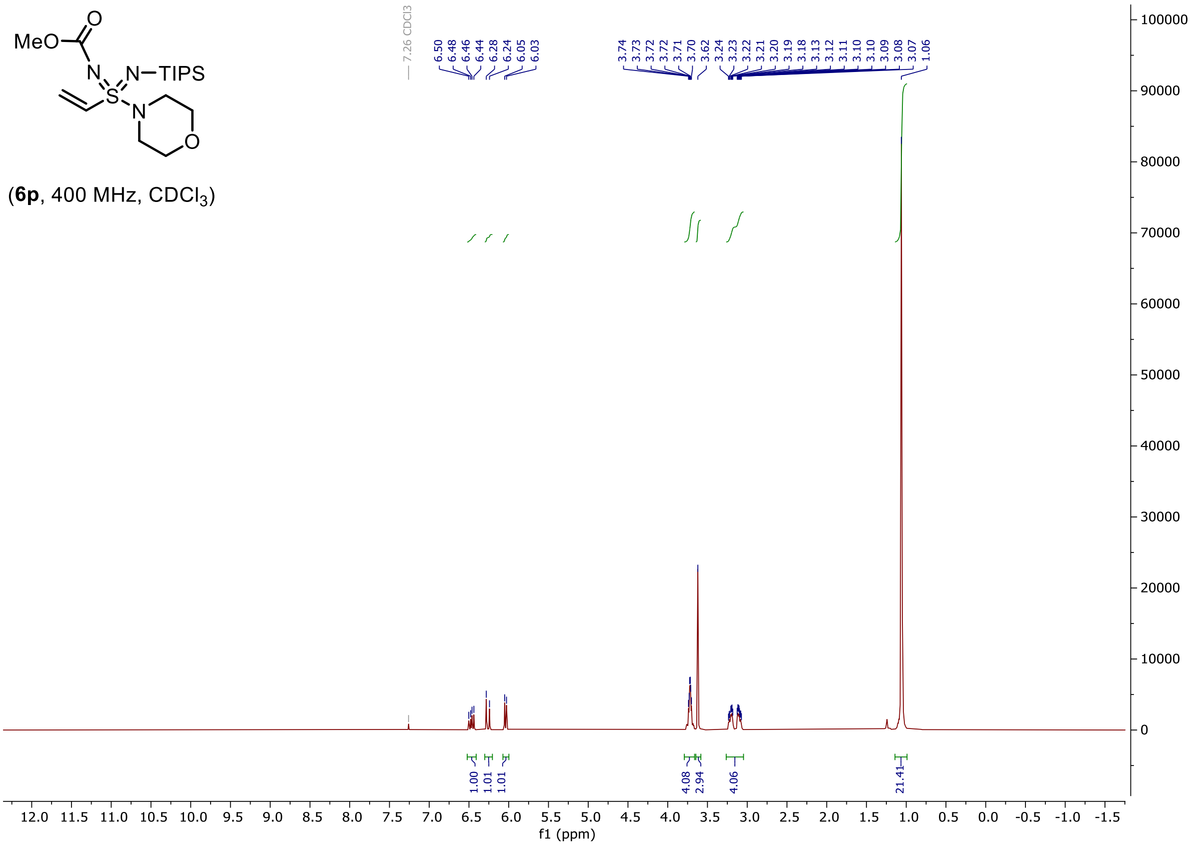

(**6p**, 400 MHz, $CDCl_3$)

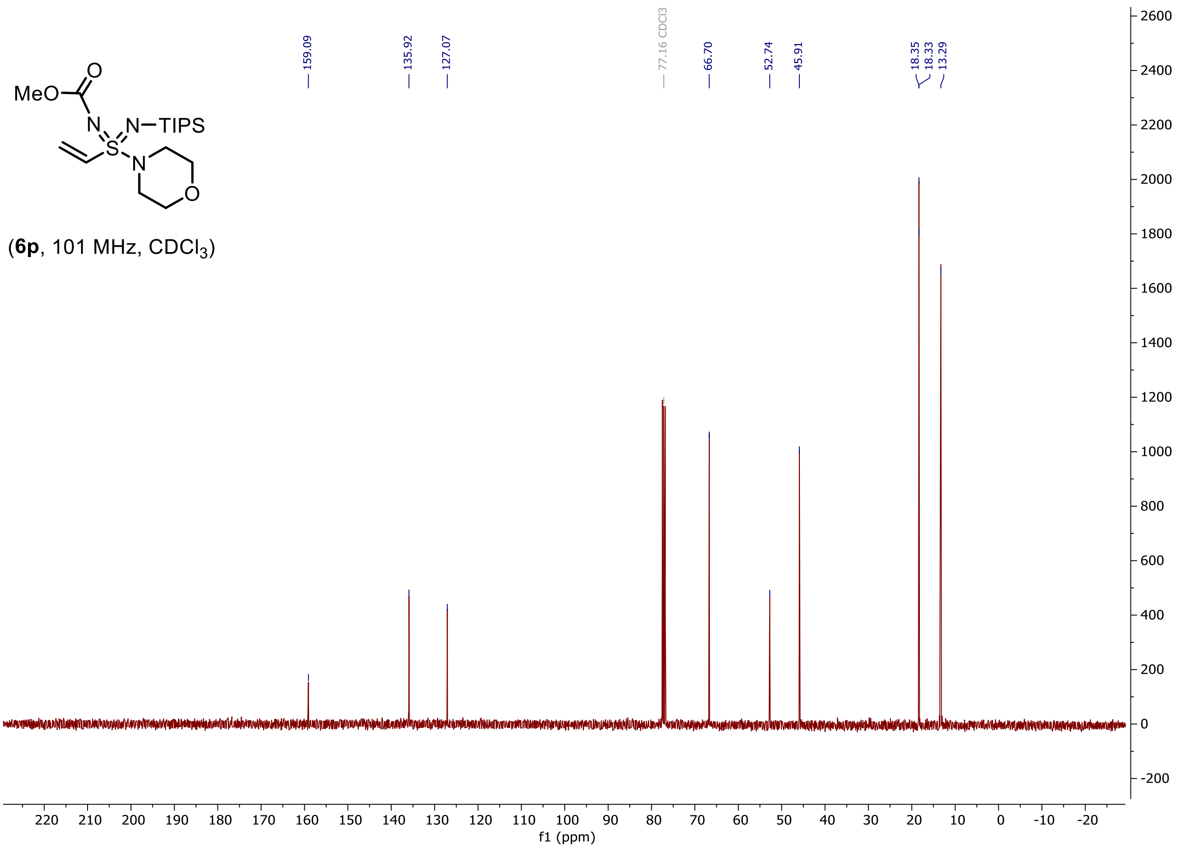

(**6p**, 101 MHz, $CDCl_3$)

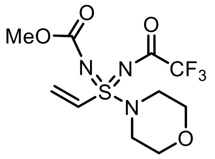

(**6q**, 500 MHz, $CDCl_3$)

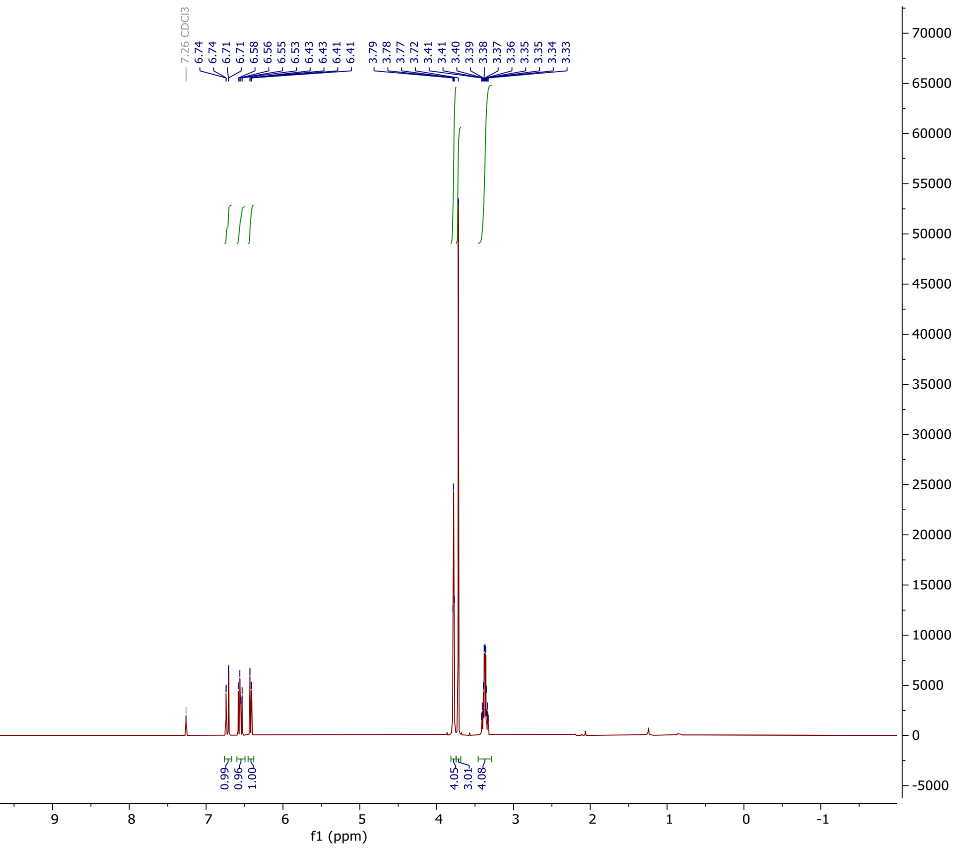

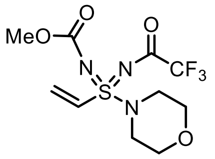

(**6q**, 126 MHz, $CDCl_3$)

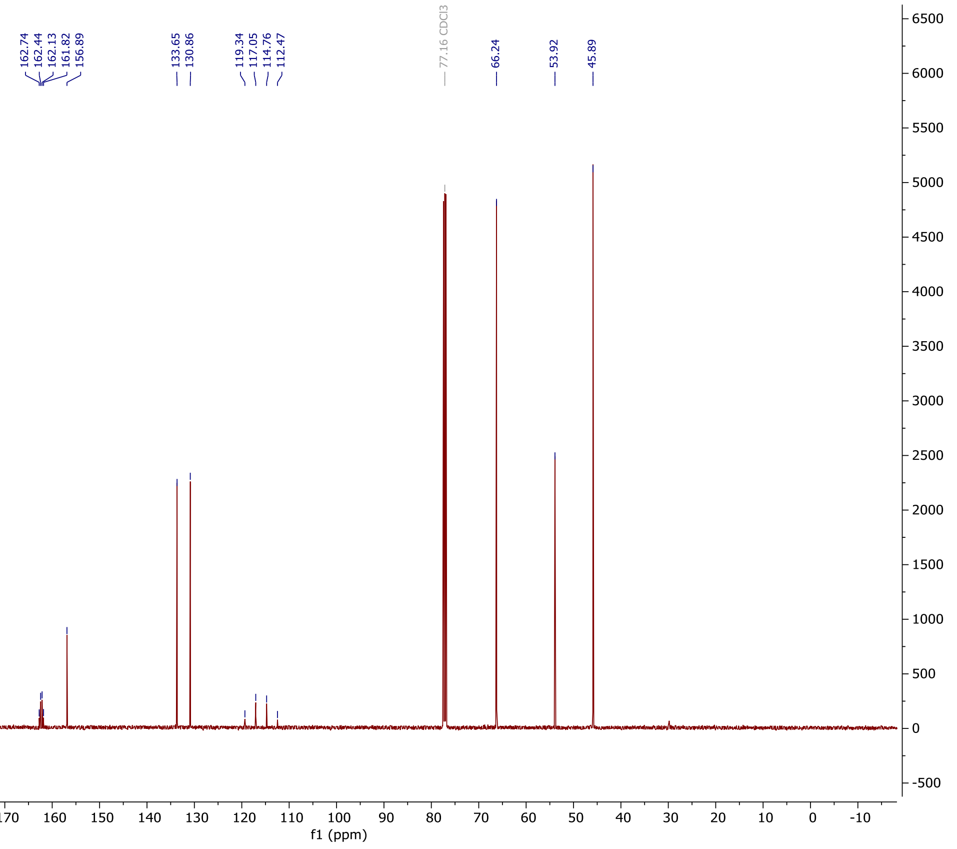

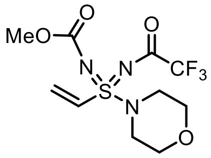

(**6q**, 377 MHz, $CDCl_3$)

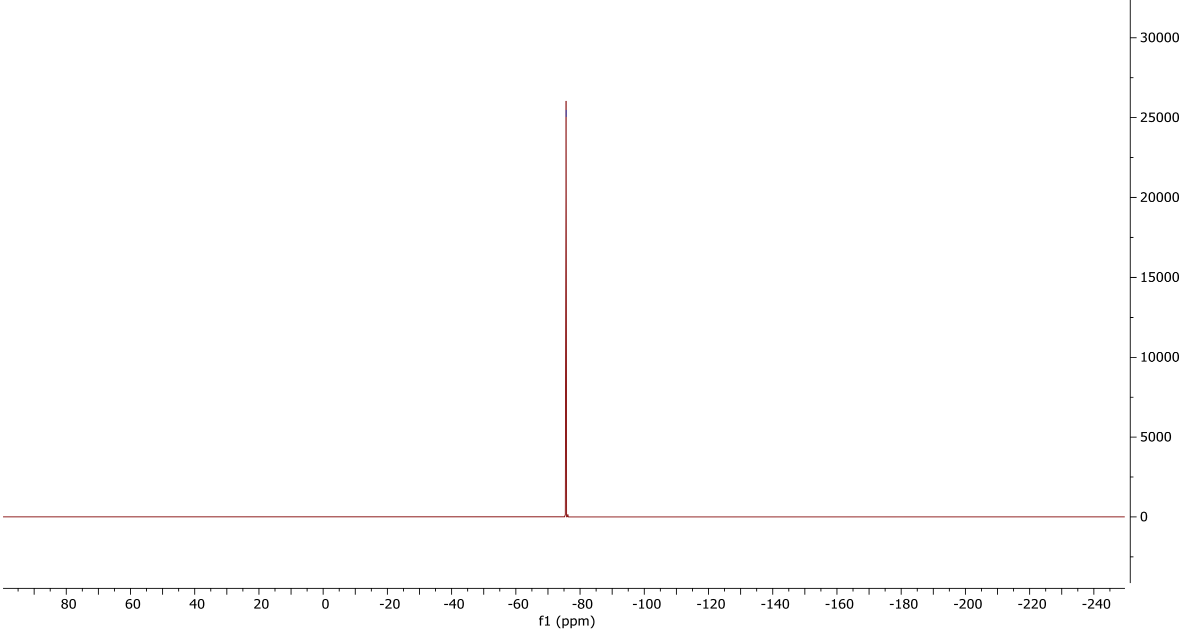

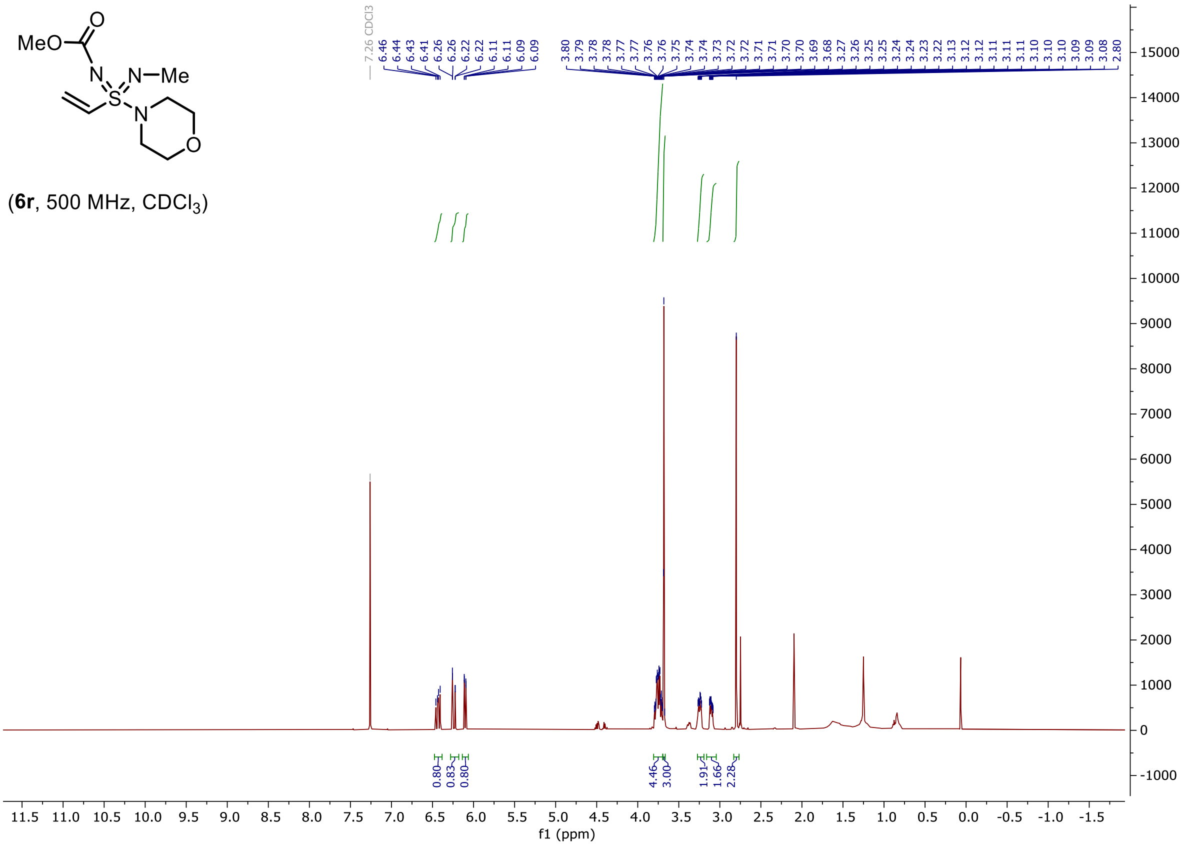

(**6r**, 500 MHz, $CDCl_3$)

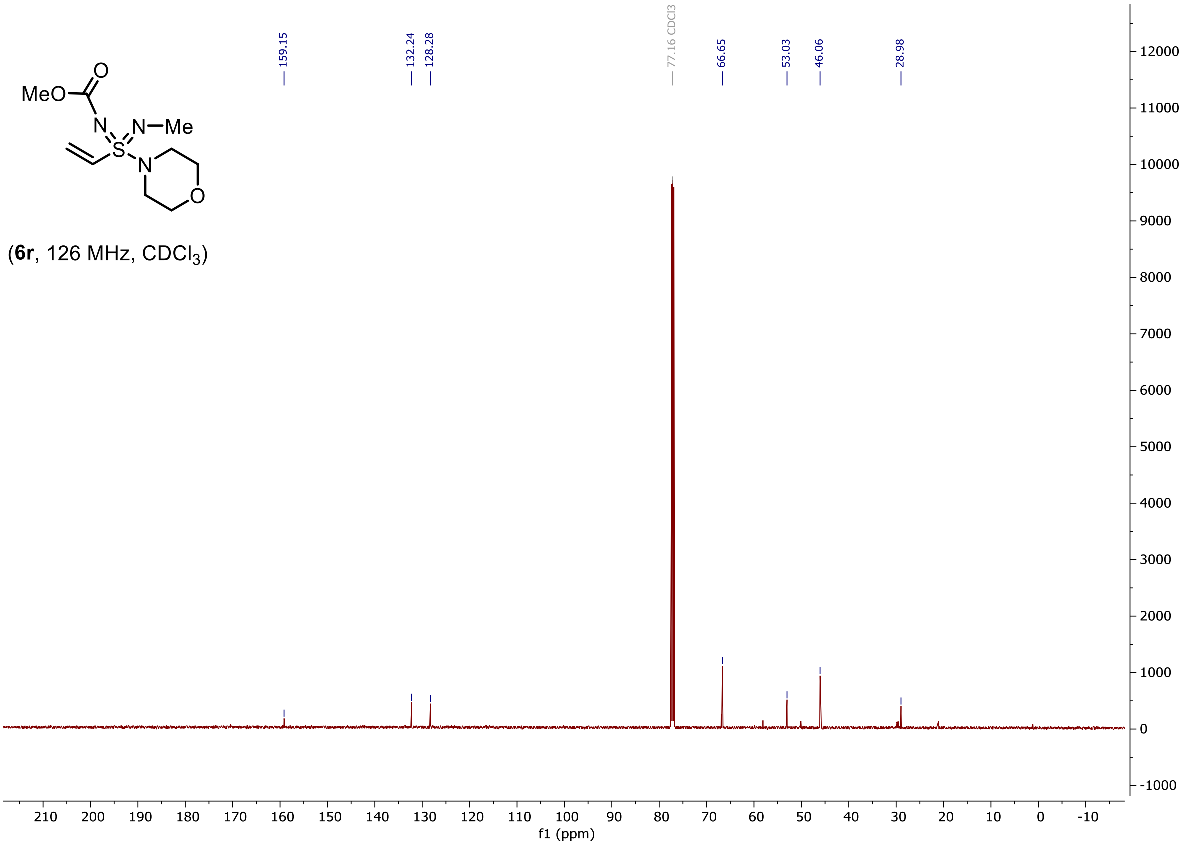

(**6r**, 126 MHz, $CDCl_3$)

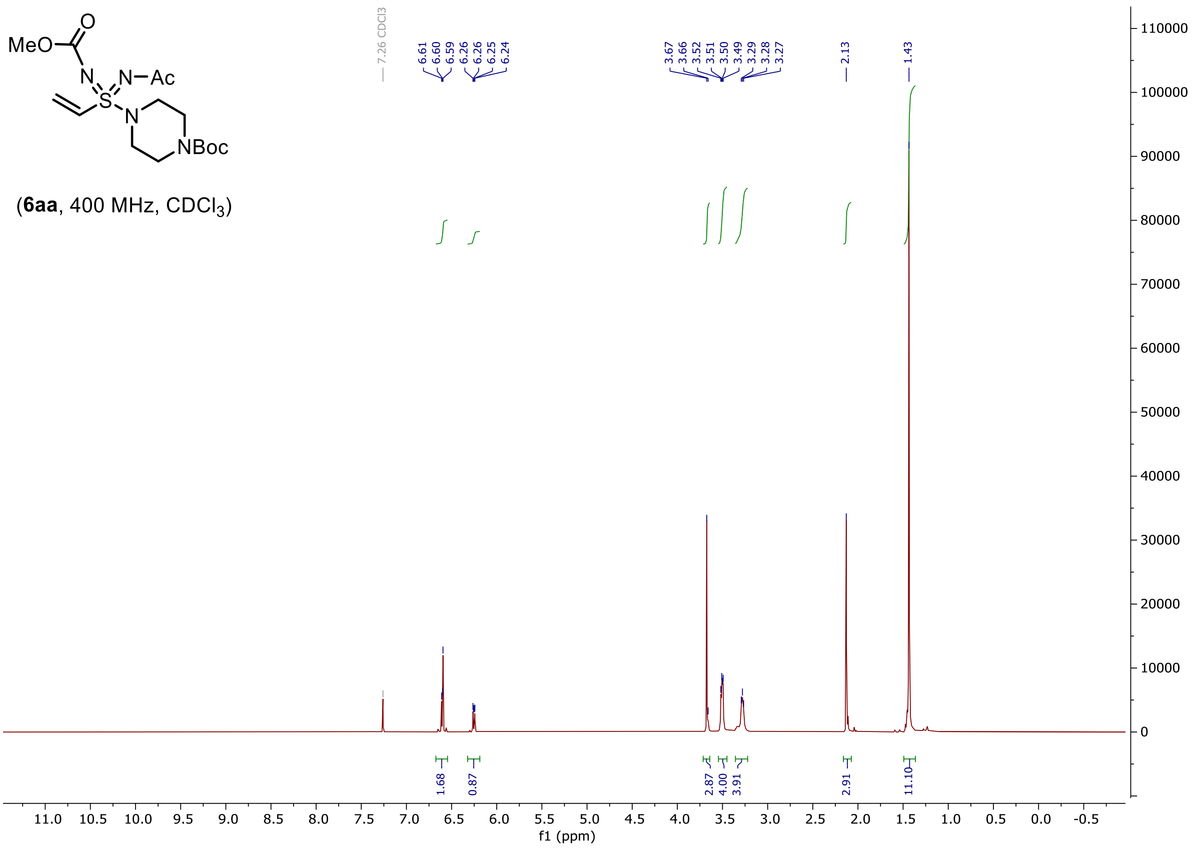

MeO
O
N
N−Ac
S
N
NBoc
(6aa, 400 MHz, CDCl3)
7.26 CDCl3
6.61
6.60
6.59
6.26
6.26
6.25
6.24
3.67
3.66
3.52
3.51
3.50
3.49
3.29
3.28
3.27
2.13
1.43
1.68
0.87
2.87
4.00
3.91
2.91
11.10
f1 (ppm)

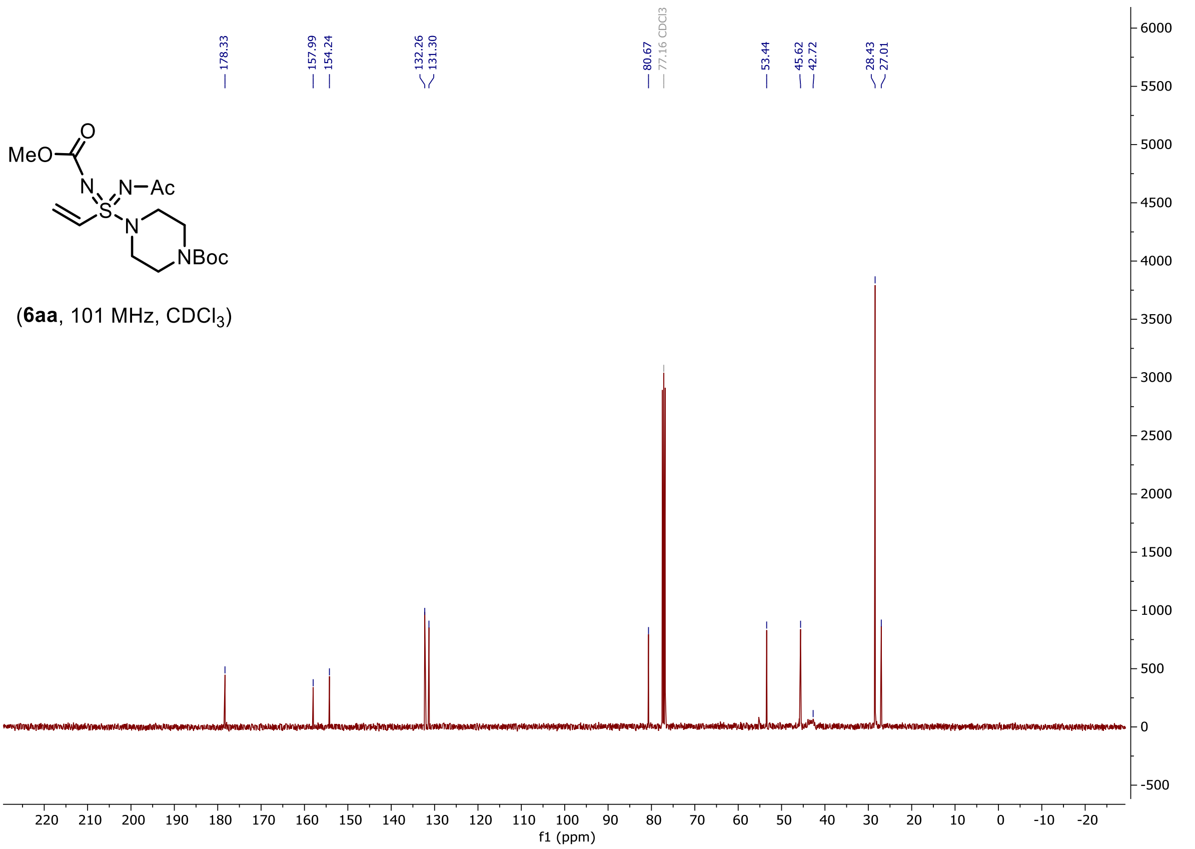

MeO
O
N
N−Ac
S
N
NBoc
(6aa, 101 MHz, CDCl3)
178.33
157.99
154.24
132.26
131.30
80.67
77.16 CDCl3
53.44
45.62
42.72
28.43
27.01
f1 (ppm)

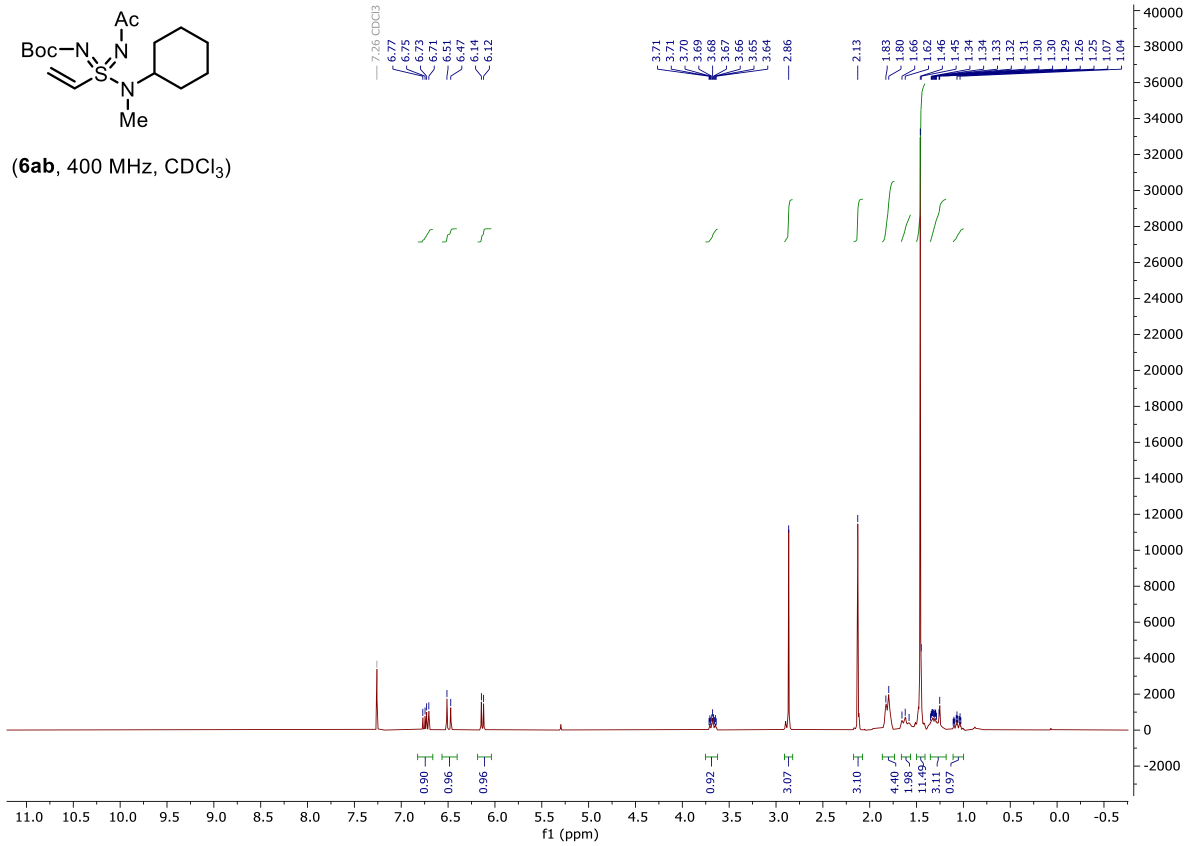

(**6ab**, 400 MHz, $CDCl_3$)

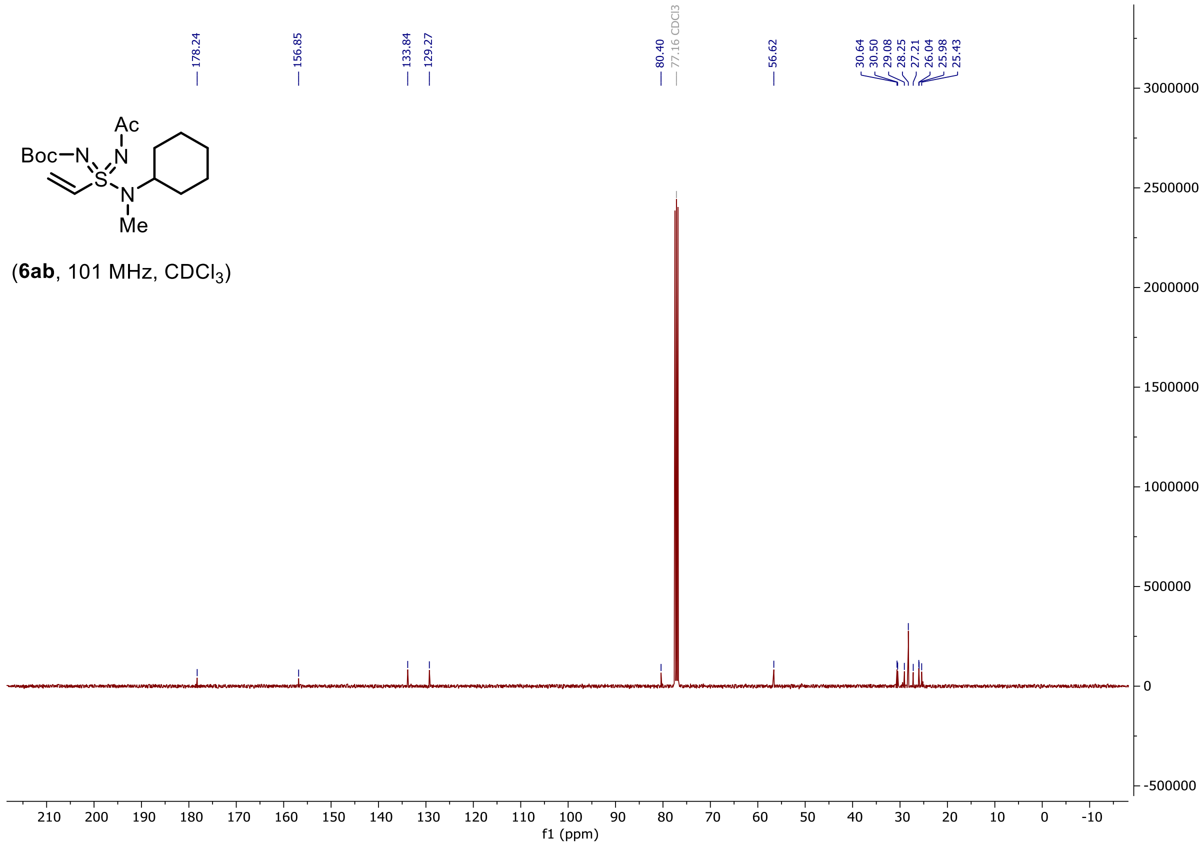

(**6ab**, 101 MHz, $CDCl_3$)

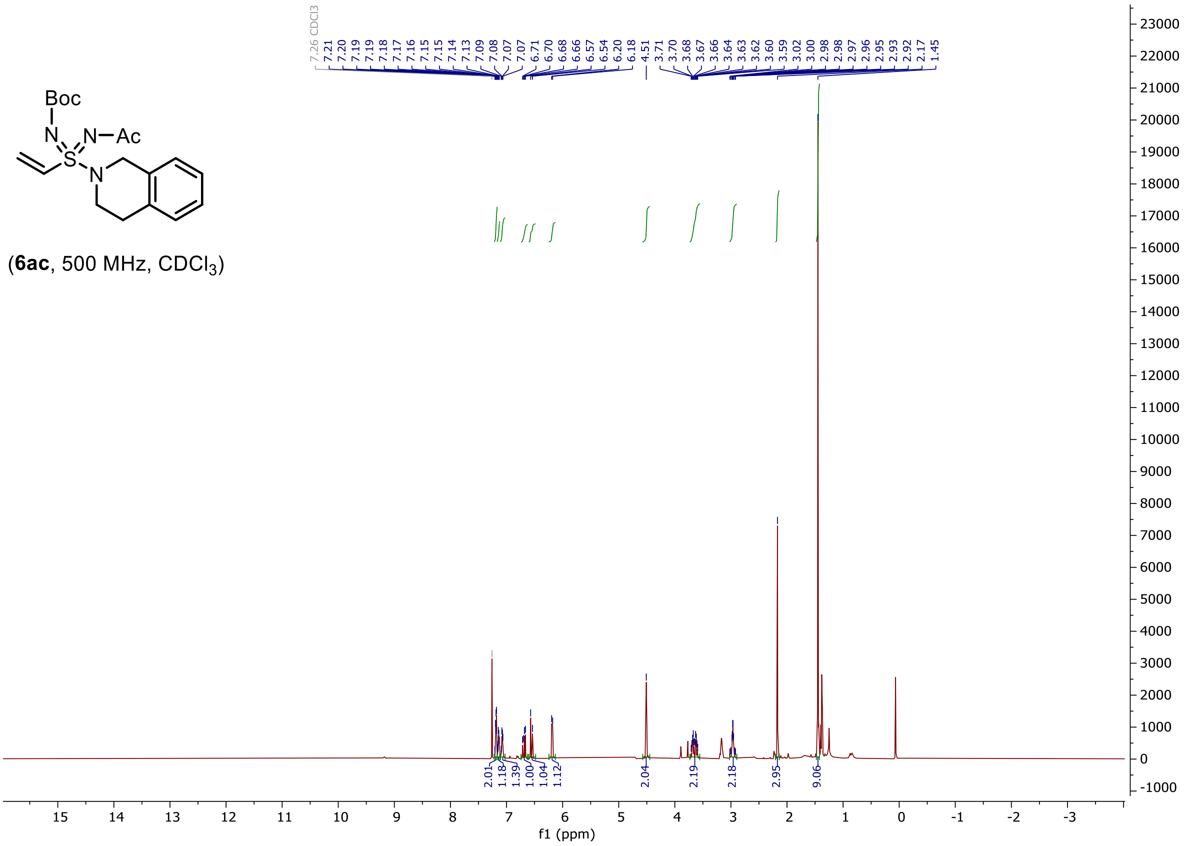

(**6ac**, 500 MHz, $CDCl_3$)

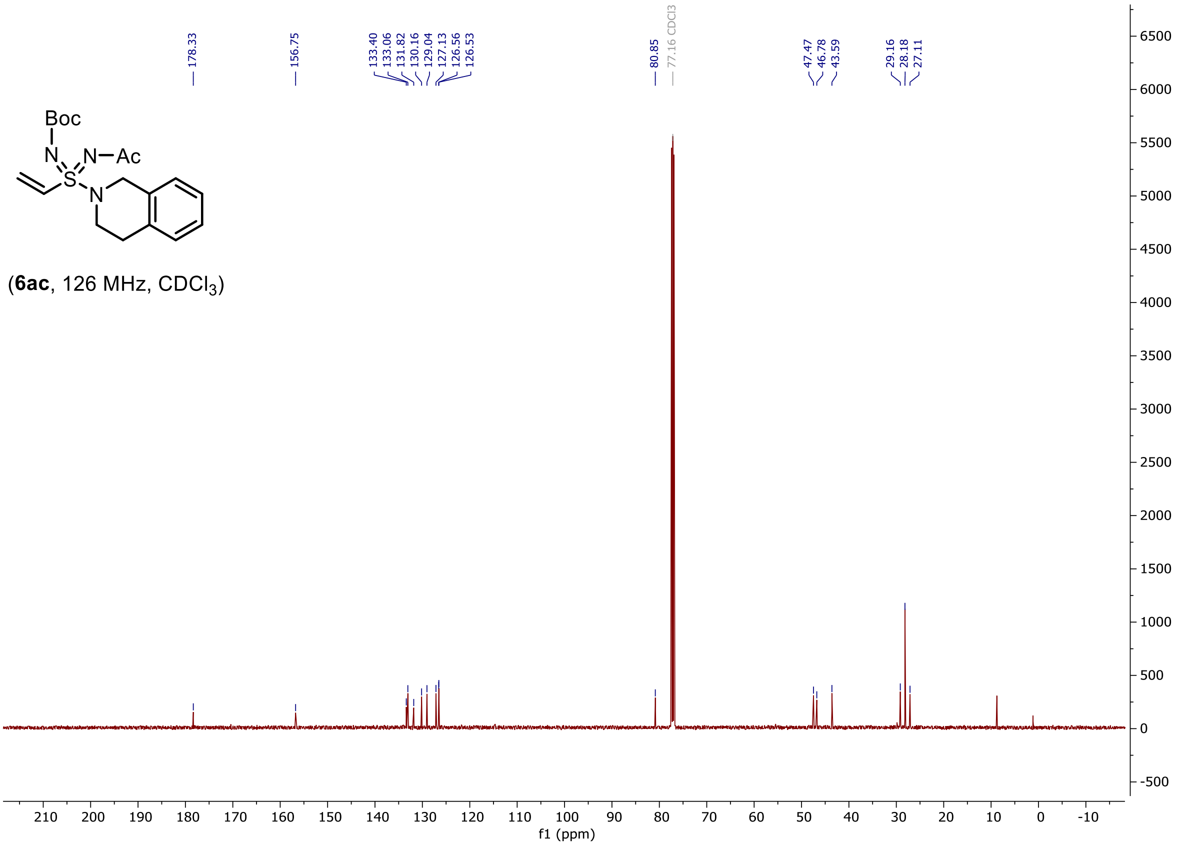

(**6ac**, 126 MHz, $CDCl_3$)

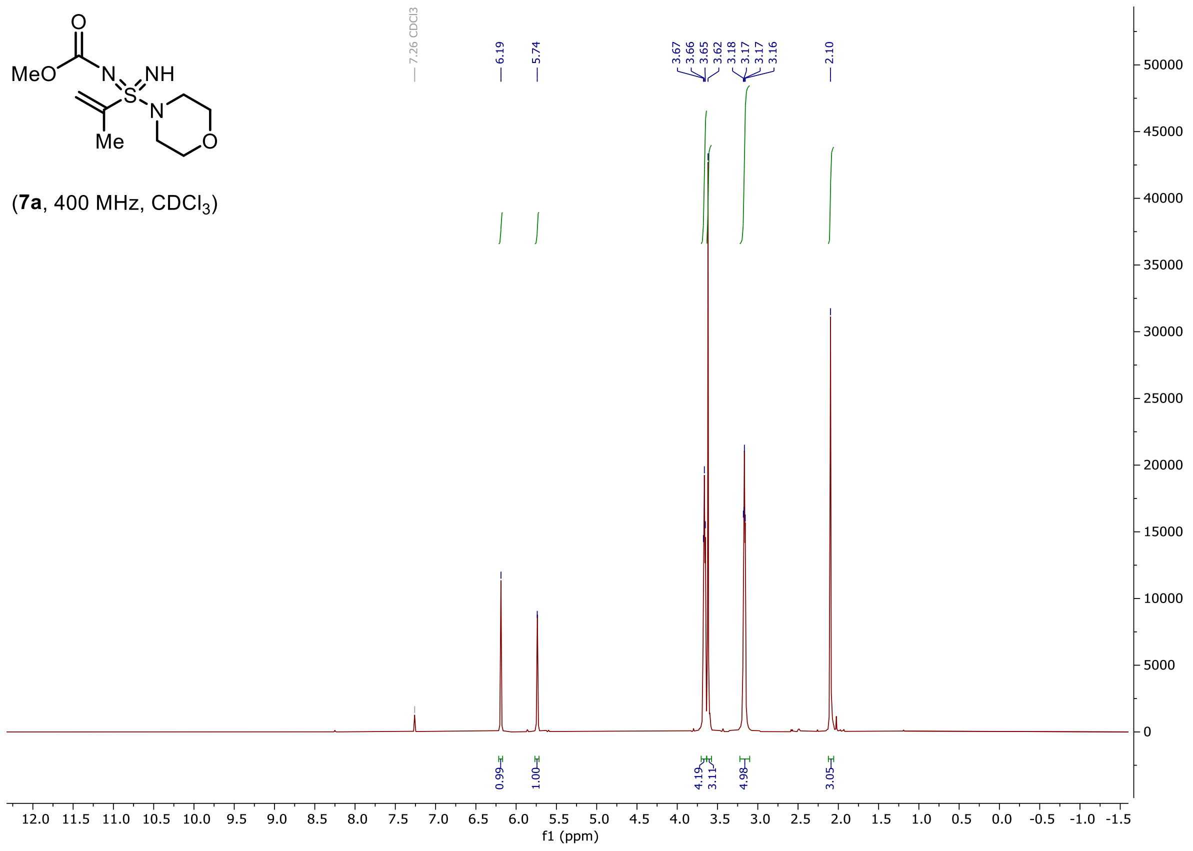

(**7a**, 400 MHz, $CDCl_3$)

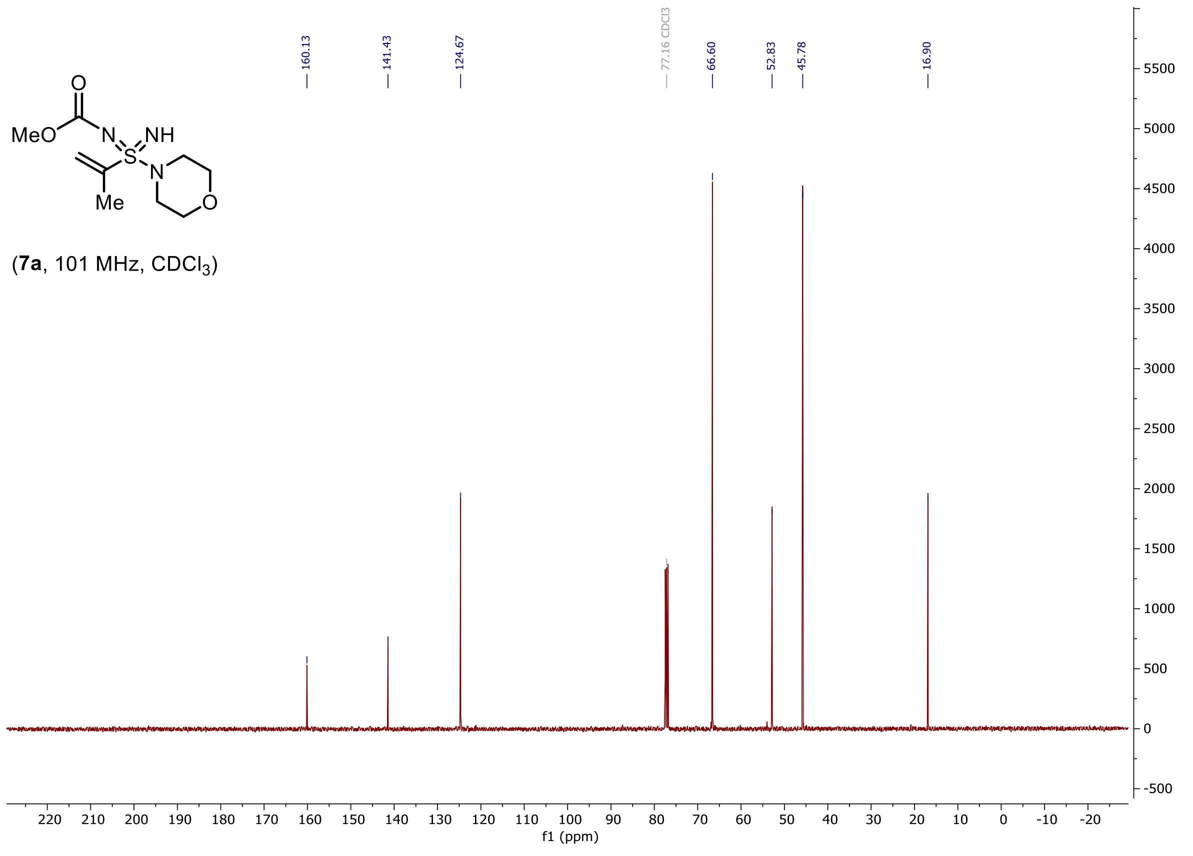

(**7a**, 101 MHz, $CDCl_3$)

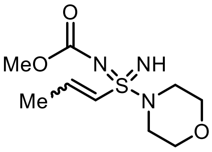

(**7b**, 400 MHz, $CDCl_3$)

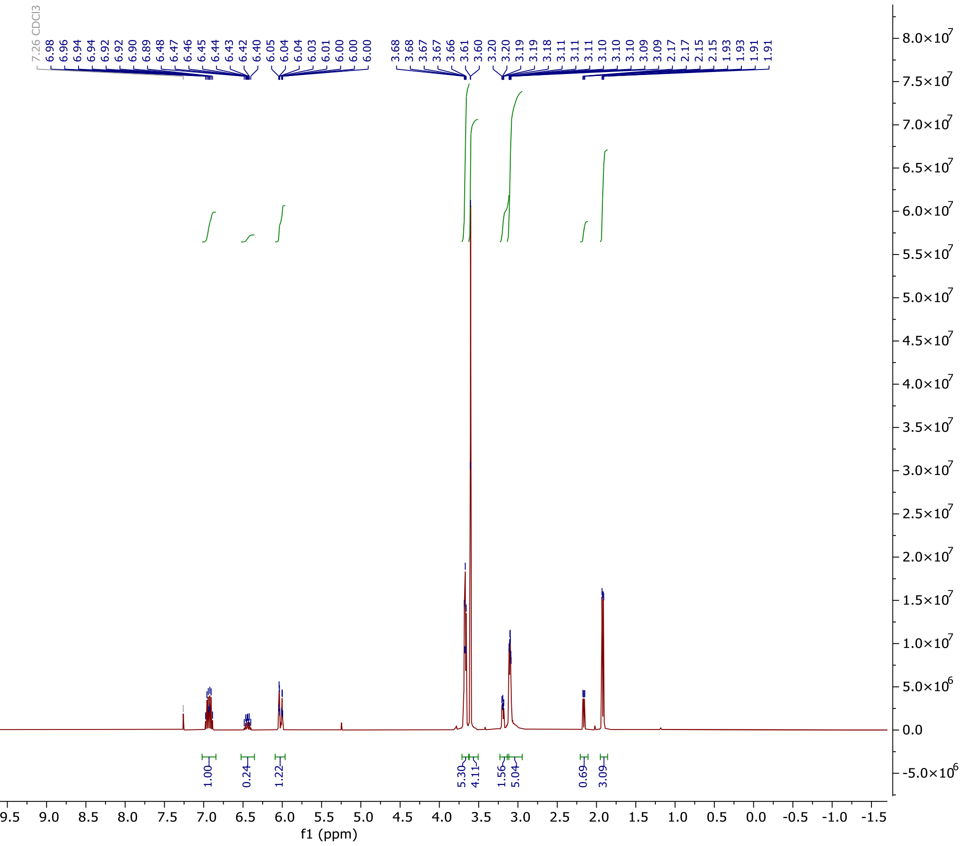

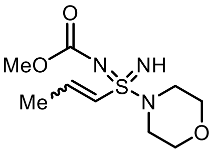

(**7b**, 101 MHz, $CDCl_3$)

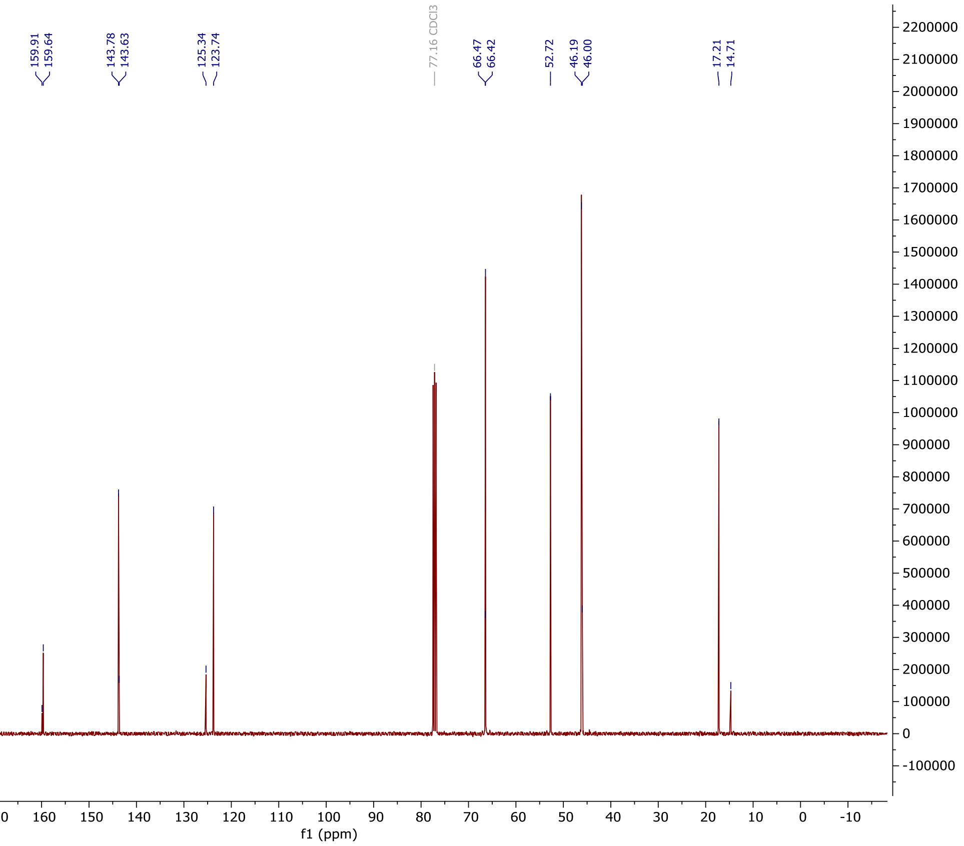

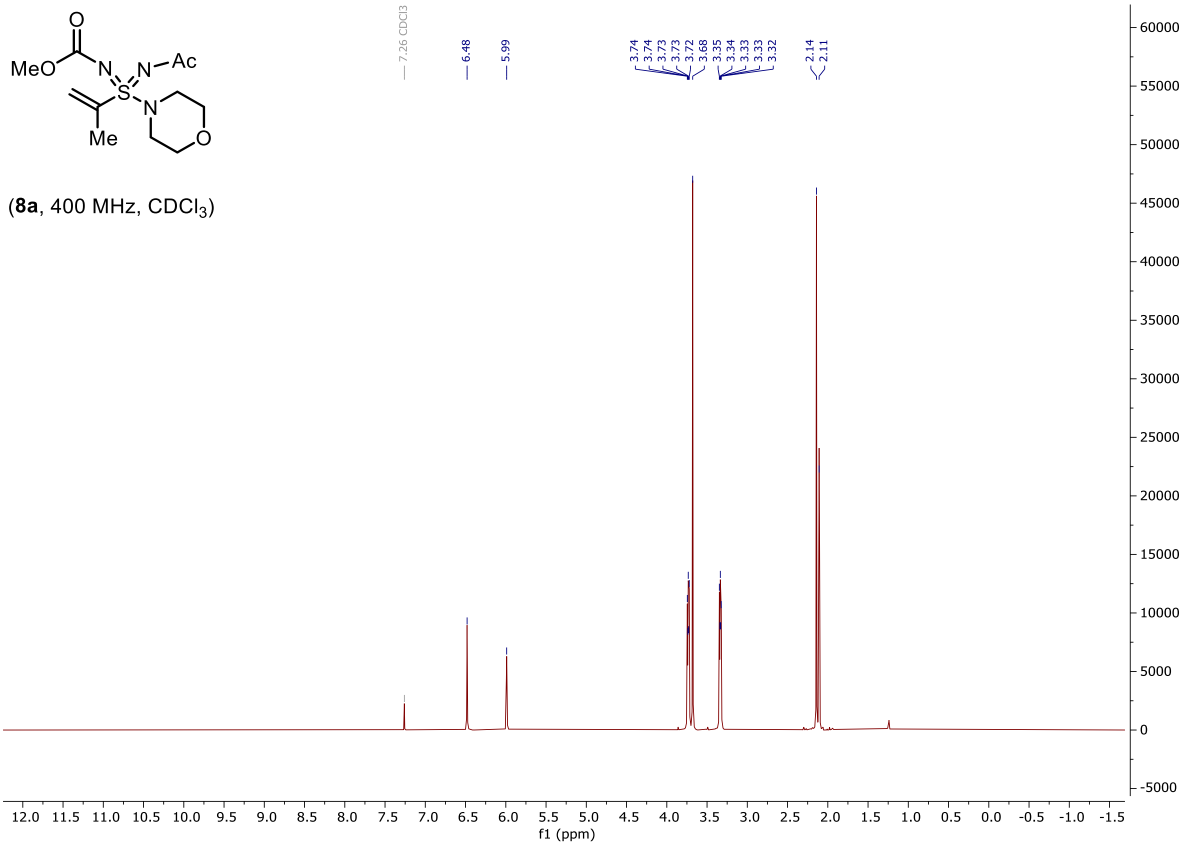
MeO
O
N
N
Ac
S
N
O
Me
(8a, 400 MHz, CDCl3)
7.26 CDCl3
6.48
5.99
3.74
3.74
3.73
3.73
3.72
3.68
3.35
3.34
3.33
3.33
3.32
2.14
2.11
f1 (ppm)

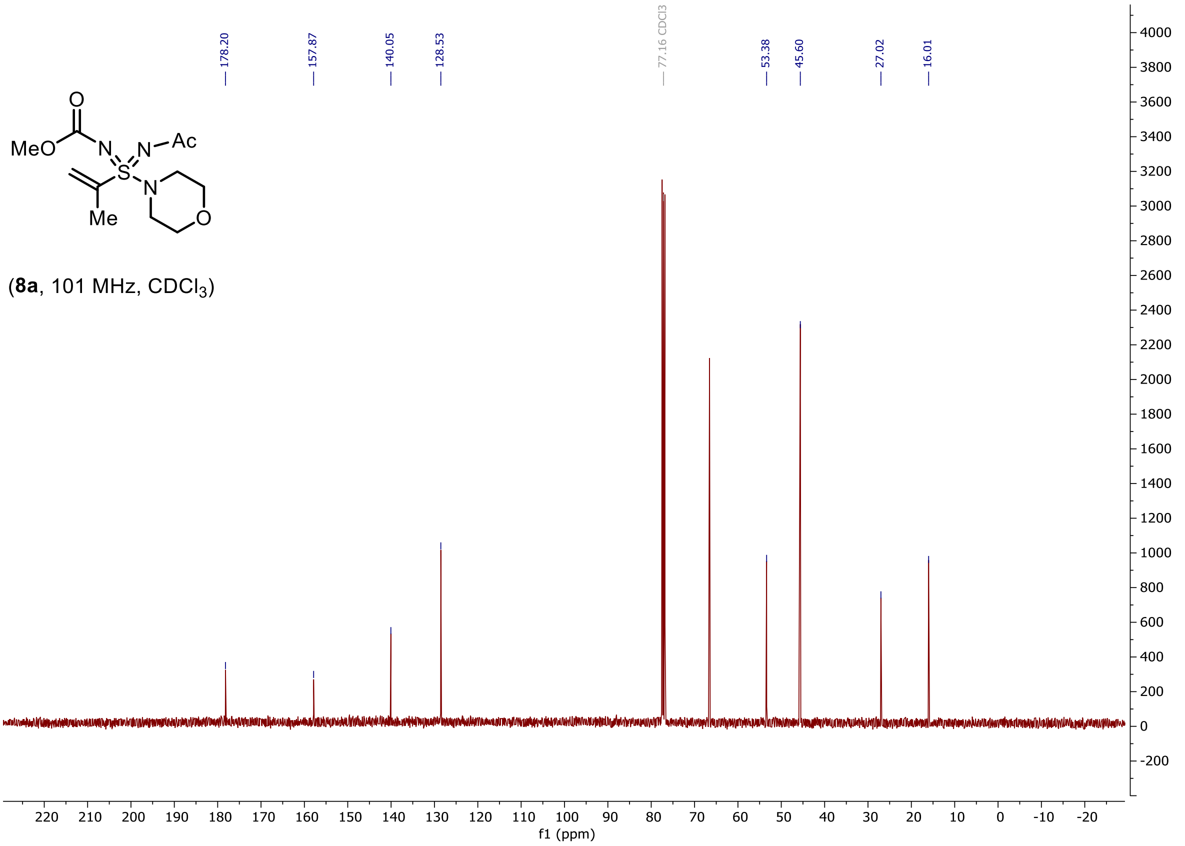
MeO
O
N
N
Ac
S
N
O
Me
(8a, 101 MHz, CDCl3)
178.20
157.87
140.05
128.53
77.16 CDCl3
53.38
45.60
27.02
16.01
f1 (ppm)

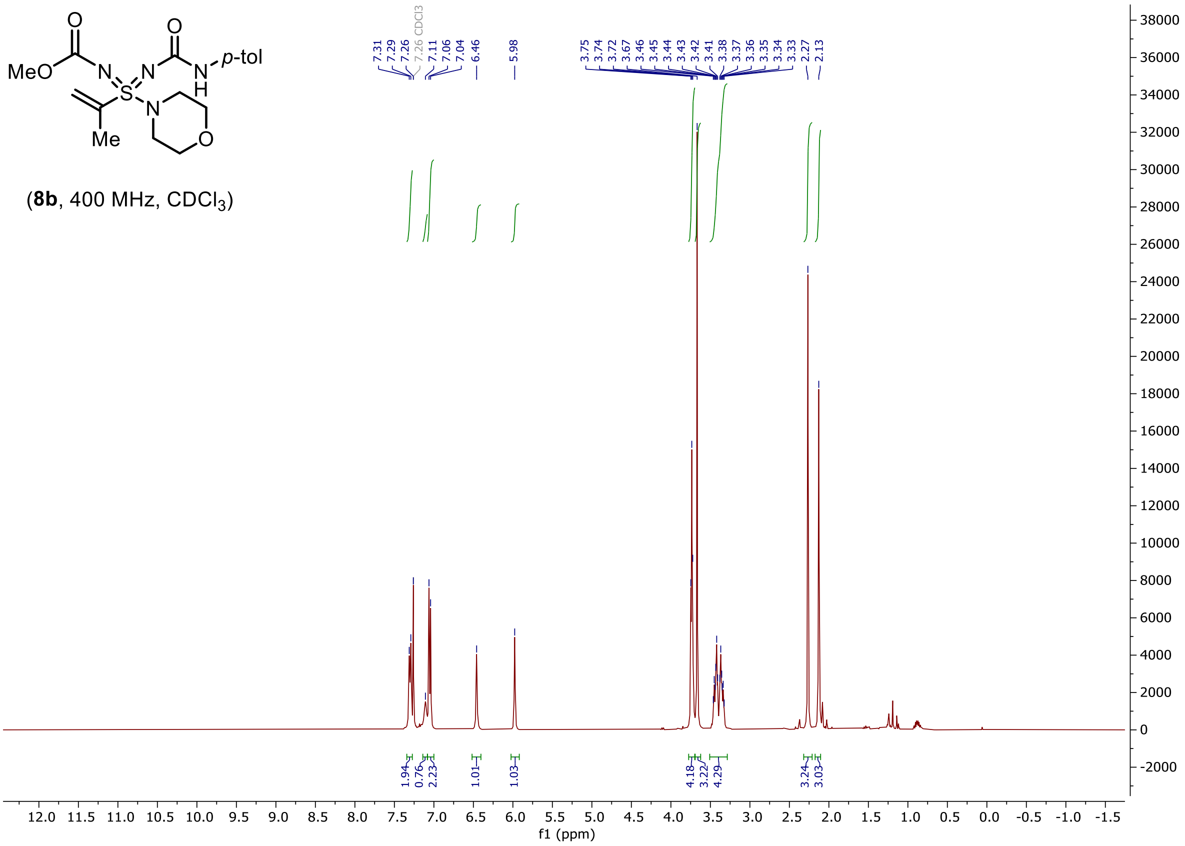
MeO
N
N
N
p-tol
H
S
N
O
Me
(8b, 400 MHz, CDCl3)
7.31
7.29
7.26
7.26 CDCl3
7.11
7.06
7.04
6.46
5.98
3.75
3.74
3.72
3.67
3.46
3.45
3.44
3.43
3.42
3.41
3.38
3.37
3.36
3.35
3.34
3.33
2.27
2.13
1.94
0.76
2.23
1.01
1.03
4.18
3.22
4.29
3.24
3.03
f1 (ppm)

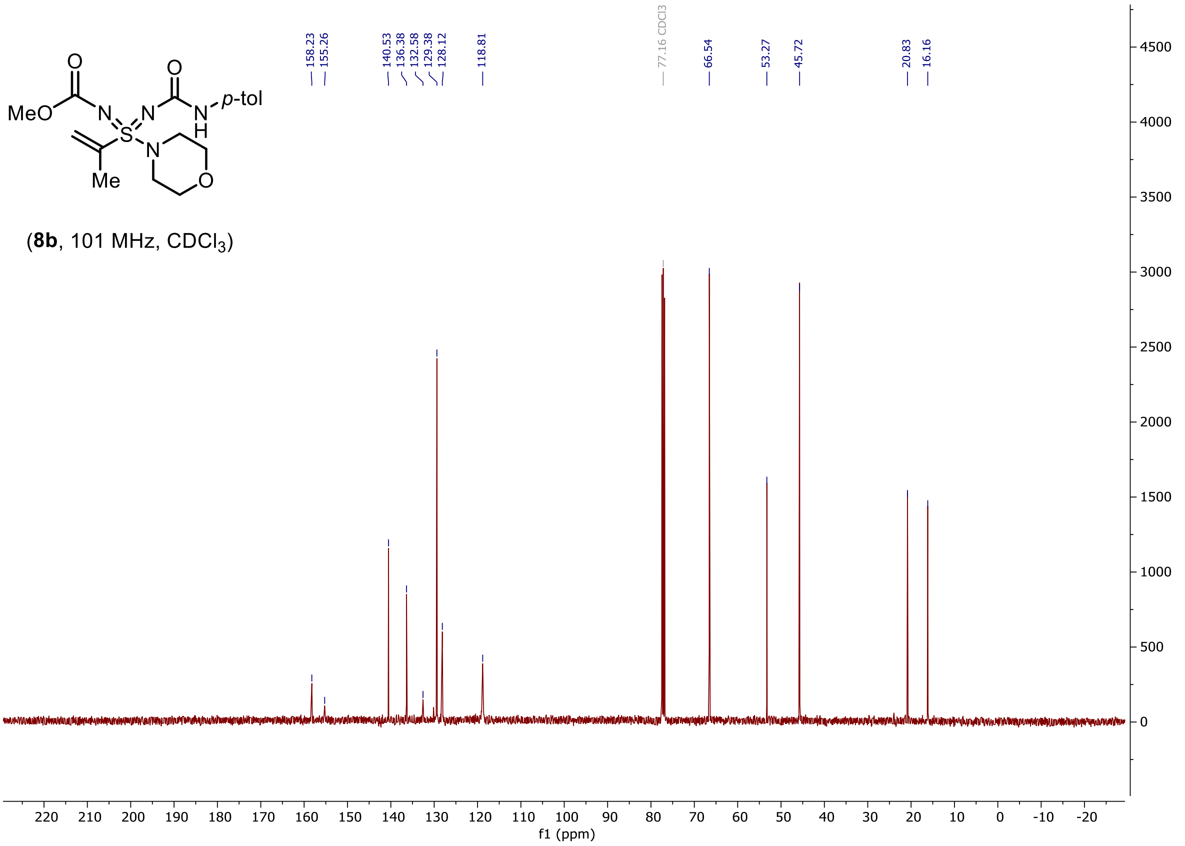
MeO
N
N
N
p-tol
H
S
N
O
Me
(8b, 101 MHz, CDCl3)
158.23
155.26
140.53
136.38
132.58
129.38
128.12
118.81
77.16 CDCl3
66.54
53.27
45.72
20.83
16.16
f1 (ppm)

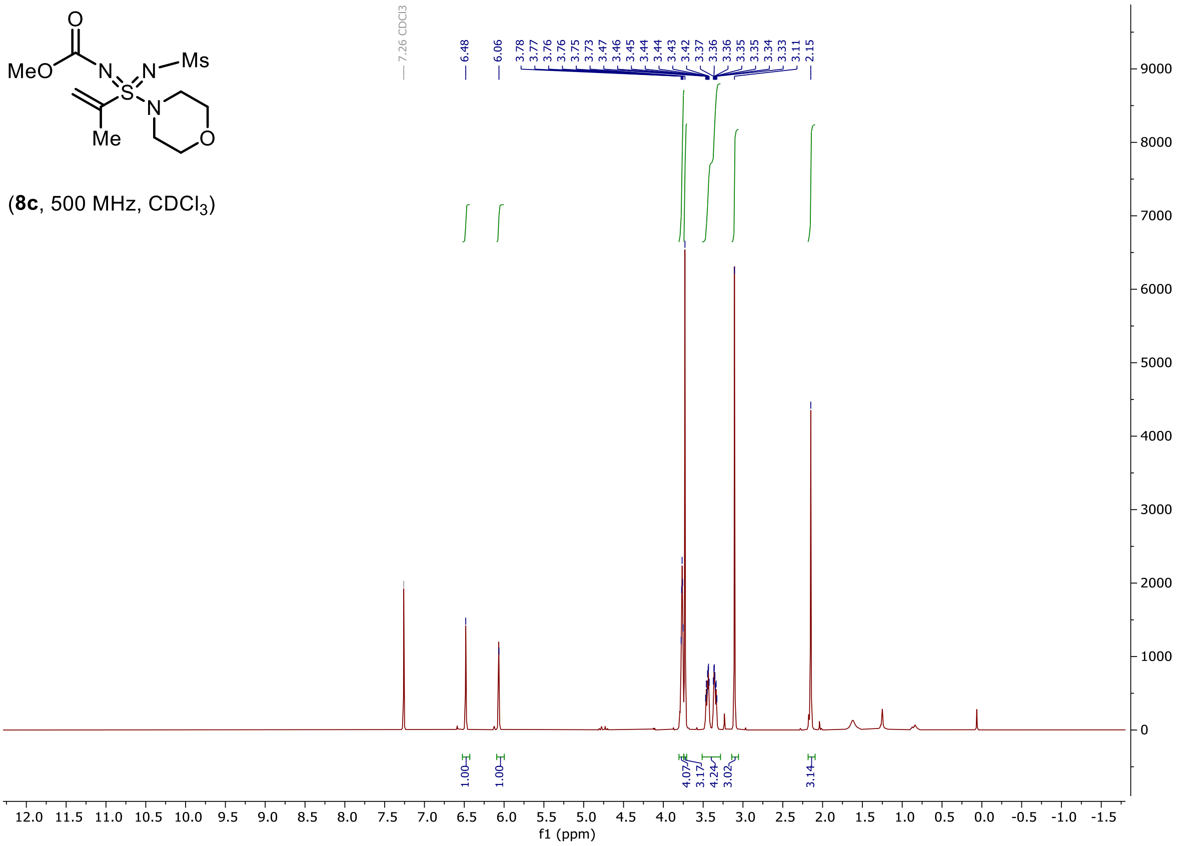

(**8c**, 500 MHz, $CDCl_3$)

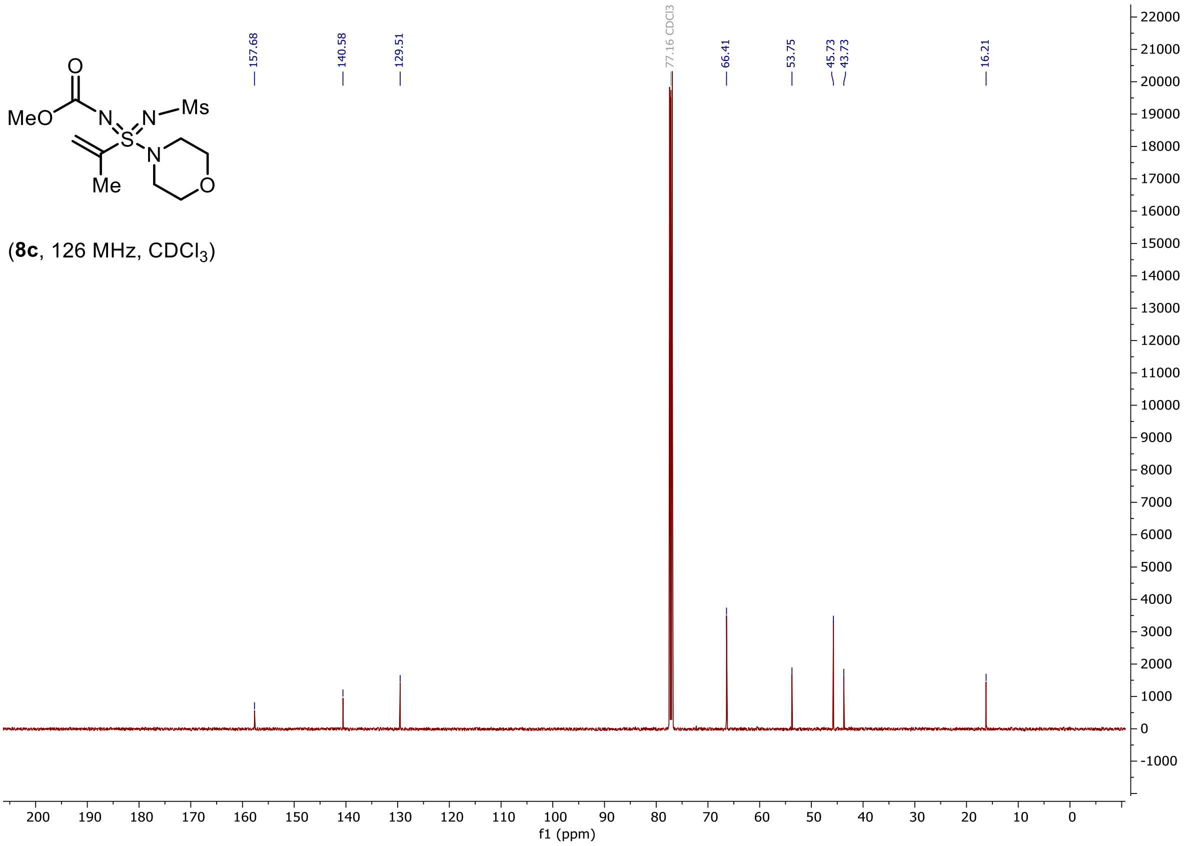

(**8c**, 126 MHz, $CDCl_3$)

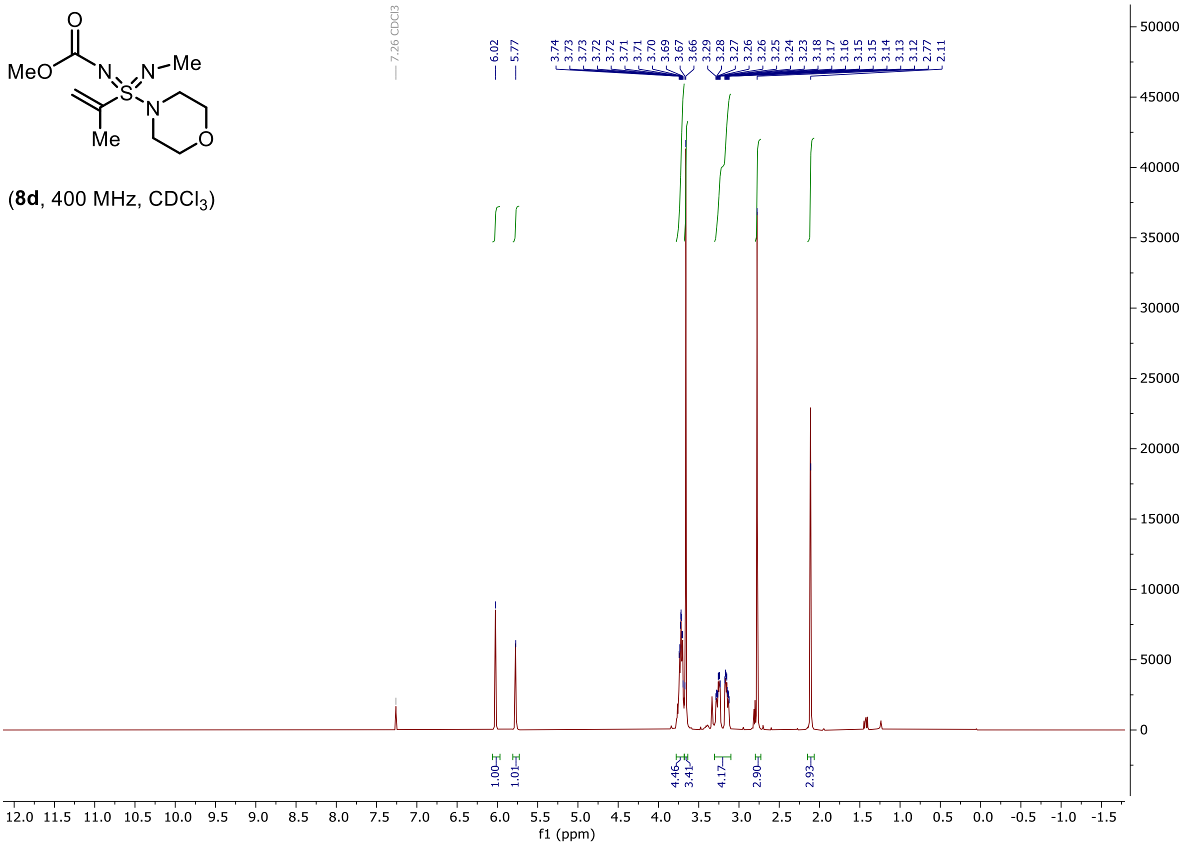

(**8d**, 400 MHz, $CDCl_3$)

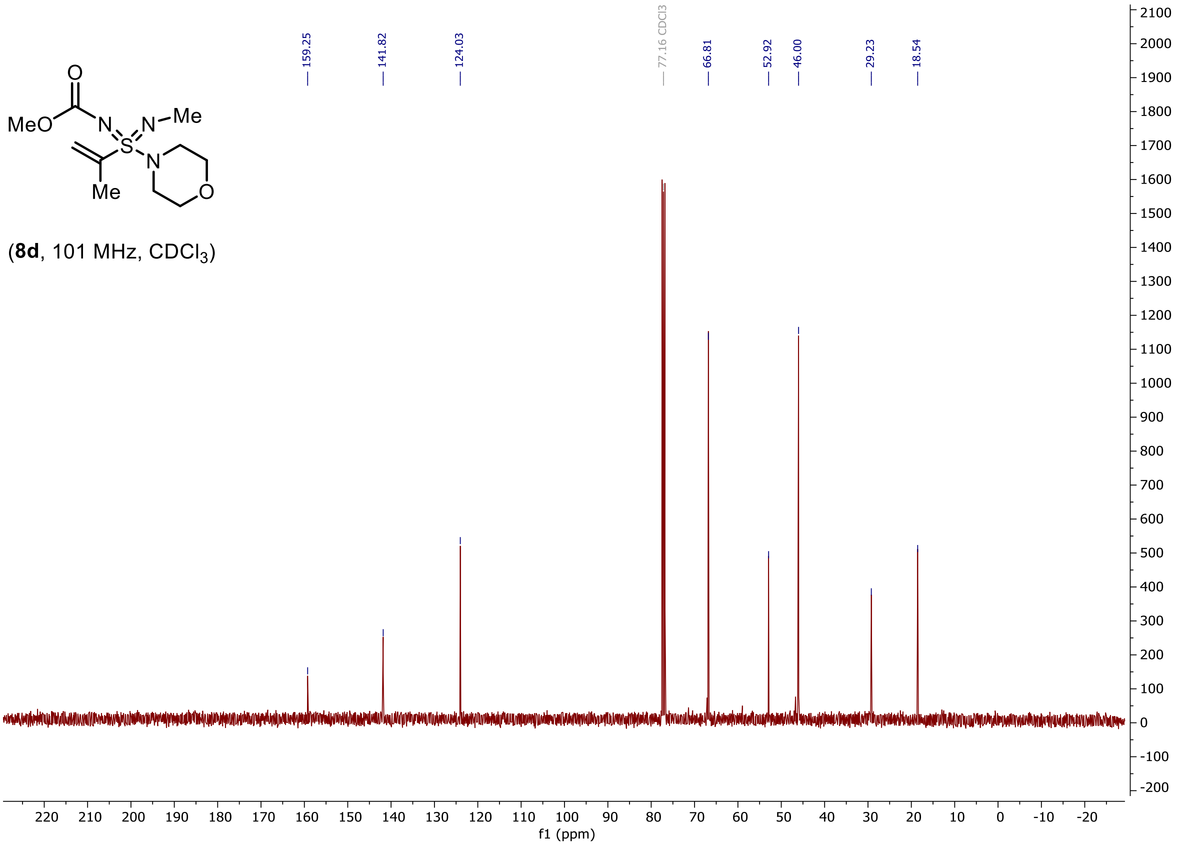

(**8d**, 101 MHz, $CDCl_3$)

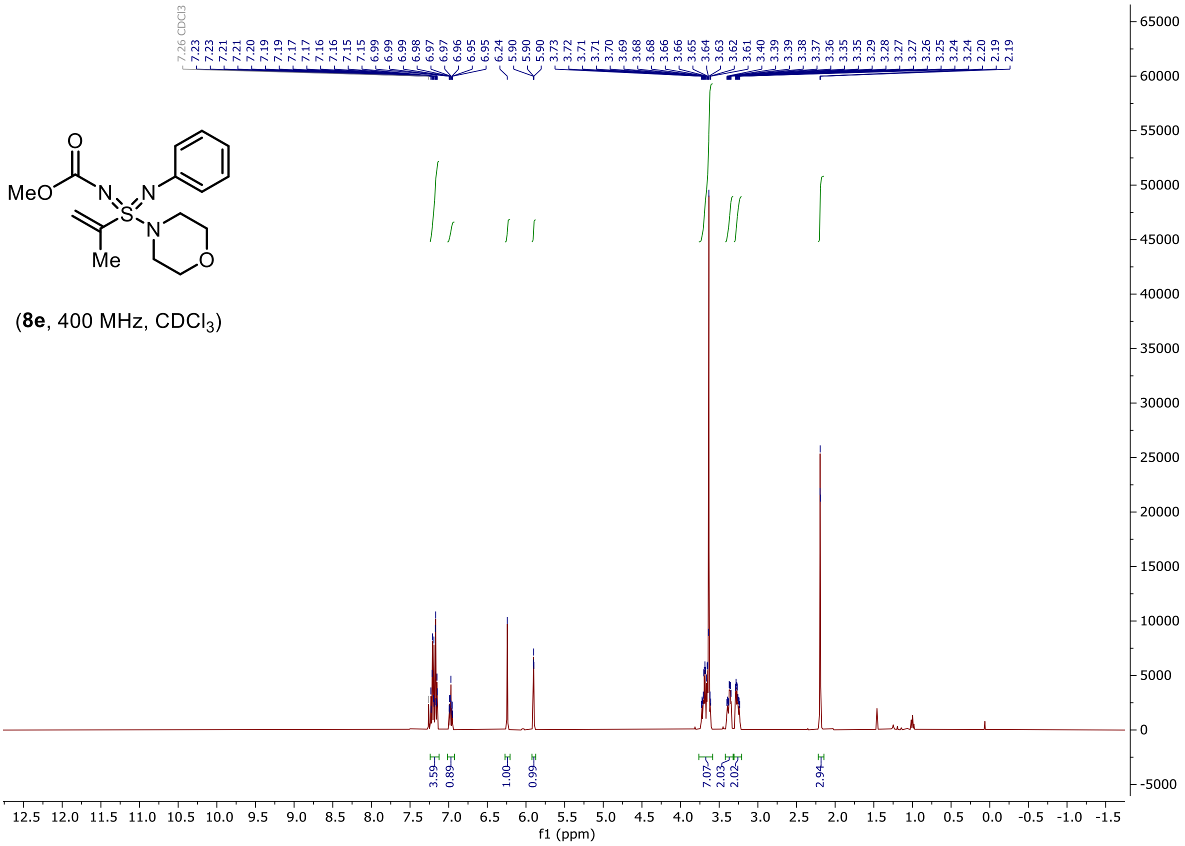
(8e, 400 MHz, CDCl3)
7.26 CDCl3
3.59
0.89
1.00
0.99
7.07
2.03
2.02
2.94
f1 (ppm)

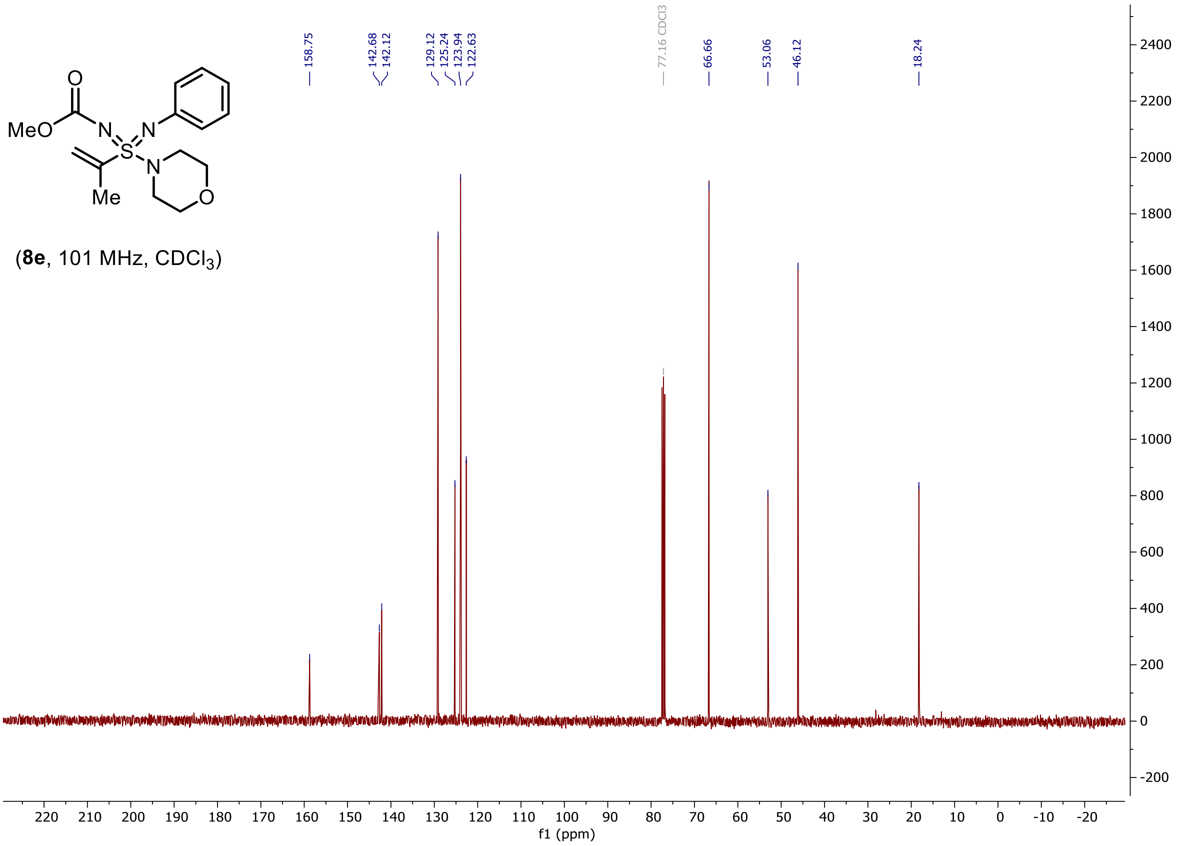
(8e, 101 MHz, CDCl3)
158.75
142.68
142.12
129.12
125.24
123.94
122.63
77.16 CDCl3
66.66
53.06
46.12
18.24
f1 (ppm)

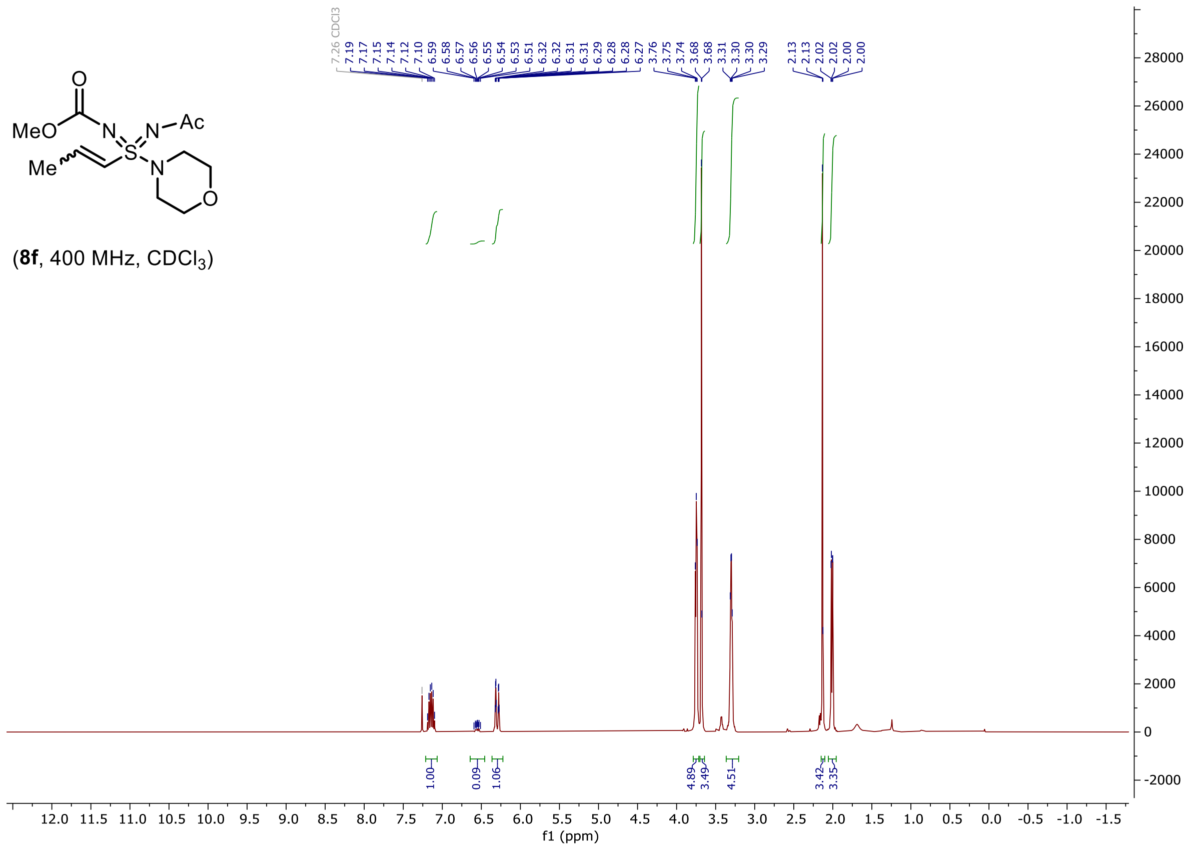

MeO
O
N
S
N
Ac
Me
N
O
(8f, 400 MHz, CDCl3)
7.26 CDCl3
7.19 7.17 7.15 7.14 7.12 7.10 6.59 6.58 6.57 6.56 6.55 6.54 6.53 6.51 6.32 6.32 6.31 6.31 6.29 6.28 6.28 6.27 3.76 3.75 3.74 3.68 3.68 3.31 3.30 3.30 3.29 2.13 2.13 2.02 2.02 2.00 2.00
1.00 0.09 1.06 4.89 3.49 4.51 3.42 3.35
f1 (ppm)

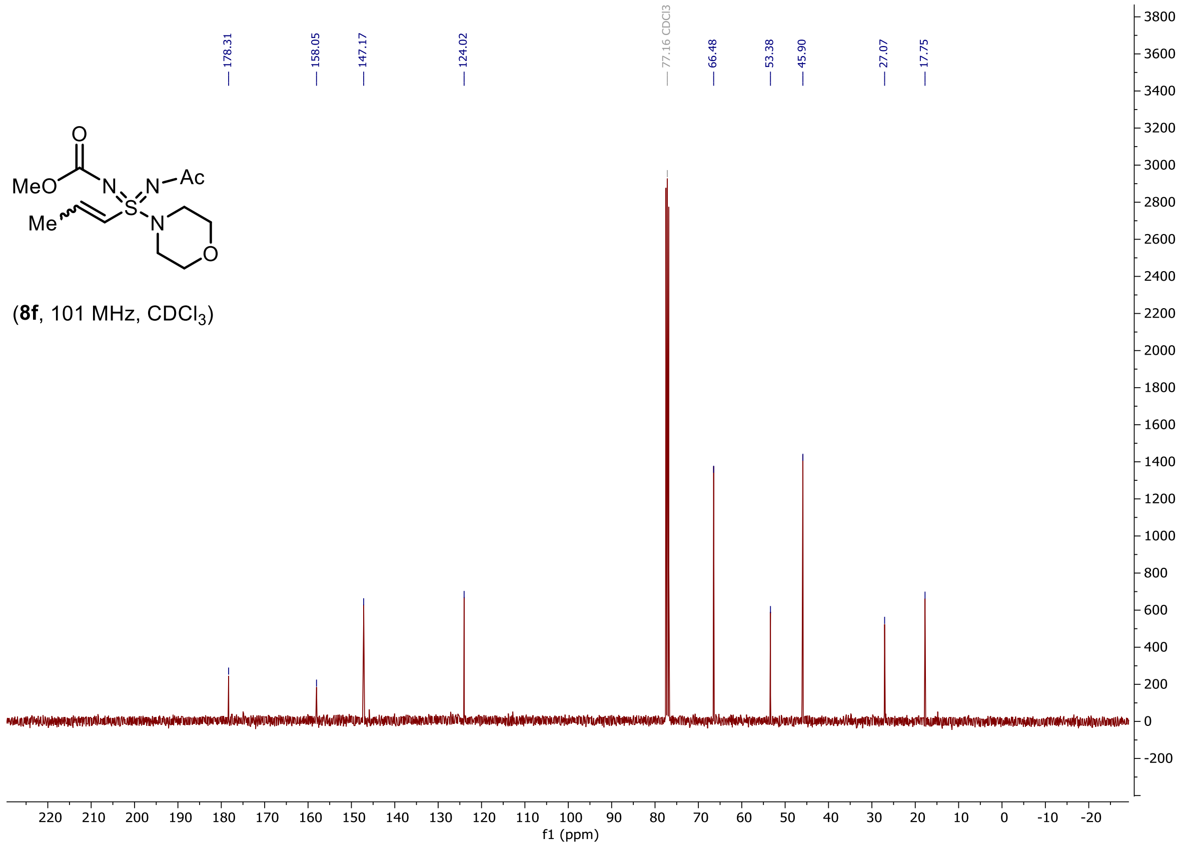

MeO
O
N
S
N
Ac
Me
N
O
(8f, 101 MHz, CDCl3)
178.31
158.05
147.17
124.02
77.16 CDCl3
66.48
53.38
45.90
27.07
17.75
f1 (ppm)

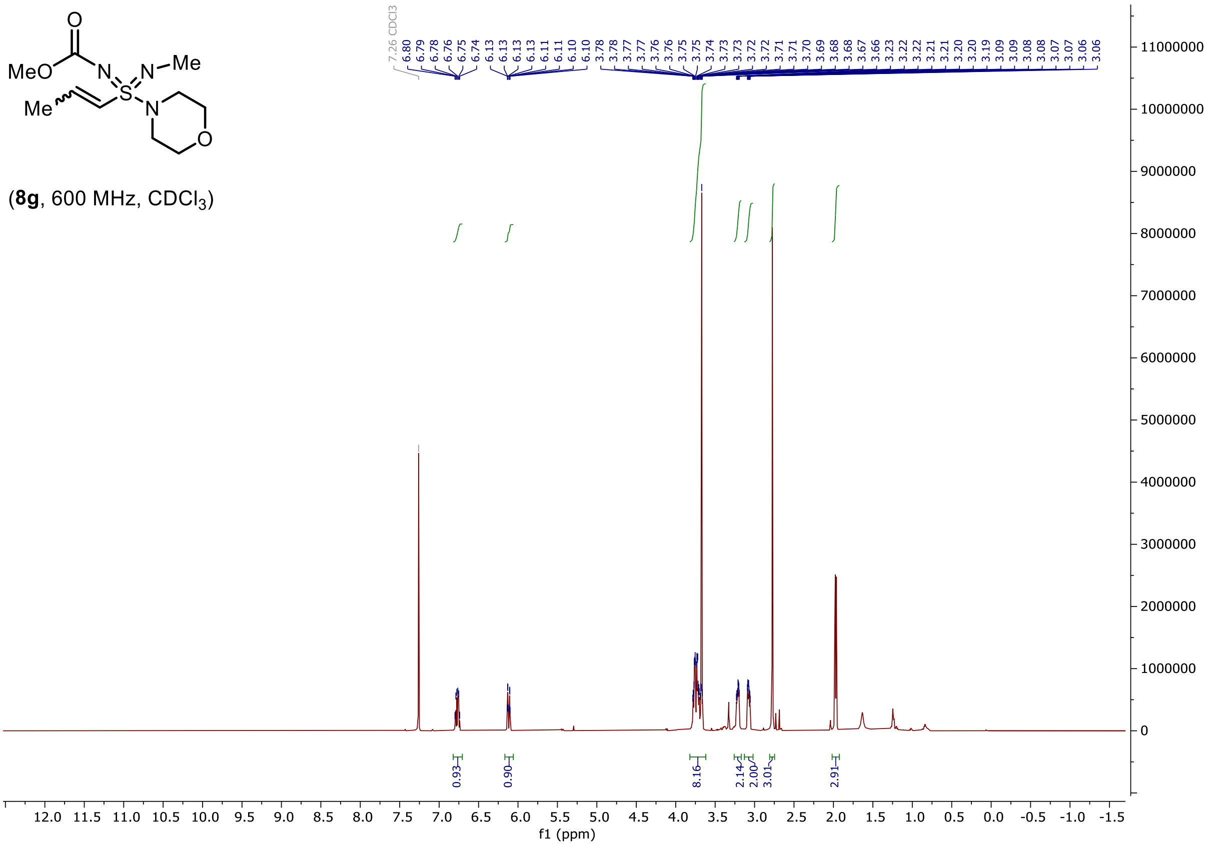
MeO
N
N–Me
S
N
O
Me
(8g, 600 MHz, CDCl3)
7.26 CDCl3
0.93
0.90
8.16
2.14
2.00
3.01
2.91
f1 (ppm)

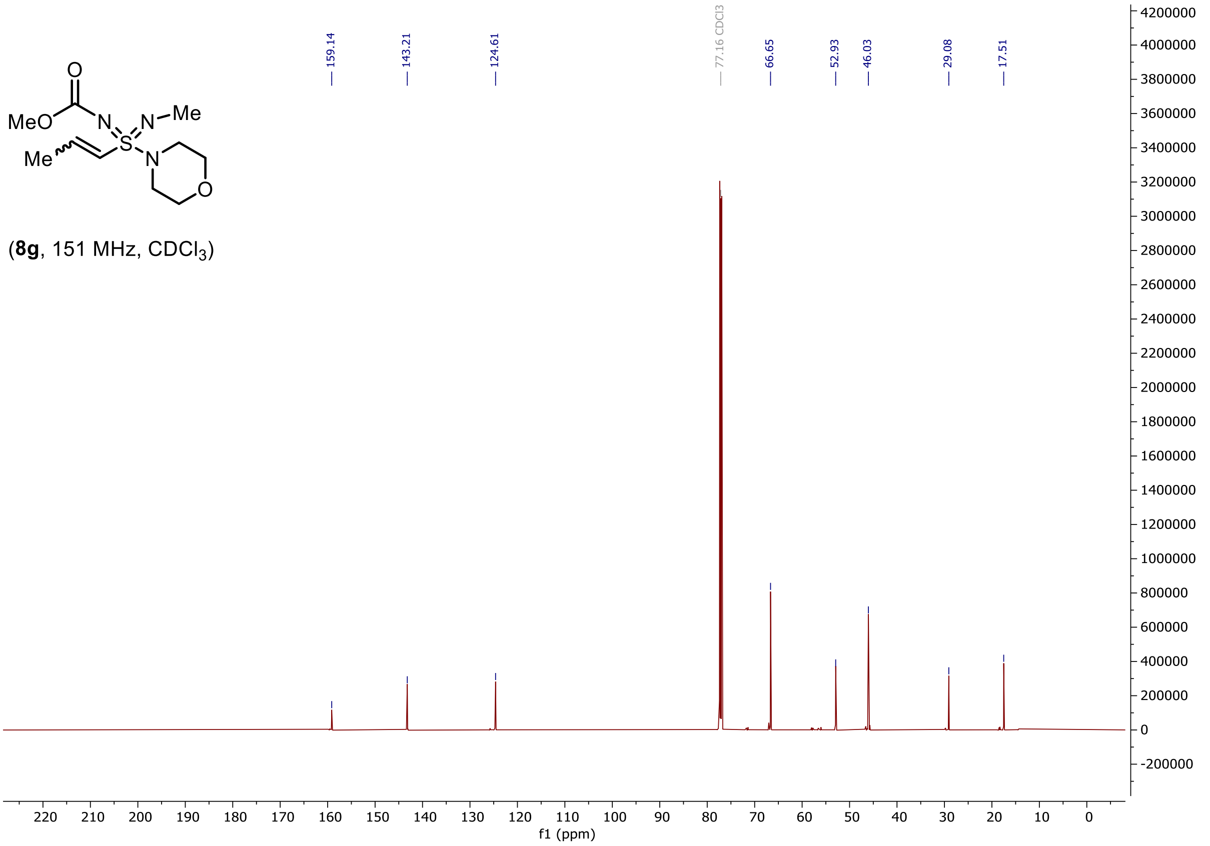
MeO
N
N–Me
S
N
O
Me
(8g, 151 MHz, CDCl3)
159.14
143.21
124.61
77.16 CDCl3
66.65
52.93
46.03
29.08
17.51
f1 (ppm)

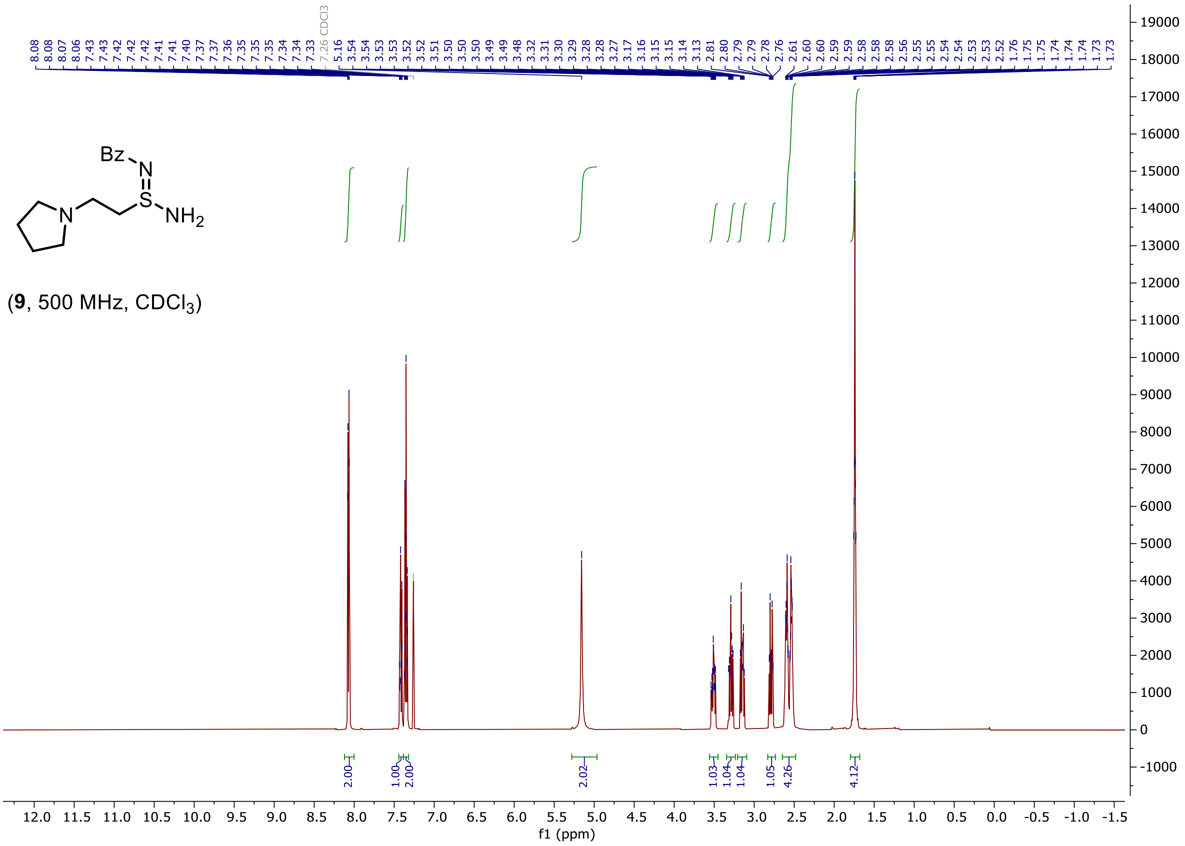
Bz
N
S
NH2
N
(9, 500 MHz, CDCl3)
f1 (ppm)

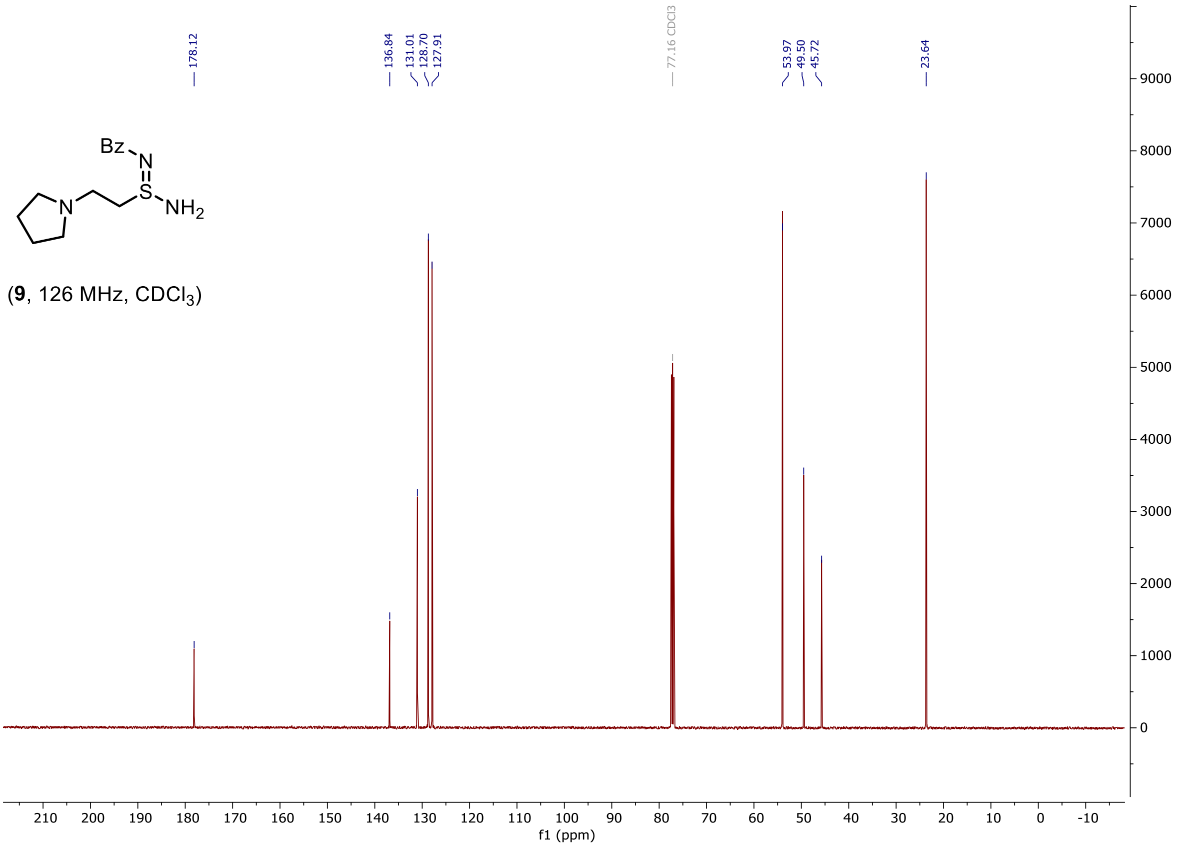
178.12
136.84
131.01
128.70
127.91
77.16 CDCl3
53.97
49.50
45.72
23.64
Bz
N
S
NH2
N
(9, 126 MHz, CDCl3)
f1 (ppm)

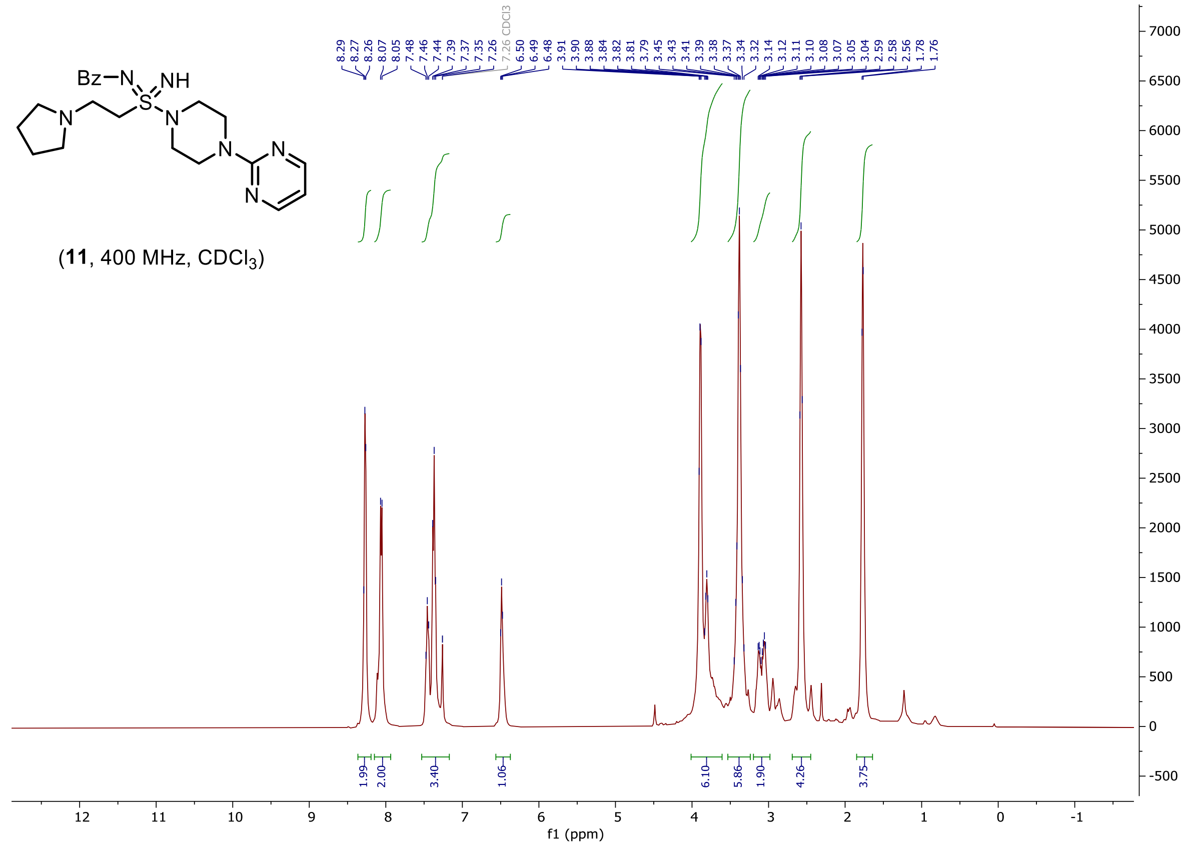

(**11**, 400 MHz, $CDCl_3$)

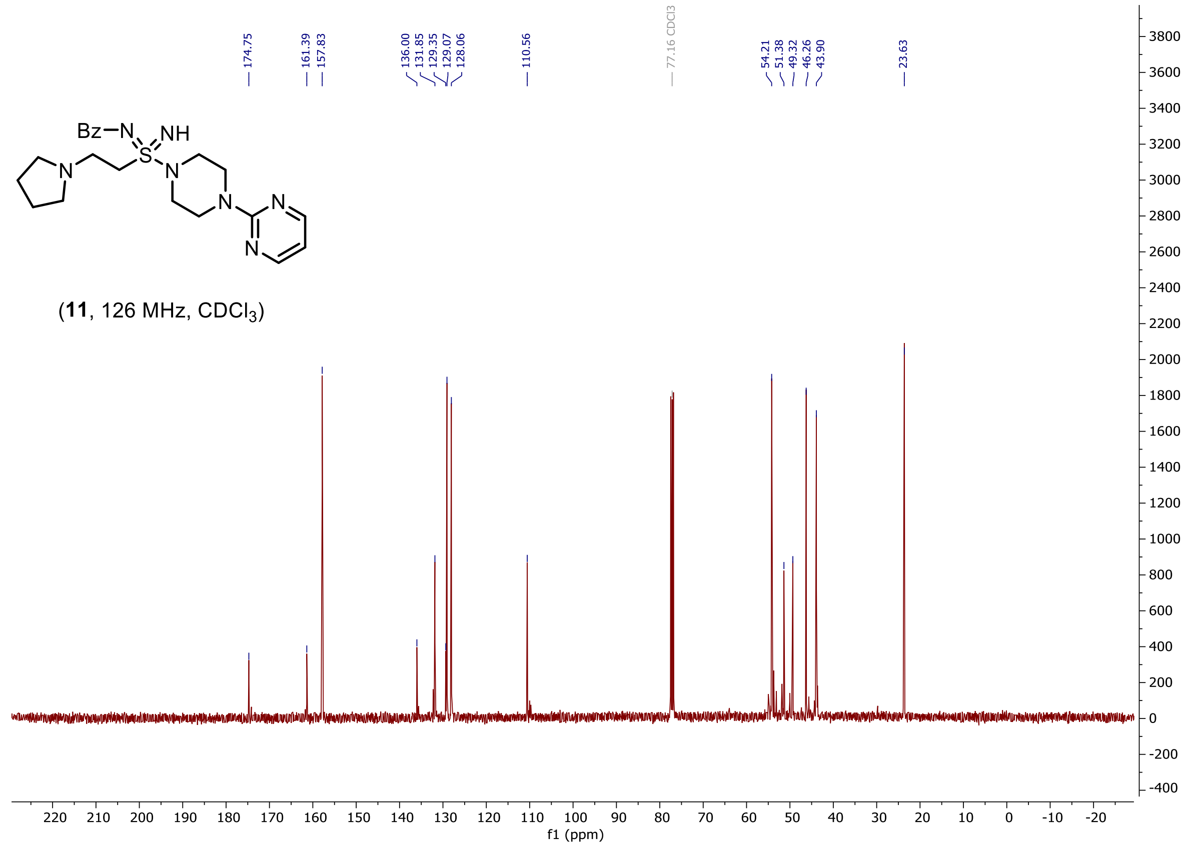

(**11**, 126 MHz, $CDCl_3$)

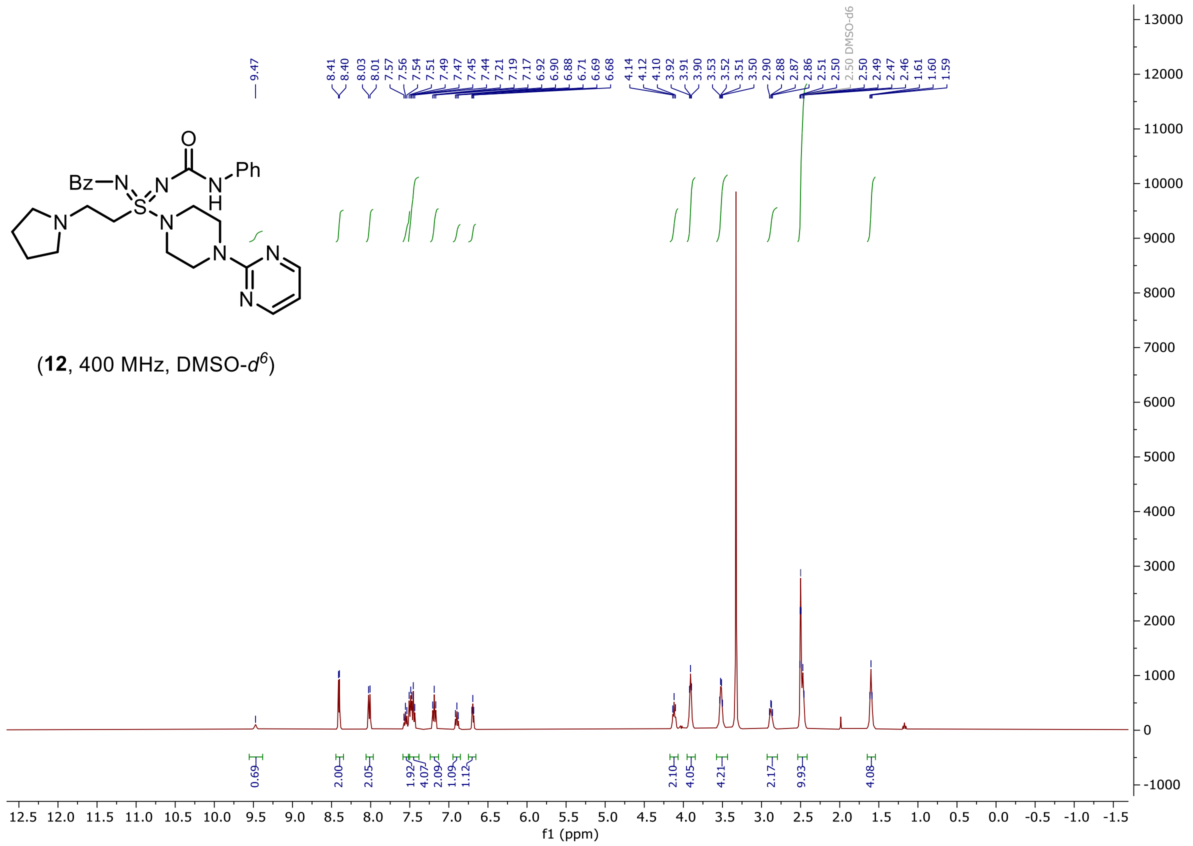

(**12**, 400 MHz, DMSO-d^6)

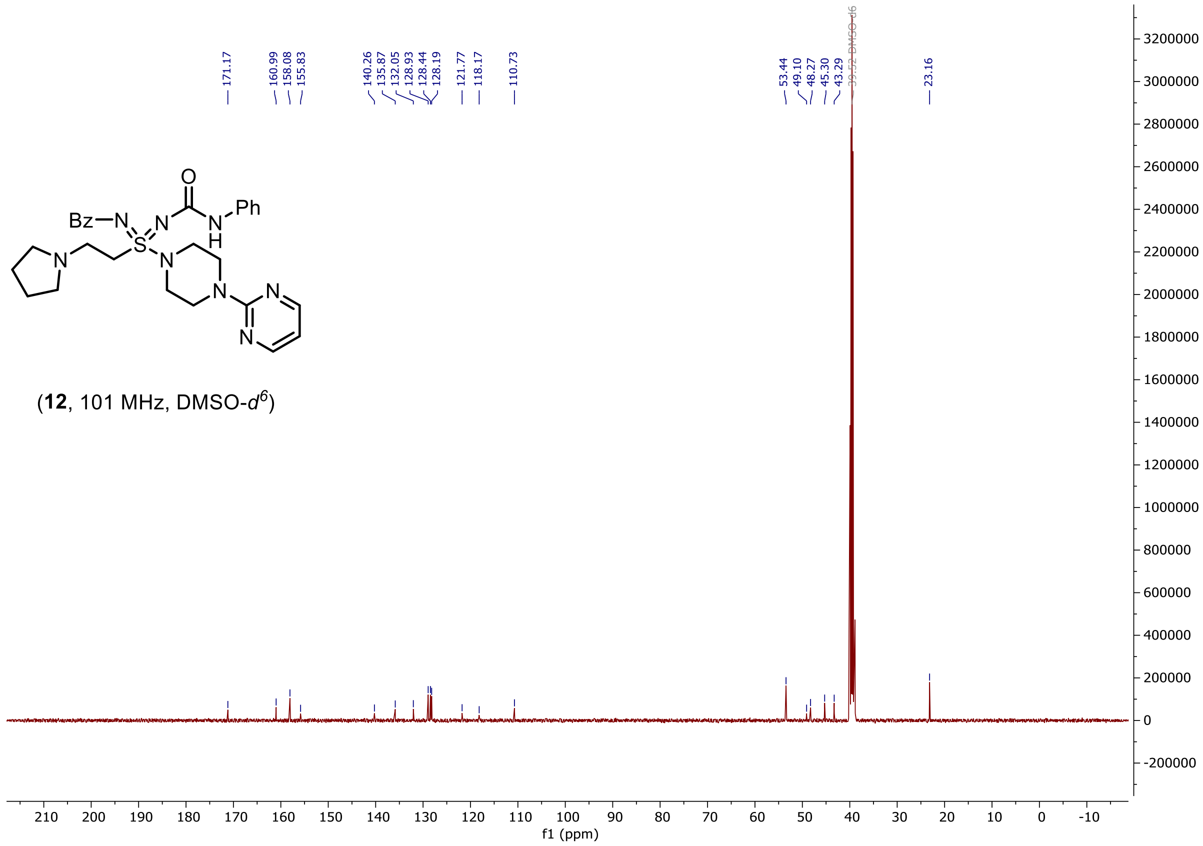

(**12**, 101 MHz, DMSO-d^6)

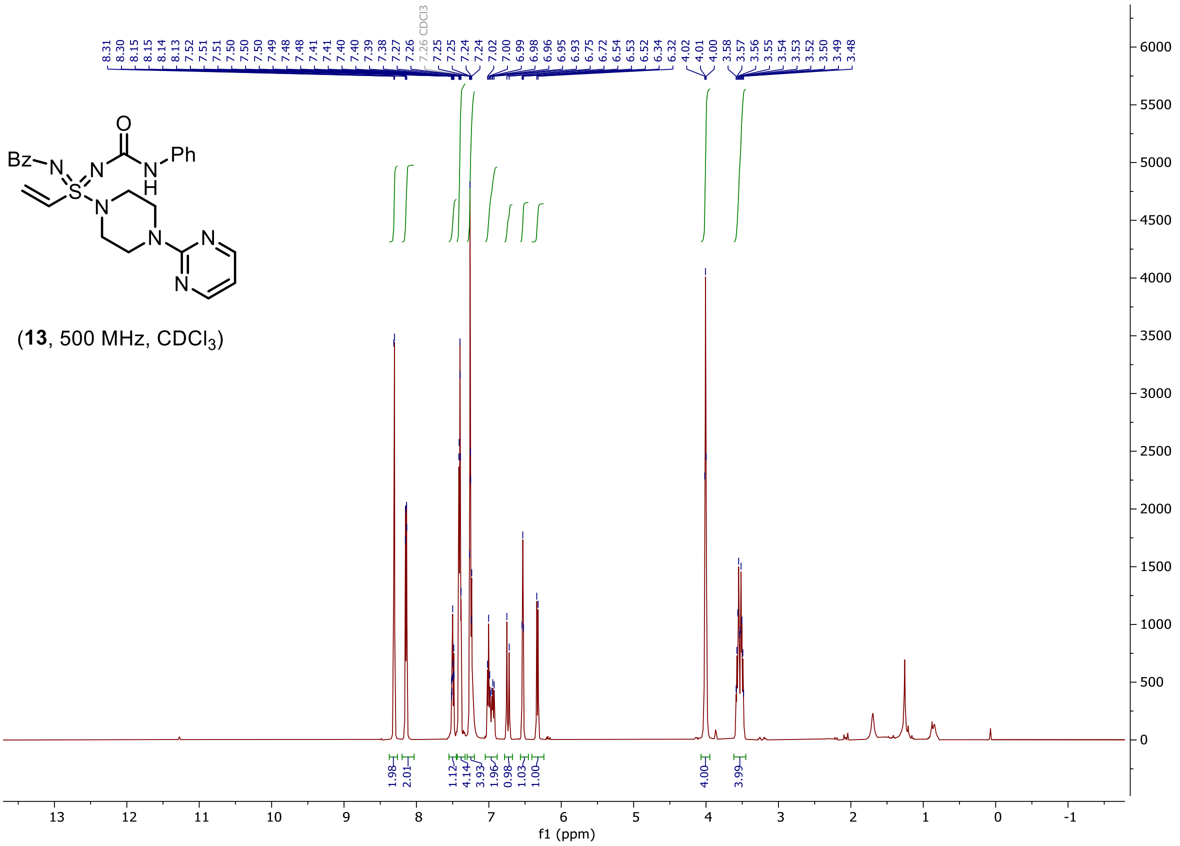

(**13**, 500 MHz, $CDCl_3$)

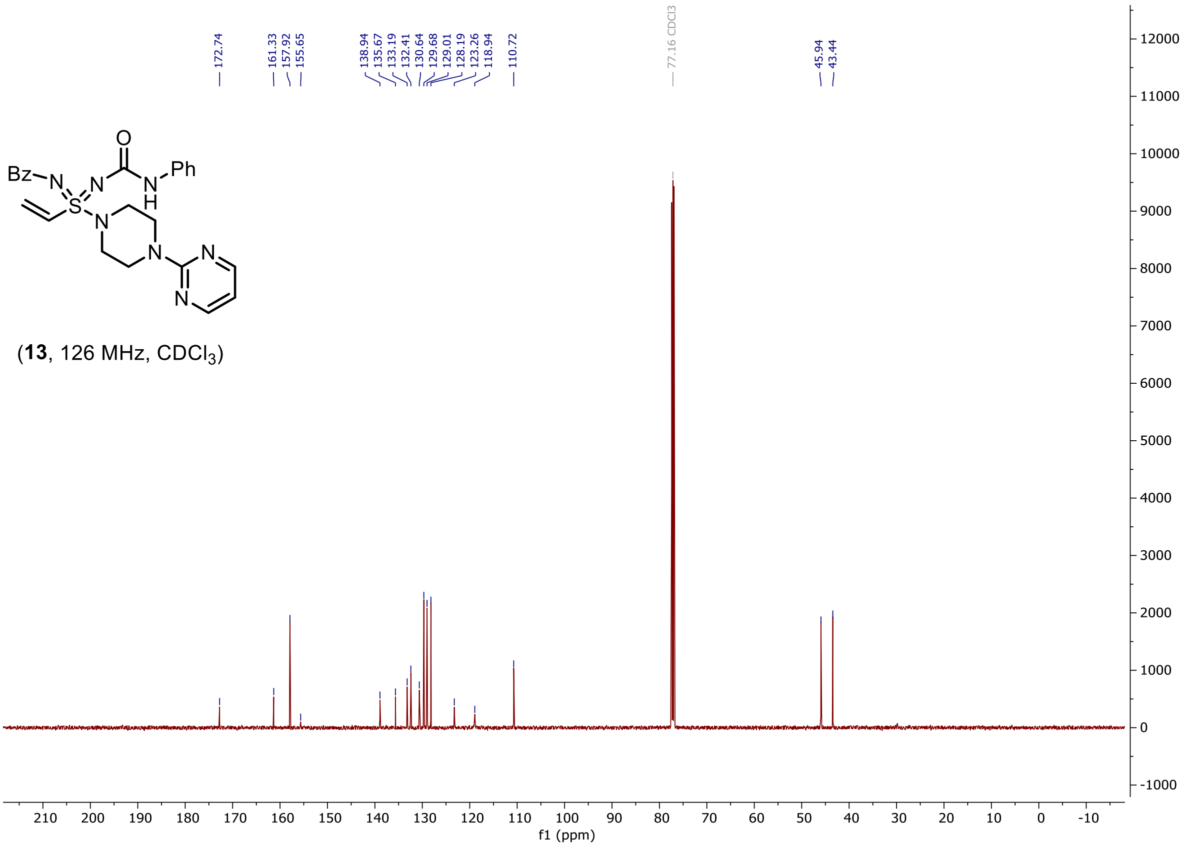

(**13**, 126 MHz, $CDCl_3$)

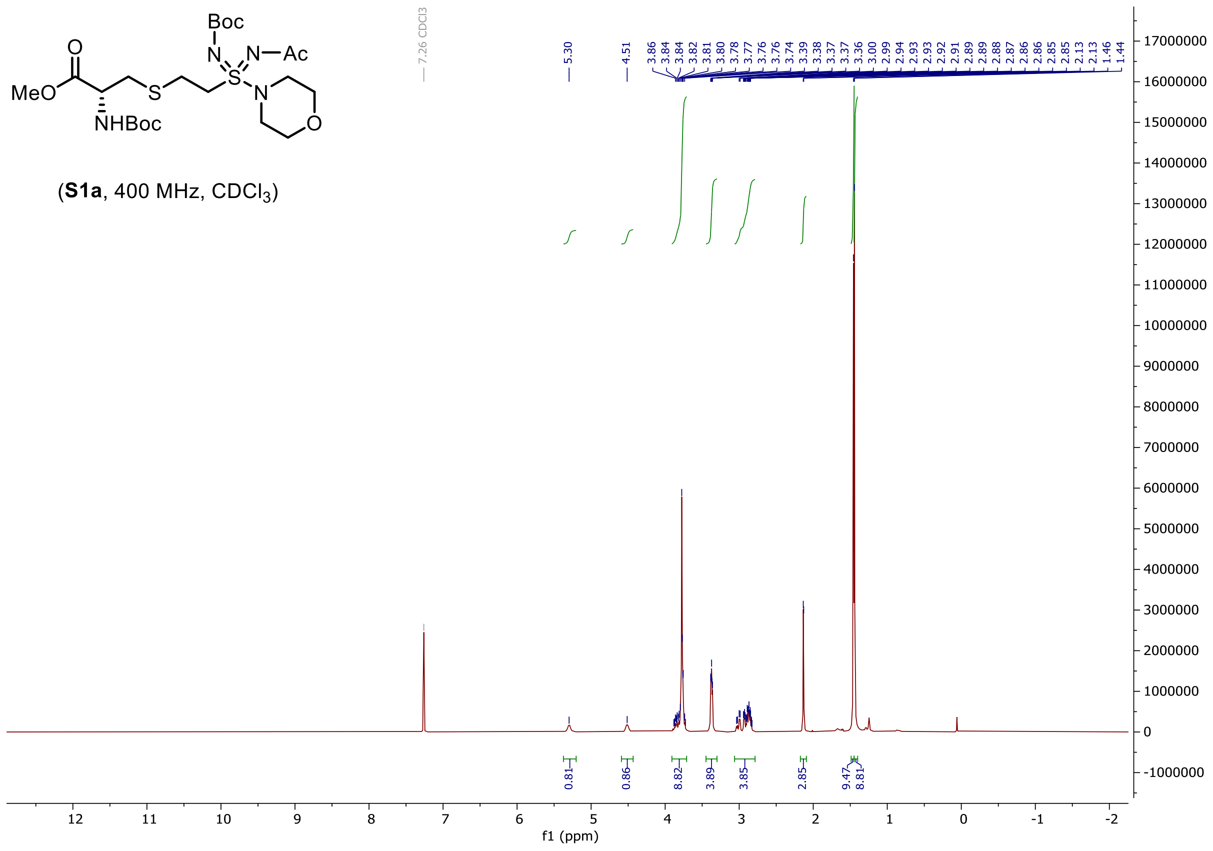
(S1a, 400 MHz, CDCl3)
f1 (ppm)

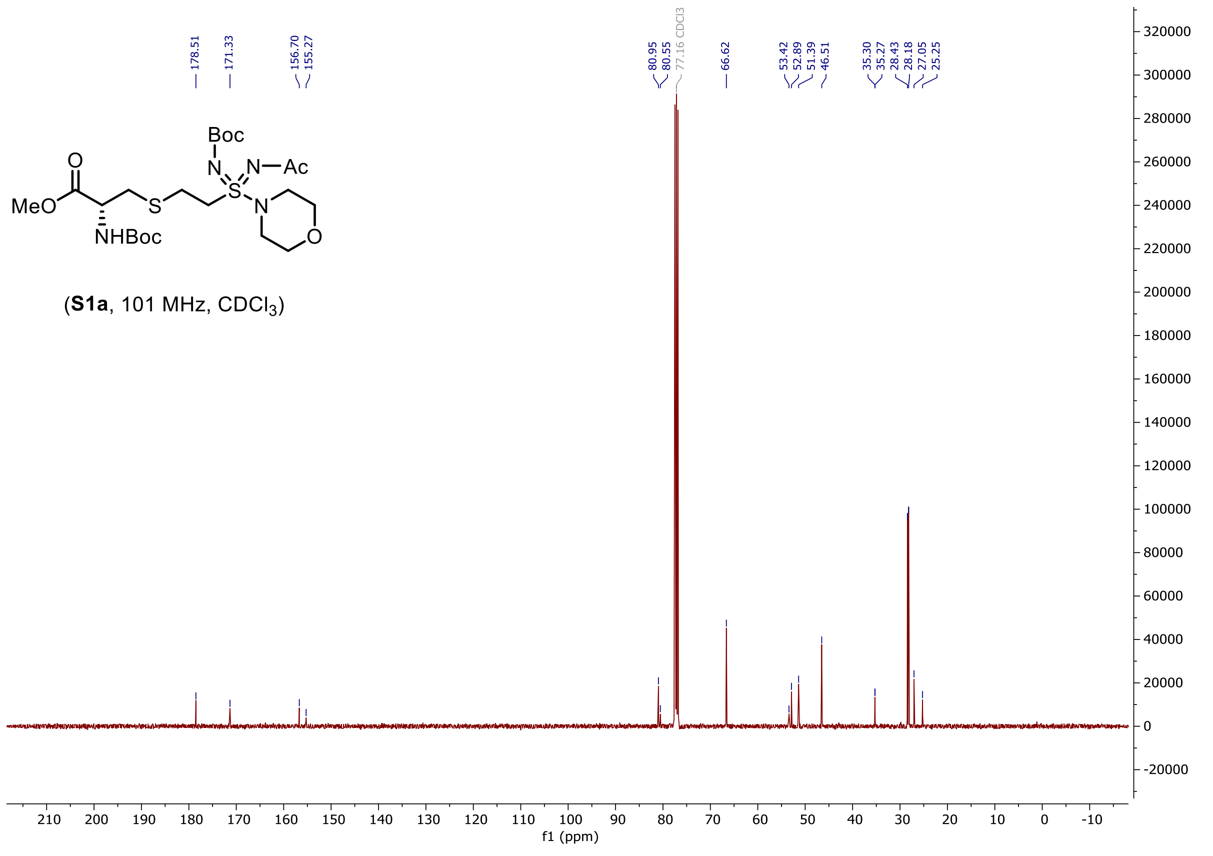
(S1a, 101 MHz, CDCl3)
f1 (ppm)

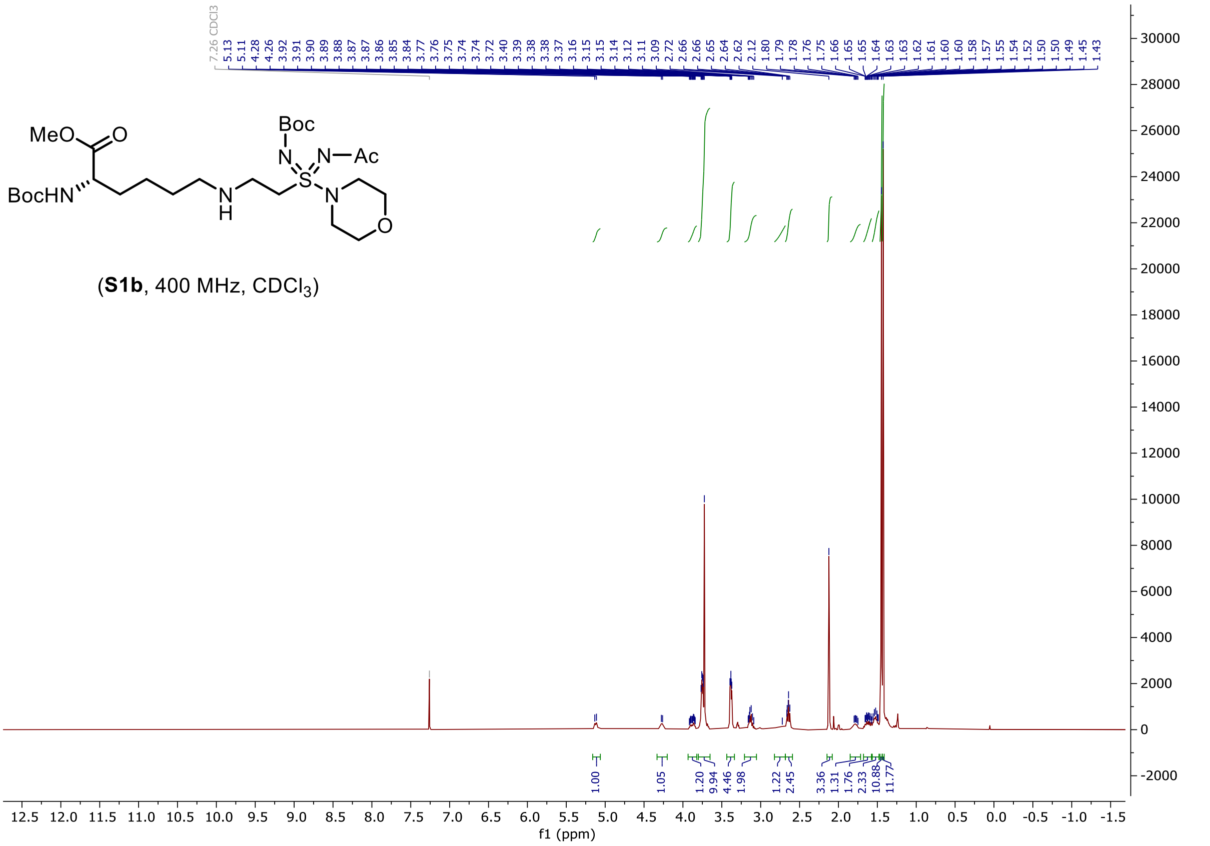

(**S1b**, 400 MHz, $CDCl_3$)

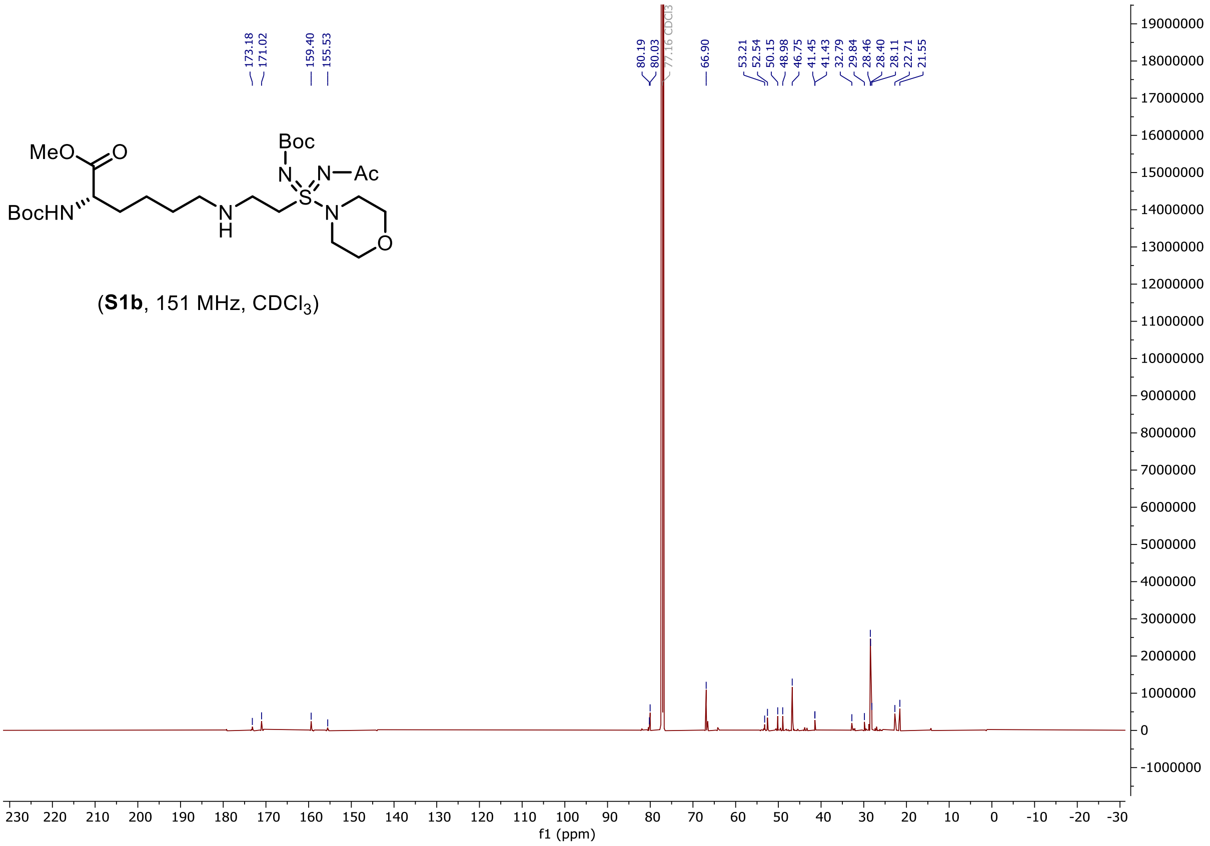

(**S1b**, 151 MHz, $CDCl_3$)

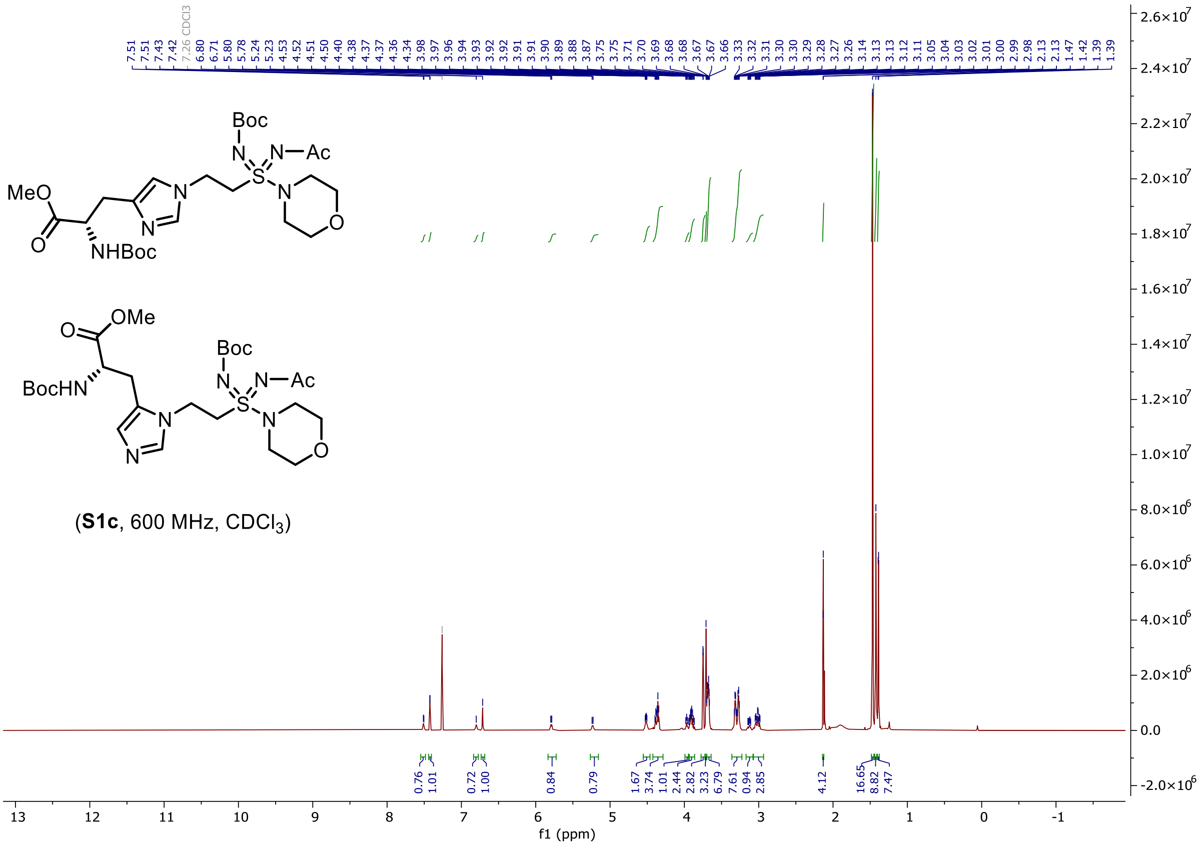

(**S1c**, 600 MHz, $CDCl_3$)

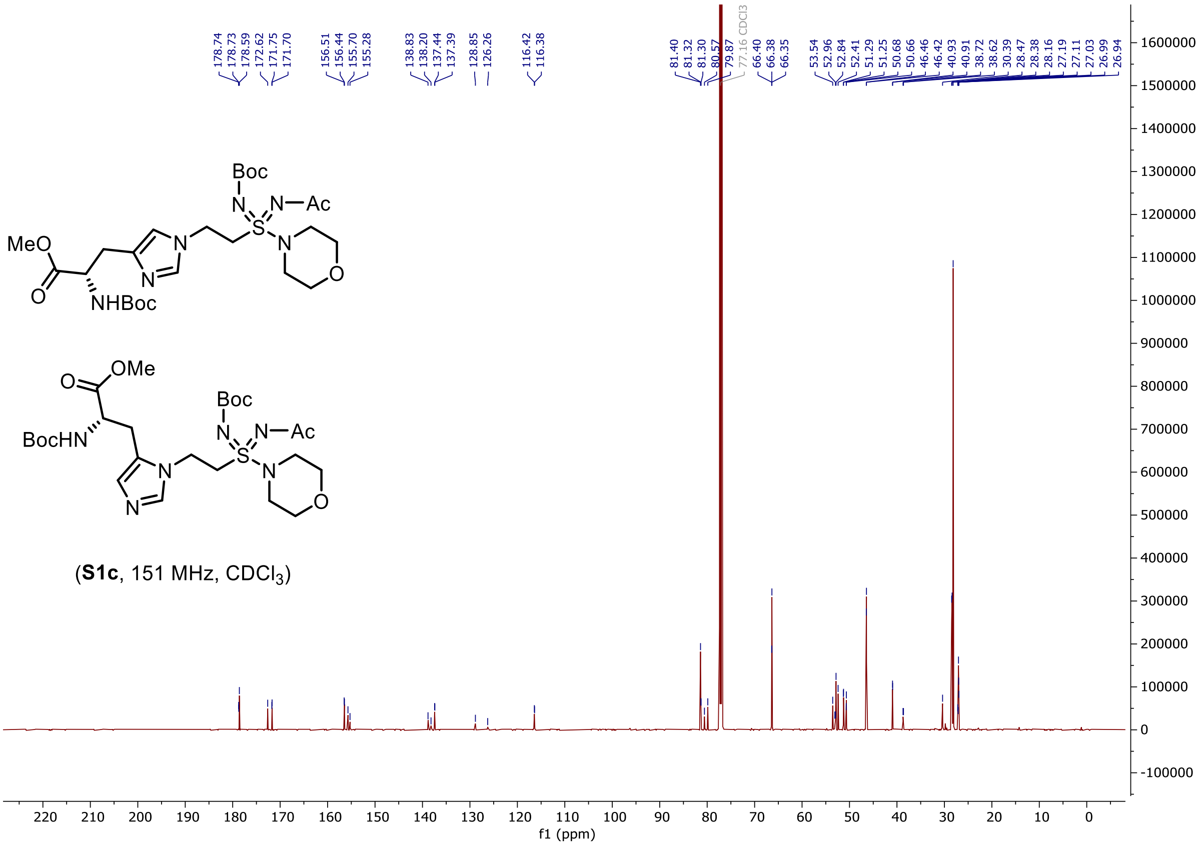

(**S1c**, 151 MHz, $CDCl_3$)

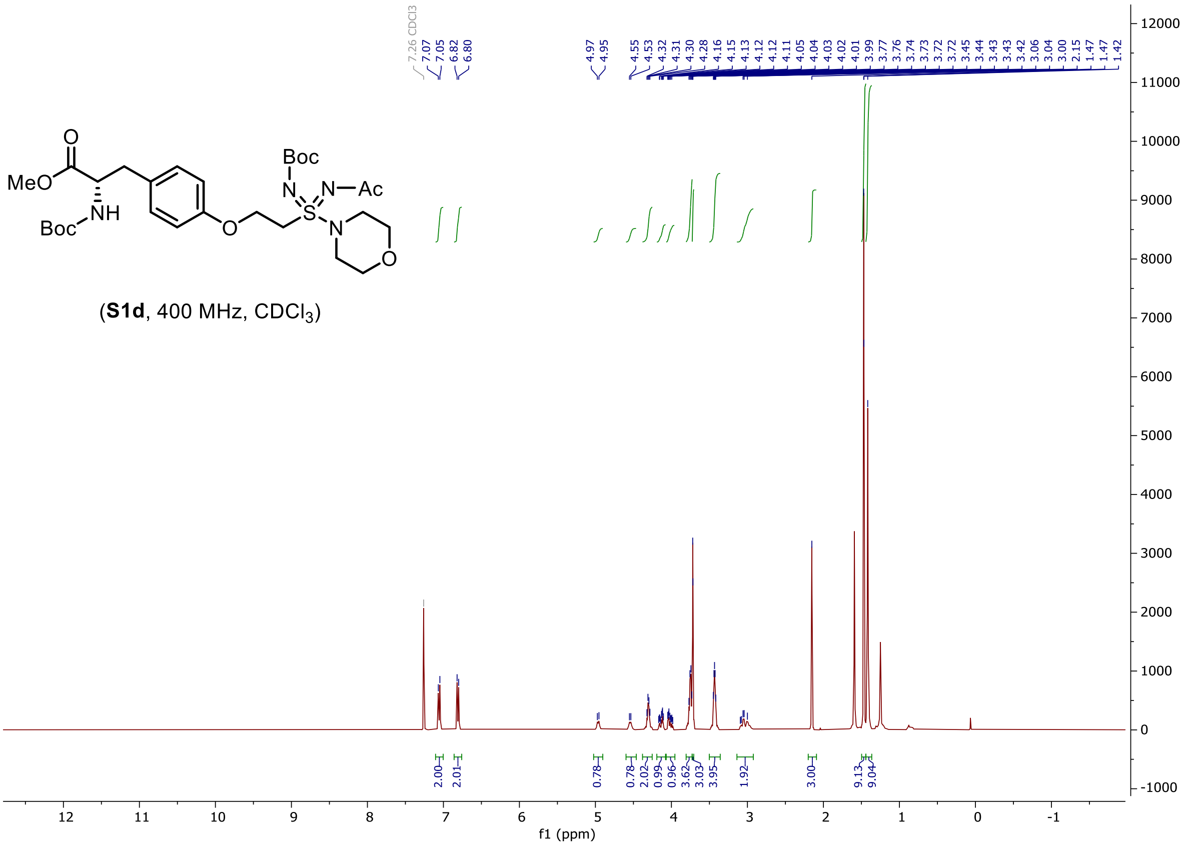

(**S1d**, 400 MHz, $CDCl_3$)

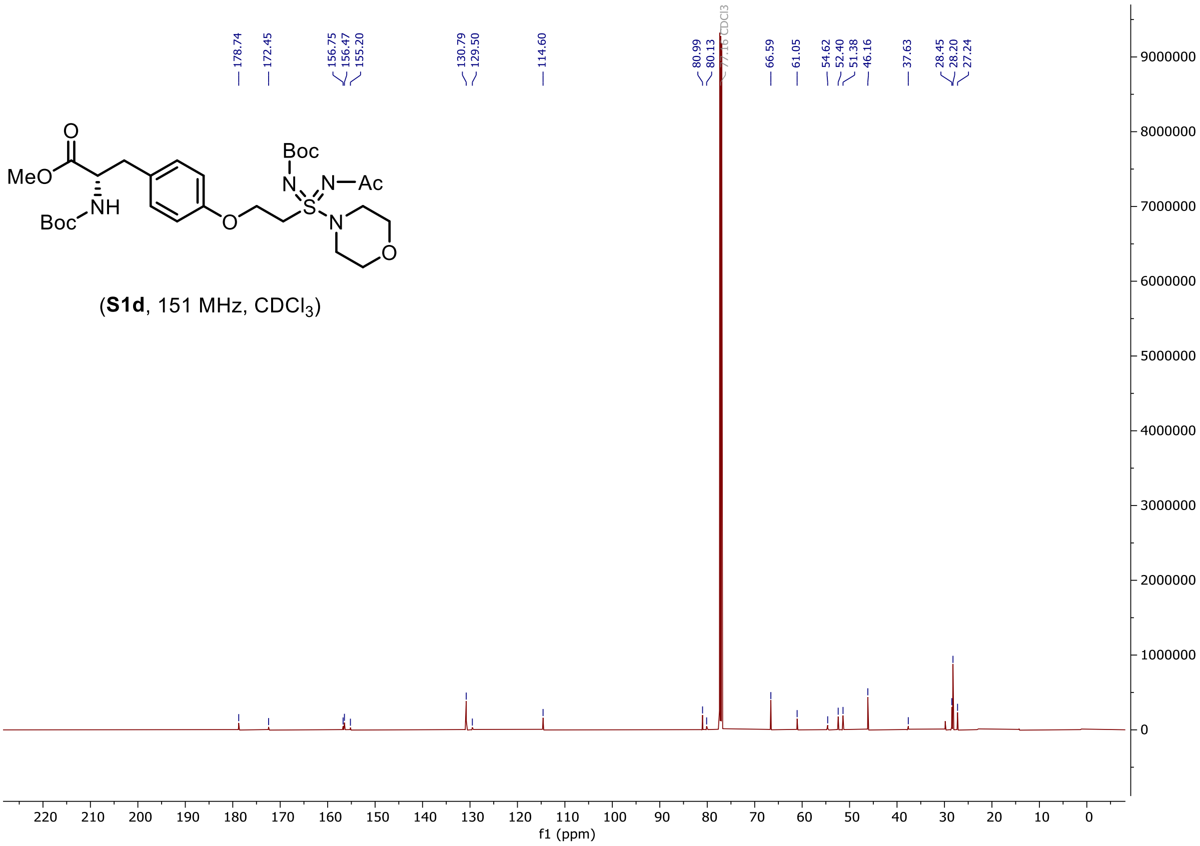

(**S1d**, 151 MHz, $CDCl_3$)

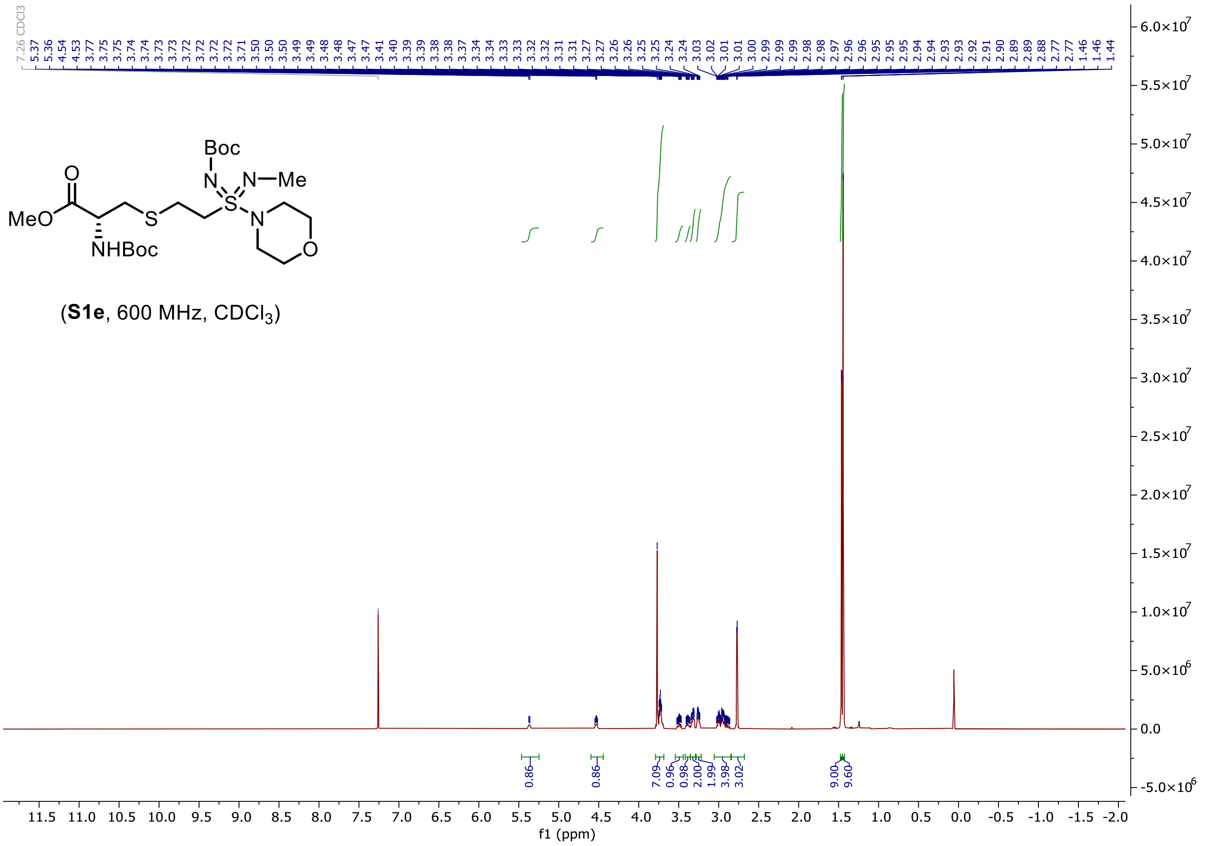

7.26 CDCl3
5.37
5.36
4.54
4.53
3.77
3.75
3.75
3.74
3.74
3.73
3.73
3.72
3.72
3.72
3.71
3.50
3.50
3.50
3.49
3.49
3.48
3.48
3.47
3.47
3.41
3.40
3.39
3.39
3.38
3.38
3.37
3.37
3.34
3.34
3.33
3.33
3.32
3.32
3.31
3.31
3.27
3.27
3.26
3.26
3.25
3.25
3.24
3.24
3.03
3.02
3.01
3.01
3.00
2.99
2.99
2.99
2.98
2.98
2.97
2.96
2.96
2.95
2.95
2.95
2.94
2.94
2.93
2.93
2.92
2.91
2.90
2.89
2.89
2.88
2.77
2.77
1.46
1.44
Boc
N
N—Me
S
N
O
MeO
O
S
NHBoc
(S1e, 600 MHz, CDCl3)
0.86
0.86
7.09
0.96
0.98
2.00
1.99
3.98
3.02
9.00
9.60
11.5 11.0 10.5 10.0 9.5 9.0 8.5 8.0 7.5 7.0 6.5 6.0 5.5 5.0 4.5 4.0 3.5 3.0 2.5 2.0 1.5 1.0 0.5 0.0 -0.5 -1.0 -1.5 -2.0
f1 (ppm)

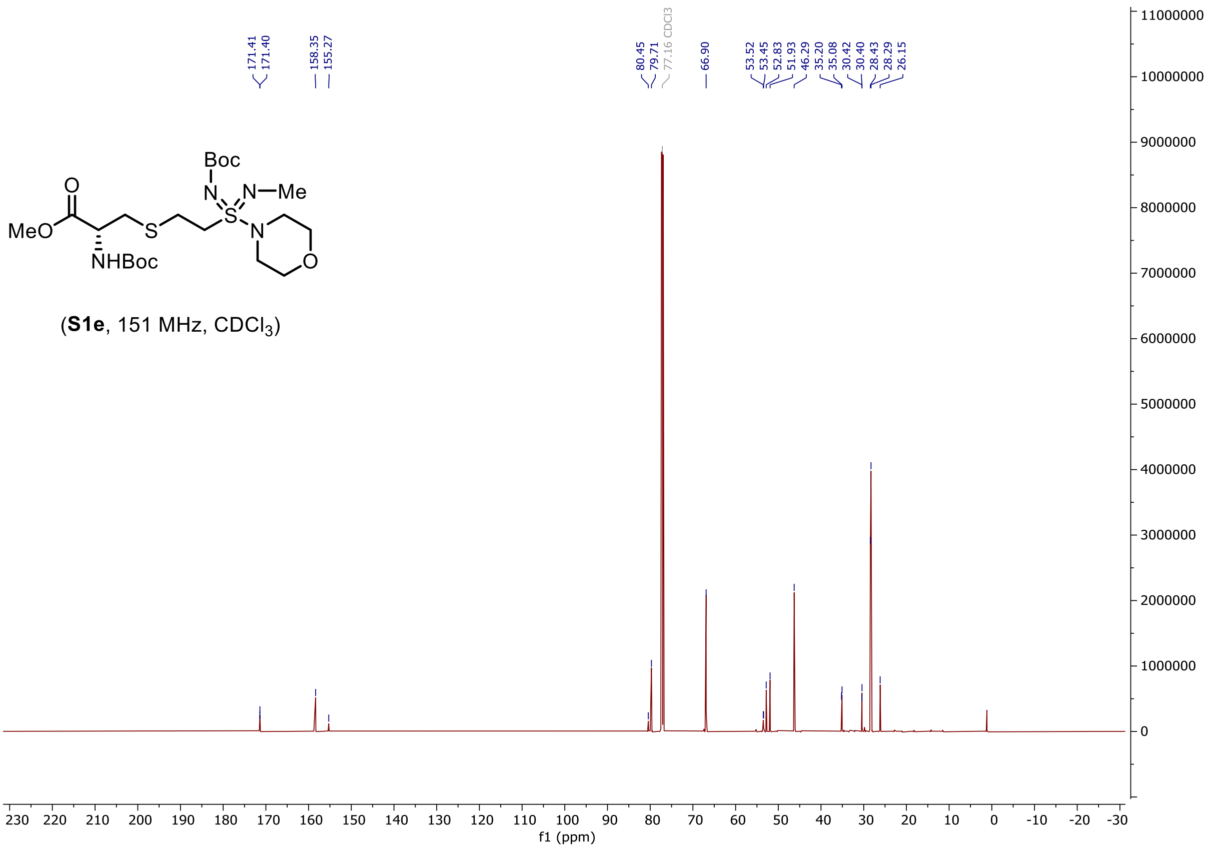

171.41
171.40
158.35
155.27
80.45
79.71
77.16 CDCl3
66.90
53.52
53.45
52.83
51.93
46.29
35.20
35.08
30.42
30.40
28.43
28.29
26.15
Boc
N
N—Me
S
N
O
MeO
O
S
NHBoc
(S1e, 151 MHz, CDCl3)
230 220 210 200 190 180 170 160 150 140 130 120 110 100 90 80 70 60 50 40 30 20 10 0 -10 -20 -30
f1 (ppm)

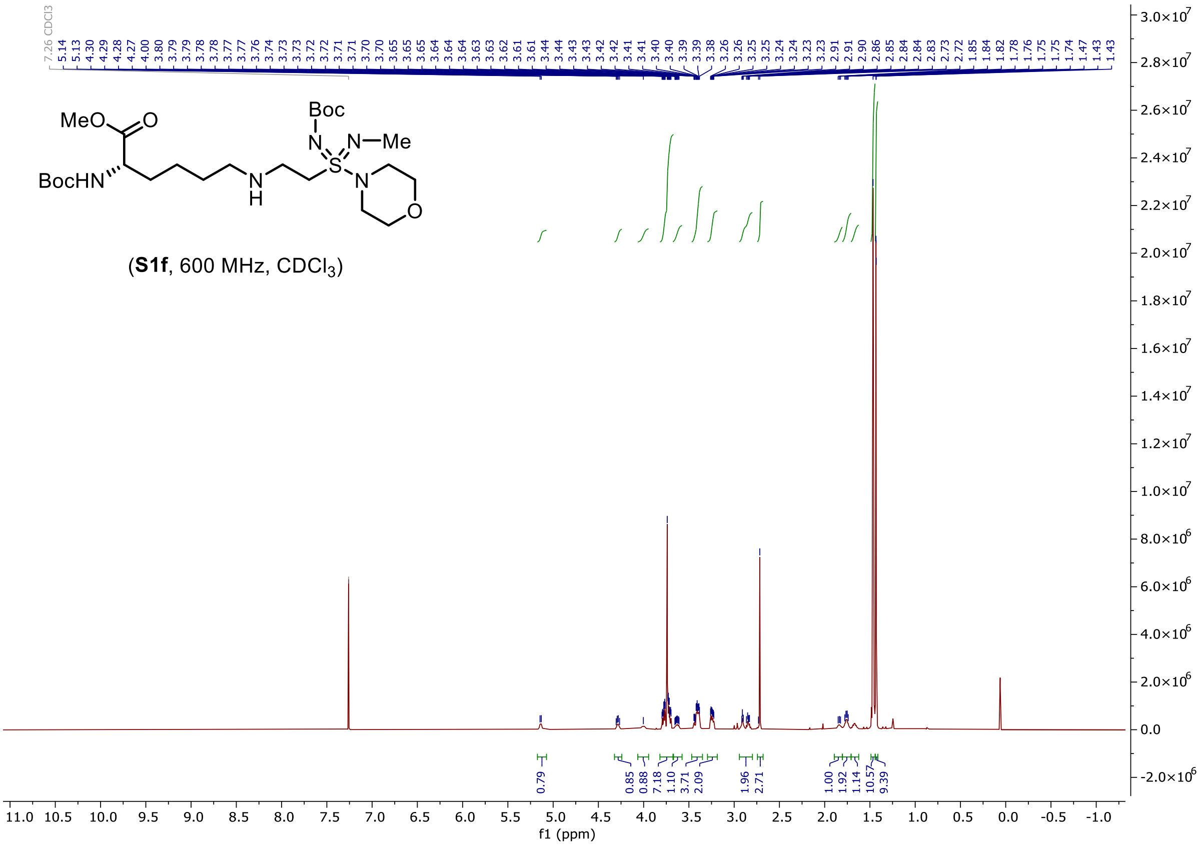
7.26 CDCl3
5.14 5.13 4.30 4.29 4.28 4.27 4.00 3.80 3.79 3.79 3.78 3.78 3.77 3.77 3.76 3.74 3.73 3.73 3.72 3.72 3.71 3.71 3.70 3.65 3.65 3.64 3.64 3.64 3.63 3.62 3.61 3.61 3.44 3.44 3.43 3.43 3.42 3.42 3.41 3.41 3.40 3.39 3.39 3.38 3.26 3.26 3.25 3.25 3.24 3.24 3.23 3.23 2.91 2.91 2.90 2.86 2.85 2.84 2.84 2.83 2.73 2.72 1.85 1.84 1.82 1.78 1.76 1.75 1.75 1.74 1.47 1.43
MeO
O
BocHN
N
H
Boc
N
N—Me
S
N
O
(**S1f**, 600 MHz, CDCl$_3$)
0.79 0.85 0.88 7.18 1.10 3.71 2.09 1.96 2.71 1.00 1.92 1.14 10.57 9.39
f1 (ppm)

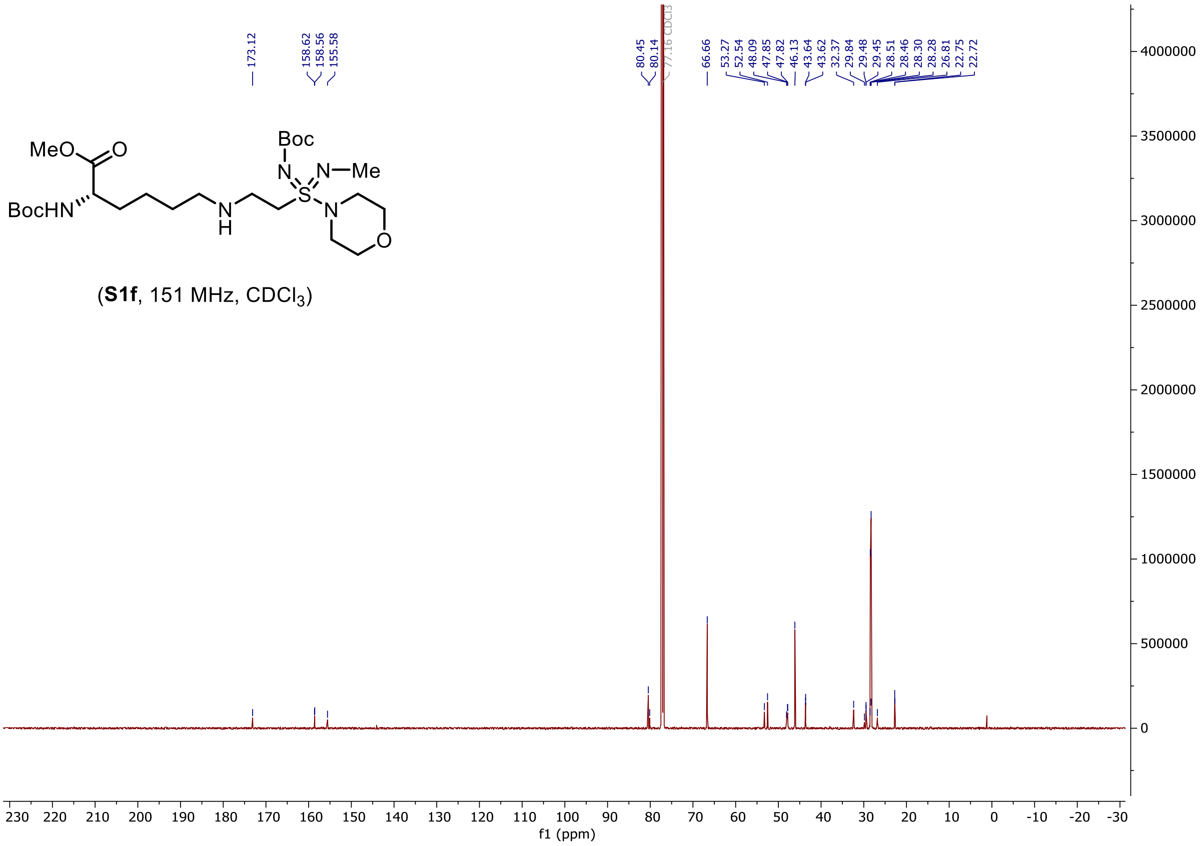
173.12
158.62 158.56 155.58
80.45 80.14 77.16 CDCl3
66.66
53.27 52.54 48.09 47.85 47.82 46.13 43.64 43.62 32.37 29.84 29.48 29.45 28.51 28.46 28.30 28.28 26.81 22.75 22.72
MeO
O
BocHN
N
H
Boc
N
N—Me
S
N
O
(**S1f**, 151 MHz, CDCl$_3$)
f1 (ppm)

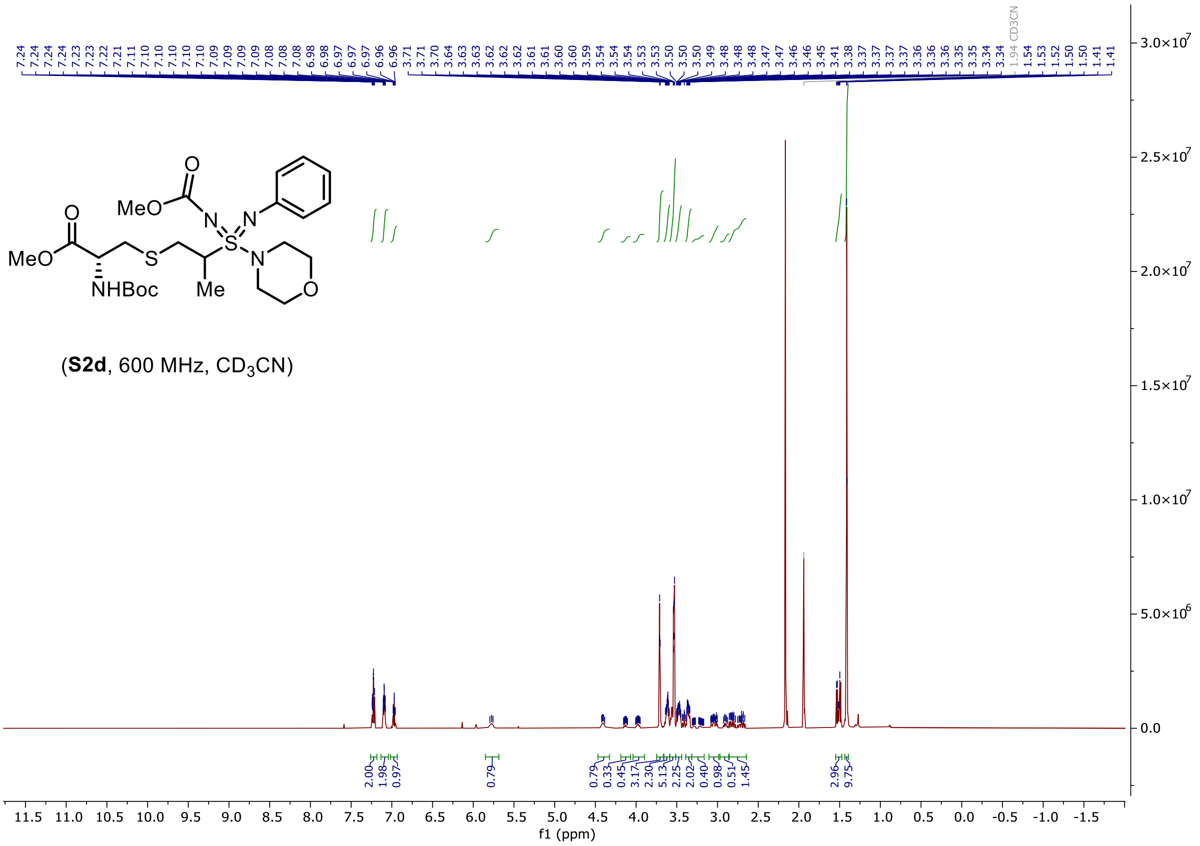
(S2d, 600 MHz, CD3CN)
f1 (ppm)

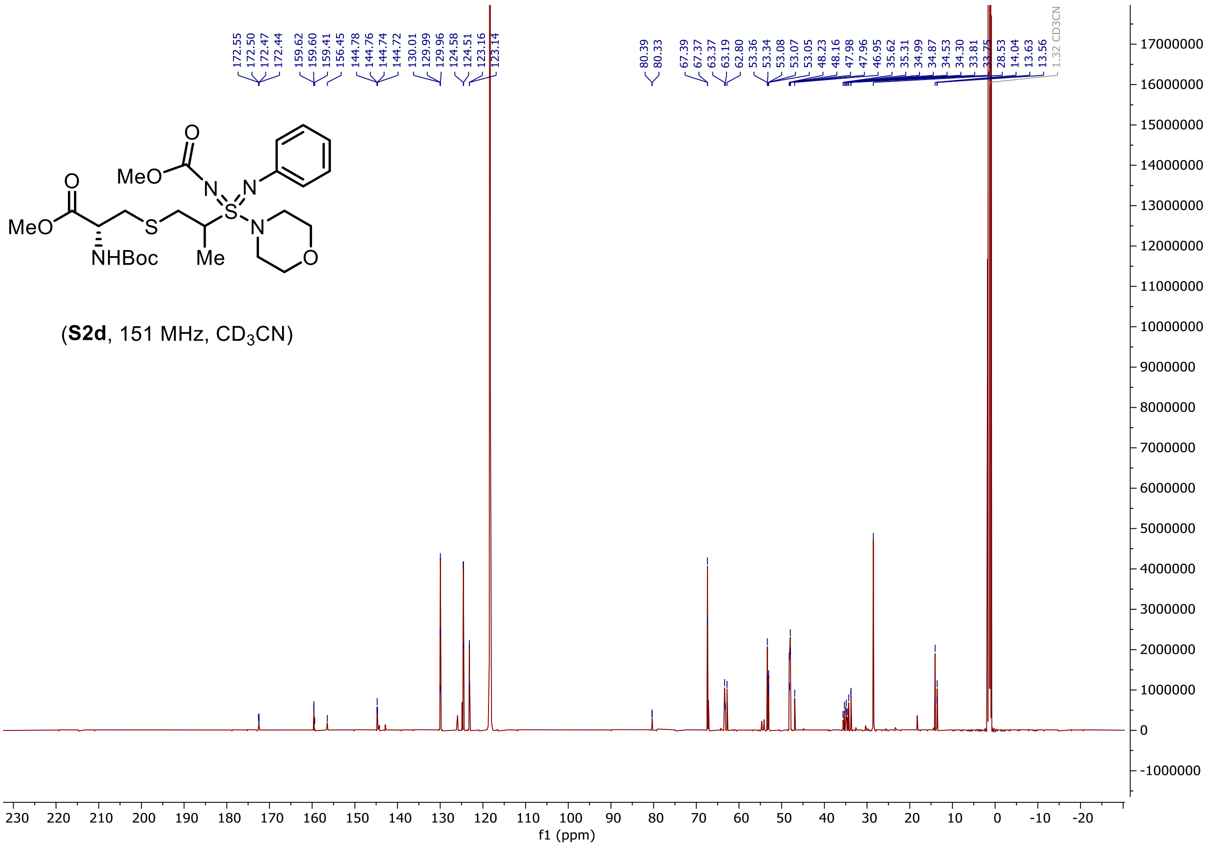
(S2d, 151 MHz, CD3CN)
f1 (ppm)

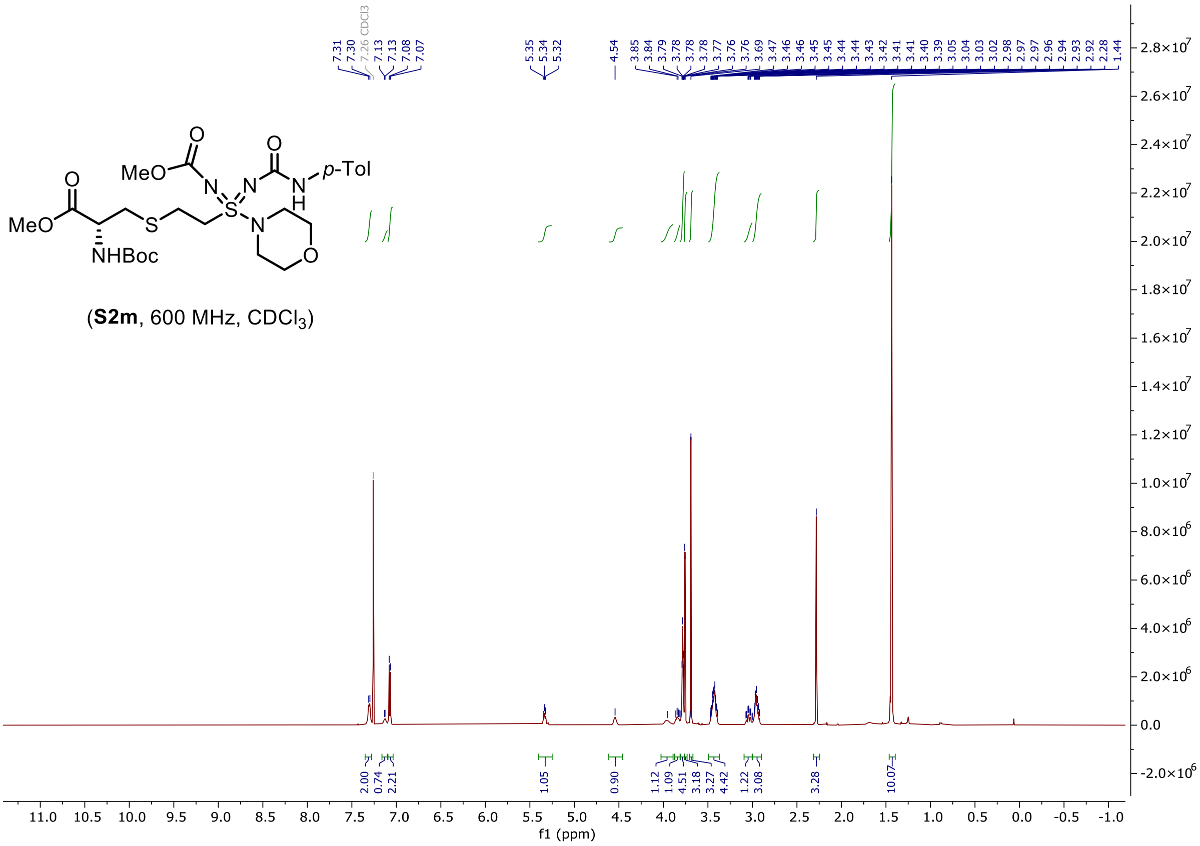
7.31 7.30 7.26 CDCl3 7.13 7.13 7.08 7.07 5.35 5.34 5.32 4.54 3.85 3.84 3.79 3.78 3.78 3.78 3.77 3.76 3.69 3.47 3.46 3.46 3.45 3.45 3.44 3.44 3.43 3.42 3.41 3.40 3.39 3.05 3.04 3.03 3.02 2.98 2.97 2.97 2.96 2.94 2.93 2.92 2.28 1.44
MeO
N
N
N
p-Tol
H
MeO
S
S
N
O
NHBoc
(S2m, 600 MHz, CDCl3)
2.00 0.74 2.21 1.05 0.90 1.12 1.09 4.51 3.18 3.27 4.42 1.22 3.08 3.28 10.07
f1 (ppm)

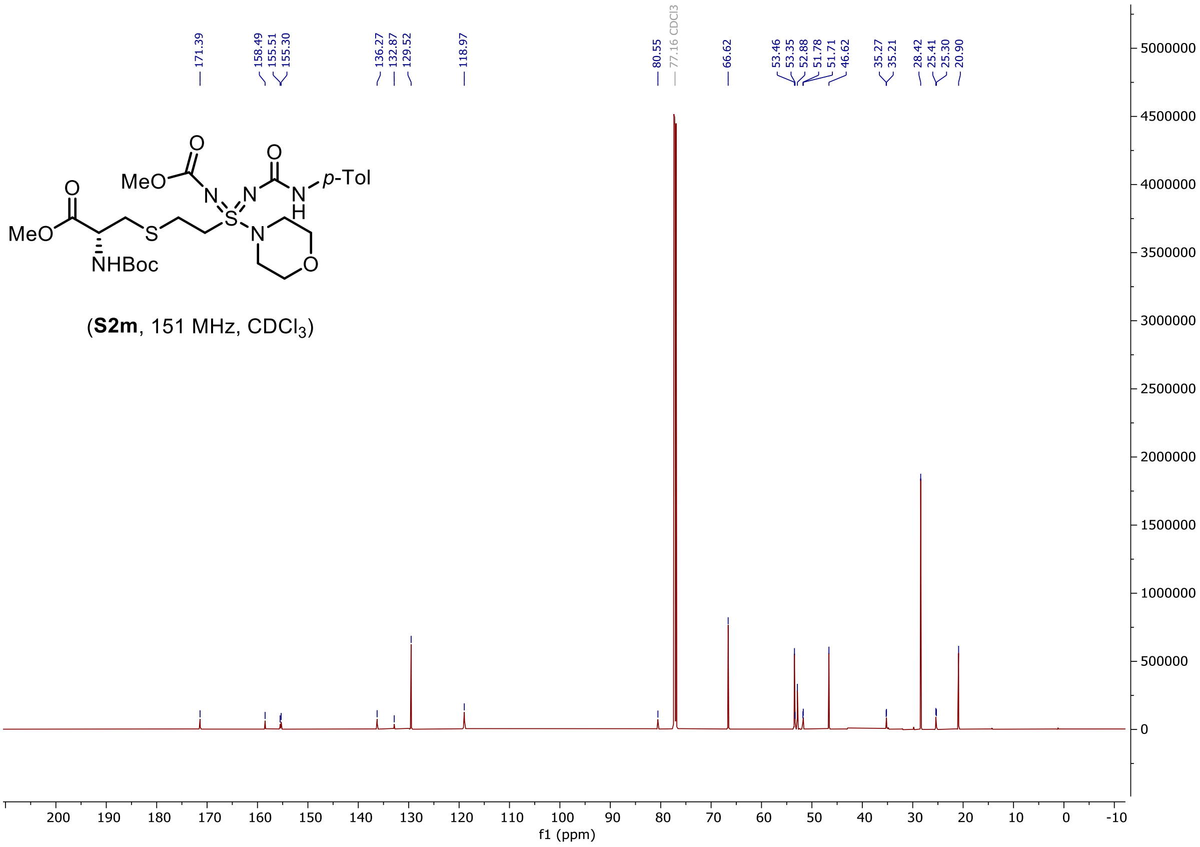
171.39 158.49 155.51 155.30 136.27 132.87 129.52 118.97 80.55 77.16 CDCl3 66.62 53.46 53.35 52.88 51.78 51.71 46.62 35.27 35.21 28.42 25.41 25.30 20.90
MeO
N
N
N
p-Tol
H
MeO
S
S
N
O
NHBoc
(S2m, 151 MHz, CDCl3)
f1 (ppm)
